# Association of Vitamins and Minerals with Type 1 Diabetes Risk: A Mendelian Randomization Study

**DOI:** 10.3390/nu17203297

**Published:** 2025-10-20

**Authors:** Lucia Shi, Wiame Belbellaj, Despoina Manousaki

**Affiliations:** 1 Azrieli Research Center of the Sainte-Justine University Hospital, Université de Montréal, Montreal, QC H3T 1C5, Canada; lucia.shi@mail.mcgill.ca (L.S.); wiame.belbellaj@umontreal.ca (W.B.); 2 Faculty of Medicine and Health Sciences, McGill University, Montreal, QC H4A 3T2, Canada; 3 Department of Pediatrics, Université de Montréal, Montreal, QC H3T 1C5, Canada; 4 Department of Biochemistry and Molecular Medicine, Université de Montréal, Montreal, QC H3C 3J7, Canada

**Keywords:** vitamins, micronutrients, potassium, type 1 diabetes, mendelian randomization, GWAS

## Abstract

**Background/Objectives:** Previous studies suggest that nutrient deficiencies can alter immune responses in animals. However, the impact of micronutrients on autoimmune diseases like type 1 diabetes (T1D) in humans remains unclear since the described associations are based on observational data and they cannot establish causality. This study aims to examine the causal relationship between various micronutrients and T1D using Mendelian randomization (MR). **Methods:** We performed a two-sample MR analysis using genetic variants from genome-wide association studies (GWASs) of 17 micronutrients as instrumental variables (IVs). We analyzed T1D GWAS datasets of European (18,942 cases/520,580controls), multi-ancestry (25,717 cases/583,311 controls), Latin American/Hispanic (2295 cases/55,134 controls), African American/Afro-Caribbean (6451 cases/109,410 controls), and East Asian (1219 cases/132,032 controls) ancestries. We applied the inverse variance weighted (IVW) method in our main analysis, and additional MR estimators (MR-Egger, weighted median, weighted mode, MR-PRESSO) to address pleiotropy, and the Steiger test to test directionality in sensitivity analyses. **Results:** Following Bonferroni correction (*p* < 0.05/17), we found positive association between potassium levels and T1D risk (OR = 1.098, 95% CI [1.075, 1.122] *p* = 5.5 × 10^−18^) in the multi-ancestry analysis. Zinc, vitamin B12, retinol, and alpha tocopherol showed nominal associations. Vitamin C, D, K1, B6, beta- and gamma-tocopherol, magnesium, iron, copper, selenium, carotene, and folate showed no significant effects on T1D risk. For the multi-ancestry analysis, we had sufficient power to detect ORs for T1D larger than 1.065. **Conclusions:** Higher serum potassium levels were associated with increased T1D risk in our MR study, though supporting observational evidence is currently limited. Other micronutrients are unlikely to have large effects on T1D.

## 1. Introduction

Type 1 diabetes, previously termed insulin-dependent diabetes, is a chronic autoimmune condition caused by the destruction of pancreatic beta cells. It leads to insulin deficiency and hyperglycemia, requiring lifelong insulin therapy to maintain glucose homeostasis [1]. Genetic and environmental factors influence the development of T1D, though their precise contributions remain uncertain [2]. Among these risk factors, deficiencies in micronutrients have been associated with an increased risk of onset of T1D in observational studies [3,4]. Micronutrients, such as vitamins and minerals, are essential compounds required in small amounts for physiological functions. They have anti-inflammatory and antioxidant properties [5] that may be protective against T1D [6].

Indeed, prior reports suggest that supplementation of micronutrients like magnesium, zinc, and vitamins C, E, and D, may lower T1D risk by modulating oxidative stress and enhancing immune function [3,7,8,9]. However, conflicting studies report no association between serum levels of these micronutrients and T1D diagnosis [10,11,12,13]. Other vitamins, such as vitamin A, B6, B12, and K, have been less extensively studied in relation to T1D, but show potential in modulating its risk. Vitamin A supports pancreatic β-cell development and insulin production, with animal studies indicating that its deficiency may reduce β-cell mass and insulin secretion [14]. Vitamin K shows protective effects against hyperglycemia and may enhance insulin secretion and β-cell proliferation in T1D animal models [15,16]. Potassium, another critical micronutrient, regulates β-cell membrane potential through ATP-sensitive potassium channels, and its dysregulation may impair insulin secretion [17]. Additional micronutrients, such as various trace elements, may also play a role in modulating pathways linked to T1D [18].

Given the observational nature of the studies on the role of micronutrients in T1D, more research is needed to confirm causal links and address challenges like unmeasured confounders. Mendelian randomization (MR) provides a powerful method to evaluate causal associations between exposures and outcomes by using genetic variants as instruments, thus reducing bias from confounding and reverse causality. Unlike observational studies, MR uses randomly inherited genetic variants, which are unaffected by environmental or lifestyle factors, to mimic a randomized controlled trial [19,20]. In this study, we employed two-sample MR to test if blood levels of 17 circulating micronutrients can causally impact the risk of developing T1D.

## 2. Materials and Methods

### 2.1. MR Study Design

**Figure 1:**
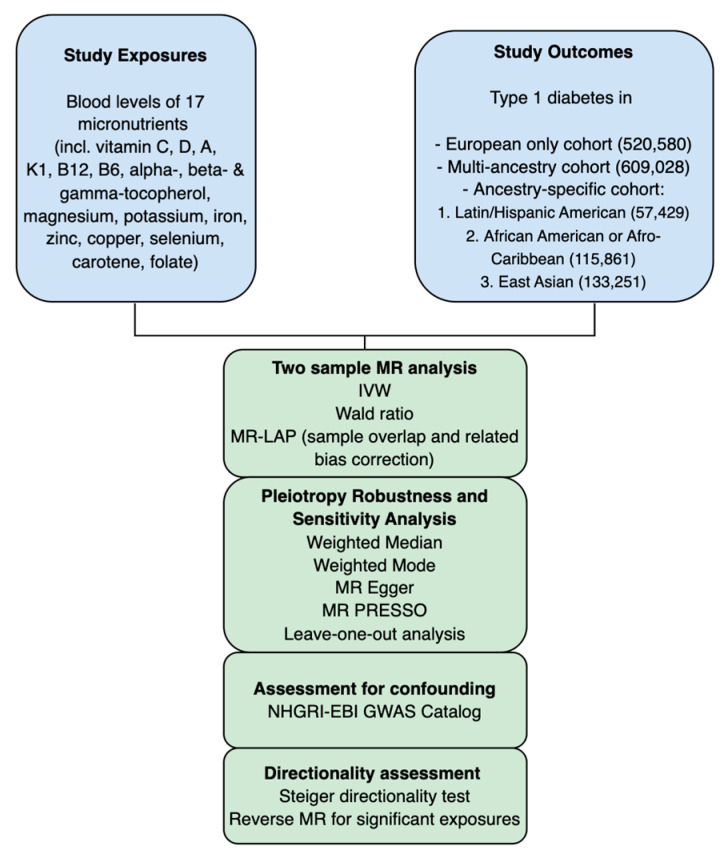
Flowchart with the design of our MR study.

**Figure 2:**
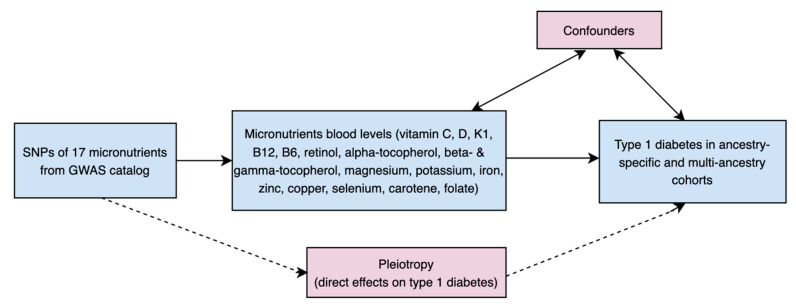
Direct acyclic graph (DAG) of our MR study.

A flowchart outlining our study appears in Figure 1, and a direct acyclic graph of the main MR study is shown in Figure 2. To analyze the causal effect of circulating micronutrients levels on T1D, two-sample MR analyses were performed using single nucleotide polymorphisms (SNPs) significantly associated with levels of these 17 micronutrients in genome-wide association studies (GWAS) as IVs.

Our MR analyses respected the three core MR assumptions:

1. Relevance Assumption: SNPs must demonstrate a strong association with the exposure variable.
2. Independence Assumption: SNPs are not correlated with any confounding factors.
3. Exclusion Restriction Assumption: SNPs influence the outcome exclusively via the exposure.

### 2.2. Data Source

We identified SNPs associated with circulating levels of copper, selenium, and zinc from a GWAS by Evans et al. [21], which measured red blood cell concentrations of copper and zinc in 2603 individuals of European ancestry, and of selenium in 5477 individuals. SNPs associated with serum levels of other minerals, including magnesium, potassium, and iron, were obtained from Verma et al. [22], who conducted a large-scale GWAS on 2068 traits in up to 635,969 participants from the VA Million Veteran Program [23].

SNPs for serum levels of vitamins were also sourced from the GWAS Catalog (https://www.ebi.ac.uk/gwas/, accessed on 6 July 2025). Specifically, vitamin C-associated SNPs were derived from a GWAS by Zheng et al. [24], SNPs for retinol from Chen et al. [25], for vitamin B12 from Grarup et al. [26], for vitamin B6 from Tanaka et al. [27], for vitamin K from Dashti et al. [28], for alpha-tocopherol from Major et al. [29], and for beta- and gamma-tocopherol from Surendran et al. [30]. For the outcome data, we used SNPs associated with T1D from several GWAS. First, we used a European GWAS by Chiou et al. [31], which included 18,942 T1D cases and 520,580 controls across nine cohorts. Additional outcome data were obtained from the multi-ancestry, Hispanic/Latin American, and African American/Afro-Caribbean GWAS conducted by Verma et al. [22,23], as well as an East Asian-specific GWAS by Sakaue et al. [32]. Table S1 provides more details and characteristics of populations included in GWAS data on exposures and outcome.

### 2.3. Instrumental Variable (IV) Selection

To ensure the validity of our genetic instruments, we applied a series of filtering steps to the exposure data. To meet the relevance assumption, SNPs were selected based on strong association with each micronutrient level (GWAS *p*-value ≤ 5 × 10^−6^). We calculated the F-statistic for each SNP using a published formula [33]. We excluded SNPs located within the HLA region (chr6: 28.5–33.4 Mb) due to its extensive linkage disequilibrium and associations with multiple traits [34]. We also removed rare variants (MAF < 1% or >99%) and performed linkage disequilibrium (LD) clumping with R^2^ < 0.001 in a 10 Mb window using the 1000 Genomes reference panel to retain independent instruments. We excluded indels to reduce strand ambiguity and alignment errors. We retained SNPs that passed all quality control filters (Tables S4–S8). To minimize potential bias due to pleiotropy or confounding, we conducted a comprehensive search using the gwasrapidd package (v0.99.18) in R (v4.4.3). This tool queries the NHGRI-EBI GWAS Catalog to retrieve previously reported associations for each SNP. Through this process, we identified and excluded SNPs associated with T1D diagnosis or other autoimmune conditions that could act as confounders (Table S10). This step ensured that the selected IVs satisfy the MR assumption of independence from confounders, strengthening the robustness of our causal inference.

### 2.4. Mendelian Randomization Analysis

We performed univariate MR analyses using the TwoSampleMR R package (v0.6.15). Exposure and outcome datasets were harmonized to align effect alleles and ensure consistent orientation of SNP effects. The primary causal estimates were obtained using the inverse variance weighted (IVW) method, which combines SNP-specific ratio estimates weighted by the inverse of their variance. When only a single SNP was available for an exposure, the Wald ratio method was applied. To correct for bias due to sample overlap, in the analyses for potassium, magnesium, and iron as exposures sharing GWAS sources with T1D as outcome from Verma et al. [22], we applied the MR-Lap method (v0.0.3.3) to calculate an adjusted IVW estimate [35].

Robustness to horizontal pleiotropy was assessed using several sensitivity methods: weighted median, weighted mode, MR-Egger regression, and MR-PRESSO (v1.0). MR-Egger assessed whether its intercept differed significantly from zero, with a non-zero intercept indicating the presence of directional pleiotropy [36]. MR-PRESSO identified outlier variants and recalculated the estimates, excluding the outliers [37]. We generated and examined forest and leave-one-out sensitivity analysis plots to visually detect SNP outliers that may contribute to heterogeneity or horizontal pleiotropy among the genetic instruments and also used the Cochran’s Q test to estimate heterogeneity. Scatter plots were also generated for each MR analysis using ggplot2 (v3.5.2) in R (v4.4.3). These plots are displayed in Figures S1–S3.

To confirm the directionality of the observed associations, the Steiger directionality test was conducted. For exposures demonstrating significant causal effects, namely potassium, we also performed a reverse MR analysis using T1D as the exposure (from the Chiou et al. GWAS) and potassium as the outcome (Table S3). This was done to further validate the inferred causal direction and assess reverse causation (i.e., the possibility that T1D influences potassium levels rather than the reverse).

We calculated the statistical power for our MR analyses using the online tool “mRnd” set for binary outcomes (https://shiny.cnsgenomics.com/mRnd/, accessed on 18 July 2025). Parameters included a significance threshold of 0.05, the proportion of T1D cases reported in the GWAS, the R^2^ value (representing the proportion of variance in each exposure explained by its SNP instruments), and an OR reflecting the MR OR from our IVW results. We also estimated the smallest MR odds ratio to achieve 80% statistical power (Table S9).

Given that causal associations were tested across 17 exposures, we applied a Bonferroni correction to account for multiple testing and reduce the likelihood of type I errors. The significance threshold was thus adjusted to 0.0029 (0.05/17). Associations with *p*-values below this threshold were considered statistically significant, while nominal associations between 0.0029 and 0.05 were regarded as suggestive.

## 3. Results

### 3.1. MR Results

We assessed potential causal links between micronutrients and T1D through univariable Mendelian randomization analyses. The large F statistics of the used SNP-IVs suggested that the analyses were not affected by weak instrument bias (Tables S4–S8). Detailed results of the main MR analyses and sensitivity analyses appear in Table S2.

**Figure 3:**
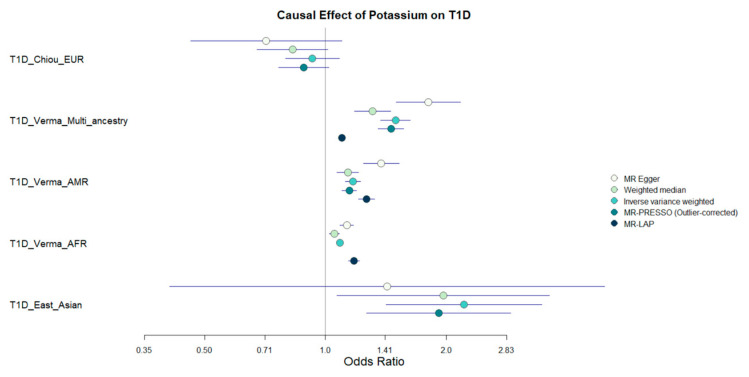
Forest plot with the results of the MR analysis on the effect of serum potassium levels on T1D risk across five ancestry-stratified GWAS cohorts. ORs with 95% CI are presented for each population.

Among the 17 micronutrients tested, potassium was the only exposure for which we observed significant associations with multiple MR estimators (Table S2, Figure 3). SNPs identified as associated with known confounders or T1D-related traits were excluded from the analysis and highlighted in red in Table S10. Using the MR-Lap method to adjust for sample overlap, 296 potassium SNPs were retained as instruments in the Multi-ancestry MR. The average F-stat of these SNPs was 45 and the cumulative R^2^ of the potassium levels explained by the SNPs was 0.03614. Potassium levels were positively associated with T1D risk in the multi-ancestry, Latin American/Hispanic, and African American/Afro-Caribbean analyses, (OR Multi-ancestry MR = 1.098, 95% CI [1.075, 1.122], *p* = 5.52 × 10^−18^; OR Latin American/Hispanic Cohort = 1.265, 95% CI [1.208, 1.324], *p* = 8 × 10^−24^; OR African American/Afro-Caribbean cohort: MR = 1.176, 95% CI [1.139, 1.215], *p* = 4.24 × 10^−23^). In some analyses, there was evidence of pleiotropy based on the MR-Egger intercept *p*-value and of heterogeneity according to the Cochran’ Q test (Table S2), but the pleiotropy-robust MR methods (weighted median, weighted mode, MR-PRESSO) consistently supported the positive association between genetically predicted potassium levels and T1D risk. For the East Asian population, the association was only significant (OR = 1.915, 95% CI [1.265, 2.897], *p* = 0.00262) in the MR-PRESSO outlier-corrected analysis. In contrast, potassium was not significantly associated with T1D in the European cohort. Forest and scatter plots of the MR analyses are available in Supplementary Figures S1 and S2.

These results suggest that, while pleiotropy and heterogeneity may have influenced our IVW estimates, the overall consistent findings across diverse populations and analytical approaches support a causal role for elevated potassium levels in increasing T1D susceptibility. The Steiger directionality test did not indicate evidence of reverse causation in our MR analyses, and no significant reverse causal effects were observed, suggesting that potassium levels are unlikely to be influenced by T1D diagnosis (Table S3).

We performed a power analysis to assess the minimum effects of the various nutrients on T1D that our MR study could detect across different ancestral cohorts. In the European MR analysis for potassium, we had sufficient power to detect ORs for T1D as small as 1.073. In the multi-ancestry analysis, the smallest detectable OR for 80% power was 1.065. For the Hispanic/Latin American analysis, it was 1.21. For the African American/Afro-Caribbean analysis, it was 1.125, and for the East Asian analysis, it was 1.28. These findings suggest that while our study was well-powered in the European and multi-ancestry analyses, the power was more limited in non-European cohorts, meaning that smaller effects may have gone undetected. The full results of the power analysis are presented in Table S9.

### 3.2. Suggestive MR Associations

Several micronutrients showed nominally significant associations with T1D risk in our MR analysis (Table S2). Higher genetically predicted alpha-tocopherol levels were associated with a decreased risk of T1D (OR = 0.26, 95% CI [0.095, 0.71], *p* = 0.009), as were zinc levels (OR = 0.92, 95% CI [0.85, 0.99], *p* = 0.023). In contrast, retinol (OR = 1.18, 95% CI [1.01, 1.38], *p* = 0.039) and vitamin B12 (OR = 1.03, 95% CI [1.01, 1.05], *p* = 0.012) were associated with increased T1D risk. However, none of these associations remained statistically significant after correction for multiple testing.

The MR-STROBE checklist of our MR study appears in Table S11.

## 4. Discussion

In this study, we used univariate MR to assess the potential causal relationship between circulating vitamins and minerals and T1D risk across multiple ancestries. Among the 17 micronutrients examined, potassium was the only exposure showing a significant association across multiple MR estimators and surviving multiple testing correction. Our results suggest that genetically elevated potassium levels are linked to an increased risk of developing T1D in non-European cohorts. This finding adds to a small body of literature exploring the role of ion homeostasis in autoimmune diseases and provides novel insights into potential mechanisms underlying T1D pathogenesis.

Although very few studies have directly examined potassium’s role in T1D, its known effects on immune regulation, insulin secretion, and β-cell function suggest several biologically plausible pathways linking it to disease risk. Potassium plays a critical role in maintaining cellular membrane potential and regulating ion channels, which are essential for immune cell activation and insulin secretion from the pancreatic β-cell [38]. In the context of T1D, an autoimmune disease characterized by T-cell-mediated destruction of insulin-producing β-cells [2], potassium dysregulation could influence immune responses or β-cell vulnerability, potentially contributing to disease risk.

In immune cells, potassium channels, particularly voltage-gated Kv1.3 channels, play an important role in T-cell activation and proliferation. Kv1.3 channels are highly expressed in effector memory T cells implicated in various autoimmune diseases, including T1D [39]. Experimental studies in non-obese diabetic (NOD) mice suggest that modulation of potassium signaling can influence the balance of proinflammatory T-cell responses, such as Th1 polarization and interferon-γ (IFN-γ) production, which drive insulitis and β-cell destruction [40,41]. Elevated extracellular potassium levels may indirectly enhance Kv1.3 channel activity by altering membrane potential, thus amplifying autoreactive immune responses.

Potassium also influences pancreatic β-cell function, a key target in T1D. ATP-sensitive potassium (KATP) channels in β-cells regulate membrane depolarization, controlling calcium influx and insulin secretion [17]. Hypokalemia (low levels of potassium) is known to impair insulin secretion by hyperpolarizing β-cells and reducing their sensitivity to glucose. A study showed that potassium depletion impairs insulin release in healthy volunteers, while supplementation restores it, highlighting potassium’s role in β-cell function [42]. Although less extensively studied, hyperkalemia (high levels of potassium) could have deleterious effects as well. Excessive depolarization from elevated potassium levels may disrupt normal KATP channel dynamics, promote β-cell stress, and increase susceptibility to autoimmune attack [17].

Our findings align with a broader literature on potassium signaling in other autoimmune conditions. For example, in rheumatoid arthritis, elevated potassium concentrations in the synovial fluid have been associated with increased T-cell infiltration and inflammation [43,44]. Similarly, in systemic lupus erythematosus (SLE), modulation of potassium channels has been shown to influence T-cell signaling and disease severity [45]. These parallels suggest that dysregulated potassium homeostasis may have proinflammatory effects across a range of autoimmune diseases, including T1D. Also, although our focus is on T1D, findings from type 2 diabetes (T2D) offer context. In T2D, hypokalemia has been linked to impaired glucose tolerance [46].

Finally, the identified suggestive MR associations may reflect underlying biological roles of these micronutrients in T1D development. Alpha-tocopherol (vitamin E) and zinc are known to exert antioxidant and immunomodulatory effects, which could help protect pancreatic β-cells from oxidative and inflammatory damage [9,10]. In contrast, elevated retinol levels may contribute to immune activation, as excessive vitamin A has been associated with proinflammatory responses in some autoimmune contexts [47]. Vitamin B12 plays a key role in DNA methylation and immune cell function through one-carbon metabolism. These processes can influence T-cell differentiation and cytokine expression, potentially modulating autoimmune pathways involved in T1D [48]. Although these findings did not survive correction for multiple testing, they need to be replicated in future larger MR studies, since they may suggest plausible mechanisms worth studying.

While our findings suggest potassium as a novel biomarker of T1D risk, the mechanisms remain speculative due to limited previous evidence. Despite the biologically plausible pathways, our findings should be interpreted with caution. MR relies on several key assumptions, including the validity of the instrumental variables and the absence of horizontal pleiotropy. While we used multiple MR methods to strengthen causal inference and found consistent results, we cannot entirely exclude the possibility of residual confounding or pleiotropic effects. Genetic instruments for some micronutrients (copper, selenium and zinc) were associated with their red blood cell concentrations, while for the other micronutrients their serum levels were measured in the GWAS used to derive genetic instruments. These differences in the biological matrices used to measure the levels of the micronutrients may have influenced their GWAS associations and the identification of genetic instruments, and as a consequence the results of our MR analyses. Additionally, effects of the genetic instruments on the micronutrients were assessed in European GWAS since specific data on other ancestries are lacking, while their effects on T1D were sought in multi-ancestry datasets or non-European datasets. This may have introduced bias, due to ancestry acting as a confounder. However, our decision to include analyses on multiple ancestries enhances the generalizability of our findings Additionally, some of the GWAS used for micronutrient exposures were based on limited sample sizes, which may reduce the power of our MR analyses for certain nutrients. These constraints highlight the need for further replication in MR and mechanistic studies to validate our findings and explore the underlying mechanisms in greater depth.

## 5. Conclusions

This two-sample MR study indicates a potential causal relationship between higher genetically predicted potassium levels and increased risk of type 1 diabetes. While associations with alpha-tocopherol, zinc, vitamin B12, and retinol, did not remain significant after multiple testing correction, we cannot exclude the small effects of these and other of the tested micronutrients on T1D. These findings highlight the need for further research, validation MR studies using larger and more harmonized GWAS data for micronutrients, and notably follow-up studies to better understand the role of potassium in T1D development.

## Data Availability

All GWAS data used are publicly available through the GWAS catalog: https://www.ebi.ac.uk/gwas/home (accessed on 6 July 2025). All other data supporting the reported results can be found in the Supplementary Tables. The codes used for this study are available at https://github.com/lucia-sh/Micronutrients-T1D-MR-2025.git, accessed on 5 October 2025.

## Supplementary Materials

The following supporting information can be downloaded at: https://www.mdpi.com/article/10.3390/nu17203297/s1. Table S1: Description of the GWAS datasets; Table S2: Results of the MR analyses testing associations between the micronutrients and T1D; Table S3: Reverse MR with Chiou et al. T1D as exposure and potassium as outcome; Table S4: Harmonization results for exposure SNPs and Chiou et al. T1D outcome; Table S5: Harmonization results for exposure SNPs and Verma et al. (multi-ancestry) T1D outcome; Table S6: Harmonization results for exposure SNPs and Verma et al. (Hispanic/Latin American) T1D outcome; Table S7: Harmonization results for exposure SNPs and Verma et al. (African) T1D outcome; Table S8: Harmonization results for exposure SNPs and Sakaue et al. T1D outcome; Table S9: Power analysis for the Two-Sample MR Analysis; Table S10: SNP-Trait Associations in Potassium GWAS; Table S11: MR-STROBE checklist; Figure S1: Scatter plot of MR analysis for micronutrients on the T1D outcomes; Figure S2: Forest plots of MR analysis for micronutrients on the T1D outcomes; Figure S3: Leave-one-out sensitivity analysis plot of MR analysis for micronutrients on the T1D outcomes. Reference [49] is cited in the Supplementary Materials.
