# Supplementary material for: Association of Vitamins and Minerals with Type 1 Diabetes Risk: A Mendelian Randomization Study
Source: Nutrients. 2025 Oct 20;17(20):3297. doi: 10.3390/nu17203297 (PMC12566610; doi:10.3390/nu17203297)
Supplement: Supplementary file 1 [file nutrients-17-03297-s001.zip › Supplement table S11.pdf]

Table S11: MR-STROBE checklist[1 2]

Item No.	Section	Checklist item	Page No.	Relevant text from manuscript
1	**TITLE and ABSTRACT**	Indicate Mendelian randomization (MR) as the study's design in the title and/or the abstract if that is a main purpose of the study	1	We performed a two-sample MR analysis using genetic variants from genome-wide association studies (GWAS) of 17 micronutrients as instrumental variables (IVs).
	**INTRODUCTION**			
2	**Background**	Explain the scientific background and rationale for the reported study. What is the exposure? Is a potential causal relationship between exposure and outcome plausible? Justify why MR is a helpful method to address the study question	2	Prior reports suggest that supplementation of micronutrients like magnesium and zinc, and vitamins C, E, and D, may lower types 1 diabetes (T1D) risk by modulating oxidative stress and enhancing immune function [3,7-9]. However, conflicting studies report no association between serum levels of these micronutrients and T1D diagnosis [10-13].<br>Given the uncertainty surrounding the role of micronutrients in T1D, more research is needed to confirm causal links and address challenges like unmeasured confounders.<br>Mendelian randomization provides a powerful method to evaluate causal links between exposures and outcomes by using genetic variants as instruments, thus reducing bias from confounding and reverse causality. Unlike observational studies, MR uses randomly inherited genetic variants, which are unaffected by environmental or lifestyle factors, to mimic a randomized controlled trial [19,20].
3	**Objectives**	State specific objectives clearly, including pre-specified causal hypotheses (if any). State that MR is a method that, under specific assumptions, intends to estimate causal effects	2	In this study, we employed two-sample MR to test if blood levels of 17 circulating micronutrients have a causal impact on the risk of developing T1D.
	**METHODS**			
4	**Study design and data sources**	Present key elements of the study design early in the article. Consider including a table listing sources of data for all phases of the study. For each data source contributing to the analysis, describe the following:	2-3	See Figure 1 & 2

		a)	Setting: Describe the study design and the underlying population, if possible. Describe the setting, locations, and relevant dates, including periods of recruitment, exposure, follow-up, and data collection, when available.	3-4	To analyze the causal effect of circulating micronutrients levels on T1D, a two-sample MR analysis was performed using single nucleotide polymorphisms (SNPs) as IVs for the different 17 micronutrients.<br>Figure 1 provides a summary of the design used for the study.<br>We extracted available genetic IVs from five T1D datasets. We extracted available genetic instruments from five T1D GWAS datasets. Summary-level data for all SNPs were obtained from the available GWAS reporting associations with various micronutrient levels. To assess potential causal effects, we conducted univariable two-sample Mendelian randomization (MR) analyses and multiple sensitivity analyses.
		b)	Participants: Give the eligibility criteria, and the sources and methods of selection of participants. Report the sample size, and whether any power or sample size calculations were carried out prior to the main analysis	3-4	See Supplementary table 1
		c)	Describe measurement, quality control and selection of genetic variants	4	SNPs were selected based on strong association with each micronutrient in the exposure (p-value $\leq 5 \times 10^{-6}$). We calculated the F-statistic for each SNP and kept only those with $F > 10$ to reduce weak instrument bias.<br>We excluded SNPs located within the HLA region. We also removed rare variants (MAF < 1% or > 99%) and performed linkage disequilibrium (LD) clumping with $R^2 < 0.001$ in a 10 Mb window using the 1000 Genomes reference panel to retain independent instruments. We excluded indels to reduce strand ambiguity and alignment errors. We retained SNPs that passed all quality control filters (Tables S4–S8).
		d)	For each exposure, outcome, and other relevant variables, describe methods of assessment and diagnostic criteria for diseases	3-4	See Supplementary table 1
		e)	Provide details of ethics committee approval and participant informed consent, if relevant		Not applicable
5	**Assumptions**		Explicitly state the three core IV assumptions for the main analysis (relevance, independence and exclusion restriction) as well assumptions for any additional or sensitivity analysis	3	The MR analysis was built on three core assumptions:

					1. Relevance Assumption: SNPs must demonstrate a strong association with the exposure variable.<br>2. Independence Assumption: SNPs are not correlated with any confounding factors.<br>3. Exclusion Restriction Assumption: SNPs influence the outcome exclusively via exposure.
6	**Statistical methods: main analysis**		Describe statistical methods and statistics used		
		a)	Describe how quantitative variables were handled in the analyses (i.e., scale, units, model)	4-6	Using MR methods and OR
		b)	Describe how genetic variants were handled in the analyses and, if applicable, how their weights were selected	4-6	1. SNPs must reach genome-wide significance $P < 5\times10^{-6}$<br>2. Clumping technique<br>3. Harmonise
		c)	Describe the MR estimator (e.g. two-stage least squares, Wald ratio) and related statistics. Detail the included covariates and, in case of two-sample MR, whether the same covariate set was used for adjustment in the two samples	4-6	We used the inverse-variance weighted (IVW) method as the primary MR estimator. For exposures with a single SNP, the Wald ratio was applied. Sensitivity analyses included MR-Egger, weighted median, weighted mode and MR-Lap.
		d)	Explain how missing data were addressed		No
		e)	If applicable, indicate how multiple testing was addressed	5	Given that causal associations were tested across 17 exposures, we applied a Bonferroni correction to account for multiple testing and reduce the likelihood of type I errors. The significance threshold was thus adjusted to 0.0029 (0.05/17).
7	**Assessment of assumptions**		Describe any methods or prior knowledge used to assess the assumptions or justify their validity	4-5	IVW, MR-Egger, weighted median
8	**Sensitivity analyses and additional analyses**		Describe any sensitivity analyses or additional analyses performed (e.g. comparison of effect estimates from different approaches, independent replication, bias analytic techniques, validation of instruments, simulations)	4-5	To correct for bias due to sample overlap, we applied the MR-Lap method.<br>Robustness to horizontal pleiotropy was assessed using several sensitivity methods: weighted median, weighted mode, MR-Egger regression, and MR-PRESSO (v1.0). MR-Egger assessed directional pleiotropy by examining whether the intercept differed significantly from zero, with a non-zero intercept indicating the presence of directional pleiotropy [36]. MR-PRESSO identified

					outlier variants and recalculated the estimates, excluding the outliers [37]. We generated and examined forest and leave-one-out sensitivity analysis plots to visually detect SNP outliers that may contribute to heterogeneity or horizontal pleiotropy among the genetic instruments.<br>To confirm the directionality of the observed associations, the Steiger directionality test was conducted. For exposures demonstrating significant causal effects, namely potassium, we also performed a reverse MR analysis using T1D as the exposure (from the Chiou et al. GWAS) and potassium as the outcome
9	**Software and pre-registration**				
		a)	Name statistical software and package(s), including version and settings used	4-5	We conducted a comprehensive search using the gwasrapidd package (v0.99.18) in R (v4.4.3)<br>All analyses were carried out with the<br>packages “TwoSampleMR” (v0.6.15), “MR-PRESSO” (v1.0), “MR-Lap” (v0.0.3.3) of R (4.4.3)<br>Plots were generated using ggplot2 (v3.5.2)
		b)	State whether the study protocol and details were pre-registered (as well as when and where)		No
	**RESULTS**				
10	**Descriptive data**				
		a)	Report the numbers of individuals at each stage of included studies and reasons for exclusion. Consider use of a flow diagram	4	Supplementary Table 1 provides more details and characteristics of populations included in GWAS data on exposure and outcome.
		b)	Report summary statistics for phenotypic exposure(s), outcome(s), and other relevant variables (e.g. means, SDs, proportions)	4	These traits are detailed in previous<br>studies.
		c)	If the data sources include meta-analyses of previous studies, provide the assessments of heterogeneity across these studies	5	Our study used summary statistics from previously published GWAS. We relied on the original GWAS publications’ quality control and heterogeneity evaluations.
		d)	For two-sample MR:	3-4	The micronutrient GWAS data were mostly derived from individuals of European ancestry. For T1D, we used summary statistics from five cohorts,

			i. Provide justification of the similarity of the genetic variant-exposure associations between the exposure and outcome samples<br>ii. Provide information on the number of individuals who overlap between the exposure and outcome studies		including three ethnicity-specific cohorts, one multi-ancestry cohort, and one European ancestry cohort. While the ancestral diversity in the T1D cohorts introduces some heterogeneity, the majority overlap with European populations supports reasonable similarity in genetic variant-exposure associations. Sensitivity analyses were conducted to assess the robustness of findings across these diverse cohorts.<br>To correct for bias due to sample overlap, specifically for potassium, magnesium, and iron exposures sharing GWAS sources with the multi-ancestry and ethnic-specific outcomes from Verma et al. [22], we applied the MR-Lap method (v0.0.3.3).
11	**Main results**				
		a)	Report the associations between genetic variant and exposure, and between genetic variant and outcome, preferably on an interpretable scale	5	We assessed potential causal links between micronutrients and T1D through univariable Mendelian randomization analyses, using harmonized SNP instruments and their GWAS-derived exposure estimates (Tables S4–S8).
		b)	Report MR estimates of the relationship between exposure and outcome, and the measures of uncertainty from the MR analysis, on an interpretable scale, such as odds ratio or relative risk per SD difference	5-6	Among the 17 micronutrients tested, potassium was the only exposure for which we observed statistically significant associations after multiple MR methods (Table S2, Figure 3). Using the MR-Lap method, potassium levels were positively associated with T1D risk in the multi-ancestry, Latin American/Hispanic, and African American/Afro-Caribbean cohorts, with odds ratios (ORs) consistently above 1 and highly significant p-values:<br>Multi-ancestry cohort: OR = 1.098, 95% CI [1.075, 1.122], $p = 5.52 \times 10^{-18}$<br>Latin American/Hispanic cohort: OR = 1.265, 95% CI [1.208, 1.324], $p = 8 \times 10^{-24}$<br>African American/Afro-Caribbean cohort: OR = 1.176, 95% CI [1.139, 1.215], $p = 4.24 \times 10^{-23}$<br>For the East Asian population, the association was also statistically significant (OR = 1.915, 95% CI [1.265, 2.897], p = 0.00262), based on the MR-PRESSO outlier-corrected results<br>See Figure 3

		c)	If relevant, consider translating estimates of relative risk into absolute risk for a meaningful time period		No
		d)	Consider plots to visualize results (e.g. forest plot, scatterplot of associations between genetic variants and outcome versus between genetic variants and exposure)	5-6	The plots (forest plot, scatterplot) are available in Supplementary Figures S1, S2, S3, S4
12	**Assessment of assumptions**				
		a)	Report the assessment of the validity of the assumptions	5-6	The large F statistics suggested that the analyses would not be affected by weak instrument bias (Table S4-S8)<br>SNPs identified as associated with known confounders or T1D-related traits were excluded from the analysis. They are highlighted in red in Supplementary Table S10.<br>To detect and correct for horizontal pleiotropy, MR-PRESSO was applied to each MR analysis to identify outlier SNPs. MR-Egger intercept tests indicated minimal directional pleiotropy for significant associations. Steiger filtering confirmed correct causal directionality. Leave-one-out analyses assessed the influence of individual SNPs.<br>See supplementary table S2
		b)	Report any additional statistics (e.g., assessments of heterogeneity across genetic variants, such as I^2, Q statistic or E-value)		No
13	**Sensitivity analyses and additional analyses**				
		a)	Report any sensitivity analyses to assess the robustness of the main results to violations of the assumptions	6	Other MR methods, including MR Egger, weighted median, and weighted mode, generally showed directionally consistent estimates, although not all reached statistical significance. These consistent findings across diverse populations and analytical approaches strengthen the evidence for a potentially protective causal role of potassium in T1D pathogenesis.
		b)	Report results from other sensitivity analyses or additional analyses	6	The Steiger directionality test did not indicate evidence of reverse causation in our MR analyses,

				suggesting that potassium levels are unlikely to be influenced by T1D risk.
		c) Report any assessment of direction of causal relationship (e.g., bidirectional MR)	5-6	We performed a reverse MR analysis using T1D as the exposure (from the Chiou et al. GWAS) and potassium as the outcome.<br>No significant reverse causal effects were observed (Table S3).
		d) When relevant, report and compare with estimates from non-MR analyses		No
		e) Consider additional plots to visualize results (e.g., leave-one-out analyses)	4-5	We generated and examined leave-one-out sensitivity analysis plots to visually detect SNP outliers that may contribute to heterogeneity or horizontal pleiotropy among the genetic instruments.<br>See Supplementary Figures S3.
	**DISCUSSION**			
14	**Key results**	Summarize key results with reference to study objectives	7	In this study, we used univariate MR to assess the potential causal relationship between circulating micronutrient levels and T1D risk. Among the 17 micronutrients examined, potassium was the only exposure with a statistically significant association across multiple MR methods.
15	**Limitations**	Discuss limitations of the study, taking into account the validity of the IV assumptions, other sources of potential bias, and imprecision. Discuss both direction and magnitude of any potential bias and any efforts to address them	6-8	although statistically significant associations were observed in most cohorts, the analyses were generally underpowered to detect small to moderate effect sizes.<br>While we used multiple MR methods to strengthen causal inference and found consistent results, we cannot entirely exclude the possibility of residual confounding or pleiotropic effects. Additionally, some of the GWAS used for micronutrient exposures were based on limited sample sizes, since the complete summary statistics were not provided, which may reduce the power or precision of our estimates for certain nutrients.
16	**Interpretation**			

		a) Meaning: Give a cautious overall interpretation of results in the context of their limitations and in comparison with other studies	7	Our results suggest that genetically elevated potassium levels are associated with increased risk of developing T1D.
		b) Mechanism: Discuss underlying biological mechanisms that could drive a potential causal relationship between the investigated exposure and the outcome, and whether the gene-environment equivalence assumption is reasonable. Use causal language carefully, clarifying that IV estimates may provide causal effects only under certain assumptions	7-8	In the context of T1D, an autoimmune disease characterized by T-cell-mediated destruction of insulin-producing β-cells [2], potassium dysregulation could influence immune responses or β-cell vulnerability, potentially contributing to disease risk.<br>Elevated extracellular potassium levels may indirectly enhance Kv1.3 channel activity by altering membrane potential, thus amplifying autoreactive immune responses.<br>Although less extensively studied, hyperkalemia (high levels of potassium) could have deleterious effects as well. Excessive depolarization from elevated potassium levels may disrupt normal KATP channel dynamics, promote β-cell stress, and increase susceptibility to autoimmune attack [17].<br>dysregulated potassium homeostasis may have proinflammatory effects across a range of autoimmune diseases, including T1D.<br>The suggestive associations, such as alpha-tocopherol, zinc, retinol, and vitamin B12, may influence T1D development through antioxidant, immunomodulatory, or epigenetic mechanisms, highlighting biologically plausible pathways despite results not surviving multiple testing correction.
		c) Clinical relevance: Discuss whether the results have clinical or public policy relevance, and to what extent they inform effect sizes of possible interventions	8	Future studies are needed to validate our findings and explore the underlying mechanisms in greater depth. Experimental models that manipulate potassium levels in immune and pancreatic tissues could clarify causal pathways. In addition, large-scale epidemiological studies examining serum potassium in early life or in at-risk populations may help to determine whether potassium status influences T1D onset or progression.
17	**Generalizability**	Discuss the generalizability of the study results (a) to other populations, (b) across other exposure periods/timings, and (c) across other levels of exposure		No

	**OTHER INFORMATION**			
18	**Funding**	Describe sources of funding and the role of funders in the present study and, if applicable, sources of funding for the databases and original study or studies on which the present study is based	9	
19	**Data and data sharing**	Provide the data used to perform all analyses or report where and how the data can be accessed, and reference these sources in the article. Provide the statistical code needed to reproduce the results in the article, or report whether the code is publicly accessible and if so, where	9	All GWAS data used are publicly available through the GWAS catalog: https://www.ebi.ac.uk/gwas/home (accessed on 6 July 2025). All other data supporting the reported results can be found in the supplementary tables. The codes used for this study are available at https://github.com/lucia-sh/Micronutrients-T1D-MR-2025.git
20	**Conflicts of Interest**	All authors should declare all potential conflicts of interest	9	The authors declare no conflict of interest.

This checklist is copyrighted by the Equator Network under the Creative Commons Attribution 3.0 Unported (CC BY 3.0) license.

1. Skrivankova VW, Richmond RC, Woolf BAR, Yarmolinsky J, Davies NM, Swanson SA, et al. Strengthening the Reporting of Observational Studies in Epidemiology using Mendelian Randomization (STROBE-MR) Statement. JAMA. 2021;under review.

2. Skrivankova VW, Richmond RC, Woolf BAR, Davies NM, Swanson SA, VanderWeele TJ, et al. Strengthening the Reporting of Observational Studies in Epidemiology using Mendelian Randomisation (STROBE-MR): Explanation and Elaboration. BMJ. 2021;375:n2233.
