# Supplementary material for: Association of Vitamins and Minerals with Type 1 Diabetes Risk: A Mendelian Randomization Study
Source: Nutrients. 2025 Oct 20;17(20):3297. doi: 10.3390/nu17203297 (PMC12566610; doi:10.3390/nu17203297)
Supplement: Supplementary file 1 [file nutrients-17-03297-s001.zip › Supplemental Table S1-S10 (pdf version).pdf]

Table S1 : Description of the GWAS datasets

Exposure:

Trait	GWAS population	Sex distribution	Sample size	First author	Year	Method of measurement	Mean micronutrient level	GWAS link
Vitamin C	European-ancestry	54% Female, 46% Male	52018	Zheng, JS	2020	fluorometric assay, High-Performance Liquid	50.95 µmol/L	https://www.ebi.ac.uk/gwas/studies/GCST011816
Vitamin D	European-ancestry	50.2% Female, 49.8% Male	79366	Jiang, X	2018	Chemiluminescent Immunoassays (CLIA), Liq	0.05 µmol/L	https://www.ebi.ac.uk/gwas/studies/GCST005367
Retinol (vitamin a)	European-ancestry	52.3% Female, 47.7% Male	8247	Chen, Y	2023	LC-MS/MS	2.0 µmol/L	https://www.ebi.ac.uk/gwas/studies/GCST90200405
Vitamin K1 (3 models used, each	European-ancestry	53% Female, 47% Male	2138	Dashti, HS.	2014	LC-MS/MS	0.00145 µmol/L	https://www.ebi.ac.uk/gwas/studies/GCST002653
Vitamin B12	European-ancestry	64.6% Female, 35.4% Male	45576	Grarup, N	2013	CLIA	409 pmol/L	https://www.ebi.ac.uk/gwas/studies/GCST90277442
Vitamin B6	European-ancestry	57.4% Female, 42.6% Male	2934	Tanaka T	2009	LC-MS/MS	42.6 nmol/L	https://www.ebi.ac.uk/gwas/studies/GCST000358
Alpha-tocopherol	European-ancestry	74% Female, 26% Male	3767	Major, JM	2011	HPLC	16.2 mg/L	https://www.ebi.ac.uk/gwas/studies/GCST001142
Beta & gamma-tocopherol	European-ancestry	53% Female, 47% Male	14296	Surendran, P	2022	Nuclear Magnetic Resonance (NMR)	1.2 µmol/L	https://www.ebi.ac.uk/gwas/studies/GCST90245213
Magnesium	European-ancestry	7% Female, 93% Male	145571	Verma, A	2024	Inductively Coupled Plasma-Mass Spectrome	0.85 mmol/L	https://www.ebi.ac.uk/gwas/studies/GCST90475480
Potassium	Multi-ancestry (African Amer	8% Female, 92% Male	603756	Verma, A	2024	Automated clinical chemistry analyzers	4.3 mmol/L	https://www.ebi.ac.uk/gwas/studies/GCST90480654
Iron	Multi-ancestry (African Amer	8% Female, 92% Male	127,495	Verma, A	2024	Automated clinical chemistry analyzers	18.2 µmol/L	https://www.ebi.ac.uk/gwas/studies/GCST90479654
Zinc	European-ancestry	86% Female, 14% Male	2603	Evans, DM	2013	ICP-MS	13.7 µmol/L	https://www.ebi.ac.uk/gwas/studies/GCST002040
Copper	European-ancestry	86% Female, 14% Male	2603	Evans, DM	2014	ICP-MS	15.5 µmol/L	https://www.ebi.ac.uk/gwas/studies/GCST002041
Selenium	European-ancestry	93% Female, 7% Male	5477	Evans, DM	2015	ICP-MS	1.10 µmol/L	https://www.ebi.ac.uk/gwas/studies/GCST002039
Carotene	European-ancestry	52% Female, 48% Male	8256	Chen, Y	2023	LC-MS/MS	0.12 µmol/L	https://www.ebi.ac.uk/gwas/studies/GCST90200142
Folate	European-ancestry	65.2% Female, 34.8% Male	21708	Grarup, N	2013	CLIA	22.6 nmol/L	https://www.ebi.ac.uk/gwas/studies/GCST90277443
**Outcome:**								
Type 1 Diabetes	European-ancestry	49% Female, 51% Male	520,580	Chiou, J.	2021	Clinical diagnosis with autoantibody positivi	NA	https://www.ebi.ac.uk/gwas/studies/GCST90014023
Type 1 Diabetes	Multi-ancestry (African Amer	8% Female, 92% Male	609,028	Verma, A	2024	Clinical diagnosis and insulin dependence	NA	https://www.ebi.ac.uk/gwas/studies/GCST90479879
Type 1 Diabetes	Hispanic or Latin American	8% Female, 92% Male	57429	Verma, A	2025	Clinical diagnosis and insulin dependence	NA	https://www.ebi.ac.uk/gwas/studies/GCST90475659
Type 1 Diabetes	African American or Afro-Cari	8% Female, 92% Male	115861	Verma, A	2024	Clinical diagnosis and insulin dependence	NA	https://www.ebi.ac.uk/gwas/studies/GCST90475660
Type 1 Diabetes	East Asian	46.3% Female, 53.7% Male	133251	Sakaue, S	2021	Clinical diagnosis from past medical history a	NA	https://www.ebi.ac.uk/gwas/studies/GCST90018705

Table S2 : Results of the MR analyses testing associations between the micronutrients and T1D

Significant associations
Nominal associations

Outcome: Chiou, J (T1D)

Method	N of SNPs	OR	Beta	95% LL	95% UL	Standard Error	P-val	Steiger test	MR Egger intercept	MR Egger intercept p-value	Cochran-Q	Cochran-Q p-val
**Vitamin C**												
MR Egger	33	0.88367082	-0.1236707	0.628146681	1.243139769	0.17413822	0.48289699	TRUE	-0.002142345	0.8132669	82.36782	1.53E-06
Weighted median	33	0.84404095	-0.1695543	0.694952698	1.025113108	0.09916185	0.08728917	TRUE				
Inverse variance weighted	33	0.85439673	-0.1573596	0.702168714	1.039627308	0.10011323	0.11599308	TRUE			82.51862	2.43E-06
Weighted mode	33	0.87957626	-0.128315	0.719068314	1.075912236	0.10279791	0.22100447	TRUE				
MR-PRESSO (Outlier-corrected)	33	0.87992667	-0.1279167	0.737303345	1.050138938	0.09022407	0.16563271	NA				
**Vitamin D**	**N of SNPs**	**OR**	**Beta**	**95% LL**	**95% UL**	**Standard Error**	**P-val**	**Steiger test**	**MR Egger intercept**	**MR Egger intercept p-value**	**Cochran-Q**	**Cochran-Q p-val**
MR Egger	18	0.98524166	-0.0148683	0.653641469	1.485066615	0.209351	0.9442611	TRUE	-0.003262032	0.5897658	11.92755	0.748952
Weighted median	18	0.97064176	-0.0297978	0.68891425	1.367580106	0.1749187	0.864733	TRUE				
Inverse variance weighted	18	0.90509828	-0.0997117	0.685745091	1.194617234	0.1416008	0.4813237	TRUE			12.23029	0.7859958
Weighted mode	18	0.96570052	-0.0349015	0.70920523	1.314961398	0.1575045	0.8272726	TRUE				
MR-PRESSO (Outlier-corrected)	18	NA	NA	NA	NA	NA	NA	NA				
**Retinol (vitamin A)**	**N of SNPs**	**OR**	**Beta**	**95% LL**	**95% UL**	**Standard Error**	**P-val**	**Steiger test**	**MR Egger intercept**	**MR Egger intercept p-value**	**Cochran-Q**	**Cochran-Q p-val**
MR Egger	NA	NA	NA	NA	NA	NA	NA	NA	NA	NA		
Weighted median	NA	NA	NA	NA	NA	NA	NA	NA				
Inverse variance weighted	2	0.85244891	-0.159642	0.496111001	1.464730965	0.2761804	0.5632403	TRUE			6.584881	0.01028485
Weighted mode	NA	NA	NA	NA	NA	NA	NA	NA				
MR-PRESSO (Outlier-corrected)	NA	NA	NA	NA	NA	NA	NA	NA				
**Vitamin K (1st model)**	**N of SNPs**	**OR**	**Beta**	**95% LL**	**95% UL**	**Standard Error**	**P-val**	**Steiger test**	**MR Egger intercept**	**MR Egger intercept p-value**	**Cochran-Q**	**Cochran-Q p-val**
MR Egger	5	0.89006425	-0.1164616	0.651167985	1.216605214	0.15945204	0.5180233	TRUE	0.007739262	0.8434319	4.782672	0.1884204
Weighted median	5	0.97193051	-0.028471	0.874289586	1.080475999	0.05401666	0.5981398	TRUE				
Inverse variance weighted	5	0.9195698	-0.0838493	0.844875468	1.000867754	0.04322281	0.0523879	TRUE			4.856482	0.3023428
Weighted mode	5	0.98363868	-0.0164966	0.858989152	1.126376345	0.06913385	0.8231275	TRUE				
MR-PRESSO (Outlier-corrected)	NA	NA	NA	NA	NA	NA	NA	NA				
**Vitamin K (2nd model)**	**N of SNPs**	**OR**	**Beta**	**95% LL**	**95% UL**	**Standard Error**	**P-val**	**Steiger test**	MR Egger intercept	**MR Egger intercept p-value**	**Cochran-Q**	**Cochran-Q p-val**
MR Egger	5	0.97748214	-0.0227753	-0.202353694	0.156803174	0.09162165	0.8197315	TRUE	-0.013652	0.5821224	5.543171	0.1360792
Weighted median	5	0.96904722	-0.0314419	-0.125409906	0.062526026	0.04794284	0.511939	TRUE				
Inverse variance weighted	5	0.93278116	-0.0695847	-0.161415227	0.022245907	0.04685233	0.1374932	TRUE			6.241775	0.1818053
Weighted mode	5	0.97517221	-0.0251412	-0.119598226	0.069315826	0.04819236	0.6294438	TRUE				
MR-PRESSO (Outlier-corrected)	NA	NA	NA	NA	NA	NA	NA	NA				
**Vitamin K (3rd model)**	**N of SNPs**	**OR**	**Beta**	**95% LL**	**95% UL**	**Standard Error**	**P-val**	**Steiger test**	MR Egger intercept	**MR Egger intercept p-value**	**Cochran-Q**	**Cochran-Q p-val**
MR Egger	4	0.9406594	-0.0611742	-0.201794262	0.079445942	0.07174495	0.483665	TRUE	0.003425932	0.8692188	1.221663	0.5428994
Weighted median	4	0.96821442	-0.0323017	-0.127721311	0.063117891	0.04868347	0.5070074	TRUE				
Inverse variance weighted	4	0.95122103	-0.0500088	-0.127553907	0.027536267	0.03956382	0.2062286	TRUE			1.256465	0.7394959
Weighted mode	4	0.97212599	-0.0282699	-0.144338218	0.087798498	0.05921855	0.6657129	TRUE				
MR-PRESSO (Outlier-corrected)	NA	NA	NA	NA	NA	NA	NA	NA				
**Vitamin B12**	**N of SNPs**	**OR**	**Beta**	**95% LL**	**95% UL**	**Standard Error**	**P-val**	**Steiger test**	**MR Egger intercept**	**MR Egger intercept p-value**	**Cochran-Q**	**Cochran-Q p-val**
MR Egger	9	1.16081975	0.14912644	0.657304243	2.050043821	0.29017078	0.6231243	TRUE	0.008608061	0.838043	53.61886	2.80E-09
Weighted median	9	1.03933771	0.03858369	0.89555243	1.206208406	0.07596847	0.6115299	TRUE				
Inverse variance weighted	9	1.22597513	0.20373655	0.958334157	1.568362145	0.12566087	0.1049489	TRUE			53.96358	7.02E-09
Weighted mode	9	1.00435621	0.00434675	0.838719385	1.202704284	0.09195196	0.9634553	TRUE				
MR-PRESSO (Outlier-corrected)	9	1.0048389	0.00482723	0.918694527	1.099060881	0.045729	0.9193673	NA				
**Vitamin B6**	**N of SNPs**	**OR**	**Beta**	**95% LL**	**95% UL**	**Standard Error**	**P-val**	**Steiger test**	**MR Egger intercept**	**MR Egger intercept p-value**	**Cochran-Q**	**Cochran-Q p-val**
MR Egger	NA	NA	NA	NA	NA	NA	NA	NA	NA	NA	NA	NA
Weighted median	NA	NA	NA	NA	NA	NA	NA	NA				
Inverse variance weighted	NA	NA	NA	NA	NA	NA	NA	NA				
Weighted mode	NA	NA	NA	NA	NA	NA	NA	NA				
MR-PRESSO (Outlier-corrected)	NA	NA	NA	NA	NA	NA	NA	NA				
Wald Ratio	1	1.01077	0.01071241	0.991783021	1.030120464	0.009675172	0.26820465	TRUE				
**Alpha-tocopherol (vitamin E)**	**N of SNPs**	**OR**	**Beta**	**95% LL**	**95% UL**	**Standard Error**	**P-val**	**Steiger test**	**MR Egger intercept**	**MR Egger intercept p-value**	**Cochran-Q**	**Cochran-Q p-val**
MR Egger	NA	NA	NA	NA	NA	NA	NA	NA	NA	NA	NA	NA
Weighted median	NA	NA	NA	NA	NA	NA	NA	NA				
Inverse variance weighted	NA	NA	NA	NA	NA	NA	NA	NA				
Weighted mode	NA	NA	NA	NA	NA	NA	NA	NA				
MR-PRESSO (Outlier-corrected)	NA	NA	NA	NA	NA	NA	NA	NA				
Wald Ratio	1	0.26017521	-1.3464	0.094780461	0.714188747	0.5152	0.008965837	TRUE				
**Gamma and beta-tocopherol (vitamin E)**	**N of SNPs**	**OR**	**Beta**	**95% LL**	**95% UL**	**Standard Error**	**P-val**	**Steiger test**	**MR Egger intercept**	**MR Egger intercept p-value**	**Cochran-Q**	**Cochran-Q p-val**
MR Egger	NA	NA	NA	NA	NA	NA	NA	NA	NA	NA	NA	NA
Weighted median	NA	NA	NA	NA	NA	NA	NA	NA				
Inverse variance weighted	NA	NA	NA	NA	NA	NA	NA	NA				
Weighted mode	NA	NA	NA	NA	NA	NA	NA	NA				
MR-PRESSO (Outlier-corrected)	NA	NA	NA	NA	NA	NA	NA	NA				
Wald Ratio	1	1.10363249	0.098607	0.82792145	1.471159692	0.1466551	0.5013457	TRUE				
**Magnesium**	**N of SNPs**	**OR**	**Beta**	**95% LL**	**95% UL**	**Standard Error**	**P-val**	**Steiger test**	**MR Egger intercept**	**MR Egger intercept p-value**	**Cochran-Q**	**Cochran-Q p-val**
MR Egger	81	1.37002017	0.31482546	1.009145126	1.859945819	0.15598056	0.0469473	TRUE	-0.0176771	0.008459147	119.8663	0.002072452
Weighted median	81	1.03094609	0.03047691	0.877021221	1.211886104	0.08250051	0.7118185	TRUE				
Inverse variance weighted	81	0.93045906	-0.0720772	0.820606423	1.055017412	0.06409922	0.2608166	TRUE			130.9345	0.000285935
Weighted mode	81	1.03498468	0.03438662	0.844469301	1.268481018	0.10379261	0.7412833	TRUE				
MR-PRESSO	81	0.97859635	-0.021636	0.883848065	1.083501627	0.05195616	0.678099	NA				
**Potassium**	**N of SNPs**	**OR**	**Beta**	**95% LL**	**95% UL**	**Standard Error**	**P-val**	**Steiger test**	**MR Egger intercept**	**MR Egger intercept p-value**	**Cochran-Q**	**Cochran-Q p-val**
MR Egger	259	0.71145943	-0.3404369	0.461107628	1.097736153	0.22126883	0.12514035	TRUE	0.007632828	0.1582419	435.7166	3.80E-11
Weighted median	259	0.82710364	-0.1898253	0.67424871	1.014611408	0.10425049	0.06862856	TRUE				
Inverse variance weighted	259	0.92730735	-0.0754702	0.794513965	1.082295552	0.07885434	0.33852496	TRUE			439.0854	2.46E-11
Weighted mode	259	0.87080993	-0.1383316	0.613395353	1.236249875	0.17878268	0.43979214	TRUE				
MR-PRESSO (Outlier-corrected)	259	0.88250027	-0.1249962	0.763406189	1.02017346	0.0739637	0.09222376	NA				
**Iron**	**N of SNPs**	**OR**	**Beta**	**95% LL**	**95% UL**	**Standard Error**	**P-val**	**Steiger test**	**MR Egger intercept**	**MR Egger intercept p-value**	**Cochran-Q**	**Cochran-Q p-val**
MR Egger	20	1.089189	0.08543338	0.85213788	1.39218394	0.12522465	0.5037722	TRUE	-0.01519493	0.1816485	43.528	0.000673561
Weighted median	20	0.98310151	-0.0170429	0.845228481	1.14346428	0.07709459	0.825042	TRUE				
Inverse variance weighted	20	0.95776939	-0.0431483	0.808559404	1.134514299	0.08640453	0.6175153	TRUE			48.19648	0.00024028
Weighted mode	20	1.02286315	0.0226057	0.899717955	1.162863326	0.0654488	0.7335919	TRUE				
MR-PRESSO (Outlier-corrected)	20	0.98406525	-0.0160631	0.859268357	1.12698718	0.06918925	0.8190318	NA				
**Zinc**	**N of SNPs**	**OR**	**Beta**	**95% LL**	**95% UL**	**Standard Error**	**P-val**	**Steiger test**	**MR Egger intercept**	**MR Egger intercept p-value**	**Cochran-Q**	**Cochran-Q p-val**
MR Egger	6	1.03870036	0.03797028	0.767395222	1.405922804	0.15445082	0.81790486	TRUE	-0.02566629	0.452218	2.649254	0.6181233
Weighted median	6	0.90991623	-0.0944027	0.827961699	0.999982903	0.04815594	0.04995433	TRUE				
Inverse variance weighted	6	0.91712443	-0.0865121	0.850795828	0.98862406	0.03830152	0.02390126	TRUE			3.341403	0.6475091
Weighted mode	6	0.89517039	-0.1107412	0.796092816	1.006578642	0.05984607	0.12347982	TRUE				
MR-PRESSO (Outlier-corrected)	NA	NA	NA	NA	NA	NA	NA	NA				
**Copper**	**N of SNPs**	**OR**	**Beta**	**95% LL**	**95% UL**	**Standard Error**	**P-val**	**Steiger test**	**MR Egger intercept**	**MR Egger intercept p-value**	**Cochran-Q**	**Cochran-Q p-val**
MR Egger	6	0.94403085	-0.0575964	0.789409372	1.128938018	0.09126215	0.5622338	TRUE	0.000452311	0.9848319	10.88583	0.02787754
Weighted median	6	0.94985763	-0.0514432	0.8623766	1.04621289	0.04929594	0.2966899	TRUE				
Inverse variance weighted	6	0.94544841	-0.056096	0.861326723	1.037785854	0.04754358	0.238046	TRUE			10.88694	0.05366822
Weighted mode	6	0.97913165	-0.0210892	0.869308176	1.102829603	0.06069817	0.7424057	TRUE				
MR-PRESSO (Outlier-corrected)	NA	NA	NA	NA	NA	NA	NA	NA				
**Selenium**	**N of SNPs**	**OR**	**Beta**	**95% LL**	**95% UL**	**Standard Error**	**P-val**	**Steiger test**	**MR Egger intercept**	**MR Egger intercept p-value**	**Cochran-Q**	**Cochran-Q p-val**
MR Egger	NA	NA	NA	NA	NA	NA	NA	NA	NA	NA	NA	NA
Weighted median	NA	NA	NA	NA	NA	NA	NA	NA				
Inverse variance weighted	NA	NA	NA	NA	NA	NA	NA	NA				
Weighted mode	NA	NA	NA	NA	NA	NA	NA	NA				
MR-PRESSO (Outlier-corrected)	NA	NA	NA	NA	NA	NA	NA	NA				
Wald Ratio	1	1.02959549	0.029166	0.908383188	1.166982055	0.0639056	0.648108	TRUE				
**Carotene**	**N of SNPs**	**OR**	**Beta**	**95% LL**	**95% UL**	**Standard Error**	**P-val**	**Steiger test**	**MR Egger intercept**	**MR Egger intercept p-value**	**Cochran-Q**	**Cochran-Q p-val**
MR Egger	NA	NA	NA	NA	NA	NA	NA	NA	NA	NA	NA	NA
Weighted median	NA	NA	NA	NA	NA	NA	NA	NA				
Inverse variance weighted	2	0.96890695	-0.0315867	0.86773914	1.081869691	0.056264	0.574528	TRUE			0.9324675	0.3342226
Weighted mode	NA	NA	NA	NA	NA	NA	NA	NA				
MR-PRESSO (Outlier-corrected)	NA	NA	NA	NA	NA	NA	NA	NA				
**Folate**	**N of SNPs**	**OR**	**Beta**	**95% LL**	**95% UL**	**Standard Error**	**P-val**	**Steiger test**	**MR Egger intercept**	**MR Egger intercept p-value**	**Cochran-Q**	**Cochran-Q p-val**
MR Egger	NA	NA	NA	NA	NA	NA	NA	NA	NA	NA	NA	NA
Weighted median	NA	NA	NA	NA	NA	NA	NA	NA				
Inverse variance weighted	2	0.71632307	-0.333624	0.438766862	1.169456454	0.2500832	0.1821865	TRUE			3.158398	0.07553728
Weighted mode	NA	NA	NA	NA	NA	NA	NA	NA				
MR-PRESSO (Outlier-corrected)	NA	NA	NA	NA	NA	NA	NA	NA				

Outcome: Verma, A (T1D)
Multi-ancestry

Method	N of SNPs	OR	Beta	95% LL	95% UL	Standard Error	P-val	Steiger test	MR Egger intercept	MR Egger intercept p-value	Cochran-Q	Cochran-Q p-val
**Vitamin C**												
MR Egger	32	0.98811324	-0.011958	0.827113485	1.180452014	0.09074255	0.8960389	TRUE	0.00220488	0.6478293	46.4456	0.02816331
Weighted median	32	1.02508283	0.02477342	0.899358248	1.168382914	0.0667588	0.7105717	TRUE				
Inverse variance weighted	32	1.02195262	0.02171513	0.92068539	1.134358346	0.05324083	0.6833719	TRUE			46.77522	0.03435474
Weighted mode	32	1.02784826	0.02746755	0.90894579	1.162304793	0.06272315	0.6644825	TRUE				
MR-PRESSO (Outlier-corrected)	NA	NA	NA	NA	NA	NA	NA	NA				
**Vitamin D**	**N of SNPs**	**OR**	**Beta**	**95% LL**	**95% UL**	**Standard Error**	**P-val**	**Steiger test**	**MR Egger intercept**	**MR Egger intercept p-value**	**Cochran-Q**	**Cochran-Q p-val**
MR Egger	18	0.84474839	-0.1687165	0.615100804	1.160134789	0.1618636	0.3127499	TRUE	0.007334062	0.1210628	17.70158	0.3416591
Weighted median	18	0.99779041	-0.002212	0.7841836	1.269582418	0.1229082	0.9856409	TRUE				
Inverse variance weighted	18	1.02679557	0.02644286	0.819901218	1.285897775	0.1148032	0.8178337	TRUE			20.66775	0.2415053
Weighted mode	18	0.94137035	-0.0604187	0.737299661	1.201923961	0.1246644	0.6341098	TRUE				
MR-PRESSO (Outlier-corrected)	18	NA	NA	NA	NA	NA	NA	NA				
**Retinol (vitamin A)**	**N of SNPs**	**OR**	**Beta**	**95% LL**	**95% UL**	**Standard Error**	**P-val**	**Steiger test**	**MR Egger intercept**	**MR Egger intercept p-value**	**Cochran-Q**	**Cochran-Q p-val**
MR Egger	NA	NA	NA	NA	NA	NA	NA	NA	NA	NA	NA	NA
Weighted median	NA	NA	NA	NA	NA	NA	NA	NA				
Inverse variance weighted	NA	NA	NA	NA	NA	NA	NA	NA				
Weighted mode	NA	NA	NA	NA	NA	NA	NA	NA				
MR-PRESSO (Outlier-corrected)	NA	NA	NA	NA	NA	NA	NA	NA				

Wald ratio	1	0.83961208	-0.1748153	0.623973547	1.129772973	0.1514449	0.2483706	TRUE				
**Vitamin K (1st model)**	**N of SNPs**	**OR**	**Beta**	**95% LL**	**95% UL**	**Standard Error**	**P-val**	**Steiger test**	**MR Egger intercept**	**MR Egger intercept p-value**	**Cochran-Q**	**Cochran-Q p-val**
MR Egger	5	0.97489709	-0.0254234	0.808317195	1.175806169	0.0956007	0.8075199	TRUE	0.01211372	0.6117497	1.512071	0.6794871
Weighted median	5	1.01310421	0.0130191	0.94533768	1.085728591	0.03532254	0.7124426	TRUE				
Inverse variance weighted	5	1.02635124	0.02601003	0.969554907	1.086474693	0.029045	0.3705157	TRUE			1.830952	0.766815
Weighted mode	5	1.0110382	0.01097772	0.925352015	1.104658789	0.04518305	0.819986	TRUE				
MR-PRESSO (Outlier-corrected)	NA	NA	NA	NA	NA	NA	NA	NA				
**Vitamin K (2nd model)**	**N of SNPs**	**OR**	**Beta**	**95% LL**	**95% UL**	**Standard Error**	**P-val**	**Steiger test**	**MR Egger intercept**	**MR Egger intercept p-value**	**Cochran-Q**	**Cochran-Q p-val**
MR Egger	5	0.98493814	-0.0151764	0.92465695	1.049149243	0.032222466	0.669779512	TRUE	0.009983536	0.400223	1.451767	0.6934465
Weighted median	5	1.00135283	0.00135192	0.955396333	1.049519931	0.023969864	0.955022594	TRUE				
Inverse variance weighted	5	1.00946603	0.00942151	0.970393774	1.050111508	0.020140228	0.639930788	TRUE			2.408142	0.6611563
Weighted mode	5	0.99905247	-0.000948	0.95518368	1.044936027	0.022910022	0.968977329	TRUE				
MR-PRESSO (Outlier-corrected)	NA	NA	NA	NA	NA	NA	NA	NA				
**Vitamin K (3rd model)**	**N of SNPs**	**OR**	**Beta**	**95% LL**	**95% UL**	**Standard Error**	**P-val**	**Steiger test**	**MR Egger intercept**	**MR Egger intercept p-value**	**Cochran-Q**	**Cochran-Q p-val**
MR Egger	4	0.98755101	-0.0125271	0.922136881	1.057605456	0.03496657	0.754429	TRUE	0.008340269	0.5552507	1.39768	0.4971616
Weighted median	4	1.00094573	0.00094528	0.951834957	1.052590419	0.02566781	0.9706225	TRUE				
Inverse variance weighted	4	1.00703643	0.00701179	0.966090792	1.049717461	0.02117819	0.74058	TRUE			1.89083	0.5953715
Weighted mode	4	0.99943911	-0.000561	0.954000787	1.047041621	0.02373966	0.9826291	TRUE				
MR-PRESSO (Outlier-corrected)	NA	NA	NA	NA	NA	NA	NA	NA				
**Vitamin B12**	**N of SNPs**	**OR**	**Beta**	**95% LL**	**95% UL**	**Standard Error**	**P-val**	**Steiger test**	**MR Egger intercept**	**MR Egger intercept p-value**	**Cochran-Q**	**Cochran-Q p-val**
MR Egger	9	1.01792528	0.01776652	0.877123844	1.181329056	0.07595592	0.8217518	TRUE	0.0036881	0.7632289	10.9347	0.1414927
Weighted median	9	1.01716732	0.01702163	0.942419062	1.097844266	0.03894228	0.6620397	TRUE				
Inverse variance weighted	9	1.03914296	0.0383963	0.969028918	1.114330108	0.03564139	0.2813483	TRUE			11.08797	0.1967644
Weighted mode	9	1.04284812	0.04195555	0.96118164	1.131453373	0.04160583	0.3427832	TRUE				
MR-PRESSO (Outlier-corrected)	NA	NA	NA	NA	NA	NA	NA	NA				
**Vitamin B6**	**N of SNPs**	**OR**	**Beta**	**95% LL**	**95% UL**	**Standard Error**	**P-val**	**Steiger test**	**MR Egger intercept**	**MR Egger intercept p-value**	**Cochran-Q**	**Cochran-Q p-val**
MR Egger	NA	NA	NA	NA	NA	NA	NA	NA	NA	NA	NA	NA
Weighted median	NA	NA	NA	NA	NA	NA	NA	NA				
Inverse variance weighted	NA	NA	NA	NA	NA	NA	NA	NA				
Weighted mode	NA	NA	NA	NA	NA	NA	NA	NA				
MR-PRESSO (Outlier-corrected)	NA	NA	NA	NA	NA	NA	NA	NA				
Wald Ratio	1	1.00275691	0.00275312	0.987628557	1.018117	0.007755982	0.7226143	TRUE				
**Alpha-tocopherol (vitamin E)**	**N of SNPs**	**OR**	**Beta**	**95% LL**	**95% UL**	**Standard Error**	**P-val**	**Steiger test**	**MR Egger intercept**	**MR Egger intercept p-value**	**Cochran-Q**	**Cochran-Q p-val**
MR Egger	NA	NA	NA	NA	NA	NA	NA	NA	NA	NA	NA	NA
Weighted median	NA	NA	NA	NA	NA	NA	NA	NA				
Inverse variance weighted	NA	NA	NA	NA	NA	NA	NA	NA				
Weighted mode	NA	NA	NA	NA	NA	NA	NA	NA				
MR-PRESSO (Outlier-corrected)	NA	NA	NA	NA	NA	NA	NA	NA				
Wald Ratio	1	1.31455539	0.2734985	0.667818447	2.587613256	0.3455293	0.428632	TRUE				
**Gamma and beta-tocopherol (vitamin E)**	**N of SNPs**	**OR**	**Beta**	**95% LL**	**95% UL**	**Standard Error**	**P-val**	**Steiger test**	**MR Egger intercept**	**MR Egger intercept p-value**	**Cochran-Q**	**Cochran-Q p-val**
MR Egger	NA	NA	NA	NA	NA	NA	NA	NA	NA	NA	NA	NA
Weighted median	NA	NA	NA	NA	NA	NA	NA	NA				
Inverse variance weighted	NA	NA	NA	NA	NA	NA	NA	NA				
Weighted mode	NA	NA	NA	NA	NA	NA	NA	NA				
MR-PRESSO (Outlier-corrected)	NA	NA	NA	NA	NA	NA	NA	NA				
Wald Ratio	1	0.88682669	-0.1201057	0.696127025	1.129767352	0.1235293	0.3309086	TRUE				
**Magnesium**	**N of SNPs**	**OR**	**Beta**	**95% LL**	**95% UL**	**Standard Error**	**P-val**	**Steiger test**	**MR Egger intercept**	**MR Egger intercept p-value**	**Cochran-Q**	**Cochran-Q p-val**
MR Egger	82	0.96692599	-0.0336333	0.580337183	1.611039067	0.26046565	0.89758074	TRUE	-0.006141382	0.5755227	715.2468	1.02E-102
Weighted median	82	0.9610594	-0.0397191	0.862879918	1.070409861	0.05497994	0.47003131	TRUE				
Inverse variance weighted	82	0.84449486	-0.1690166	0.695701983	1.02511073	0.09888636	0.08741377	TRUE			718.073	8.74E-103
Weighted mode	82	0.9205326	-0.0828029	0.735485317	1.15213758	0.11450094	0.47166277	TRUE				
MR-PRESSO (Outlier-corrected)	82	0.93367207	-0.06863	0.86726713	1.005161517	0.03764196	0.07162555	NA				
MR-LAP	83	0.97392656	-0.0264194	0.934441632	1.015079925	0.02111568	0.2108712	NA				
**Potassium**	**N of SNPs**	**OR**	**Beta**	**95% LL**	**95% UL**	**Standard Error**	**P-val**	**Steiger test**	**MR Egger intercept**	**MR Egger intercept p-value**	**Cochran-Q**	**Cochran-Q p-val**
MR Egger	294	1.80479335	0.5904461	1.49966722	2.172001233	0.09449126	1.46E-09	TRUE	-0.006256358	0.02606004	522.1033	3.12E-15
Weighted median	294	1.31105729	0.2708339	1.181347117	1.45500944	0.05315229	3.48E-07	TRUE				
Inverse variance weighted	294	1.4955845	0.4025171	1.373278714	1.628782979	0.04352857	2.30E-21	TRUE			531.0486	5.55E-16
Weighted mode	294	1.1241137	0.1169949	0.884627072	1.428434244	0.12223674	3.39E-01	TRUE				
MR-PRESSO (Outlier-corrected)	294	1.45693469	0.3763347	1.353668208	1.568078997	0.03750847	1.54E-20	NA				
MR-LAP	296	1.09878002	0.09420049	1.075554068	1.122507519	0.01090028	5.52E-18	NA				
**Iron**	**N of SNPs**	**OR**	**Beta**	**95% LL**	**95% UL**	**Standard Error**	**P-val**	**Steiger test**	**MR Egger intercept**	**MR Egger intercept p-value**	**Cochran-Q**	**Cochran-Q p-val**
MR Egger	21	1.13932192	0.13043328	0.949333749	1.367332029	0.09307558	0.17722711	TRUE	-0.003834973	0.6029622	45.20602	0.000641307
Weighted median	21	1.10164049	0.09680042	0.997188575	1.217033359	0.05082439	0.05683134	TRUE				
Inverse variance weighted	21	1.09820733	0.09367915	0.974829522	1.23720026	0.06080195	0.1233835	TRUE			45.87172	0.000839335
Weighted mode	21	1.10483568	0.09969662	1.008763573	1.210057458	0.04641389	0.04414823	TRUE				
MR-PRESSO (Outlier-corrected)	21	1.07690016	0.07408669	0.977594534	1.186293407	0.0493607	0.1498109	NA				
MR-LAP	21	1.01739096	0.01724147	0.993963264	1.041370851	0.01188597	0.1468987	NA				
**Zinc**	**N of SNPs**	**OR**	**Beta**	**95% LL**	**95% UL**	**Standard Error**	**P-val**	**Steiger test**	**MR Egger intercept**	**MR Egger intercept p-value**	**Cochran-Q**	**Cochran-Q p-val**
MR Egger	6	1.00323604	0.00323081	0.816537711	1.232622245	0.10505765	0.97694	TRUE	0.001691685	0.9412912	3.943765	0.41367
Weighted median	6	1.02754143	0.02716899	0.967391381	1.091431466	0.03077608	0.3773467	TRUE				
Inverse variance weighted	6	1.01128009	0.01121695	0.961796744	1.063309301	0.02559647	0.6612251	TRUE			3.949908	0.5566504
Weighted mode	6	1.02989383	0.02945572	0.949930541	1.116588267	0.04123578	0.5069787	TRUE				
MR-PRESSO (Outlier-corrected)	NA	NA	NA	NA	NA	NA	NA	NA				
**Copper**	**N of SNPs**	**OR**	**Beta**	**95% LL**	**95% UL**	**Standard Error**	**P-val**	**Steiger test**	**MR Egger intercept**	**MR Egger intercept p-value**	**Cochran-Q**	**Cochran-Q p-val**
MR Egger	6	0.99083133	-0.009211	0.917707019	1.069782289	0.03911537	0.8253995	TRUE	0.003040526	0.8043097	7.932721	0.09407402
Weighted median	6	0.99396845	-0.0060498	0.954298508	1.035287452	0.02078007	0.7709476	TRUE				
Inverse variance weighted	6	0.99860706	-0.0013939	0.954321448	1.044947769	0.02314327	0.9519729	TRUE			8.071691	0.1523286
Weighted mode	6	0.99705029	-0.0029541	0.955549061	1.040353999	0.02169138	0.8969875	TRUE				
MR-PRESSO (Outlier-corrected)	NA	NA	NA	NA	NA	NA	NA	NA				
**Selenium**	**N of SNPs**	**OR**	**Beta**	**95% LL**	**95% UL**	**Standard Error**	**P-val**	**Steiger test**	**MR Egger intercept**	**MR Egger intercept p-value**	**Cochran-Q**	**Cochran-Q p-val**
MR Egger	NA	NA	NA	NA	NA	NA	NA	NA	NA	NA	NA	NA
Weighted median	NA	NA	NA	NA	NA	NA	NA	NA				
Inverse variance weighted	NA	NA	NA	NA	NA	NA	NA	NA				
Weighted mode	NA	NA	NA	NA	NA	NA	NA	NA				
MR-PRESSO (Outlier-corrected)	NA	NA	NA	NA	NA	NA	NA	NA				
Wald Ratio	1	0.98504867	-0.0150642	0.901186125	1.076715289	0.04539757	0.7400184	TRUE				
**Carotene**	**N of SNPs**	**OR**	**Beta**	**95% LL**	**95% UL**	**Standard Error**	**P-val**	**Steiger test**	**MR Egger intercept**	**MR Egger intercept p-value**	**Cochran-Q**	**Cochran-Q p-val**
MR Egger	NA	NA	NA	NA	NA	NA	NA	NA	NA	NA	NA	NA
Weighted median	NA	NA	NA	NA	NA	NA	NA	NA				
Inverse variance weighted	2	0.96804024	-0.0324816	0.827931286	1.131859524	0.07976709	0.6838568	TRUE			4.175571	0.04101074
Weighted mode	NA	NA	NA	NA	NA	NA	NA	NA				
MR-PRESSO (Outlier-corrected)	NA	NA	NA	NA	NA	NA	NA	NA				
**Folate**	**N of SNPs**	**OR**	**Beta**	**95% LL**	**95% UL**	**Standard Error**	**P-val**	**Steiger test**	**MR Egger intercept**	**MR Egger intercept p-value**	**Cochran-Q**	**Cochran-Q p-val**
MR Egger	NA	NA	NA	NA	NA	NA	NA	NA	NA	NA	NA	NA
Weighted median	NA	NA	NA	NA	NA	NA	NA	NA				
Inverse variance weighted	2	0.89668702	-0.1090484	0.730633071	1.100480717	0.1044875	0.2966472	TRUE			0.3460588	0.5563527
Weighted mode	NA	NA	NA	NA	NA	NA	NA	NA				
MR-PRESSO (Outlier-corrected)	NA	NA	NA	NA	NA	NA	NA	NA				

Outcome: Verma, A (T1D)
Hispanic or Latin American

Method	N of SNPs	OR	Beta	95% LL	95% UL	Standard Error	P-val	Steiger test	MR Egger intercept	MR Egger intercept p-value	Cochran-Q	Cochran-Q p-val
**Vitamin C**												
MR Egger	34	0.95112402	-0.0501108	0.8655726	1.045131156	0.04808837	0.3051944	TRUE	0.002201965	0.3558124	28.10012	0.6643992
Weighted median	34	0.94949849	-0.0518213	0.88018357	1.024271994	0.03867523	0.1802745	TRUE				
Inverse variance weighted	34	0.98800334	-0.0120692	0.939345068	1.039182122	0.02576693	0.6394994	TRUE			28.97796	0.6677536
Weighted mode	34	0.95390935	-0.0471866	0.875432648	1.039420972	0.04380124	0.2891633	TRUE				
MR-PRESSO (Outlier-corrected)	NA	NA	NA	NA	NA	NA	NA	NA				
**Vitamin D**	**N of SNPs**	**OR**	**Beta**	**95% LL**	**95% UL**	**Standard Error**	**P-val**	**Steiger test**	**MR Egger intercept**	**MR Egger intercept p-value**	**Cochran-Q**	**Cochran-Q p-val**
MR Egger	17	0.96758246	-0.0329546	0.816997206	1.145922906	0.08630866	0.7079474	TRUE	-0.001910549	0.4356048	13.39735	0.5716363
Weighted median	17	0.95071397	-0.050542	0.822053513	1.099511208	0.07418763	0.4956988	TRUE				
Inverse variance weighted	17	0.91919631	-0.0842556	0.820646772	1.029580423	0.05786068	0.1453428	TRUE			14.03904	0.5958055
Weighted mode	17	0.94991143	-0.0513865	0.812959812	1.109933987	0.07943218	0.5268543	TRUE				
MR-PRESSO (Outlier-corrected)	17	NA	NA	NA	NA	NA	NA	NA				
**Retinol (vitamin A)**	**N of SNPs**	**OR**	**Beta**	**95% LL**	**95% UL**	**Standard Error**	**P-val**	**Steiger test**	**MR Egger intercept**	**MR Egger intercept p-value**	**Cochran-Q**	**Cochran-Q p-val**
MR Egger	NA	NA	NA	NA	NA	NA	NA	NA	NA	NA	NA	NA
Weighted median	NA	NA	NA	NA	NA	NA	NA	NA				
Inverse variance weighted	NA	NA	NA	NA	NA	NA	NA	NA				
Weighted mode	NA	NA	NA	NA	NA	NA	NA	NA				
MR-PRESSO (Outlier-corrected)	NA	NA	NA	NA	NA	NA	NA	NA				
Wald ratio	1	1.17869171	0.1644051	1.008698824	1.377332962	0.07946117	0.03854608	TRUE				
**Vitamin K (1st model)**	**N of SNPs**	**OR**	**Beta**	**95% LL**	**95% UL**	**Standard Error**	**P-val**	**Steiger test**	**MR Egger intercept**	**MR Egger intercept p-value**	**Cochran-Q**	**Cochran-Q p-val**
MR Egger	5	1.12090061	0.11413248	0.93050106	1.350259807	0.09498191	0.3157178	TRUE	-0.01785136	0.4461635	6.385212	0.09430105
Weighted median	5	1.0189747	0.01879693	0.97554752	1.06433508	0.02222109	0.3976058	TRUE				
Inverse variance weighted	5	1.03435574	0.03377876	0.988027753	1.082856018	0.02337921	0.1485084	TRUE			8.013308	0.09109192
Weighted mode	5	1.01796652	0.01780703	0.966048425	1.072674837	0.02670834	0.541438	TRUE				
MR-PRESSO (Outlier-corrected)	NA	NA	NA	NA	NA	NA	NA	NA				
**Vitamin K (2nd model)**	**N of SNPs**	**OR**	**Beta**	**95% LL**	**95% UL**	**Standard Error**	**P-val**	**Steiger test**	**MR Egger intercept**	**MR Egger intercept p-value**	**Cochran-Q**	**Cochran-Q p-val**
MR Egger	5	1.00304123	0.00303662	0.948856139	1.060320598	0.02833403	0.9214177	TRUE	0.004737642	0.6507847	9.521526	0.02310346
Weighted median	5	1.00658063	0.00655907	0.983156298	1.030563065	0.01201339	0.5850794	TRUE				
Inverse variance weighted	5	1.01409995	0.01400147	0.982355942	1.046869735	0.01622604	0.3881918	TRUE			10.318352	0.0353933
Weighted mode	5	1.0038975	0.00388993	0.982065442	1.026214911	0.01121799	0.7462469	TRUE				
MR-PRESSO (Outlier-corrected)	NA	NA	NA	NA	NA	NA	NA	NA				
**Vitamin K (3rd model)**	**N of SNPs**	**OR**	**Beta**	**95% LL**	**95% UL**	**Standard Error**	**P-val**	**Steiger test**	**MR Egger intercept**	**MR Egger intercept p-value**	**Cochran-Q**	**Cochran-Q p-val**
MR Egger	4	1.00271997	0.00271628	0.930943836	1.080030082	0.03789419	0.9493791	TRUE	0.005399758	0.7334979	9.413936	0.009032123
Weighted median	4	1.00667133	0.00664918	0.981013478	1.033000251	0.01317258	0.6137176	TRUE				
Inverse variance weighted	4	1.0145694	0.01446428	0.976401474	1.054229321	0.01956414	0.4597088	TRUE			10.133662	0.017463467
Weighted mode	4	1.00356378	0.00355745	0.978991229	1.028753099	0.01264798	0.79679078	TRUE				
MR-PRESSO (Outlier-corrected)	NA	NA	NA	NA	NA	NA	NA	NA				
**Vitamin B12**	**N of SNPs**	**OR**	**Beta**	**95% LL**	**95% UL**	**Standard Error**	**P-val**	**Steiger test**	**MR Egger intercept**	**MR Egger intercept p-value**	**Cochran-Q**	**Cochran-Q p-val**
MR Egger	9	0.98169378	-0.0184759	0.905928667	1.063795326	0.04097901	0.665724	TRUE	-0.000661308	0.9162989	4.578108	0.7112924

Weighted median	9	0.96739326	-0.0331502	0.922538787	1.01442858	0.02422228	0.1711299	TRUE				
Inverse variance weighted	9	0.97785356	-0.0223954	0.94095742	1.016196454	0.01962349	0.253765	TRUE			4.589979	0.800365
Weighted mode	9	0.97212436	-0.0282715	0.922989851	1.0238745	0.02646199	0.3165226	TRUE				
MR-PRESSO (Outlier-corrected)	NA	NA	NA	NA	NA	NA	NA	NA				
**Vitamin B6**	**N of SNPs**	**OR**	**Beta**	**95% LL**	**95% UL**	**Standard Error**	**P-val**	**Steiger test**	**MR Egger intercept**	**MR Egger intercept p-value**	**Cochran-Q**	**Cochran-Q p-val**
MR Egger	NA	NA	NA	NA	NA	NA	NA	NA	NA	NA	NA	NA
Weighted median	NA	NA	NA	NA	NA	NA	NA	NA				
Inverse variance weighted	NA	NA	NA	NA	NA	NA	NA	NA				
Weighted mode	NA	NA	NA	NA	NA	NA	NA	NA				
MR-PRESSO (Outlier-corrected)	NA	NA	NA	NA	NA	NA	NA	NA				
Wald Ratio	1	0.99580374	-0.0042051	0.987882529	1.003788475	0.004074695	0.302072117	TRUE				
**Alpha-tocopherol (vitamin E)**	**N of SNPs**	**OR**	**Beta**	**95% LL**	**95% UL**	**Standard Error**	**P-val**	**Steiger test**	**MR Egger intercept**	**MR Egger intercept p-value**	**Cochran-Q**	**Cochran-Q p-val**
MR Egger	NA	NA	NA	NA	NA	NA	NA	NA	NA	NA	NA	NA
Weighted median	NA	NA	NA	NA	NA	NA	NA	NA				
Inverse variance weighted	NA	NA	NA	NA	NA	NA	NA	NA				
Weighted mode	NA	NA	NA	NA	NA	NA	NA	NA				
MR-PRESSO (Outlier-corrected)	NA	NA	NA	NA	NA	NA	NA	NA				
Wald Ratio	1	1.09060699	0.08673441	0.780943319	1.523060087	0.17040159	0.61075222	TRUE				
**Gamma and beta-tocopherol (vitamin E)**	**N of SNPs**	**OR**	**Beta**	**95% LL**	**95% UL**	**Standard Error**	**P-val**	**Steiger test**	**MR Egger intercept**	**MR Egger intercept p-value**	**Cochran-Q**	**Cochran-Q p-val**
MR Egger	NA	NA	NA	NA	NA	NA	NA	NA	NA	NA	NA	NA
Weighted median	NA	NA	NA	NA	NA	NA	NA	NA				
Inverse variance weighted	NA	NA	NA	NA	NA	NA	NA	NA				
Weighted mode	NA	NA	NA	NA	NA	NA	NA	NA				
MR-PRESSO (Outlier-corrected)	NA	NA	NA	NA	NA	NA	NA	NA				
Wald Ratio	1	0.87117775	-0.1379093	0.743870537	1.02027252	0.08060154	0.08708111	TRUE				
**Magnesium**	**N of SNPs**	**OR**	**Beta**	**95% LL**	**95% UL**	**Standard Error**	**P-val**	**Steiger test**	**MR Egger intercept**	**MR Egger intercept p-value**	**Cochran-Q**	**Cochran-Q p-val**
MR Egger	83	0.93757349	-0.0644601	0.830758443	1.058122307	0.06171228	0.29934892	TRUE	0.001459667	0.5762983	141.4039	3.78E-05
Weighted median	83	0.94508534	-0.0564801	0.891179641	1.002251686	0.02996388	0.05943827	TRUE				
Inverse variance weighted	83	0.96797262	-0.0325515	0.923747085	1.014315502	0.02385994	0.17248145	TRUE			141.9534	4.49E-05
Weighted mode	83	0.92527715	-0.077662	0.854936815	1.001404779	0.04033967	0.05767124	TRUE				
MR-PRESSO (Outlier-corrected)	83	0.9871842	-0.0128986	0.953617539	1.021932386	0.01764998	0.4667576	NA				
MR-LAP	90	0.9940618	-0.0059559	0.939162946	1.052169776	0.02898489	0.8371948	NA				
**Potassium**	**N of SNPs**	**OR**	**Beta**	**95% LL**	**95% UL**	**Standard Error**	**P-val**	**Steiger test**	**MR Egger intercept**	**MR Egger intercept p-value**	**Cochran-Q**	**Cochran-Q p-val**
MR Egger	291	1.37661068	0.31962445	1.243257301	1.524267717	0.05198452	2.59E-09	TRUE	-0.004796808	0.000742808	313.2647	0.15627576
Weighted median	291	1.13572719	0.12727314	1.066429692	1.209527693	0.03212082	7.42E-05	TRUE				
Inverse variance weighted	291	1.17137849	0.15818125	1.12226937	1.222636559	0.02185122	4.52E-13	TRUE			325.8672	0.07222785
Weighted mode	291	1.09972247	0.09505785	0.985049179	1.227745316	0.05618447	9.17E-02	TRUE				
MR-PRESSO (Outlier-corrected)	291	1.14558406	0.1359146	1.097206899	1.196094221	0.02201369	2.17E-09	NA				
MR-LAP	293	1.26538902	0.2353796	1.208690465	1.324747251	0.02338882	7.99E-24	NA				
**Iron**	**N of SNPs**	**OR**	**Beta**	**95% LL**	**95% UL**	**Standard Error**	**P-val**	**Steiger test**	**MR Egger intercept**	**MR Egger intercept p-value**	**Cochran-Q**	**Cochran-Q p-val**
MR Egger	21	1.02925811	0.02883826	0.941755586	1.124890863	0.04533049	0.53225	TRUE	0.001432956	0.6662934	24.7152	0.1701318
Weighted median	21	1.04674661	0.04568689	0.981571073	1.116249757	0.03279987	0.1636505	TRUE				
Inverse variance weighted	21	1.04527734	0.04428225	0.98963098	1.104052668	0.02791092	0.1126131	TRUE			24.9648	0.202781
Weighted mode	21	1.05347683	0.05209596	0.991238893	1.119622563	0.03106922	0.1091491	TRUE				
MR-PRESSO (Outlier-corrected)	NA	NA	NA	NA	NA	NA	NA	NA				
MR-LAP	21	1.04407718	0.04313341	0.987999133	1.103338164	0.02816677	0.125681	NA				
**Zinc**	**N of SNPs**	**OR**	**Beta**	**95% LL**	**95% UL**	**Standard Error**	**P-val**	**Steiger test**	**MR Egger intercept**	**MR Egger intercept p-value**	**Cochran-Q**	**Cochran-Q p-val**
MR Egger	6	1.07383253	0.07123406	0.957471445	1.204334936	0.05851706	0.290386	TRUE	-0.01803751	0.2080053	3.193204	0.5260294
Weighted median	6	1.00489682	0.00488487	0.966963212	1.044318555	0.0196325	0.8035034	TRUE				
Inverse variance weighted	6	0.98628682	-0.0138081	0.957496587	1.015942723	0.01511482	0.3609557	TRUE			5.443139	0.3642212
Weighted mode	6	1.01437348	0.01427116	0.96549143	1.065730394	0.02519858	0.5956317	TRUE				
MR-PRESSO (Outlier-corrected)	NA	NA	NA	NA	NA	NA	NA	NA				
**Copper**	**N of SNPs**	**OR**	**Beta**	**95% LL**	**95% UL**	**Standard Error**	**P-val**	**Steiger test**	**MR Egger intercept**	**MR Egger intercept p-value**	**Cochran-Q**	**Cochran-Q p-val**
MR Egger	6	1.00466434	0.00465349	0.977446621	1.032639948	0.0140128	0.756495	TRUE	-4.09E-05	0.9931189	2.824752	0.5875677
Weighted median	6	0.99997081	-2.919E-05	0.978324037	1.022096542	0.01116589	0.9979139	TRUE				
Inverse variance weighted	6	1.00456868	0.00455827	0.986200036	1.023279446	0.00941548	0.628297	TRUE			2.824836	0.726969
Weighted mode	6	0.99440242	-0.0056133	0.97032798	1.01907417	0.012504	0.8787547	TRUE				
MR-PRESSO (Outlier-corrected)	NA	NA	NA	NA	NA	NA	NA	NA				
**Selenium**	**N of SNPs**	**OR**	**Beta**	**95% LL**	**95% UL**	**Standard Error**	**P-val**	**Steiger test**	**MR Egger intercept**	**MR Egger intercept p-value**	**Cochran-Q**	**Cochran-Q p-val**
MR Egger	NA	NA	NA	NA	NA	NA	NA	NA	NA	NA	NA	NA
Weighted median	NA	NA	NA	NA	NA	NA	NA	NA				
Inverse variance weighted	NA	NA	NA	NA	NA	NA	NA	NA				
Weighted mode	NA	NA	NA	NA	NA	NA	NA	NA				
MR-PRESSO (Outlier-corrected)	NA	NA	NA	NA	NA	NA	NA	NA				
Wald Ratio	1	0.99756891	-0.0024341	0.947685628	1.050077896	0.026172652	0.92590356	TRUE				
**Carotene**	**N of SNPs**	**OR**	**Beta**	**95% LL**	**95% UL**	**Standard Error**	**P-val**	**Steiger test**	**MR Egger intercept**	**MR Egger intercept p-value**	**Cochran-Q**	**Cochran-Q p-val**
MR Egger	NA	NA	NA	NA	NA	NA	NA	NA	NA	NA	NA	NA
Weighted median	NA	NA	NA	NA	NA	NA	NA	NA				
Inverse variance weighted	2	1.01849546	0.0183265	0.974220784	1.064782255	0.02267542	0.41897036	TRUE			1.109098	0.2922782
Weighted mode	NA	NA	NA	NA	NA	NA	NA	NA				
MR-PRESSO (Outlier-corrected)	NA	NA	NA	NA	NA	NA	NA	NA				
**Folate**	**N of SNPs**	**OR**	**Beta**	**95% LL**	**95% UL**	**Standard Error**	**P-val**	**Steiger test**	**MR Egger intercept**	**MR Egger intercept p-value**	**Cochran-Q**	**Cochran-Q p-val**
MR Egger	NA	NA	NA	NA	NA	NA	NA	NA	NA	NA	NA	NA
Weighted median	NA	NA	NA	NA	NA	NA	NA	NA				
Inverse variance weighted	2	1.02220085	0.021958	0.916782237	1.139741301	0.0555323	0.69253	TRUE			0.3609199	0.5479958
Weighted mode	NA	NA	NA	NA	NA	NA	NA	NA				
MR-PRESSO (Outlier-corrected)	NA	NA	NA	NA	NA	NA	NA	NA				

Outcome: Verma, A (T1D)

African American or Afro-Caribbean

Method	N of SNPs	OR	Beta	95% LL	95% UL	Standard Error	P-val	Steiger test	MR Egger intercept	MR Egger intercept p-value	Cochran-Q	Cochran-Q p-val
**Vitamin C**												
MR Egger	33	1.024611	0.02431303	0.974125815	1.077712639	0.02577951	0.3529149	TRUE	-0.001390008	0.3744265	34.42619	0.307095
Weighted median	33	1.01291223	0.01282958	0.969727511	1.058020088	0.02222946	0.5638428	TRUE				
Inverse variance weighted	33	1.00703796	0.00701331	0.973737168	1.041477608	0.01715672	0.6827017	TRUE			35.32813	0.3137779
Weighted mode	33	1.01450541	0.01440121	0.971444502	1.059475063	0.02212875	0.5198268	TRUE				
MR-PRESSO (Outlier-corrected)	NA	NA	NA	NA	NA	NA	NA	NA				
**Vitamin D**	**N of SNPs**	**OR**	**Beta**	**95% LL**	**95% UL**	**Standard Error**	**P-val**	**Steiger test**	**MR Egger intercept**	**MR Egger intercept p-value**	**Cochran-Q**	**Cochran-Q p-val**
MR Egger	17	0.96673583	-0.03383	0.803112154	1.163695696	0.0946076	0.7256374	TRUE	0.001214702	0.6048399	22.10503	0.1050794
Weighted median	17	0.9918003	-0.0082335	0.858374191	1.145966235	0.07371513	0.9110664	TRUE				
Inverse variance weighted	17	1.00439256	0.00438294	0.893597352	1.128925024	0.05963415	0.9414105	TRUE			22.51675	0.1272763
Weighted mode	17	0.98649355	-0.0135985	0.857039058	1.135501957	0.07177209	0.852109	TRUE				
MR-PRESSO (Outlier-corrected)	NA	NA	NA	NA	NA	NA	NA	NA				
**Retinol (vitamin A)**	**N of SNPs**	**OR**	**Beta**	**95% LL**	**95% UL**	**Standard Error**	**P-val**	**Steiger test**	**MR Egger intercept**	**MR Egger intercept p-value**	**Cochran-Q**	**Cochran-Q p-val**
MR Egger	NA	NA	NA	NA	NA	NA	NA	NA	NA	NA	NA	NA
Weighted median	NA	NA	NA	NA	NA	NA	NA	NA				
Inverse variance weighted	NA	NA	NA	NA	NA	NA	NA	NA				
Weighted mode	NA	NA	NA	NA	NA	NA	NA	NA				
MR-PRESSO (Outlier-corrected)	NA	NA	NA	NA	NA	NA	NA	NA				
Wald ratio	1	1.14852071	0.13847478	0.940321907	1.402817293	0.102044789	0.174781211	TRUE				
**Vitamin K (1st model)**	**N of SNPs**	**OR**	**Beta**	**95% LL**	**95% UL**	**Standard Error**	**P-val**	**Steiger test**	**MR Egger intercept**	**MR Egger intercept p-value**	**Cochran-Q**	**Cochran-Q p-val**
MR Egger	5	1.02468227	0.02438259	0.947805237	1.10779485	0.03979022	0.5833471	TRUE	-0.007307535	0.4888582	1.515673	0.6786577
Weighted median	5	0.99445142	0.00289056	0.974980189	1.031608507	0.01440239	0.8409333	TRUE				
Inverse variance weighted	5	0.99445142	-0.005564	0.972111238	1.017304999	0.01159235	0.6312455	TRUE			2.134634	0.7110123
Weighted mode	5	1.00423502	0.00422608	0.969122181	1.040620047	0.0181585	0.8273926	TRUE				
MR-PRESSO (Outlier-corrected)	NA	NA	NA	NA	NA	NA	NA	NA				
**Vitamin K (2nd model)**	**N of SNPs**	**OR**	**Beta**	**95% LL**	**95% UL**	**Standard Error**	**P-val**	**Steiger test**	MR Egger intercept	**MR Egger intercept p-value**	**Cochran-Q**	**Cochran-Q p-val**
MR Egger	6	0.99727045	-0.0027333	0.981881431	1.012900667	0.007934409	0.7478314	TRUE	-0.000267742	0.9415632	2.718586	0.6059662
Weighted median	6	0.99741195	-0.0025914	0.986657147	1.008283991	0.005531259	0.6394262	TRUE				
Inverse variance weighted	6	0.99679311	-0.003212	0.987015607	1.006667468	0.005029277	0.5230381	TRUE			2.724673	0.7423424
Weighted mode	6	0.99729497	-0.0027087	0.987287001	1.00740438	0.005145817	0.6211242	TRUE				
MR-PRESSO (Outlier-corrected)	NA	NA	NA	NA	NA	NA	NA	NA				
**Vitamin K (3rd model)**	**N of SNPs**	**OR**	**Beta**	**95% LL**	**95% UL**	**Standard Error**	**P-val**	**Steiger test**	MR Egger intercept	**MR Egger intercept p-value**	**Cochran-Q**	**Cochran-Q p-val**
MR Egger	4	1.00240545	0.00240256	0.984037664	1.021116077	0.009435543	0.8227998	TRUE	-0.003954565	0.4915782	1.317372	0.5175309
Weighted median	4	0.99710928	-0.0028949	0.985361897	1.008996718	0.006046629	0.632107	TRUE				
Inverse variance weighted	4	0.99590111	-0.0041073	0.985580764	1.006329517	0.005314738	0.4396311	TRUE			2.014581	0.5693859
Weighted mode	4	0.99711908	-0.0028851	0.98668995	1.007658442	0.005364461	0.6280423	TRUE				
MR-PRESSO (Outlier-corrected)	NA	NA	NA	NA	NA	NA	NA	NA				
**Vitamin B12**	**N of SNPs**	**OR**	**Beta**	**95% LL**	**95% UL**	**Standard Error**	**P-val**	**Steiger test**	**MR Egger intercept**	**MR Egger intercept p-value**	**Cochran-Q**	**Cochran-Q p-val**
MR Egger	9	1.0359943	0.03536164	0.990231721	1.083871747	0.02304997	0.168868636	TRUE	-0.001610522	0.7143001	7.469166	0.3817155
Weighted median	9	1.03468172	0.03409386	1.00835261	1.061698305	0.01315099	0.009528326	TRUE				
Inverse variance weighted	9	1.02811872	0.02773065	1.006052516	1.050668919	0.01106958	0.012240938	TRUE			7.624289	0.4710034
Weighted mode	9	1.03620884	0.03556871	1.010509126	1.062562168	0.01281348	0.024077102	TRUE				
MR-PRESSO (Outlier-corrected)	NA	NA	NA	NA	NA	NA	NA	NA				
**Vitamin B6**	**N of SNPs**	**OR**	**Beta**	**95% LL**	**95% UL**	**Standard Error**	**P-val**	**Steiger test**	**MR Egger intercept**	**MR Egger intercept p-value**	**Cochran-Q**	**Cochran-Q p-val**
MR Egger	1	NA	NA	NA	NA	NA	NA	NA	NA	NA	NA	NA
Weighted median	1	NA	NA	NA	NA	NA	NA	NA				
Inverse variance weighted	1	NA	NA	NA	NA	NA	NA	NA				
Weighted mode	1	NA	NA	NA	NA	NA	NA	NA				
MR-PRESSO (Outlier-corrected)	1	NA	NA	NA	NA	NA	NA	NA				
Wald Ratio	1	0.99199946	-0.0080327	0.983574894	1.000496185	0.004351417	0.064892205	TRUE				
**Alpha-tocopherol (vitamin E)**	**N of SNPs**	**OR**	**Beta**	**95% LL**	**95% UL**	**Standard Error**	**P-val**	**Steiger test**	**MR Egger intercept**	**MR Egger intercept p-value**	**Cochran-Q**	**Cochran-Q p-val**
MR Egger	NA	NA	NA	NA	NA	NA	NA	NA	NA	NA	NA	NA
Weighted median	NA	NA	NA	NA	NA	NA	NA	NA				
Inverse variance weighted	NA	NA	NA	NA	NA	NA	NA	NA				
Weighted mode	NA	NA	NA	NA	NA	NA	NA	NA				
MR-PRESSO (Outlier-corrected)	NA	NA	NA	NA	NA	NA	NA	NA				
Wald Ratio	NA	1.0270799	0.02671973	0.798466469	1.321148936	0.128460222	0.835228967	TRUE				
**Gamma and beta-tocopherol (vitamin E)**	**N of SNPs**	**OR**	**Beta**	**95% LL**	**95% UL**	**Standard Error**	**P-val**	**Steiger test**	**MR Egger intercept**	**MR Egger intercept p-value**	**Cochran-Q**	**Cochran-Q p-val**
MR Egger	NA	NA	NA	NA	NA	NA	NA	NA	NA	NA	NA	NA
Weighted median	NA	NA	NA	NA	NA	NA	NA	NA				
Inverse variance weighted	NA	NA	NA	NA	NA	NA	NA	NA				
Weighted mode	NA	NA	NA	NA	NA	NA	NA	NA				

MR-PRESSO (Outlier-corrected)	NA	NA	NA	NA	NA	NA	NA	NA				
Wald Ratio	1	1.09997741	0.09528964	0.947540288	1.2769381	0.07610993	0.2105699	TRUE				
**Magnesium**	**N of SNPs**	**OR**	**Beta**	**95% LL**	**95% UL**	**Standard Error**	**P-val**	**Steiger test**	**MR Egger intercept**	**MR Egger intercept p-value**	**Cochran-Q**	**Cochran-Q p-val**
MR Egger	82	0.9942768	-0.0057396	0.877708884	1.126326024	0.06362279	0.928343	TRUE	0.000237152	0.9271271	195.4305	1.20E-11
Weighted median	82	1.02327833	0.02301152	0.981340202	1.0670087	0.02135082	0.2811313	TRUE				
Inverse variance weighted	82	0.99972675	-0.0002733	0.957193917	1.044149535	0.02218163	0.9901701	TRUE			195.451	1.88E-11
Weighted mode	82	1.0110071	0.01094696	0.940596183	1.086688816	0.03683078	0.767057	TRUE				
MR-PRESSO (Outlier-corrected)	82	1.02504889	0.02474031	1.003443281	1.047119706	0.01086885	0.02522952	NA				
MR-LAP	88	0.9959338	-0.0040745	0.938814782	1.056528035	0.03013397	0.8924439	NA				
**Potassium**	**N of SNPs**	**OR**	**Beta**	**95% LL**	**95% UL**	**Standard Error**	**P-val**	**Steiger test**	**MR Egger intercept**	**MR Egger intercept p-value**	**Cochran-Q**	**Cochran-Q p-val**
MR Egger	290	1.12967598	0.12193085	1.087091557	1.173928558	0.0196046	1.75E-09	TRUE	-0.001651852	0.02518565	291.2857	0.4348087
Weighted median	290	1.05242677	0.05109871	1.02345551	1.082218135	0.014241864	3.33E-04	TRUE				
Inverse variance weighted	290	1.08666349	0.08311198	1.066868992	1.106825244	0.009379488	7.93E-19	TRUE			296.4073	0.3694977
Weighted mode	290	1.02235216	0.02210601	0.941890836	1.109686914	0.041822401	5.98E-01	TRUE				
MR-PRESSO (Outlier-corrected)	NA	NA	NA	NA	NA	NA	NA	NA				
MR-LAP	293	1.17624081	0.1623236	1.139034208	1.21466277	0.01639943	4.24E-23	NA				
**Iron**	**N of SNPs**	**OR**	**Beta**	**95% LL**	**95% UL**	**Standard Error**	**P-val**	**Steiger test**	**MR Egger intercept**	**MR Egger intercept p-value**	**Cochran-Q**	**Cochran-Q p-val**
MR Egger	21	0.98317451	-0.0169687	0.911371191	1.060634918	0.03869202	0.6659287	TRUE	0.001027724	0.667605	21.78664	0.295003
Weighted median	21	1.01468049	0.01457378	0.955910044	1.077064211	0.03044145	0.6321181	TRUE				
Inverse variance weighted	21	0.99669892	-0.0033065	0.954161003	1.041133233	0.02225322	0.8818794	TRUE			22.00482	0.3402489
Weighted mode	21	1.01323204	0.01314527	0.952808326	1.077487619	0.03137081	0.6796614	TRUE				
MR-PRESSO (Outlier-corrected)	NA	NA	NA	NA	NA	NA	NA	NA				
MR-LAP	21	0.99906079	-0.0009397	0.963396361	1.036045494	0.01854628	0.9595922	NA				
**Zinc**	**N of SNPs**	**OR**	**Beta**	**95% LL**	**95% UL**	**Standard Error**	**P-val**	**Steiger test**	**MR Egger intercept**	**MR Egger intercept p-value**	**Cochran-Q**	**Cochran-Q p-val**
MR Egger	6	0.98981347	-0.0102388	0.915881136	1.069713814	0.039607101	0.8087714	TRUE	0.002913641	0.7454585	3.229433	0.5201901
Weighted median	6	0.99968048	-0.0003196	0.977519186	1.022344195	0.011437648	0.9777099	TRUE				
Inverse variance weighted	6	1.00314951	0.00314456	0.984793029	1.02184815	0.009422623	0.7385876	TRUE			3.350461	0.6461254
Weighted mode	6	0.99939328	-0.0006069	0.973333682	1.026150585	0.013480311	0.9658329	TRUE				
MR-PRESSO (Outlier-corrected)	NA	NA	NA	NA	NA	NA	NA	NA				
**Copper**	**N of SNPs**	**OR**	**Beta**	**95% LL**	**95% UL**	**Standard Error**	**P-val**	**Steiger test**	**MR Egger intercept**	**MR Egger intercept p-value**	**Cochran-Q**	**Cochran-Q p-val**
MR Egger	6	1.0013111	0.00131024	0.985985397	1.016875024	0.007869377	0.8758417	TRUE	-0.002927271	0.4358904	6.038817	0.1962681
Weighted median	6	0.99815309	-0.0018486	0.987561597	1.00885818	0.005442751	0.7341222	TRUE				
Inverse variance weighted	6	0.99665097	-0.0033547	0.985802573	1.007618742	0.005583939	0.5479943	TRUE			7.168102	0.2084355
Weighted mode	6	0.99835623	-0.0016451	0.988466289	1.00834512	0.005079396	0.7591368	TRUE				
MR-PRESSO (Outlier-corrected)	NA	NA	NA	NA	NA	NA	NA	NA				
**Selenium**	**N of SNPs**	**OR**	**Beta**	**95% LL**	**95% UL**	**Standard Error**	**P-val**	**Steiger test**	**MR Egger intercept**	**MR Egger intercept p-value**	**Cochran-Q**	**Cochran-Q p-val**
MR Egger	NA	NA	NA	NA	NA	NA	NA	NA	NA	NA	NA	NA
Weighted median	NA	NA	NA	NA	NA	NA	NA	NA				
Inverse variance weighted	NA	NA	NA	NA	NA	NA	NA	NA				
Weighted mode	NA	NA	NA	NA	NA	NA	NA	NA				
MR-PRESSO (Outlier-corrected)	NA	NA	NA	NA	NA	NA	NA	NA				
Wald Ratio	1	0.96330625	-0.0373839	0.934004073	0.993527716	0.0157605	0.01769209	TRUE				
**Carotene**	**N of SNPs**	**OR**	**Beta**	**95% LL**	**95% UL**	**Standard Error**	**P-val**	**Steiger test**	**MR Egger intercept**	**MR Egger intercept p-value**	**Cochran-Q**	**Cochran-Q p-val**
MR Egger	NA	NA	NA	NA	NA	NA	NA	NA	NA	NA	NA	NA
Weighted median	NA	NA	NA	NA	NA	NA	NA	NA				
Inverse variance weighted	2	0.99999106	-8.94E-06	0.970714067	1.030151048	0.0151604	0.999529321	TRUE			0.4687697	0.4935537
Weighted mode	NA	NA	NA	NA	NA	NA	NA	NA				
MR-PRESSO (Outlier-corrected)	NA	NA	NA	NA	NA	NA	NA	NA				
**Folate**	**N of SNPs**	**OR**	**Beta**	**95% LL**	**95% UL**	**Standard Error**	**P-val**	**Steiger test**	**MR Egger intercept**	**MR Egger intercept p-value**	**Cochran-Q**	**Cochran-Q p-val**
MR Egger	NA	NA	NA	NA	NA	NA	NA	NA	NA	NA	NA	NA
Weighted median	NA	NA	NA	NA	NA	NA	NA	NA				
Inverse variance weighted	2	0.94898105	-0.0523665	0.868283551	1.037178501	0.04534209	0.2481236	TRUE			0.348587	0.5549141
Weighted mode	NA	NA	NA	NA	NA	NA	NA	NA				
MR-PRESSO (Outlier-corrected)	NA	NA	NA	NA	NA	NA	NA	NA				

Outcome: Sakaue, S (T1D)

Method	N of SNPs	OR	Beta	95% LL	95% UL	Standard Error	P-val	Steiger test	MR Egger intercept	MR Egger intercept p-value	Cochran-Q	Cochran-Q p-val
**Vitamin C**												
MR Egger	22	0.1625694	-1.8166503	0.034483271	0.766424087	0.7911381	0.03259979	TRUE	0.06671373	0.06258025	25.1011	0.19759062
Weighted median	22	0.51379804	-0.665925	0.229136641	1.152100465	0.4119958	0.10602101	TRUE				
Inverse variance weighted	22	0.68848496	-0.3732618	0.367414509	1.290127455	0.3204096	0.24403852	TRUE			29.98251	0.09234012
Weighted mode	22	0.44212926	-0.816153	0.183068861	1.067785532	0.4498673	0.08396005	TRUE				
MR-PRESSO (Outlier-corrected)	22	NA	NA	NA	NA	NA	NA	NA				
**Vitamin D**	**N of SNPs**	**OR**	**Beta**	**95% LL**	**95% UL**	**Standard Error**	**P-val**	**Steiger test**	**MR Egger intercept**	**MR Egger intercept p-value**	**Cochran-Q**	**Cochran-Q p-val**
MR Egger	8	3.00179067	1.099209	0.613983923	14.67586835	0.8096916	0.22343851	TRUE	0.004160006	0.8668378	3.195712	0.7839124
Weighted median	8	3.27474826	1.186241	0.773243396	13.86882351	0.7364298	0.1072234	TRUE				
Inverse variance weighted	8	3.31825486	1.199439	1.080783577	10.18780776	0.5723228	0.03610534	TRUE			3.226336	0.8633048
Weighted mode	8	3.08816789	1.127578	0.860174059	11.0870362	0.6521421	0.1274287	TRUE				
MR-PRESSO (Outlier-corrected)	8	NA	NA	NA	NA	NA	NA	NA				
**Retinol (vitamin A)**	**N of SNPs**	**OR**	**Beta**	**95% LL**	**95% UL**	**Standard Error**	**P-val**	**Steiger test**	**MR Egger intercept**	**MR Egger intercept p-value**	**Cochran-Q**	**Cochran-Q p-val**
MR Egger	NA	NA	NA	NA	NA	NA	NA	NA	NA	NA	NA	NA
Weighted median	NA	NA	NA	NA	NA	NA	NA	NA				
Inverse variance weighted	NA	NA	NA	NA	NA	NA	NA	NA				
Weighted mode	NA	NA	NA	NA	NA	NA	NA	NA				
Wald Ratio	1	2.38701803	0.8700449	0.564935274	10.08585466	0.735249497	0.236677223	TRUE				
MR-PRESSO (Outlier-corrected)	NA	NA	NA	NA	NA	NA	NA	NA				
**Vitamin K (1st model)**	**N of SNPs**	**OR**	**Beta**	**95% LL**	**95% UL**	**Standard Error**	**P-val**	**Steiger test**	**MR Egger intercept**	**MR Egger intercept p-value**	**Cochran-Q**	**Cochran-Q p-val**
MR Egger	4	0.72763189	-0.31796	0.127788909	4.143146503	0.8874569	0.75441488	TRUE	0.009797812	0.9597682	0.8838983	0.6427823
Weighted median	4	0.75168547	-0.2854373	0.538518063	1.04923323	0.1701515	0.09343548	TRUE				
Inverse variance weighted	4	0.7648417	-0.2680864	0.57788527	1.012281935	0.1430069	0.06084229	TRUE			0.8871408	0.8285299
Weighted mode	4	0.74708695	-0.2915737	0.527301127	1.058482307	0.1777601	0.19948428	TRUE				
MR-PRESSO (Outlier-corrected)	NA	NA	NA	NA	NA	NA	NA	NA				
**Vitamin K (2nd model)**	**N of SNPs**	**OR**	**Beta**	**95% LL**	**95% UL**	**Standard Error**	**P-val**	**Steiger test**	MR Egger intercept	**MR Egger intercept p-value**	**Cochran-Q**	**Cochran-Q p-val**
MR Egger	5	0.98273639	-0.0174144	0.863236504	1.118778938	0.06614909	0.8093966	TRUE	-0.04864151	0.2448311	1.03178	0.7935628
Weighted median	5	0.92729898	-0.0754792	0.832654219	1.032701668	0.05492734	0.1693904	TRUE				
Inverse variance weighted	5	0.92195337	-0.0812606	0.837262695	1.015210668	0.04916162	0.0983455	TRUE			3.112782	0.5391322
Weighted mode	5	0.93422474	-0.0680383	0.844653264	1.033294844	0.05142389	0.2563712	TRUE				
MR-PRESSO (Outlier-corrected)	NA	NA	NA	NA	NA	NA	NA	NA				
**Vitamin K (3rd model)**	**N of SNPs**	**OR**	**Beta**	**95% LL**	**95% UL**	**Standard Error**	**P-val**	**Steiger test**	MR Egger intercept	**MR Egger intercept p-value**	**Cochran-Q**	**Cochran-Q p-val**
MR Egger	4	0.96762152	-0.0329143	0.837179212	1.118388265	0.07387901	0.6995298	TRUE	-0.03358802	0.5110911	0.6818904	0.7110979
Weighted median	4	0.92758548	-0.0751703	0.823558712	1.044752251	0.06068883	0.2154867	TRUE				
Inverse variance weighted	4	0.92814589	-0.0745664	0.838327991	1.027586817	0.05192832	0.1510172	TRUE			1.3101214	0.7267311
Weighted mode	4	0.93320576	-0.0691296	0.842988417	1.033078233	0.05187372	0.2748229	TRUE				
MR-PRESSO (Outlier-corrected)	NA	NA	NA	NA	NA	NA	NA	NA				
**Vitamin B12**	**N of SNPs**	**OR**	**Beta**	**95% LL**	**95% UL**	**Standard Error**	**P-val**	**Steiger test**	**MR Egger intercept**	**MR Egger intercept p-value**	**Cochran-Q**	**Cochran-Q p-val**
MR Egger	6	0.34444815	-1.0658117	0.051023553	2.325289446	0.9743144	0.3354505	TRUE	0.08528972	0.3977045	6.829704	0.1451659
Weighted median	6	0.73553475	-0.3071575	0.368379227	1.468626138	0.3527984	0.3839553	TRUE				
Inverse variance weighted	6	0.80631532	-0.2152804	0.389288521	1.670083645	0.3715072	0.5622663	TRUE			8.35778	0.1375889
Weighted mode	6	0.71159696	-0.3402436	0.335827375	1.507828922	0.3831196	0.4151677	TRUE				
MR-PRESSO (Outlier-corrected)	6	NA	NA	NA	NA	NA	NA	NA				
**Vitamin B6**	**N of SNPs**	**OR**	**Beta**	**95% LL**	**95% UL**	**Standard Error**	**P-val**	**Steiger test**	**MR Egger intercept**	**MR Egger intercept p-value**	**Cochran-Q**	**Cochran-Q p-val**
MR Egger	NA	NA	NA	NA	NA	NA	NA	NA	NA	NA	NA	NA
Weighted median	NA	NA	NA	NA	NA	NA	NA	NA				
Inverse variance weighted	NA	NA	NA	NA	NA	NA	NA	NA				
Weighted mode	NA	NA	NA	NA	NA	NA	NA	NA				
MR-PRESSO (Outlier-corrected)	NA	NA	NA	NA	NA	NA	NA	NA				
Wald Ratio	1	0.98716492	-0.0129182	0.933817147	1.043560386	0.02834514	0.6485732	TRUE				
**Alpha-tocopherol (vitamin E)**	**N of SNPs**	**OR**	**Beta**	**95% LL**	**95% UL**	**Standard Error**	**P-val**	**Steiger test**	**MR Egger intercept**	**MR Egger intercept p-value**	**Cochran-Q**	**Cochran-Q p-val**
MR Egger	NA	NA	NA	NA	NA	NA	NA	NA	NA	NA	NA	NA
Weighted median	NA	NA	NA	NA	NA	NA	NA	NA				
Inverse variance weighted	NA	NA	NA	NA	NA	NA	NA	NA				
Weighted mode	NA	NA	NA	NA	NA	NA	NA	NA				
MR-PRESSO (Outlier-corrected)	NA	NA	NA	NA	NA	NA	NA	NA				
Wald Ratio	1	0.1615997	-1.822633	0.016666651	1.56686925	1.159037	0.1158251	TRUE				
**Gamma and beta-tocopherol (vitamin E)**	**N of SNPs**	**OR**	**Beta**	**95% LL**	**95% UL**	**Standard Error**	**P-val**	**Steiger test**	**MR Egger intercept**	**MR Egger intercept p-value**	**Cochran-Q**	**Cochran-Q p-val**
MR Egger	NA	NA	NA	NA	NA	NA	NA	NA	NA	NA	NA	NA
Weighted median	NA	NA	NA	NA	NA	NA	NA	NA				
Inverse variance weighted	NA	NA	NA	NA	NA	NA	NA	NA				
Weighted mode	NA	NA	NA	NA	NA	NA	NA	NA				
MR-PRESSO (Outlier-corrected)	NA	NA	NA	NA	NA	NA	NA	NA				
Wald Ratio	1	1.8600322	0.6205938	0.670904326	5.156800536	0.5202666	0.2329328	TRUE				
**Magnesium**	**N of SNPs**	**OR**	**Beta**	**95% LL**	**95% UL**	**Standard Error**	**P-val**	**Steiger test**	MR Egger intercept	**MR Egger intercept p-value**	**Cochran-Q**	**Cochran-Q p-val**
MR Egger	60	0.64862922	-0.432894	0.276975377	1.518979288	0.4341493	0.3228536	TRUE	0.0180678	0.3403407	65.99253	0.2201247
Weighted median	60	0.87325545	-0.1355272	0.548931634	1.389198655	0.2368644	0.5672055	TRUE				
Inverse variance weighted	60	0.9525344	-0.0486291	0.683419909	1.327619764	0.1693963	0.7740567	TRUE			67.04419	0.2206282
Weighted mode	60	0.7730646	-0.2573927	0.440956874	1.355300065	0.2864365	0.3725169	TRUE				
MR-PRESSO	60	NA	NA	NA	NA	NA	NA	NA				
**Potassium**	**N of SNPs**	**OR**	**Beta**	**95% LL**	**95% UL**	**Standard Error**	**P-val**	**Steiger test**	MR Egger intercept	**MR Egger intercept p-value**	**Cochran-Q**	**Cochran-Q p-val**
MR Egger	117	1.42293096	0.3527188	0.407882513	4.964009117	0.6374974	0.581141212	TRUE	0.01159595	0.4592194	156.186	0.006409552
Weighted median	117	1.9660668	0.676035	1.067665008	3.620441472	0.3115107	0.029993029	TRUE				
Inverse variance weighted	117	2.21384916	0.7947327	1.416301777	3.460511145	0.2278998	0.000488101	TRUE			156.9351	0.006822725
Weighted mode	117	1.99638391	0.6913375	0.805839848	4.945832269	0.4628611	0.137990694	TRUE				
MR-PRESSO (Outlier-corrected)	117	1.91461565	0.6495169	1.265218249	2.897328669	0.2113634	0.002627786	NA				
**Iron**	**N of SNPs**	**OR**	**Beta**	**95% LL**	**95% UL**	**Standard Error**	**P-val**	**Steiger test**	MR Egger intercept	**MR Egger intercept p-value**	**Cochran-Q**	**Cochran-Q p-val**
MR Egger	13	1.90625568	0.64514094	0.900064752	4.037276989	0.3828722	0.12011383	TRUE	-0.03521554	0.2055237	4.874133	0.9371085
Weighted median	13	1.54251776	0.43341599	0.937519522	2.537932258	0.2540478	0.08800036	TRUE				
Inverse variance weighted	13	1.25294768	0.22549892	0.810736027	1.936361328	0.2220978	0.3099563	TRUE			6.68467	0.8777293
Weighted mode	13	1.46283273	0.38037478	0.86583421	2.471465741	0.2675697	0.18061026	TRUE				
MR-PRESSO (Outlier-corrected)	NA	NA	NA	NA	NA	NA	NA	NA				
**Zinc**	**N of SNPs**	**OR**	**Beta**	**95% LL**	**95% UL**	**Standard Error**	**P-val**	**Steiger test**	MR Egger intercept	**MR Egger intercept p-value**	**Cochran-Q**	**Cochran-Q p-val**
MR Egger	6	0.52568337	-0.6430562	0.154295289	1.791000915	0.6254239	0.3619671	TRUE	0.1549615	0.3095855	10.6048	0.0313836
Weighted median	6	1.013184	0.01309785	0.792355017	1.295557927	0.1254304	0.9168335	TRUE				

Inverse variance weighted	6	1.06084251	0.05906341	0.762568815	1.475783959	0.1684316	0.7258382	TRUE			14.18896	0.01445265
Weighted mode	6	0.98836839	-0.0116998	0.771714378	1.265846659	0.1262454	0.9297605	TRUE				
MR-PRESSO (Outlier-corrected)	NA	NA	NA	NA	NA	NA	NA	NA				
**Copper**	**N of SNPs**	**OR**	**Beta**	**95% LL**	**95% UL**	**Standard Error**	**P-val**	**Steiger test**	**MR Egger intercept**	**MR Egger intercept p-value**	**Cochran-Q**	**Cochran-Q p-val**
MR Egger	5	1.02442556	0.02413203	0.914429316	1.147653203	0.05795262	0.7051086	TRUE	-0.01212358	0.7223455	0.6673479	0.8808558
Weighted median	5	1.01514385	0.01503033	0.918981905	1.121368155	0.05077509	0.7672165	TRUE				
Inverse variance weighted	5	1.00882059	0.00878192	0.928065224	1.096602867	0.04256897	0.8365577	TRUE			0.8197196	0.935785
Weighted mode	5	1.01734773	0.01719898	0.934995871	1.106952916	0.04306742	0.7100393	TRUE				
MR-PRESSO (Outlier-corrected)	NA	NA	NA	NA	NA	NA	NA	NA				
**Selenium**	**N of SNPs**	**OR**	**Beta**	**95% LL**	**95% UL**	**Standard Error**	**P-val**	**Steiger test**	**MR Egger intercept**	**MR Egger intercept p-value**	**Cochran-Q**	**Cochran-Q p-val**
MR Egger	NA	NA	NA	NA	NA	NA	NA	NA	NA	NA	NA	NA
Weighted median	NA	NA	NA	NA	NA	NA	NA	NA				
Inverse variance weighted	NA	NA	NA	NA	NA	NA	NA	NA				
Weighted mode	NA	NA	NA	NA	NA	NA	NA	NA				
MR-PRESSO (Outlier-corrected)	NA	NA	NA	NA	NA	NA	NA	NA				
Wald Ratio	1	0.90645125	-0.098218	0.529864606	1.550686453	0.2739366	0.7199371	TRUE				
**Carotene**	**N of SNPs**	**OR**	**Beta**	**95% LL**	**95% UL**	**Standard Error**	**P-val**	**Steiger test**	**MR Egger intercept**	**MR Egger intercept p-value**	**Cochran-Q**	**Cochran-Q p-val**
MR Egger	NA	NA	NA	NA	NA	NA	NA	NA	NA	NA	NA	NA
Weighted median	NA	NA	NA	NA	NA	NA	NA	NA				
Inverse variance weighted	2	1.16872545	0.1559138	0.79846563	1.710680006	0.1943761	0.4224809	TRUE			0.6534007	0.4188995
Weighted mode	NA	NA	NA	NA	NA	NA	NA	NA				
MR-PRESSO (Outlier-corrected)	NA	NA	NA	NA	NA	NA	NA	NA				
**Folate**	**N of SNPs**	**OR**	**Beta**	**95% LL**	**95% UL**	**Standard Error**	**P-val**	**Steiger test**	**MR Egger intercept**	**MR Egger intercept p-value**	**Cochran-Q**	**Cochran-Q p-val**
MR Egger	NA	NA	NA	NA	NA	NA	NA	NA	NA	NA	NA	NA
Weighted median	NA	NA	NA	NA	NA	NA	NA	NA				
Inverse variance weighted	2	1		1	1			TRUE				
Weighted mode	NA	NA	NA	NA	NA	NA	NA	NA				
MR-PRESSO (Outlier-corrected)	NA	NA	NA	NA	NA	NA	NA	NA				
Wald Ratio	1	1.260435	0.2314569	0.165035569	9.626387831	1.037271	0.8234263	TRUE				

Table S3: Reverse MR with Chiou et al. T1D as exposure and potassium as outcome

Outcome: Potassium								
**Method**	**N of SNPs**	**OR**	**Beta**	**95% LL**	**95% UL**	**Standard Error**	**P-val**	**Steiger test**
**Chiou, J (T1D)**								
MR Egger	156	1.00003443	3.44E-05	0.99967385	1.00039513	0.000183994	0.8518317	TRUE
Weighted median	156	1.00002243	2.24E-05	0.99977599	1.000268938	0.000125751	0.8584264	TRUE
Inverse variance weighted	156	1.00005778	5.78E-05	0.99969635	1.000419339	0.000184426	0.7540685	TRUE
Weighted mode	156	1.00026279	2.63E-04	0.99974914	1.000776711	0.000262067	0.317598	TRUE
MR-PRESSO (Outlier-corrected)	NA	NA	NA	NA	NA	NA	NA	NA

Harmonization results

SNP	effect_allele.e	other_allele.e	effect_allele.o	other_allele.o	beta.exposure	beta.outcome	eaf.exposure	eaf.outcome	pval.outco	se.outcom	se.exposur	pval.expos	R2	Fstat
rs10224046	G	T	G	T	0.085811	-0.001751	0.324266	0.2498	0.5544	0.002961	0.015436	2.71E-08	5.94E-05	30.90406
rs10484565	A	G	A	G	0.6635	0.005224	0.086649	0.0658	0.2948	0.004987	0.023923	2.64E-169	0.001475	769.2185
rs1050979	G	A	G	A	0.106196	0.000464	0.515138	0.416	0.8599	0.002627	0.014129	5.65E-14	0.000109	56.49274
rs10751776	C	A	C	A	0.078145	0.000766	0.509915	0.5637	0.765	0.002564	0.01405	2.67E-08	5.94E-05	30.93491
rs10801128	G	A	G	A	0.096073	-0.000608	0.717441	0.6052	0.8172	0.002632	0.015681	8.98E-10	7.21E-05	37.53655
rs111309115	A	G	A	G	0.696189	0.009492	0.003104	0.002	0.7331	0.02783	0.126746	3.96E-08	7.44E-05	30.17065
rs11203203	A	G	A	G	0.143803	-3.10E-05	0.348548	0.313	0.9905	0.002635	0.014405	1.81E-23	0.000191	99.65717
rs112067814	A	G	A	G	-0.563186	-0.01246	0.141299	0.0033	0.5297	0.01983	0.064676	3.10E-18	0.000209	75.82566
rs112214065	T	G	T	G	-0.499591	-0.04053	0.020233	0.0293	3.93E-09	0.006879	0.053033	4.50E-21	0.00017	88.74338
rs112214065	T	G	C	T	-0.499591	0.02722	0.020233	0.9972	0.4841	0.0389	0.053033	4.50E-21	0.00017	88.74338
rs112790956	C	G	C	G	1.366226	0.04006	0.005693	0.0019	0.2066	0.03172	0.09492	5.69E-47	0.000398	207.1709
rs113374757	T	C	T	C	-0.171277	0.002098	0.16347	0.1611	0.5265	0.003312	0.020769	1.63E-16	0.000168	68.00892
rs114064559	T	C	T	C	1.294961	-0.02301	0.015934	0.0074	0.07694	0.01301	0.220964	4.61E-09	9.28E-05	34.34545
rs114165612	T	C	T	C	0.417209	-0.01934	0.0062	0.0178	0.02102	0.00838	0.071415	5.16E-09	6.56E-05	34.12932
rs114378220	T	C	T	C	0.177902	-0.008251	0.070292	0.0467	0.2544	0.007239	0.030444	5.11E-09	6.68E-05	34.14738
rs114708995	T	C	T	C	0.371997	-0.01835	0.21572	0.01	0.134	0.01225	0.054617	9.69E-12	0.000128	46.38975
rs114764675	A	G	C	A	-0.663501	0.01448	0.007841	0.9914	0.2413	0.01236	0.101955	7.63E-11	8.13E-05	42.35116
rs114764675	A	G	A	G	-0.663501	-0.001563	0.007841	0.0039	0.946	0.0231	0.101955	7.63E-11	8.13E-05	42.35116
rs115997781	A	G	A	G	-0.742789	-0.0127	0.060437	0.0078	0.376	0.01435	0.105155	1.62E-12	0.000137	49.89648
rs116864159	C	T	C	T	0.519597	0.02089	0.005701	0.0059	0.2776	0.01925	0.092363	1.85E-08	6.08E-05	31.64728
rs116960428	A	G	A	G	0.597904	0.02091	0.004952	0.0062	0.1709	0.01527	0.089206	2.05E-11	8.63E-05	44.92354
rs117259036	C	T	C	T	0.784723	-0.02931	0.009926	0.0017	0.3819	0.03352	0.1425	3.65E-08	7.48E-05	30.32508
rs117473648	T	A	T	A	0.513396	-0.000364	0.005414	0.0221	0.9887	0.02571	0.093586	4.11E-08	5.78E-05	30.09417
rs117564671	G	T	G	T	0.515613	0.009899	0.005699	0.0073	0.7115	0.02677	0.092953	2.91E-08	5.91E-05	30.76947
rs117580141	T	C	T	C	0.530741	0.02357	0.005457	0.0058	0.1864	0.01784	0.093046	1.17E-08	6.25E-05	32.53636
rs117985689	T	C	T	C	1.547694	0.02785	0.001164	0.0009	0.468	0.03838	0.176451	1.77E-18	0.00019	76.93445
rs118172403	C	T	C	T	0.581282	0.02212	0.005133	0.0067	0.1196	0.01421	0.088241	4.47E-11	8.34E-05	43.39424
rs12128789	C	T	C	T	0.126969	0.000806	0.131779	0.1454	0.8122	0.003392	0.021535	3.73E-09	6.68E-05	34.762
rs12257077	T	C	T	C	0.231245	0.005926	0.031887	0.0596	0.2312	0.00495	0.036955	3.91E-10	7.52E-05	39.15592
rs12464462	G	A	G	A	-0.087955	-0.005101	0.410445	0.3929	0.0421	0.002509	0.014341	8.61E-10	7.23E-05	37.61503
rs12644686	G	C	G	C	-0.10775	-0.003068	0.193778	0.2118	0.3356	0.003186	0.01932	2.44E-08	5.97E-05	31.10423
rs12742756	G	A	G	A	-0.083107	-0.001796	0.428204	0.3552	0.4883	0.002591	0.015077	3.54E-08	5.84E-05	30.38397
rs12927355	T	C	T	C	-0.203881	-0.004571	0.316184	0.2967	0.08283	0.002635	0.015188	4.41E-41	0.000346	180.1986
rs13018977	A	T	A	T	0.100335	0.004025	0.224602	0.2052	0.1813	0.003011	0.017882	2.01E-08	7.76E-05	31.48268
rs13259300	C	A	C	A	-0.092191	-0.004885	0.598278	0.6363	0.06178	0.002615	0.014669	3.28E-10	7.59E-05	39.49801
rs1350275	G	T	G	T	-0.093665	-0.001198	0.69842	0.6405	0.6425	0.00258	0.015283	8.86E-10	7.21E-05	37.56095
rs137976064	T	G	C	T	-0.736207	0.02334	0.002746	0.9959	0.1485	0.01616	0.134536	4.45E-08	5.75E-05	29.94484
rs138627733	A	G	A	G	0.682962	-0.01086	0.003294	0.0025	0.6527	0.02414	0.12148	1.89E-08	7.79E-05	31.60694
rs140555963	T	C	T	C	0.63378	0.02263	0.004573	0.0047	0.2143	0.01823	0.093428	1.17E-11	8.84E-05	46.0174
rs143661959	G	T	G	T	0.683417	-0.008646	0.003267	0.0025	0.7208	0.02419	0.121447	1.83E-08	7.81E-05	31.66627
rs144706316	C	T	C	T	0.578307	-0.02936	0.004216	0.0055	0.06896	0.01614	0.101952	1.41E-08	6.18E-05	32.17545
rs147336858	G	A	G	A	0.682799	-0.008075	0.00324	0.0025	0.7389	0.02423	0.122292	2.36E-08	7.69E-05	31.17372
rs147831298	G	A	G	A	2.0391	0.1118	0.014931	0.001	0.1065	0.06927	0.22452	1.07E-19	0.008587	82.47482
rs148172051	C	G	C	G	-0.873607	0.01272	0.043488	0.0447	0.2265	0.01052	0.131003	2.58E-11	0.000122	44.47014
rs148215845	C	T	C	T	0.513148	0.02455	0.005626	0.0059	0.1605	0.01749	0.092346	2.75E-08	5.93E-05	30.87794
rs148289426	T	C	T	C	0.693936	-0.01167	0.003215	0.0025	0.6305	0.02425	0.12325	1.80E-08	7.82E-05	31.70034
rs148613310	G	A	G	A	0.681809	-0.003171	0.003256	0.0024	0.8971	0.02453	0.122259	2.45E-08	7.67E-05	31.10017
rs150178585	A	G	A	G	0.694612	0.003961	0.003209	0.0023	0.875	0.02517	0.124462	2.39E-08	7.68E-05	31.14655
rs150826336	A	G	A	G	0.689553	0.007383	0.003175	0.0023	0.7712	0.02539	0.126094	4.54E-08	7.37E-05	29.90511
rs150853727	A	G	A	G	-0.3337	0.004026	0.036335	0.0716	0.3623	0.00442	0.038541	4.79E-18	0.000144	74.96622
rs151019335	T	C	T	C	0.681601	-0.0114	0.003254	0.0025	0.6367	0.02414	0.121546	2.05E-08	7.75E-05	31.44691
rs1574285	T	G	T	G	-0.126548	-0.007463	0.591106	0.554	0.002193	0.002436	0.014171	4.27E-19	0.000153	79.74597
rs1701704	G	T	G	T	0.244048	0.01292	0.339425	0.2734	2.48E-06	0.002742	0.014558	4.52E-63	0.00054	281.0258
rs17106304	G	C	G	C	0.115351	0.001489	0.655978	0.6604	0.5577	0.00254	0.014812	6.83E-15	0.000116	60.64772
rs17187665	A	G	A	G	0.367468	-0.01001	0.011907	0.0054	0.574	0.0178	0.066627	3.48E-08	5.95E-05	30.41849
rs17201109	T	G	T	G	0.693649	-0.01204	0.003215	0.0025	0.6197	0.02426	0.123267	1.83E-08	7.81E-05	31.66539
rs17201137	A	G	A	G	0.693951	-0.01128	0.003225	0.0025	0.6419	0.02426	0.123302	1.82E-08	7.81E-05	31.67498
rs17323934	G	C	G	C	-0.129663	-0.002213	0.223075	0.1873	0.4944	0.003239	0.016818	1.26E-14	0.000114	59.44052
rs17623914	C	T	C	T	-0.134887	0.000897	0.099705	0.0932	0.8285	0.004141	0.023381	7.97E-09	6.39E-05	33.28229
rs1808094	C	T	C	T	-0.113651	0.006581	0.523909	0.4777	0.00641	0.002413	0.0149	2.40E-14	0.000143	58.17989
rs180887798	G	T	G	T	-1.639859	0.0276	0.003913	0.0034	0.3431	0.02911	0.248054	3.82E-11	8.39E-05	43.70385
rs181304595	T	C	T	C	1.306452	0.1068	0.0023	0.0012	0.008781	0.04076	0.126226	4.18E-25	0.000264	107.1244
rs181603877	A	C	G	A	0.877484	0.1113	0.011303	0.9991	0.1314	0.0738	0.061325	1.93E-46	0.000393	204.7399
rs181603877	A	C	A	C	0.877484	0.006146	0.011303	0.0069	0.6771	0.01476	0.061325	1.93E-46	0.000393	204.7399
rs182289030	G	C	G	C	-0.532939	0.02084	0.008949	0.0065	0.1815	0.01559	0.096364	3.19E-08	7.54E-05	30.58611
rs182507103	G	A	G	A	1.881145	0.05831	0.009675	0.0025	0.1408	0.03959	0.205078	4.61E-20	0.005103	84.13562
rs183213975	A	C	A	C	1.288108	0.01213	0.007908	0.0066	0.3578	0.0132	0.06613	1.67E-84	0.000728	379.4082
rs183346334	A	G	A	G	0.689006	0.007376	0.00319	0.0023	0.7714	0.02539	0.126115	4.67E-08	7.36E-05	29.84774
rs183429563	T	C	T	C	1.670398	-0.009054	0.00063	0.0007	0.8796	0.05978	0.260016	1.33E-10	0.000102	41.2704
rs183721877	T	A	T	A	1.059351	0.00366	0.001701	0.0056	0.8311	0.01716	0.107585	7.09E-23	0.000186	96.95618
rs184004642	C	T	C	T	1.144835	0.001948	0.004971	0.0039	0.9245	0.02056	0.089671	2.51E-37	0.000313	162.9975
rs184399138	C	A	C	A	1.665973	0.000181	0.000432	0.0017	0.9945	0.02628	0.295643	1.75E-08	7.88E-05	31.75409
rs185739612	A	G	A	G	1.780611	0.01164	0.000289	0.0006	0.8484	0.06089	0.325221	4.37E-08	7.44E-05	29.97643
rs187775336	A	G	A	G	0.911934	0.000417	0.008693	0.0065	0.981	0.01753	0.075074	5.95E-34	0.000283	147.5526
rs1881146	T	A	T	A	-0.09517	-0.001304	0.310529	0.2772	0.6419	0.002805	0.017407	4.57E-08	7.37E-05	29.89174
rs188816512	C	T	C	T	1.545867	0.06334	0.047	0.0014	0.03782	0.0305	0.151845	2.42E-24	0.000285	103.6435
rs188816719	G	A	G	A	1.597226	-0.01434	0.002912	0.0037	0.4645	0.01961	0.104279	5.90E-53	0.000578	234.6054
rs189401160	A	G	A	G	1.677546	-2.50E-05	0.000432	0.0017	0.9992	0.02628	0.296519	1.54E-08	7.95E-05	32.00684
rs191836737	T	C	T	C	1.58995	-0.05411	0.00176	0.0015	0.1041	0.0333	0.147826	5.58E-27	0.000285	115.6816
rs192165457	T	G	T	G	0.98103	0.06962	0.084147	0.003	0.04885	0.03534	0.101515	4.29E-22	0.000257	93.39055
rs192726225	A	G	A	G	1.103571	0.01194	0.000714	0.0027	0.6327	0.02497	0.151762	3.55E-13	0.000102	52.87783
rs192997577	T	C	T	C	0.733883	-0.02076	0.002902	0.0042	0.247	0.01793	0.11525	1.92E-10	7.79E-05	40.54814
rs1947178	G	A	G	A	-0.103267	-0.000776	0.791944	0.6938	0.7756	0.002722	0.017134	1.67E-09	6.98E-05	36.32493
rs202535	A	C	A	C	-0.141437	0.001259	0.827931	0.8008	0.6929	0.003188	0.018453	1.79E-14	0.000113	58.74775
rs2111485	G	A	G	A	0.127631	0.004088	0.604162	0.5122	0.1026	0.002504	0.014455	1.05E-18	0.00015	77.96063
rs2188962	T	C	T	C	0.07946	-0.001968	0.403956	0.3394	0.4627	0.002679	0.014096	1.73E-08	6.10E-05	31.77639
rs229527	A	C	A	C	0.104077	-0.00139	0.416388	0.4057	0.5638	0.002408	0.014138	1.82E-13	0.000104	54.1917
rs2303137	T	A	T	A	-0.081513	-0.001123	0.441899	0.4095	0.6504	0.002479	0.014025	6.17E-09	6.49E-05	33.77903
rs2308910	G	T	A	T	0.678282	0.00567	0.00531	0.994	0.8189	0.02476	0.087554	9.41E-15	0.000115	60.0161
rs2308910	G	T	G	T	0.678282	-0.01984	0.00531	0.0048	0.2625	0.0177	0.087554	9.41E-15	0.000115	60.0161
rs231972	C	A	C	A	0.170939	-0.001084	0.118029	0.1111	0.7816	0.003908	0.021081	5.12E-16	0.000126	65.7505
rs238265	G	T	G	T	-0.090825	1.90E-05	0.695162	0.6886	0.9942	0.002627	0.015158	2.08E-09	6.90E-05	35.90262

rs2476601	G	A	G	A	-0.64304	-0.004076	0.885976	0.92468	0.3879	0.004721	0.020896	5.91E-208	0.001816	946.9975
rs2493411	C	T	C	T	0.127063	0.002367	0.13231	0.1852	0.4491	0.003127	0.022335	1.28E-08	6.22E-05	32.36424
rs2543537	T	C	T	C	-0.083441	0.000778	0.459649	0.4206	0.7471	0.002414	0.014316	5.59E-09	6.53E-05	33.9715
rs2596544	A	T	A	T	0.721034	0.01354	0.221042	0.1786	0.000155	0.003578	0.01891	0	0.002785	1453.877
rs2611211	T	C	T	C	-0.143854	0.002512	0.823656	0.7853	0.4007	0.002989	0.018689	1.39E-14	0.000114	59.24762
rs28367848	T	C	T	C	-0.246085	-0.000642	0.121232	0.0715	0.8901	0.004648	0.018135	6.08E-42	0.000354	184.1341
rs3024493	A	C	A	C	-0.163855	-0.001391	0.154343	0.1258	0.7129	0.003781	0.019641	7.26E-17	0.000134	69.59714
rs3087243	A	G	A	G	-0.19913	-0.001491	0.422477	0.4006	0.544	0.002457	0.014202	1.16E-44	0.000378	196.5955
rs3130676	T	G	C	G	1.57293	0.002245	0.001692	0.9948	0.8811	0.01501	0.154304	2.11E-24	0.000256	103.9114
rs3130676	T	G	T	G	1.57293	0.03864	0.001692	0.0014	0.3029	0.03751	0.154304	2.11E-24	0.000256	103.9114
rs3184504	C	T	C	T	-0.231498	-0.01767	0.53291	0.6125	3.05E-11	0.002657	0.014086	1.08E-60	0.000519	270.0961
rs34201669	G	C	G	C	0.691794	0.006578	0.003144	0.0023	0.7954	0.02537	0.126613	4.66E-08	7.36E-05	29.85355
rs34536443	C	G	C	G	-0.385331	-0.002595	0.042706	0.0382	0.6995	0.006724	0.038519	1.47E-23	0.000192	100.073
rs34593439	A	G	A	G	-0.218071	0.000993	0.108188	0.0911	0.8273	0.00455	0.024119	1.54E-19	0.000157	81.74786
rs35327136	A	C	A	C	-0.119196	0.002187	0.155548	0.1738	0.4923	0.003185	0.018978	3.37E-10	7.58E-05	39.4477
rs36223353	A	G	C	G	0.608299	-0.01481	0.008458	0.998	0.5265	0.02337	0.071363	1.54E-17	0.00014	72.65862
rs36223353	A	G	A	G	0.608299	-0.03052	0.008458	0.0111	0.007723	0.01146	0.071363	1.54E-17	0.00014	72.65862
rs368522239	T	C	T	C	1.146437	0.002227	0.001459	0.0026	0.9223	0.02283	0.181881	2.91E-10	9.80E-05	39.73056
rs373050754	A	G	A	G	1.804406	0.0582	0.000482	0.0013	0.07935	0.03317	0.203218	6.74E-19	0.000207	78.83935
rs373054105	T	C	T	C	2.025	0.1053	0.016189	0.001	0.1397	0.07127	0.21848	1.89E-20	0.00894	85.89761
rs374571611	C	G	C	G	1.846274	0.08083	0.010276	0.001	0.03364	0.03804	0.200816	3.79E-20	0.005127	84.52189
rs3802214	C	T	C	T	-0.106609	-0.003339	0.798827	0.797	0.2701	0.003027	0.01923	2.96E-08	6.01E-05	30.73465
rs3842753	G	T	G	T	0.710531	-0.01194	0.730763	0.6189	1.34E-05	0.002742	0.018519	0	0.00282	1472.076
rs41267106	C	T	C	T	-0.795063	-0.02631	0.007143	0.0069	0.09216	0.01562	0.104603	2.94E-14	0.000111	57.77154
rs41295159	G	C	G	C	-0.699552	-0.01621	0.008954	0.0086	0.2507	0.01411	0.090252	9.11E-15	0.000115	60.07939
rs4490209	G	C	G	C	-0.084522	0.005086	0.359915	0.3341	0.0444	0.00253	0.015457	4.55E-08	7.37E-05	29.90116
rs4548024	C	T	C	T	-0.095737	0.00053	0.233601	0.2459	0.8477	0.002761	0.016703	9.95E-09	6.31E-05	32.85261
rs4820827	C	T	C	T	-0.129657	-0.002622	0.620943	0.6622	0.3366	0.002729	0.014267	1.01E-19	0.000159	82.58968
rs532143635	T	C	T	C	1.420805	0.02149	0.002304	0.0022	0.4228	0.02681	0.135899	1.39E-25	0.000269	109.3038
rs535836924	T	C	T	C	2.66047	-0.02405	0.008634	0.0013	0.5546	0.04071	0.360882	1.68E-13	0.000149	54.34819
rs537431423	A	G	A	G	1.803154	0.05879	0.000475	0.0012	0.07654	0.03319	0.203516	8.00E-19	0.000207	78.49959
rs547034072	A	G	A	G	1.58134	0.047	0.000607	0.0016	0.1763	0.03476	0.209867	4.88E-14	0.000109	56.77555
rs549617254	C	G	C	G	1.048945	0.00573	0.001799	0.0062	0.7232	0.01618	0.10785	2.34E-22	0.000182	94.59415
rs550912181	A	G	A	G	0.687809	-0.009459	0.003206	0.0025	0.6977	0.02435	0.123661	2.67E-08	7.63E-05	30.93635
rs551576878	C	G	C	G	2.28279	0.05551	0.000338	0.0011	0.1136	0.03508	0.3352	9.74E-12	0.000124	46.37915
rs553526715	T	G	T	G	0.693038	0.02278	0.002563	0.002	0.3959	0.02683	0.125706	3.52E-08	7.49E-05	30.39492
rs555330400	A	C	A	C	318.09	0.007026	0.000331	0.0024	0.8558	0.03868	36.434	2.53E-18	0.007941	76.21505
rs55893453	G	A	G	A	0.094653	0.01392	0.201792	0.2161	2.96E-06	0.002977	0.01732	4.63E-08	5.74E-05	29.86566
rs55993634	G	C	G	C	0.219371	-0.01211	0.084897	0.0976	0.004297	0.004241	0.024379	2.29E-19	0.000156	80.97031
rs560880964	C	G	C	G	1.445576	-0.003903	0.00045	0.0009	0.9314	0.04533	0.234911	7.57E-10	9.56E-05	37.86815
rs56994090	C	T	C	T	-0.134255	0.00224	0.431066	0.4153	0.3801	0.002552	0.014594	3.60E-20	0.000163	84.62748
rs570042813	T	C	T	C	1.207977	0.08435	0.000947	0.0007	0.3598	0.09212	0.126249	1.09E-21	0.000226	91.55043
rs571792123	G	A	G	A	0.686273	0.009492	0.002864	0.0023	0.7092	0.02545	0.124679	3.71E-08	7.47E-05	30.29745
rs57209021	T	C	T	C	0.100693	-0.003761	0.225582	0.1951	0.2426	0.003219	0.018297	3.73E-08	5.93E-05	30.28572
rs572622572	A	G	A	G	1.789939	0.01662	0.000228	0.0006	0.7771	0.05871	0.312939	1.07E-08	8.12E-05	32.71568
rs573272611	A	G	C	A	1.323316	-0.1208	0.000412	0.9992	0.022	0.05275	0.234848	1.75E-08	7.88E-05	31.75063
rs573646697	T	A	T	A	1.727147	0.0133	0.000599	0.0021	0.5741	0.02366	0.236039	2.53E-13	0.000133	53.54143
rs574384	A	C	A	C	-0.133602	0.006054	0.894668	0.7322	0.07037	0.003346	0.023876	2.20E-08	6.01E-05	31.31136
rs601338	A	G	A	G	0.127096	-0.003488	0.478792	0.4732	0.143	0.002381	0.014419	1.20E-18	0.000149	77.69492
rs607703	T	C	T	C	0.092015	0.000591	0.484409	0.4684	0.8107	0.002467	0.01428	1.17E-10	7.98E-05	41.52025
rs61759532	T	C	T	C	0.118379	0.00879	0.234556	0.1746	0.014	0.003576	0.018587	1.91E-10	7.94E-05	40.56298
rs61839660	T	C	T	C	-0.357441	0.001709	0.08512	0.0749	0.717	0.004715	0.026	5.25E-43	0.000363	188.9997
rs62395823	A	G	A	G	-0.699325	0.01284	0.042745	0.0326	0.06664	0.007003	0.042518	8.72E-61	0.000519	270.5276
rs6434435	A	G	A	G	-0.122856	0.004521	0.159884	0.1876	0.1438	0.003093	0.019089	1.23E-10	7.96E-05	41.42147
rs663743	A	G	A	G	-0.099964	-0.001655	0.349007	0.2763	0.5569	0.002817	0.015092	3.50E-11	8.43E-05	43.87254
rs6908626	T	G	T	G	0.202923	0.002253	0.165571	0.1323	0.5487	0.003756	0.01852	6.14E-28	0.000231	120.0549
rs6921687	T	C	G	C	-0.356814	-0.04671	0.154538	0.9963	0.2636	0.04179	0.0236	1.21E-51	0.005408	228.5859
rs7068821	T	G	T	G	-0.165103	0.003008	0.251126	0.2433	0.2731	0.002745	0.016333	5.07E-24	0.000196	102.1827
rs7130222	G	T	G	T	-0.091985	0.000331	0.308092	0.3239	0.8975	0.002569	0.016179	1.30E-08	6.21E-05	32.32435
rs722988	C	T	C	T	0.082649	0.003951	0.353008	0.4084	0.1161	0.002514	0.014412	9.78E-09	6.32E-05	32.88712
rs7237497	C	T	C	T	-0.220466	-6.30E-05	0.839298	0.8244	0.9842	0.003185	0.018635	2.71E-32	0.000269	139.9663
rs7668577	C	A	C	A	0.093652	-0.004227	0.311698	0.3027	0.106	0.002615	0.015203	7.26E-10	7.29E-05	37.94676
rs76929655	C	T	C	T	-0.555145	-0.006998	0.012677	0.0105	0.5654	0.01217	0.071025	5.44E-15	0.000117	61.09273
rs7795896	T	C	T	C	-0.135435	-0.000869	0.691577	0.6931	0.7513	0.002743	0.016416	1.58E-16	0.000133	68.06538
rs78325861	G	C	G	C	-0.282082	-0.02086	0.039037	0.0338	0.00382	0.007208	0.042195	2.31E-11	8.74E-05	44.69181
rs7936434	C	G	C	G	0.076923	-0.005022	0.464277	0.4426	0.04006	0.002446	0.013959	3.58E-08	5.83E-05	30.36708
rs8046043	C	G	C	G	-0.084587	0.002786	0.391629	0.4066	0.2536	0.002441	0.015175	2.49E-08	5.97E-05	31.07056
rs855330	C	T	C	T	0.111208	0.000541	0.259422	0.3209	0.8429	0.00273	0.016916	4.89E-11	8.30E-05	43.21912
rs9267951	T	C	T	C	0.691817	-0.005613	0.275616	0.2279	0.07693	0.003173	0.016264	0	0.003464	1809.368
rs9273311	A	G	A	G	1.105838	0.003286	0.61659	0.5181	0.2422	0.00281	0.019133	0	0.00817	3340.533
rs9273771	G	C	G	C	0.959142	-0.0499	0.185931	0.0035	0.1873	0.03784	0.064503	5.18E-50	0.000608	221.1082
rs9274169	C	A	C	A	1.141001	0.002947	0.178935	0.1431	0.4222	0.003672	0.056294	2.43E-91	0.001129	410.8153
rs9385401	T	C	T	C	0.12042	-0.003339	0.454348	0.4211	0.1928	0.002564	0.014827	4.59E-16	0.000163	65.96136
rs9501646	A	G	A	G	0.835999	0.07555	0.159014	0.0012	0.02174	0.03292	0.063756	2.79E-39	0.000442	171.9366
rs9517712	C	T	C	T	-0.102063	-2.00E-04	0.740981	0.7164	0.9436	0.00282	0.015805	1.06E-10	8.01E-05	41.70103

Table S4 : Harmonization results for exposure SNPs and Chiou et al. T1D outcome

Outcome: Chiou, J (T1D)

Vitamin C

SNP	effect_allele.	other_allele.	effect_allele.o	other_allele.	beta.exposure	beta.outcome	eaf.exposure	eaf.outcome	pval.outcome	se.outcome	se.exposure	pval.exposure	R2	Fstat
rs10051765	T	C	T	C	-0.039	0.066067	0.6585	0.655	1.70E-05	0.015363	0.0066	3.64E-09	0.000670831	34.917355
rs10136000	A	G	A	G	0.0404	-0.001646	0.2825	0.287	9.26E-01	0.017728	0.0071	1.33E-08	0.000622069	32.377703
rs10758628	A	C	A	C	0.0304	-0.015806	0.4567	0.44	2.93E-01	0.01502	0.0063	1.28E-06	0.00044744	23.284455
rs10995578	C	G	C	G	0.0296	-0.022582	0.4784	0.483	1.29E-01	0.014864	0.0062	1.91E-06	0.000437999	22.792924
rs11062357	T	C	T	C	0.0305	0.015795	0.5118	0.513	3.07E-01	0.015453	0.0063	1.46E-06	0.000450387	23.437894
rs11641245	C	G	C	G	0.042	-0.046386	0.848	0.85	1.98E-02	0.019902	0.0086	1.13E-06	0.000458317	23.85073
rs1165189	A	C	A	C	-0.0378	0.048724	0.7469	0.746	3.13E-03	0.016491	0.0071	1.18E-07	0.00054462	28.344376
rs117885456	A	G	A	G	0.0781	-0.055714	0.0865	0.0897	3.52E-02	0.026452	0.0116	1.70E-11	0.000870705	45.330039
rs12610033	A	T	A	T	-0.0336	-0.005677	0.2831	0.309	7.50E-01	0.017814	0.0073	4.86E-06	0.000407117	21.185213
rs13028225	T	C	T	C	0.1016	0.012422	0.8569	0.86	5.87E-01	0.022894	0.0089	2.38E-30	0.002499101	130.3189
rs174547	T	C	T	C	-0.0364	0.03962	0.6721	0.64	6.83E-03	0.014647	0.0066	3.84E-08	0.000584419	30.416896
rs185137552	T	C	T	C	0.2368	0.057114	0.988	0.99457	5.60E-01	0.098077	0.0392	1.58E-09	0.000732488	36.491462
rs2366388	A	G	A	G	0.0309	0.034939	0.4908	0.467	2.39E-02	0.015465	0.0063	8.50E-07	0.000462273	24.056689
rs2559850	A	G	A	G	0.0583	-0.011676	0.5979	0.584	4.40E-01	0.015121	0.0064	6.30E-20	0.001592751	82.980713
rs2941484	T	C	T	C	0.0341	-0.017354	0.4514	0.452	2.43E-01	0.014873	0.0063	6.37E-08	0.000562919	29.297304
rs33972313	T	C	T	C	-0.3601	0.0631	0.0319	0.0319	1.24E-01	0.040981	0.0179	4.61E-90	0.007720356	404.7065
rs339969	A	C	A	C	-0.0302	-0.030315	0.6068	0.634	4.72E-02	0.015273	0.0063	1.86E-06	0.000441575	22.979088
rs4867910	T	C	T	C	-0.0314	0.003907	0.657	0.648	8.10E-01	0.016274	0.0068	3.45E-06	0.000409757	21.322664
rs542903	T	C	T	C	-0.0418	-0.017731	0.1429	0.14	4.18E-01	0.021914	0.0088	2.27E-06	0.000433573	22.5625
rs56738967	C	G	C	G	0.041	0.032409	0.321	0.315	4.78E-02	0.016378	0.0067	7.62E-10	0.000719397	37.447093
rs571563721	A	T	A	T	0.2886	0.089276	0.9875	0.99515	4.08E-01	0.107867	0.0416	4.01E-12	0.001109853	48.128906
rs6482188	A	C	A	C	-0.0347	0.022615	0.3086	0.296	1.75E-01	0.01666	0.0068	2.83E-07	0.000500365	26.040009
rs6693447	T	G	T	G	0.0393	-0.039741	0.5509	0.522	8.60E-03	0.015125	0.0064	6.25E-10	0.000724392	37.707275
rs676317	T	C	T	C	-0.0366	-0.01267	0.7264	0.726	4.67E-01	0.017419	0.0074	7.33E-07	0.000470065	24.462381
rs73035571	A	G	A	G	0.035	-0.002295	0.7863	0.781	9.01E-01	0.018511	0.0076	4.14E-06	0.000407563	21.208449
rs73850547	A	G	A	G	0.0333	0.029231	0.7381	0.732	7.56E-02	0.016452	0.0071	2.85E-06	0.000422718	21.997421
rs7640441	A	C	A	C	0.0358	-0.008574	0.2383	0.252	6.30E-01	0.017803	0.0074	1.19E-06	0.000449749	23.404675
rs7740812	A	G	A	G	-0.0384	-0.016082	0.4057	0.376	5.90E-01	0.029851	0.0064	1.88E-09	0.000691616	36
rs7740812	A	G	A	C	-0.0384	0.0048	0.4057	0.106	9.44E-01	0.0684	0.0064	1.88E-09	0.000691616	36
rs78575870	T	C	T	C	0.0551	0.042242	0.077	0.0726	1.48E-01	0.029235	0.0117	2.46E-06	0.000426196	22.178464
rs79234109	A	G	A	G	0.0453	-0.019748	0.1617	0.149	3.52E-01	0.021221	0.0084	7.70E-08	0.000558802	29.082908
rs80246741	A	G	A	G	0.1511	0.015825	0.9897	0.9894	8.30E-01	0.073512	0.0319	2.21E-06	0.000431145	22.43611
rs868822	T	G	T	G	-0.0329	-0.030316	0.2648	0.267	8.20E-02	0.017428	0.0071	3.30E-06	0.000412628	21.472129
rs9895661	T	C	T	C	0.0625	-0.015437	0.817	0.825	4.58E-01	0.020778	0.0081	1.05E-14	0.00114329	59.537418
rs9915323	A	T	A	T	0.0321	-0.028908	0.2967	0.279	6.71E-02	0.015789	0.0068	2.60E-06	0.000428222	22.283953

Vitamin D

SNP	effect_allele.	other_allele.	effect_allele.o	other_allele.	beta.exposure	beta.outcome	eaf.exposure	eaf.outcome	pval.outcome	se.outcome	se.exposure	pval.exposure	R2	Fstat
rs10741657	A	G	A	G	0.0308	-0.021532	0.5	0.408	1.32E-01	0.014289	0.0022	2.05E-46	0.00285448	196
rs10745742	T	C	T	C	0.0165	0.009419	0.5	0.37	5.38E-01	0.015283	0.0022	1.88E-14	0.000812616	56.25
rs10888491	A	G	A	G	0.012	0.013945	0.5	0.356	3.47E-01	0.014833	0.0022	8.62E-08	0.000429208	29.752066
rs11195965	A	T	A	T	-0.0157	0.001311	0.5	0.857	9.53E-01	0.022204	0.0032	6.31E-07	0.000339618	24.071289
rs11203339	T	C	T	C	-0.0104	0.018903	0.5	0.351	2.14E-01	0.0152	0.0021	8.56E-07	0.000314324	24.526077
rs12507653	A	T	A	T	0.0118	0.008928	0.5	0.655	5.63E-01	0.015439	0.0025	2.21E-06	0.000353796	22.2784
rs12785878	T	G	T	G	0.0363	-0.01043	0.5	0.748	5.26E-01	0.016446	0.0022	3.81E-62	0.003463818	272.25
rs17082722	T	C	T	C	-0.0703	0.007933	0.5	0.99174	9.22E-01	0.081149	0.0149	2.22E-06	0.000639761	22.260664
rs17216707	T	C	T	C	0.0263	-0.018173	0.5	0.807	3.42E-01	0.019126	0.0027	8.14E-23	0.001325603	94.88203
rs1809851	A	T	A	T	0.0104	-0.023038	0.5	0.406	1.45E-01	0.015816	0.0023	4.86E-06	0.000299593	20.446125
rs204286	A	T	A	T	0.01	0.010463	0.5	0.651	5.14E-01	0.016027	0.0022	4.69E-06	0.000298078	20.661157
rs2597193	A	G	A	G	0.0187	-0.001813	0.5	0.278	9.10E-01	0.016068	0.0022	6.26E-17	0.000972917	72.25
rs3755967	T	C	T	C	-0.0892	-0.003148	0.5	0.269	8.50E-01	0.016655	0.0023	0	0.018863882	1504.0907
rs4821976	A	G	A	G	0.01	0.012235	0.5	0.537	4.27E-01	0.015389	0.0021	2.74E-06	0.000339606	22.675737
rs6780224	T	C	T	C	-0.0105	0.020286	0.5	0.262	2.43E-01	0.017379	0.0023	4.46E-06	0.000303979	20.84121
rs6982502	T	C	T	C	-0.0094	0.013221	0.5	0.524	3.66E-01	0.014634	0.002	4.04E-06	0.000301867	22.09
rs7011866	T	G	T	G	-0.0404	0.012728	0.5	0.0474	7.32E-01	0.037121	0.0084	1.35E-06	0.000397555	23.131519
rs7675387	T	G	T	G	0.0181	-0.006038	0.5	0.346	7.10E-01	0.016254	0.0023	4.18E-15	0.000990304	61.930057
rs7781168	A	G	A	G	0.0096	-0.005215	0.5	0.355	7.43E-01	0.015909	0.002	2.95E-06	0.000301481	23.04
rs793000	A	G	A	G	0.0099	0.012667	0.5	0.579	4.15E-01	0.015535	0.0022	4.90E-06	0.000295236	20.25
rs8018720	C	G	C	G	-0.0168	-0.008072	0.5	0.828	6.70E-01	0.018914	0.0029	4.72E-09	0.000492331	33.560048
rs904856	A	G	A	G	0.0228	0.047894	0.5	0.949	1.29E-01	0.031537	0.0045	4.12E-07	0.000338979	25.671111
rs914787	T	C	T	C	0.0105	-0.005671	0.5	0.469	7.11E-01	0.015317	0.0022	1.25E-06	0.000345647	22.778926

Retinol (vitamin A)

SNP	effect_allele.	other_allele.	effect_allele.o	other_allele.	beta.exposure	beta.outcome	eaf.exposure	eaf.outcome	pval.outcome	se.outcome	se.exposure	pval.exposure	R2	Fstat
rs1667229	T	A	C	T	0.101271	0.010141	0.520114	0.531	4.90E-01	0.014692	0.0154605	5.74E-11	6.18E-05	42.906504
rs1667229	C	T	C	T	0.0990269	0.010141	0.526657	0.531	4.90E-01	0.014692	0.0154211	1.35E-10	5.94E-05	41.235917
rs1883711	C	G	C	G	-0.27778	0.1252	0.029437	0.0385	3.95E-03	0.043435	0.0454347	9.73E-10	5.38E-05	37.378909

Vitamin K (1st model)

SNP	effect_allele.	other_allele.	effect_allele.o	other_allele.	beta.exposure	beta.outcome	eaf.exposure	eaf.outcome	se.outcome	se.exposure	pval.exposure	R2	Fstat	
rs2108622	T	C	T	C	0.16	-0.003022	0.3	0.278	0.015451	0.032	8.78E-07	0.011568718	25	
rs2192574	C	T	C	T	0.28	-0.035858	0.11	0.138	0.02274	0.058	1.82E-06	0.010793094	23.30558859	
rs4645543	T	C	T	C	-0.42	0.008741	0.04	0.059	0.03363	0.079	2.00E-07	0.013059725	28.26470117	
rs4852146	C	T	C	T	0.18	-0.001039	0.33	0.275	0.017123	0.037	2.08E-06	0.010958593	23.66691015	
rs964184	G	C	G	C	0.23	-0.053856	0.15	0.136	0.020608	0.042	5.91E-08	0.013845254	29.98866213	
**(2nd model)**														
SNP	effect_allele.	other_allele.	effect_allele.o	other_allele.	beta.exposure	beta.outcome	eaf.exposure	eaf.outcome	pval.outcome	se.outcome	se.exposure	pval.exposure	R2	Fstat
rs2108622	T	C	T	C	0.16	-0.003022	0.3	0.278	0.845	0.015451	0.031	2.90E-07	0.012317783	26.638918
rs2192574	C	T	C	T	0.28	-0.035858	0.11	0.138	0.115	0.02274	0.057	1.49E-06	0.011170854	24.130502
rs4852146	C	T	C	T	0.19	-0.001039	0.33	0.275	0.952	0.017123	0.035	1.42E-07	0.013608776	29.469388
rs6862071	T	A	T	A	-1.14	0.038693	0.01	0.0147	0.568	0.067797	0.22	1.35E-07	0.012414742	26.85124
rs964184	G	C	G	C	0.14	-0.053856	0.15	0.136	0.00897	0.020608	0.042	5.91E-08	0.005174912	11.111111
**(3rd model)**														
SNP	effect_allele.	other_allele.	effect_allele.o	other_allele.	beta.exposure	beta.outcome	eaf.exposure	eaf.outcome	pval.outcome	se.outcome	se.exposure	pval.exposure	R2	Fstat
rs2108622	T	C	T	C	0.16	-0.003022	0.3	0.278	0.845	0.015451	0.032	9.68E-07	0.011568718	25
rs2192574	C	T	C	T	0.29	-0.035858	0.11	0.138	0.115	0.02274	0.058	8.23E-07	0.011568718	25
rs4852146	C	T	C	T	0.18	-0.001039	0.33	0.275	0.952	0.017123	0.038	3.23E-06	0.010395331	22.437673
rs6862071	T	A	T	A	-1.09	0.038693	0.01	0.0147	0.568	0.067797	0.23	1.73E-06	0.010405272	22.459357

Vitamin B12

SNP	effect_allele.	other_allele.	effect_allele.o	other_allele.	beta.exposure	beta.outcome	eaf.exposure	eaf.outcome	pval.outcome	se.outcome	se.exposure	pval.exposure	R2	Fstat
rs1131603	C	T	C	T	0.22	-0.05437	0.06	0.0546	0.112	0.034221	0.015	4.90E-49	0.004697969	215.11111
rs1141321	C	T	C	T	0.07	0.000615	0.63	0.628	0.968	0.015329	0.007	3.60E-26	0.002189525	100
rs1801222	G	A	G	A	0.12	0.004813	0.59	0.636	0.756	0.015467	0.007	3.30E-75	0.006407045	293.87755
rs2270655	G	C	G	C	0.1	-0.027274	0.94	0.9475	0.433	0.034771	0.015	2.20E-13	0.000974265	44.444444
rs2336573	T	C	T	C	0.31	-0.039395	0.03	0.0325	0.378	0.04467	0.019	8.40E-59	0.005807365	266.20499
rs34324219	C	A	C	A	0.23	0.008604	0.88	0.893	0.737	0.025583	0.011	1.10E-111	0.009501821	437.19008
rs3742801	T	C	T	C	0.05	0.018525	0.29	0.347	0.222	0.015178	0.007	1.70E-13	0.001118378	51.020408
rs41281112	C	T	C	T	0.18	-0.000814	0.95	0.9719	0.985	0.043591	0.015	8.90E-35	0.003149744	144
rs602662	A	G	A	G	0.17	0.11694	0.6	0.509	4.33E-16	0.014386	0.007	2.40E-139	0.012778372	589.79592

Vitamin B6

SNP	effect_allele.	other_allele.	effect_allele.o	other_allele.	beta.exposure	beta.outcome	eaf.exposure	eaf.outcome	pval.outcome	se.outcome	se.exposure	pval.exposure	R2	Fstat
rs4654748	C	T	C	T	1.45	0.015533	0.5	0.526	0.268	0.014029	0.281	8.00E-18	0.008999806	26.627069

Alpha-tocopherol (vitamin E)

SNP	effect_allele.	other_allele.	effect_allele.o	other_allele.	beta.exposure	beta.outcome	eaf.exposure	eaf.outcome	pval.outcome	se.outcome	se.exposure	pval.exposure	R2	Fstat
rs964184	G	C	G	C	0.04	-0.053856	0.15	0.136	0.00897	0.020608	0.01	8.00E-12	0.004231685	16

Gamma and beta-tocopherol

SNP	effect_allele.	other_allele.	effect_allele.o	other_allele.	beta.exposure	beta.outcome	eaf.exposure	eaf.outcome	pval.outcome	se.outcome	se.exposure	pval.exposure	R2	Fstat
rs11705639	A	C	T	C	0.12578066	-0.009938	0.1685	0.171	0.603	0.019102	0.0138	5.00E-20	0.014375112	83.07485
rs62508088	T	C	T	C	0.16487662	0.016258	0.0992	0.0899	0.501	0.02418	0.0179	1.00E-21	0.014676448	84.842233

Magnesium

SNP	effect_allele.	other_allele.	effect_allele.o	other_allele.	beta.exposure	beta.outcome	eaf.exposure	eaf.outcome	pval.outcome	se.outcome	se.exposure	pval.exposure	R2	Fstat
rs10043693	A	G	A	G	0.03589	0.025127	0.6921	0.7	1.30E-01	0.016584	0.004433	5.63E-16	0.000450077	65.546794
rs1035283	G	A	G	A	-0.06605	-0.054459	0.9368	0.9448	8.14E-02	0.031255	0.00826	1.27E-15	0.000439062	63.941902
rs10747045	T	G	T	G	-0.02741	0.000924	0.6696	0.673	9.51E-01	0.015162	0.004336	2.57E-10	0.000274443	39.961292
rs10952168	G	A	G	A	0.02429	0.004777	0.4401	0.42	7.66E-01	0.016032	0.004443	4.57E-08	0.000205279	29.888382
rs10974444	G	C	G	C	0.02687	-0.075383	0.6607	0.653	4.02E-07	0.014874	0.004233	2.19E-10	0.000276726	40.293853
rs11234579	C	T	C	T	-0.02714	0.027725	0.6373	0.625	6.41E-02	0.014974	0.004453	1.10E-09	0.000255114	37.146192
rs112510641	G	A	G	A	0.03035	-0.020853	0.6104	0.609	1.53E-01	0.014594	0.00418	3.86E-13	0.000362025	52.718716
rs113174770	T	G	T	G	0.04893	-0.066567	0.9334	0.9297	3.57E-01	0.072323	0.008128	1.74E-09	0.000248889	36.23957
rs115478735	A	T	A	T	-0.04194	0.055231	0.8064	0.814	3.46E-03	0.01889	0.00508	1.52E-16	0.000468012	68.159976
rs11614506	T	C	T	C	0.03278	-0.019324	0.7819	0.774	2.48E-01	0.016725	0.004892	2.09E-11	0.000308349	44.899862
rs11694498	T	A	T	A	-0.04061	-0.000731	0.6086	0.571	9.60E-01	0.014756	0.004166	1.88E-22	0.000652342	95.022718
rs117672478	C	A	C	A	-0.08527	-0.019917	0.9763	0.967	6.48E-01	0.043667	0.01506	1.51E-08	0.00022018	32.058455
rs12203597	G	A	G	A	-0.03104	-0.005449	0.3435	0.335	7.16E-01	0.014971	0.004361	1.10E-12	0.000347897	50.660708
rs12230212	T	A	T	A	0.1637	0.041141	0.989	0.99188	6.52E-01	0.091147	0.02743	2.39E-09	0.000244608	35.616049
rs1229984	T	C	T	C	0.07801	0.047208	0.03517	0.019	3.05E-01	0.046065	0.01116	2.79E-12	0.00033555	48.862104
rs12464156	T	C	T	C	-0.02627	0.008899	0.5962	0.598	5.70E-01	0.015657	0.004107	1.58E-10	0.000280983	40.913887
rs1273884	G	A	G	A	0.04993	0.012432	0.5595	0.568	4.22E-01	0.015497	0.004091	2.87E-34	0.001022236	148.95812
rs12743084	C	G	C	G	-0.08845	0.012446	0.4615	0.459	7.36E-01	0.036984	0.004065	5.97E-105	0.003241869	473.45049
rs12918968	A	C	A	C	-0.03944	0.052274	0.5741	0.563	1.19E-03	0.01613	0.004172	3.25E-21	0.00061355	89.368653
rs13143189	G	A	G	A	0.02938	0.005676	0.5727	0.576	7.44E-01	0.017349	0.004194	2.47E-12	0.000337001	49.073466
rs13146355	G	A	G	A	-0.06525	-0.02304	0.5558	0.545	1.07E-01	0.014286	0.004024	3.91E-59	0.001802986	262.93299
rs13170671	C	T	C	T	0.02378	-0.032724	0.5502	0.546	3.80E-01	0.037251	0.004199	1.48E-08	0.000220276	32.072436
rs13193692	G	T	G	T	-0.03149	-0.010003	0.7824	0.761	5.66E-01	0.017411	0.005055	4.69E-10	0.000266513	38.806368
rs142601087	T	C	T	C	0.02922	-0.027091	0.806	0.826	1.41E-01	0.018409	0.005168	1.57E-08	0.000219559	31.967996
rs143135527	C	T	C	T	0.08315	0.059947	0.9719	0.9735	2.51E-01	0.052251	0.01462	1.28E-08	0.000222159	32.346684
rs17794420	G	A	G	A	0.02859	-0.003571	0.5174	0.502	8.21E-01	0.01575	0.004373	6.19E-11	0.000293544	42.743429
rs17832417	A	T	A	T	0.03899	-0.010316	0.6265	0.611	4.88E-01	0.014883	0.004404	8.47E-19	0.000538157	78.381189
rs1853392	A	G	A	G	-0.05664	-0.028253	0.09745	0.066	3.63E-01	0.031079	0.008654	5.98E-11	0.000294182	42.836375
rs1890185	A	G	A	G	-0.02732	0.003822	0.5875	0.612	7.88E-01	0.014242	0.004063	1.75E-11	0.000310502	45.21346
rs219782	A	G	A	G	-0.06371	-0.014859	0.7483	0.747	3.98E-01	0.017572	0.004632	4.75E-43	0.001297912	189.18126
rs2439722	T	C	T	C	-0.02569	-0.023942	0.528	0.504	1.32E-01	0.015906	0.00424	1.36E-09	0.000252126	36.711024
rs250383	A	T	A	T	0.05615	-0.088853	0.8149	0.863	1.19E-01	0.056935	0.005266	1.53E-26	0.000780423	113.69407
rs2510467	A	G	A	G	0.03135	0.006598	0.4299	0.429	6.78E-01	0.015893	0.004437	1.59E-12	0.00034283	49.922489
rs2542713	A	C	A	C	0.02645	-0.038127	0.4504	0.418	9.87E-03	0.014775	0.004186	2.64E-10	0.000274198	39.925734
rs2731238	G	A	G	A	0.05395	0.009807	0.2993	0.269	7.61E-01	0.03225	0.004601	9.56E-32	0.000943625	137.49232
rs2731238	G	T	T	C	-0.1227	-0.009807	0.9493	0.731	7.61E-01	0.03225	0.009953	6.53E-35	0.001042939	151.97814
rs2731238	G	A	A	C	0.05395	0.117818	0.2993	0.0771	2.17E-02	0.051328	0.004601	9.56E-32	0.000943625	137.49232
rs2731238	G	T	G	T	-0.1227	-0.117818	0.9493	0.9229	2.17E-02	0.051328	0.009953	6.53E-35	0.001042939	151.97814
rs2818759	G	T	G	T	0.03013	-0.017715	0.344	0.334	2.59E-01	0.015702	0.00441	8.39E-12	0.000320563	46.678951
rs28441180	G	A	G	A	0.02463	-0.008101	0.491	0.493	8.34E-01	0.038577	0.004398	2.15E-08	0.000215405	31.363056
rs303968	T	C	T	C	-0.03532	0.0037	0.6028	0.6	8.15E-01	0.015855	0.004198	3.98E-17	0.000486045	70.787491
rs34872471	T	C	T	C	0.04058	0.013062	0.7066	0.735	4.30E-01	0.016542	0.004434	5.56E-20	0.000575061	83.759236
rs35249105	A	G	A	G	-0.04637	0.030987	0.538	0.541	3.75E-02	0.014896	0.004121	2.25E-29	0.000869005	126.61028
rs35347302	G	A	G	A	-0.02671	0.018951	0.7702	0.775	3.09E-01	0.018627	0.004774	2.20E-08	0.000214991	31.302783
rs35465213	G	A	G	A	-0.1494	-0.00629	0.9477	0.9326	8.43E-01	0.031695	0.009452	2.85E-56	0.001713327	249.83528
rs35934	G	T	G	T	-0.05568	0.025573	0.1741	0.156	2.01E-01	0.019996	0.005631	4.71E-23	0.000671223	97.774904
rs3732215	G	C	G	C	-0.02364	-0.01218	0.4801	0.458	4.13E-01	0.014891	0.003994	3.26E-09	0.000240605	35.033121
rs3783297	T	C	T	C	0.02579	0.012296	0.6433	0.634	4.15E-01	0.015087	0.004293	1.89E-09	0.000247859	36.089504
rs3824347	A	G	A	G	0.05695	0.001444	0.5864	0.555	9.26E-01	0.015539	0.004257	8.35E-41	0.001227941	178.96997
rs3848132	T	A	T	A	-0.04543	-0.008943	0.723	0.707	5.82E-01	0.016238	0.004775	1.82E-21	0.000621441	90.518786
rs3925584	T	C	T	C	0.07105	-0.010371	0.5475	0.539	5.00E-01	0.015391	0.00402	6.73E-70	0.002141294	312.37485
rs4077450	G	T	G	T	-0.03324	0.03597	0.2184	0.181	5.31E-02	0.018598	0.005478	1.31E-09	0.000252871	36.819507
rs425135	A	C	A	C	-0.03465	-0.039588	0.1466	0.148	5.76E-02	0.020846	0.005797	2.27E-09	0.000245372	35.727274
rs4962402	T	G	T	G	-0.03358	0.0121	0.2763	0.272	4.82E-01	0.017191	0.004637	4.43E-13	0.000360132	52.442959
rs544934737	T	C	T	C	0.02519	-0.00691	0.5678	0.567	8.53E-01	0.037383	0.004601	4.36E-08	0.00020587	29.974495
rs560609863	T	G	T	G	-0.05315	-0.074321	0.8518	0.89	3.02E-01	0.072004	0.009142	6.12E-09	0.000232142	33.800576
rs58447148	A	G	A	G	0.04638	0.060302	0.9139	0.916	3.05E-02	0.027875	0.007637	1.26E-09	0.000253301	36.882123
rs58560619	T	C	T	C	0.02335	0.017679	0.4936	0.482	2.48E-01	0.015312	0.004079	1.04E-08	0.000225061	32.769239
rs592859	C	G	C	G	0.03162	-0.026417	0.1578	0.155	2.17E-01	0.021384	0.005773	4.31E-08	0.000206045	29.999956
rs59359435	T	C	T	C	0.06372	-0.077768	0.9553	0.9548	3.64E-02	0.037166	0.01039	8.55E-10	0.000258309	37.611481
rs59685687	T	A	T	A	0.02874	0.007263	0.7714	0.774	8.73E-01	0.045379	0.005196	3.16E-08	0.000210124	30.593928
rs6003469	A	C	A	C	0.04485	-0.006694	0.6924	0.701	6.88E-01	0.016642	0.004437	5.11E-24	0.000701411	102.17533
rs606970	A	G	A	G	-0.02401	0.02754	0.5289	0.516	7.13E-02	0.015267	0.00432	2.74E-08	0.000212156	30.889923
rs62136373	C	T	C	T	0.03313	0.015585	0.8549	0.858	4.36E-01	0.02	0.006058	4.51E-08	0.000205412	29.907791
rs623297	C	T	C	T	0.02897	0.004703	0.7303	0.725	7.74E-01	0.0164	0.004862	2.56E-09	0.000243833	35.503166
rs636264	G	A	G	A	-0.03387	-0.017815	0.2082	0.192	3.25E-01	0.018083	0.005236	9.93E-11	0.000287367	41.8438
rs658903	T	A	T	A	-0.03979	0.027313	0.855	0.846	2.16E-01	0.022053	0.006028	4.07E-11	0.000299228	43.571388
rs6667005	T	C	T	C	0.02947	-0.013251	0.7844	0.773	4.53E-01	0.017641	0.004962	2.86E-09	0.000242255	35.273354
rs6936263	G	C	G	C	-0.02746	0.0232	0.2853	0.474	6.57E-01	0.0522	0.004564	1.78E-09	0.000248618	36.200102
rs6936263	G	C	T	G	-0.02746	-0.0125	0.2853	0.298	7.87E-01	0.0462	0.004564	1.78E-09	0.000248618	36.200102
rs7039	C	G	C	G	0.03185	-0.015225	0.479	0.492	3.24E-01	0.015444	0.004072	5.26E-15	0.000420099	61.179135
rs7077696	A	G	A	G	0.02798	-0.00741	0.7797	0.781	6.93E-01	0.018778	0.00501	2.34E-08	0.000214219	31.19033
rs711819	T	C	T	C	-0.03593	0.022847	0.2421	0.227	2.12E-01	0.018314	0.004965	4.61E-13	0.000359626	52.369199
rs7221296	A	G	A	G	-0.04431	0.008011	0.8126	0.808	6.86E-01	0.019834	0.005215	1.94E-17	0.000495691	72.192964
rs7241576	A	G	A	G	0.02973	0.001391	0.6835	0.677	9.32E-01	0.016386	0.004521	4.82E-11	0.000296977	43.243497
rs72848405	T	C	T	C	0.02757	0.006266	0.8042	0.813	7.77E-01	0.022073	0.005049	4.74E-08	0.000204788	29.816921
rs7374260	T	C	T	C	0.02461	-0.004804	0.5278	0.553	7.57E-01	0.015552	0.003992	7.04E-10	0.000261012	38.005125
rs73949333	A	G	A	G	0.03287	0.024315	0.776	0.78	1.96E-01	0.018817	0.005211	2.84E-10	0.000273256	39.788472
rs7416991	T	C	T	C	0.05162	-0.0048	0.3162	0.513	9.23E-01	0.0498	0.004405	1.04E-31	0.000942466	137.32328
rs7416991	T	G	G	A	-0.05192	0.0048	0.6967	0.487	9.23E-01	0.0498	0.004371	1.51E-32	0.000968318	141.09374
rs7416991	T	C	C	A	0.05162	-0.0366	0.3162	0.302	4.24E-01	0.0458	0.004405	1.04E-31	0.000942466	137.32328
rs7416991	T	G	T	G	-0.05192	0.0366	0.6967	0.698	4.24E-01	0.0458	0.004371	1.51E-32	0.000968318	141.09374
rs7742789	C	T	C	T	0.05486	0.009873	0.6933	0.709	5.57E-01	0.016818	0.004413	1.76E-35	0.001060508	154.54101
rs77759957	A	G	A	G	0.0746	-0.004209	0.9728	0.9701	9.23E-01	0.043669	0.01319	1.54E-08	0.000219697	31.988072
rs7797740	A	G	A	G	-0.03551	-0.052897	0.1499	0.145	3.18E-01	0.053019	0.006093	5.61E-09	0.000233275	33.965576
rs78433554	C	A	C	A	0.1049	-0.009138	0.986	0.9849	8.77E-01	0.059214	0.0192	4.69E-08	0.000205017	29.850288
rs7850067	A	G	A	G	0.04308	-0.005529	0.485	0.474	7.21E-01	0.015492	0.004081	4.79E-26	0.000764922	111.43412
rs7867868	C	T	C	T	-0.04157	0.011585	0.6695	0.659	4.75E-01	0.016226	0.004504	2.69E-20	0.000584844	85.185031
rs7894336	C	T	C	T	0.06273	-0.00913	0.4679	0.485	5.31E-01	0.014568	0.004011	4.00E-55	0.001677441	244.59369
rs7946549	T	A	T	A	0.04303	0.005619	0.5441	0.556	7.04E-01	0.014811	0.004036	1.52E-26	0.000780248	113.66856
rs8068318	C	T	C	T	-0.08407	-0.00574	0.3015	0.268	7.43E-01	0.01752	0.004691	8.01E-72	0.002201533	321.18202
rs9897596	T	C	T	C	-0.02319	0.01376	0.4911	0.492	3.45E-01	0.014581	0.004061	1.13E-08	0.000223959	32.608853

Potassium

SNP	effect_allele.	other_allele.	effect_allele.o	other_allele.	beta.exposure	beta.outcome	eaf.exposure	eaf.outcome	pval.outcome	se.outcome	se.exposure	pval.exposure	R2	Fstat
rs10032927	A	T	A	T	0.01561	-0.018118	0.5837	0.64	2.60E-01	0.016074	0.002494	4.01E-10	6.49E-05	39.175352
rs10046175	C	A	C	A	0.03717	-0.62953	0.9659	0.999636	3.07E-01	0.616193	0.006432	7.72E-09	5.59E-05	33.395892
rs10086982	G	A	G	A	-0.02034	0.005024	0.7603	0.74	7.75E-01	0.017561	0.003195	2.01E-10	6.71E-05	40.528467
rs10227075	T	C	T	C	-0.06498	1.033404	0.9796	0.999756	3.96E-01	1.218593	0.00832	6.01E-15	0.000102088	60.997602
rs10264133	C	T	C	T	-0.05002	0.044016	0.9737	0.999759	9.32E-01	0.515679	0.007193	3.67E-12	8.09E-05	48.35788
rs10265221	T	C	T	C	-0.03723	-0.002329	0.7481	0.717	8.98E-01	0.018128	0.002886	5.03E-38	0.000275558	166.41536
rs10270510	G	C	G	C	-0.06498	1.033404	0.9796	0.999756	3.96E-01	1.218593	0.00832	6.01E-15	0.000102088	60.997602
rs10279895	A	G	A	G	-0.06565	1.052719	0.9797	0.999957	3.63E-01	1.15619	0.008331	3.44E-15	0.000103929	62.097654
rs10502917	T	C	T	C	-0.02136	-0.027406	0.3194	0.23	1.37E-01	0.018428	0.002766	1.20E-14	9.88E-05	59.634577
rs10748853	T	C	T	C	-0.03616	0.010614	0.1179	0.045	7.71E-01	0.036529	0.003902	2.01E-20	0.00014222	85.878077
rs1076485	C	T	C	T	0.02448	0.055462	0.815	0.862	7.91E-03	0.020882	0.00308	2.01E-15	0.00010462	63.17153

rs10811662	G	A	G	A	0.01906	-0.008502	0.8401	0.832	6.53E-01	0.018911	0.003305	8.20E-09	5.51E-05	33.258516
rs11124938	C	A	C	A	-0.02548	0.045295	0.7812	0.745	4.80E-03	0.016062	0.002987	1.57E-17	0.000120508	72.765983
rs111375249	G	T	G	T	-0.05498	0.21018	0.9749	0.999221	4.86E-01	0.301876	0.007261	3.84E-14	9.60E-05	57.334575
rs111607733	C	A	C	A	-0.05535	0.2656	0.9726	0.999178	3.66E-01	0.293874	0.006865	7.85E-16	0.000108796	65.006108
rs111724190	T	C	T	C	-0.03147	-0.659938	0.9503	0.999838	2.08E-01	0.52366	0.005554	1.49E-08	5.37E-05	32.10567
rs111972532	T	C	T	C	0.03879	-0.022579	0.8139	0.798	2.37E-01	0.019102	0.003086	3.39E-36	0.000261622	157.99659
rs11217192	T	G	T	G	-0.01709	0.010432	0.7648	0.738	5.51E-01	0.017503	0.002949	7.04E-09	5.56E-05	33.584167
rs11245343	T	C	T	C	-0.02401	-0.027638	0.7283	0.672	9.17E-02	0.016386	0.002872	6.53E-17	0.000115746	69.890066
rs11264363	G	C	G	C	0.0252	-0.038988	0.4285	0.317	9.78E-03	0.01509	0.002659	2.78E-21	0.000148744	89.818212
rs112734474	T	G	T	G	-0.07519	0.38464	0.9782	0.999363	2.49E-01	0.333491	0.008227	6.70E-20	0.000139792	83.528979
rs115080005	C	T	C	T	0.03996	0.314229	0.9722	0.999789	4.85E-01	0.449646	0.00691	7.55E-09	5.60E-05	33.442202
rs11563587	T	A	T	A	-0.05898	1.042591	0.9824	0.999936	3.68E-01	1.158397	0.00864	9.02E-12	7.80E-05	46.599585
rs11563956	C	T	C	T	-0.03679	-0.446115	0.9677	0.999747	3.07E-01	0.436675	0.006511	1.64E-08	5.34E-05	31.927446
rs11563967	A	G	A	G	-0.03819	-0.301698	0.9514	0.999554	4.11E-01	0.367053	0.005682	1.85E-11	7.56E-05	45.174864
rs11563979	G	A	G	A	-0.04055	-0.246812	0.9527	0.999648	5.46E-01	0.409192	0.00576	1.98E-12	8.29E-05	49.560622
rs11563980	C	T	C	T	-0.03382	-0.137702	0.9393	0.99252	7.49E-02	0.077304	0.005234	1.07E-10	6.99E-05	41.752239
rs11563986	T	C	T	C	-0.04416	-0.303467	0.9695	0.99984	6.31E-01	0.631671	0.006736	5.74E-11	7.19E-05	42.976769
rs11563990	C	T	C	T	-0.03834	0.268727	0.9635	0.999805	6.26E-01	0.550598	0.006325	1.38E-09	6.15E-05	36.743723
rs11564010	G	C	G	C	-0.06314	1.064793	0.9796	0.99995	3.54E-01	1.147944	0.008287	2.66E-14	9.72E-05	58.051638
rs11564019	G	A	G	A	-0.06503	1.053606	0.9796	0.999948	3.56E-01	1.142327	0.008322	5.79E-15	0.000102196	61.062149
rs11564024	T	G	T	G	-0.0652	1.029068	0.9299	0.999962	3.71E-01	1.151309	0.008369	6.98E-15	0.000344857	60.694321
rs11564025	T	G	T	G	-0.06489	1.034636	0.9795	0.999755	3.96E-01	1.218818	0.008315	6.31E-15	0.000101928	60.901928
rs11564194	C	T	C	T	-0.02113	-0.021584	0.8063	0.745	1.81E-01	0.016151	0.003081	7.27E-12	7.79E-05	47.034399
rs116355131	C	A	C	A	0.06558	5.5658	0.9709	0.999802	6.56E-02	3.0225	0.01191	3.76E-08	0.0001723	30.319309
rs11711982	T	C	T	C	0.0239	0.041855	0.883	0.836	3.33E-02	0.019662	0.003863	6.33E-10	6.34E-05	38.277749
rs11743019	A	G	A	G	-0.02052	0.015603	0.7361	0.695	3.47E-01	0.016584	0.002981	6.04E-12	7.85E-05	47.383895
rs11822294	C	T	C	T	-0.01797	-0.007834	0.8251	0.798	6.90E-01	0.01965	0.003169	1.47E-08	5.33E-05	32.155234
rs11970777	C	T	C	T	-0.03918	-0.497547	0.9638	0.999799	3.26E-01	0.506171	0.006303	5.25E-10	6.40E-05	38.639745
rs11974568	T	C	T	C	-0.03409	-0.604368	0.9488	0.999611	7.10E-02	0.334692	0.005567	9.44E-10	6.21E-05	37.498298
rs11983963	T	A	T	A	-0.04007	-0.507149	0.9647	0.999821	3.72E-01	0.568202	0.00636	3.08E-10	6.57E-05	39.693962
rs1214759	A	G	A	G	-0.01681	-0.012299	0.4192	0.317	4.52E-01	0.016341	0.002573	6.59E-11	7.07E-05	42.68309
rs12378991	G	A	G	A	-0.03902	0.009497	0.9411	0.9251	7.44E-01	0.029078	0.005503	1.39E-12	8.33E-05	50.277712
rs12509595	T	C	T	C	0.03557	0.010925	0.7552	0.703	5.18E-01	0.016888	0.002892	9.95E-35	0.000250497	151.27639
rs12549572	T	C	T	C	0.02422	0.064776	0.6728	0.755	2.90E-04	0.017875	0.002648	6.11E-20	0.000138545	83.658932
rs1273886	A	G	A	G	0.02013	0.009571	0.6749	0.597	5.42E-01	0.015704	0.002776	4.30E-13	8.71E-05	52.583395
rs1275979	T	C	T	C	-0.03297	0.003354	0.4719	0.401	8.23E-01	0.015002	0.002568	1.09E-37	0.000272941	164.8344
rs12766550	C	G	C	G	0.02881	-0.007046	0.9417	0.929	8.04E-01	0.028443	0.005162	2.44E-08	5.16E-05	31.14946
rs12803281	C	T	C	T	-0.01661	0.005143	0.5864	0.556	7.28E-01	0.014812	0.002465	1.66E-11	7.52E-05	45.405182
rs12940197	G	T	G	T	-0.02288	0.027564	0.4361	0.288	5.21E-01	0.042943	0.002976	1.55E-14	9.79E-05	59.107989
rs12959198	T	C	T	C	-0.02103	-0.027833	0.7725	0.791	1.39E-01	0.018835	0.002958	1.22E-12	8.37E-05	50.545466
rs12975656	G	A	G	A	0.02031	-0.052486	0.5826	0.495	1.07E-03	0.016037	0.0027	5.53E-14	9.37E-05	56.583827
rs13042148	C	T	C	T	-0.02296	0.059517	0.8834	0.845	2.44E-01	0.051053	0.004082	1.91E-08	5.24E-05	31.63718
rs13072118	C	T	C	T	0.0161	-0.009448	0.4291	0.328	5.64E-01	0.016372	0.002608	6.93E-10	6.31E-05	38.109792
rs13072590	G	A	G	A	0.01745	-0.018968	0.786	0.714	2.70E-01	0.017211	0.003135	2.69E-08	5.13E-05	30.982451
rs13333693	A	G	A	G	0.02753	0.014112	0.7554	0.764	4.16E-01	0.017367	0.00284	3.46E-22	0.000155614	93.967082
rs1336173	T	A	T	A	0.04684	0.691938	0.9711	0.999778	4.35E-01	0.887292	0.007667	1.03E-09	6.25E-05	37.323542
rs140501563	G	A	G	A	-0.08845	-0.244441	0.982	0.999742	5.50E-01	0.409294	0.01484	2.61E-09	0.000201874	35.524492
rs141935404	C	T	C	T	-0.02551	-0.054851	0.9136	0.9124	2.57E-02	0.024592	0.004305	3.21E-09	5.82E-05	35.113539
rs142365336	G	T	G	T	0.1224	1.559198	0.986	0.999945	6.40E-01	3.334395	0.01888	9.41E-11	0.000238834	42.029948
rs1427298	C	T	C	T	-0.01837	0.044043	0.6201	0.576	3.91E-03	0.015263	0.002624	2.67E-12	8.12E-05	49.010671
rs142887200	C	T	C	T	-0.06546	1.051178	0.9795	0.999957	3.63E-01	1.155785	0.008327	3.98E-15	0.000103427	61.798064
rs143341259	T	C	T	C	0.04981	0.008266	0.9743	0.9647	8.32E-01	0.039012	0.008639	8.33E-09	5.56E-05	33.243454
rs144261869	A	G	A	G	0.03665	-0.615346	0.9654	0.999633	3.18E-01	0.616828	0.006401	1.06E-08	5.49E-05	32.783272
rs144726326	C	T	C	T	-0.0398	-0.257249	0.9534	0.999536	4.83E-01	0.366685	0.005839	9.65E-12	7.78E-05	46.461069
rs145391254	C	T	C	T	0.03637	-0.035361	0.9666	0.999225	8.88E-01	0.251494	0.006427	1.57E-08	5.36E-05	32.023589
rs149474053	T	C	T	C	0.1332	1.455379	0.9887	0.999954	6.71E-01	3.426589	0.02144	5.38E-10	0.000219333	38.597474
rs149674884	G	A	G	A	0.0641	0.307132	0.9555	0.999826	6.86E-01	0.75986	0.01031	5.19E-10	0.000219656	38.654381
rs149925191	G	T	G	T	0.03863	0.224694	0.9729	0.999787	6.24E-01	0.458764	0.006972	3.08E-08	5.14E-05	30.699737
rs1502282	G	C	G	C	-0.01458	-0.000413	0.5896	0.618	9.78E-01	0.015271	0.0025	5.62E-09	5.63E-05	34.012224
rs1529897	T	G	T	G	-0.01533	-0.065026	0.5376	0.576	4.69E-06	0.014204	0.002553	1.97E-09	5.97E-05	36.056426
rs1551287	C	T	C	T	0.02749	-0.012882	0.2156	0.199	4.99E-01	0.019073	0.00299	4.04E-20	0.000139987	84.529267
rs1574817	T	G	T	G	0.01733	0.023743	0.3903	0.334	1.49E-01	0.016447	0.002612	3.35E-11	7.37E-05	44.020075
rs1609829	T	C	T	C	-0.01753	-0.01302	0.293	0.31	4.42E-01	0.016945	0.002702	9.06E-11	6.97E-05	42.091378
rs164746	G	A	G	A	-0.02005	-0.040304	0.6178	0.589	4.41E-03	0.014155	0.002597	1.22E-14	9.87E-05	59.605297
rs17038648	C	T	C	T	0.1127	1.64754	0.9767	0.999931	5.84E-01	3.009342	0.01432	3.74E-15	0.000351924	61.938659
rs17314270	T	C	T	C	0.1032	1.290281	0.9746	0.999903	6.39E-01	2.749865	0.01385	9.40E-14	0.000315474	55.521328
rs17367435	T	C	T	C	-0.01507	-0.020331	0.5555	0.441	1.87E-01	0.015398	0.002667	1.63E-08	5.29E-05	31.928644
rs17390839	G	A	G	A	0.02233	-0.022162	0.837	0.798	2.16E-01	0.017893	0.003495	1.73E-10	6.76E-05	40.820948
rs17472490	G	A	G	A	-0.04054	-0.042088	0.9654	0.999627	9.09E-01	0.366912	0.006389	2.28E-10	6.74E-05	40.26259
rs17472728	G	T	G	T	0.05347	0.111722	0.9855	0.999865	8.41E-01	0.557105	0.009379	1.22E-08	5.44E-05	32.501791
rs17472899	A	G	A	G	-0.05945	1.035562	0.9824	0.999933	3.67E-01	1.147621	0.008667	7.20E-12	7.87E-05	47.050704
rs17473032	T	C	T	C	-0.06074	1.022888	0.9824	0.999936	3.73E-01	1.149274	0.008775	4.65E-12	8.02E-05	47.913216
rs17473487	C	T	C	T	-0.06575	1.05077	0.9303	0.999957	3.63E-01	1.155618	0.008394	4.99E-15	0.000348612	61.355512
rs17473690	G	T	G	T	-0.0628	1.048549	0.9811	0.999957	3.64E-01	1.154253	0.008636	3.70E-13	8.85E-05	52.880313
rs17473844	T	C	T	C	-0.04674	-0.374471	0.9708	0.999848	5.58E-01	0.638859	0.006952	1.85E-11	7.57E-05	45.202025
rs17473985	G	A	G	A	-0.03361	-0.398863	0.9494	0.99965	2.80E-01	0.368952	0.005585	1.83E-09	6.00E-05	36.215182
rs17501084	A	G	A	G	-0.05527	1.116879	0.9818	0.99992	3.01E-01	1.080647	0.008478	7.30E-11	7.11E-05	42.500311
rs17501559	A	G	A	G	0.05322	0.095969	0.9853	0.999866	8.65E-01	0.56426	0.009367	1.37E-08	5.40E-05	32.281128
rs17502552	G	T	G	T	-0.06301	1.065529	0.9796	0.99995	3.53E-01	1.148224	0.008296	3.21E-14	9.65E-05	57.687467
rs17502782	C	A	C	A	-0.0337	-0.395649	0.9507	0.999651	2.84E-01	0.369277	0.005628	2.18E-09	5.94E-05	35.855157
rs17502922	C	G	C	G	0.06535	5.5436	0.9708	0.999803	6.68E-02	3.0243	0.0119	4.07E-08	0.000171381	30.157634
rs17502970	C	A	C	G	-0.04408	-0.42123	0.9695	0.000212	8.34E-01	2.0109	0.006699	4.86E-11	7.25E-05	43.297539
rs17502970	C	A	C	A	-0.04408	-0.177821	0.9695	0.999862	7.82E-01	0.64215	0.006699	4.86E-11	7.25E-05	43.297539
rs1757225	A	G	A	G	-0.02184	0.005772	0.3802	0.319	7.26E-01	0.016443	0.002547	1.05E-17	0.000121768	73.52709
rs185372147	T	G	T	G	-0.06575	1.053678	0.9303	0.999957	3.62E-01	1.155731	0.008394	4.99E-15	0.000348612	61.355512
rs190945315	A	C	A	C	-0.06543	1.097761	0.9302	0.99977	3.88E-01	1.272715	0.008378	6.02E-15	0.000346548	60.992036
rs191079	T	C	T	C	0.01581	-0.030384	0.5793	0.769	7.41E-02	0.017011	0.002839	2.62E-08	5.14E-05	31.012227
rs191102591	G	T	G	T	-0.04406	-0.361321	0.9695	0.999838	5.68E-01	0.632321	0.006699	4.98E-11	7.24E-05	43.258258
rs1979845	C	T	C	T	0.03115	0.002591	0.8267	0.9037	9.17E-01	0.024799	0.003306	4.62E-21	0.000147023	88.779032
rs1984669	C	T	C	T	-0.0221	0.041164	0.7592	0.77	2.68E-02	0.01859	0.002834	6.66E-15	0.000100712	60.81138
rs1989061	T	C	T	C	0.01826	0.002497	0.3219	0.384	8.70E-01	0.015205	0.002709	1.62E-11	7.52E-05	45.434268
rs199680901	C	T	C	T	-0.01837	-0.022986	0.6385	0.569	5.42E-01	0.037724	0.002631	3.01E-12	8.07E-05	48.750224
rs1997596	C	T	C	T	-0.01956	-0.03952	0.666	0.648	1.05E-02	0.015435	0.002733	8.62E-13	8.48E-05	51.222225
rs2050542	T	C	T	C	0.04403	-0.557744	0.9731	0.999796	2.95E-01	0.532803	0.007712	1.16E-08	5.46E-05	32.595926
rs210139	A	C	A	C	-0.01598	-0.032041	0.5911	0.62	2.64E-02	0.014434	0.0024	2.89E-11	7.34E-05	44.333403
rs2168785	C	T	C	T	0.01863	-0.006854	0.3675	0.236	6.76E-01	0.016393	0.002684	4.09E-12	7.98E-05	48.179322
rs2195880	C	G	C	G	0.01569	0.010874	0.7004	0.642	4.72E-01	0.01512	0.002629	2.46E-09	5.90E-05	35.617605
rs219791	T	G	T	G	0.0173	-0.021571	0.6668	0.734	2.13E-01	0.017325	0.002688	1.26E-10	6.86E-05	41.422249
rs2207404	G	A	G	A	-0.0484	0.303112	0.9636	0.999222	3.00E-01	0.292267	0.006281	1.35E-14	9.83E-05	59.379035
rs2255390	A	G	A	G	0.02748	-0.014808	0.4329	0.449	3.13E-01	0.014667	0.002426	1.05E-29	0.000212471	128.30745
rs2327429	T	C	T	C	-0.02284	0.044968	0.737	0.717	8.68E-03	0.017134	0.002788	2.65E-16	0.000111147	67.113001
rs2340534	G	A	G	A	0.01826	0.03896	0.5376	0.488	1.78E-02	0.016446	0.002458	1.15E-13	9.14E-05	55.187127
rs2497318	C	T	C	T	-0.02938	-0.042688	0.6293	0.552	2.33E-03	0.014022	0.002633	6.77E-29	0.000206183	124.50932
rs2569882	T	C	T	C	-0.01474	-0.018963	0.5253	0.579	2.39E-01	0.016088	0.002462	2.21E-09	5.94E-05	35.844198
rs2761244	T	C	T	C	0.01525	0.00136	0.3969	0.397	9.30E-01	0.015525	0.002484	8.53E-10	6.24E-05	37.6909
rs2823263	T	C	T	C	-0.02564	0.022891	0.7425	0.713	1.38E-01	0.015414	0.002846	2.17E-19	0.000134415	81.164525
rs28398484	C	G	C	G	-0.04176	-0.402199	0.9686	0.999837	5.33E-01	0.645546	0.006668	3.89E-10	6.56E-05	39.222006
rs28398495	A	G	A	G	-0.03906	-0.308813	0.9512	0.999519	3.99E-01	0.365831	0.005686	6.69E-12	7.90E-05	47.190086
rs28398500	C	G	C	G	-0.0384	-0.309182	0.9513	0.999519	3.98E-01	0.365876	0.005685	1.49E-11	7.64E-05	45.624856
rs28398507	G	A	G	A	-0.03811	-0.343103	0.9515	0.999532	3.48E-01	0.365708	0.005688	2.15E-11	7.51E-05	44.890942
rs28398535	T	C	T	C	-0.03384	-0.513556	0.9598	0.999676	2.02E-01	0.402898	0.005987	1.62E-08	5.35E-05	31.947891
rs28398544	A	G	A	G	-0.03329	-0.537365	0.9595	0.999707	1.86E-01	0.406348	0.005932	2.04E-08	5.27E-05	31.493817
rs28416181	T	G	T	G	0.02165	-0.021142	0.71	0.74	2.26E-01	0.017469	0.002647	3.04E-16	0.00011079	66.897197

rs28430881	C	A	C	A	-0.02156	0.034427	0.5611	0.541	1.90E-02	0.014677	0.002429	7.34E-19	0.000130475	78.784808
rs28446035	C	T	C	T	0.03696	-0.609662	0.9657	0.999613	3.20E-01	0.613188	0.006433	9.40E-09	5.52E-05	33.009339
rs28558845	G	C	G	C	-0.02063	0.003878	0.82	0.853	8.49E-01	0.02041	0.003066	1.76E-11	7.50E-05	45.274551
rs28570591	G	A	G	A	-0.06608	1.052892	0.9798	0.999956	3.62E-01	1.155183	0.008333	2.30E-15	0.000105244	62.883587
rs3176466	C	T	C	T	0.04031	-0.022639	0.8668	0.9182	3.73E-01	0.025422	0.003543	5.89E-30	0.000214353	129.44441
rs34070447	A	G	A	G	0.02013	0.008915	0.5474	0.585	5.67E-01	0.015583	0.00238	2.89E-17	0.000118474	71.53748
rs34136790	C	T	C	T	0.02587	0.092569	0.8161	0.9137	1.93E-01	0.071051	0.004021	1.29E-10	6.93E-05	41.392791
rs34917191	T	C	T	C	-0.01332	0.009831	0.4203	0.403	5.15E-01	0.015105	0.002409	3.32E-08	5.06E-05	30.572774
rs35444	A	G	A	G	-0.0336	0.000102	0.6131	0.623	9.95E-01	0.015408	0.00241	3.97E-44	0.000321843	194.37682
rs35619052	G	A	G	A	0.02036	0.041559	0.7815	0.722	2.34E-02	0.018338	0.003609	1.73E-08	5.33E-05	31.82598
rs35619990	C	T	C	T	-0.01762	0.036699	0.5513	0.518	9.87E-03	0.014224	0.002433	4.59E-13	8.69E-05	52.447838
rs35812759	A	G	A	G	0.01552	0.011898	0.7522	0.751	5.08E-01	0.017979	0.002755	1.79E-08	5.26E-05	31.735126
rs36092406	G	A	G	A	-0.04798	0.302794	0.9635	0.99922	3.01E-01	0.292473	0.006277	2.19E-14	9.68E-05	58.427355
rs3751767	T	C	T	C	-0.01723	0.031985	0.6902	0.667	6.02E-02	0.017022	0.002891	2.59E-09	5.88E-05	35.520116
rs3790604	C	A	C	A	0.04697	-0.057572	0.9322	0.905	2.95E-02	0.026456	0.004789	1.12E-22	0.000159302	96.194767
rs3802177	G	A	G	A	0.01547	-0.024586	0.7385	0.673	1.07E-01	0.015264	0.00278	2.70E-08	5.13E-05	30.966423
rs3848132	T	A	T	A	-0.03504	-0.008943	0.7463	0.707	5.82E-01	0.016238	0.002922	4.26E-33	0.000238125	143.80294
rs4114858	A	G	A	G	0.01947	-0.002551	0.8371	0.9256	9.27E-01	0.027698	0.003459	1.87E-08	5.25E-05	31.683327
rs4413681	G	C	G	C	0.06552	5.5404	0.9709	0.999793	5.66E-02	2.9062	0.01196	4.44E-08	0.00017055	30.011342
rs4461961	C	T	C	T	0.01818	-0.024627	0.6471	0.739	1.42E-01	0.016771	0.002611	3.43E-12	8.03E-05	48.481273
rs4485922	A	G	A	G	-0.01442	0.026259	0.46	0.498	8.69E-02	0.015339	0.002442	3.56E-09	5.78E-05	34.868977
rs4677143	A	G	A	G	0.01596	0.029558	0.6379	0.59	5.82E-02	0.015604	0.002609	9.70E-10	6.20E-05	37.421192
rs4737371	G	A	G	A	-0.02244	-0.029858	0.8207	0.813	1.24E-01	0.019387	0.003276	7.74E-12	7.77E-05	46.919991
rs4745804	T	C	T	C	-0.01661	0.009493	0.5313	0.514	5.38E-01	0.01541	0.002577	1.20E-10	6.88E-05	41.544203
rs4766578	T	A	T	A	0.01805	0.22217	0.3993	0.482	1.08E-55	0.014132	0.002668	1.39E-11	7.58E-05	45.770195
rs4809849	C	T	C	T	0.01779	0.016921	0.4172	0.519	2.73E-01	0.01545	0.002675	3.03E-11	7.33E-05	44.228715
rs4846476	G	C	G	C	0.01665	-0.011377	0.7784	0.761	5.32E-01	0.018205	0.002851	5.38E-09	5.65E-05	34.106255
rs4848713	C	T	C	T	0.02442	-0.009005	0.8902	0.874	7.15E-01	0.024664	0.004187	5.65E-09	5.63E-05	34.016169
rs4867732	C	G	C	G	0.02908	-0.036584	0.9196	0.9391	2.09E-01	0.029107	0.004584	2.30E-10	6.67E-05	40.243856
rs4924538	A	T	A	T	-0.01606	-0.084761	0.5128	0.527	2.39E-02	0.037535	0.002619	8.81E-10	6.23E-05	37.602791
rs4936409	A	G	A	G	-0.01693	0.021548	0.491	0.549	1.52E-01	0.01503	0.002558	3.75E-11	7.25E-05	43.803906
rs547340372	G	A	G	A	-0.05554	1.11639	0.976	0.99995	3.39E-01	1.167146	0.007827	1.33E-12	8.43E-05	50.352505
rs55650455	T	C	T	C	-0.01947	-0.033615	0.8204	0.771	5.25E-02	0.017336	0.003288	3.26E-09	5.81E-05	35.064551
rs56218647	C	A	C	A	0.02388	0.025703	0.9123	0.9103	3.49E-01	0.027442	0.004155	9.36E-09	5.47E-05	33.03137
rs56237637	T	A	T	A	0.04136	0.500441	0.9645	0.999743	5.35E-01	0.805878	0.007107	6.08E-09	5.67E-05	33.867913
rs56329049	G	A	G	A	-0.04065	-0.330651	0.9643	0.999506	3.19E-01	0.331884	0.006417	2.45E-10	6.65E-05	40.128878
rs56376587	A	C	A	C	-0.02835	0.018018	0.5817	0.525	2.68E-01	0.016267	0.002564	2.19E-28	0.000202452	122.25597
rs56790613	A	G	A	G	0.0377	-0.29422	0.9673	0.999742	7.18E-01	0.815748	0.006576	1.01E-08	5.50E-05	32.866926
rs569550	T	G	T	G	0.03941	-0.014918	0.6382	0.608	3.46E-01	0.015834	0.002628	8.92E-51	0.000372341	224.88586
rs57420239	C	T	C	T	-0.03706	-0.428169	0.9678	0.999744	3.34E-01	0.443456	0.006577	1.80E-08	5.31E-05	31.750835
rs57484993	A	G	A	G	0.03418	-0.03356	0.9219	0.9105	2.08E-01	0.026651	0.004607	1.22E-13	9.12E-05	55.043709
rs576943137	C	T	C	T	-0.02548	-0.115813	0.8922	0.857	3.90E-02	0.056109	0.004521	1.79E-08	5.26E-05	31.763608
rs578097	A	G	A	G	-0.02281	-0.000639	0.652	0.672	9.69E-01	0.016402	0.002544	3.25E-19	0.000133137	80.392652
rs57850577	A	G	A	G	-0.05408	0.260481	0.9691	0.999236	3.80E-01	0.296608	0.006479	7.43E-17	0.000116604	69.67186
rs57944734	C	T	C	T	-0.04826	0.380457	0.9619	0.999126	1.78E-01	0.282387	0.006162	5.02E-15	0.000101584	61.338231
rs58320941	G	T	G	T	-0.04411	-0.303404	0.9695	0.999839	6.31E-01	0.631779	0.006735	5.96E-11	7.18E-05	42.894234
rs59482369	T	C	T	C	-0.04242	0.144026	0.959	0.999091	5.87E-01	0.26501	0.006235	1.05E-11	7.75E-05	46.288
rs60085321	C	G	C	G	-0.05059	0.226761	0.9688	0.999166	4.48E-01	0.298678	0.006686	4.02E-14	9.48E-05	57.252785
rs6013199	G	A	G	A	-0.07916	4.444034	0.9722	0.999968	5.15E-01	6.824339	0.01317	1.89E-09	0.000205301	36.127676
rs6031431	A	G	A	G	0.01961	-0.042742	0.5497	0.532	2.96E-03	0.014385	0.002464	1.81E-15	0.000104898	63.339376
rs60772526	C	T	C	T	0.0605	0.072534	0.127	0.077	1.00E-02	0.028158	0.003746	1.27E-58	0.000431845	260.84061
rs6108787	T	G	T	G	0.01367	0.015494	0.5405	0.532	3.10E-01	0.015255	0.002403	1.31E-08	5.36E-05	32.361563
rs61169316	G	A	G	A	0.01814	0.029385	0.7215	0.662	7.28E-02	0.016376	0.002753	4.56E-11	7.19E-05	43.417233
rs61793790	G	C	G	C	-0.03709	0.009862	0.9597	0.939	7.72E-01	0.034035	0.006649	2.49E-08	5.21E-05	31.117235
rs61897793	G	A	G	A	-0.02178	0.073866	0.8642	0.83	1.11E-04	0.019111	0.003653	2.56E-09	5.95E-05	35.548101
rs62193645	C	T	C	T	0.03441	0.022314	0.8796	0.863	3.14E-01	0.022181	0.003785	1.04E-19	0.000136873	82.648995
rs62330329	T	C	T	C	0.02703	-0.038953	0.2697	0.303	2.58E-01	0.034466	0.004425	1.04E-09	6.18E-05	37.313462
rs62374068	A	G	A	G	0.05943	-0.005083	0.7821	0.771	7.86E-01	0.018712	0.002918	3.67E-92	0.000686566	414.80204
rs62435145	G	T	G	T	-0.0256	0.019266	0.434	0.319	2.75E-01	0.017637	0.0027	2.68E-21	0.000148877	89.898491
rs66482211	A	G	A	G	-0.04204	0.008984	0.9505	0.9507	7.91E-01	0.033895	0.005675	1.34E-13	9.09E-05	54.87742
rs66561220	T	C	T	C	-0.02949	0.003691	0.7728	0.714	8.44E-01	0.018776	0.003016	1.49E-22	0.000158328	95.606379
rs66561220	T	C	C	A	-0.02949	-1.177719	0.7728	3.90E-05	7.07E-01	3.132004	0.003016	1.49E-22	0.000158328	95.606379
rs6697367	C	T	C	T	-0.01868	-0.01536	0.6768	0.665	3.74E-01	0.017269	0.002575	4.19E-13	8.72E-05	52.625869
rs6703881	T	C	T	C	-0.02296	0.023171	0.1968	0.156	2.42E-01	0.019808	0.00312	1.91E-13	8.97E-05	54.154504
rs6716091	C	T	C	T	0.02352	0.013218	0.7446	0.721	4.23E-01	0.016492	0.002796	4.19E-17	0.00011719	70.762033
rs6755070	G	T	G	T	0.01832	-0.024278	0.6877	0.65	5.34E-01	0.039054	0.002757	3.10E-11	7.31E-05	44.154748
rs6794202	C	T	C	T	0.02384	0.028524	0.7896	0.753	9.68E-02	0.017177	0.003025	3.39E-15	0.000102862	62.110031
rs6816915	G	A	G	A	-0.01441	-0.01351	0.4714	0.528	3.77E-01	0.015281	0.002594	2.83E-08	5.11E-05	30.859438
rs6826742	G	T	G	T	-0.01667	0.021965	0.4695	0.442	1.38E-01	0.014826	0.00243	7.13E-12	7.79E-05	47.060729
rs6877631	T	C	T	C	-0.0154	0.007554	0.6149	0.542	6.23E-01	0.015388	0.002594	2.99E-09	5.84E-05	35.245323
rs690054	C	T	C	T	-0.03594	-0.006995	0.5832	0.545	6.38E-01	0.014867	0.002501	8.68E-47	0.000341917	206.50414
rs6934923	G	T	G	T	-0.05549	0.303182	0.9748	0.99921	3.16E-01	0.302257	0.007262	2.25E-14	9.77E-05	58.387107
rs6934923	G	T	T	C	-0.05549	-0.095817	0.9748	0.00453	5.37E-01	0.155382	0.007262	2.25E-14	9.77E-05	58.387107
rs6941546	G	A	G	A	-0.02568	-0.054744	0.9129	0.9429	9.59E-02	0.032877	0.004349	3.63E-09	5.77E-05	34.86673
rs6961048	C	G	C	G	0.04642	-0.001179	0.8895	0.892	9.60E-01	0.023317	0.003761	5.76E-35	0.000252252	152.33637
rs697976	T	C	T	C	0.01364	0.034177	0.4779	0.461	1.44E-02	0.01397	0.0024	1.34E-08	5.35E-05	32.300278
rs7038668	T	C	T	C	-0.0279	0.01669	0.5014	0.535	2.73E-01	0.015237	0.002425	1.38E-30	0.000219195	132.36858
rs71537958	G	C	G	C	-0.01936	-0.005195	0.7389	0.676	7.55E-01	0.016627	0.002891	2.22E-11	7.43E-05	44.845051
rs716877	C	G	C	G	0.03596	0.006428	0.5162	0.6	6.85E-01	0.015847	0.002627	1.35E-42	0.000310259	187.37827
rs7187932	G	A	G	A	-0.0223	-0.062306	0.7746	0.812	1.02E-03	0.018963	0.002875	9.15E-15	9.96E-05	60.163629
rs7218857	T	C	T	C	-0.02113	0.023863	0.5618	0.587	1.35E-01	0.015985	0.002471	1.30E-17	0.000121099	73.122916
rs7222451	G	A	G	A	-0.01388	-0.001449	0.4975	0.547	9.22E-01	0.014871	0.002471	1.99E-08	5.23E-05	31.552476
rs72969822	C	G	C	G	-0.0249	0.047579	0.8821	0.869	3.36E-02	0.022386	0.003796	5.55E-11	7.13E-05	43.027517
rs73776711	G	A	G	A	-0.0479	0.312509	0.9631	0.999164	2.81E-01	0.290158	0.006173	8.96E-15	9.97E-05	60.211365
rs7416991	T	C	C	A	0.01931	-0.0366	0.4025	0.302	4.24E-01	0.0458	0.002593	1.01E-13	9.18E-05	55.457401
rs7416991	T	G	T	G	-0.01795	0.0366	0.6404	0.698	4.24E-01	0.0458	0.002504	7.97E-13	8.51E-05	51.387827
rs7416991	T	C	T	C	0.01931	-0.0048	0.4025	0.513	9.23E-01	0.0498	0.002593	1.01E-13	9.18E-05	55.457401
rs7416991	T	G	G	A	-0.01795	0.0048	0.6404	0.487	9.23E-01	0.0498	0.002504	7.97E-13	8.51E-05	51.387827
rs74496669	G	T	G	T	0.03972	-0.710228	0.9698	0.999648	2.62E-01	0.6327	0.00716	2.96E-08	5.15E-05	30.774601
rs74518739	C	T	C	T	-0.04059	-0.330209	0.9527	0.999644	4.19E-01	0.408273	0.005761	1.91E-12	8.31E-05	49.641209
rs75248620	G	A	G	A	0.03605	-0.065729	0.9368	0.9177	1.76E-02	0.027678	0.00518	3.55E-12	8.02E-05	48.434076
rs75411357	A	T	A	T	0.03822	-0.578287	0.9656	0.999614	3.44E-01	0.611637	0.006723	1.35E-08	5.41E-05	32.318794
rs75741381	C	G	C	G	-0.01862	0.012713	0.7999	0.85	5.56E-01	0.021608	0.003124	2.60E-09	5.88E-05	35.525263
rs76075621	T	C	T	C	0.03689	-0.606308	0.9658	0.999628	3.24E-01	0.61415	0.006443	1.05E-08	5.49E-05	32.782423
rs761356	T	C	T	C	0.02838	-0.031343	0.6163	0.549	2.56E-02	0.014043	0.002531	3.76E-29	0.000208204	125.73046
rs76200870	G	A	G	A	-0.05573	0.221822	0.9737	0.999181	4.59E-01	0.29941	0.007021	2.17E-15	0.000105448	63.005743
rs76666870	T	C	T	C	0.03689	-0.589342	0.9658	0.999618	3.36E-01	0.612751	0.006443	1.05E-08	5.49E-05	32.782423
rs76821958	A	G	A	G	-0.04048	-0.268483	0.9734	0.999344	3.67E-01	0.297425	0.006957	6.08E-09	5.61E-05	33.856106
rs76987554	C	T	C	T	-0.07778	0.264681	0.982	0.999348	4.28E-01	0.334007	0.009027	7.31E-18	0.000124251	74.241885
rs77081469	C	A	C	A	0.06558	5.5658	0.9709	0.999802	6.56E-02	3.0225	0.01191	3.76E-08	0.0001723	30.319309
rs7726795	T	C	T	C	0.02854	0.015464	0.6314	0.701	3.28E-01	0.015816	0.002657	7.04E-27	0.000191065	115.37848
rs77290217	T	C	T	C	-0.04754	0.126465	0.9649	0.999112	6.41E-01	0.270844	0.006553	4.18E-13	8.81E-05	52.630563
rs7749375	G	A	G	A	-0.05485	0.235417	0.975	0.999192	4.37E-01	0.302754	0.007327	7.38E-14	9.38E-05	56.040355
rs7766997	A	G	A	G	-0.05467	0.208451	0.9749	0.999183	4.88E-01	0.300433	0.007245	4.68E-14	9.53E-05	56.940512
rs7767054	G	A	G	A	-0.05415	0.212611	0.9743	0.999184	4.79E-01	0.300661	0.00719	5.22E-14	9.49E-05	56.720381
rs780101	C	T	C	T	0.02158	-0.005504	0.5339	0.585	7.23E-01	0.015537	0.002504	7.30E-18	0.000123004	74.273558
rs78132593	C	A	C	A	0.01948	-0.012699	0.8381	0.789	4.81E-01	0.018013	0.003462	1.87E-08	5.24E-05	31.660938
rs7836572	T	C	T	C	0.02144	-0.018418	0.4105	0.459	2.32E-01	0.015421	0.002459	2.91E-18	0.000125898	76.020812
rs78716205	G	A	G	A	0.0679	0.036083	0.9669	0.9543	3.28E-01	0.036926	0.006869	5.19E-23	0.000161816	97.713038
rs79030490	C	A	C	A	-0.07534	0.214775	0.9817	0.99934	5.38E-01	0.348366	0.008966	4.59E-17	0.00011817	70.607976
rs79392591	C	G	C	G	-0.0245	0.001025	0.8683	0.857	9.60E-01	0.020491	0.003564	6.53E-12	7.83E-05	47.25598
rs79557018	C	T	C	T	-0.05487	0.216729	0.9749	0.999183	4.71E-01	0.300417	0.007246	3.81E-14	9.60E-05	57.342056
rs79590186	C	T	C	T	-0.07237	0.387128	0.976	0.999349	2.45E-01	0.333262	0.007995	1.49E-19	0.000137128	81.937028

rs79889868	G	C	G	C	-0.05507	0.261425	0.9696	0.999237	3.77E-01	0.29617	0.00659	6.80E-17	0.000116873	69.832779
rs80176668	A	G	A	G	-0.07368	0.384346	0.9774	0.999364	2.49E-01	0.333553	0.007748	2.05E-21	0.000151342	90.431557
rs80289662	C	G	C	G	-0.05427	0.262017	0.9694	0.999234	3.77E-01	0.296306	0.006589	1.86E-16	0.000113537	67.839186
rs80328223	C	T	C	T	-0.07624	0.294819	0.9784	0.99933	3.74E-01	0.331437	0.008251	2.62E-20	0.000142888	85.379302
rs8077544	G	A	G	A	0.01629	-0.009034	0.3428	0.207	6.12E-01	0.017819	0.00285	1.13E-08	5.41E-05	32.670249
rs8125560	A	C	A	C	-0.01906	-0.020262	0.825	0.819	3.07E-01	0.019825	0.003137	1.28E-09	6.11E-05	36.91618
rs820429	G	T	G	T	-0.04348	0.008942	0.6755	0.602	5.53E-01	0.015058	0.002787	7.92E-55	0.000402968	243.39135
rs880315	T	C	T	C	0.04161	-0.032978	0.6739	0.643	4.79E-02	0.016673	0.002572	7.72E-59	0.000433317	261.73
rs923000	A	C	A	C	0.02374	0.013052	0.637	0.564	3.59E-01	0.014216	0.002662	4.99E-19	0.000131713	79.532627
rs9321413	C	G	C	G	0.03641	-0.60418	0.9653	0.999629	3.26E-01	0.615078	0.006408	1.36E-08	5.40E-05	32.28467
rs9321414	A	C	A	C	0.03723	-0.59854	0.9642	0.999614	2.79E-01	0.552782	0.006659	2.30E-08	5.23E-05	31.258493
rs9483608	A	G	A	G	0.03662	-0.636877	0.9657	0.99964	3.02E-01	0.617286	0.006344	7.99E-09	5.58E-05	33.32041
rs9483610	G	A	G	A	0.03663	-0.617647	0.9657	0.999616	3.15E-01	0.614085	0.00635	8.18E-09	5.57E-05	33.275638
rs9483614	C	T	C	T	0.03847	-0.60641	0.9663	0.999626	3.21E-01	0.611153	0.006761	1.30E-08	5.42E-05	32.375949
rs9493708	C	T	C	T	0.03629	-0.62754	0.9655	0.999628	3.07E-01	0.613835	0.006343	1.09E-08	5.48E-05	32.732902
rs9493713	C	A	C	A	0.03717	-0.635242	0.9659	0.999637	3.01E-01	0.614706	0.006432	7.72E-09	5.59E-05	33.395892
rs9493714	C	T	C	T	0.03664	-0.63399	0.9646	0.999631	2.99E-01	0.61002	0.006371	9.05E-09	5.54E-05	33.074685
rs9493720	T	C	T	C	0.03665	-0.603077	0.9654	0.999621	3.27E-01	0.615357	0.006401	1.06E-08	5.49E-05	32.783272
rs9493722	T	C	T	C	0.03711	-0.60926	0.9659	0.99962	3.20E-01	0.612491	0.006433	8.22E-09	5.57E-05	33.277815
rs9493723	T	C	T	C	0.03665	-0.602347	0.9654	0.99962	3.26E-01	0.612657	0.006401	1.06E-08	5.49E-05	32.783272
rs9493727	A	G	A	G	0.03721	-0.60068	0.9641	0.999619	2.78E-01	0.553226	0.006656	2.32E-08	5.23E-05	31.253072
rs9493728	A	G	A	G	0.03714	-0.598857	0.9642	0.999618	2.79E-01	0.553056	0.006656	2.48E-08	5.21E-05	31.135595
rs9493729	G	T	G	T	0.03978	-0.300025	0.9699	0.999749	7.20E-01	0.838434	0.007166	2.92E-08	5.16E-05	30.815978
rs9603367	C	T	C	T	0.05097	-0.025664	0.609	0.519	9.46E-02	0.015351	0.002692	7.22E-80	0.000593419	358.49174
rs963837	T	C	T	C	0.0215	-0.007339	0.6211	0.54	6.35E-01	0.015439	0.002687	1.29E-15	0.000106032	64.023821
rs9819344	C	T	C	T	0.03573	0.133391	0.9518	0.99941	6.46E-01	0.290594	0.005812	8.11E-10	6.33E-05	37.793301
rs9821489	G	A	G	A	0.04282	-0.099268	0.8886	0.99511	3.88E-01	0.115037	0.004507	2.20E-21	0.000149484	90.264755
rs9822474	A	G	A	G	-0.01603	0.023022	0.5519	0.537	1.19E-01	0.014762	0.00239	2.06E-11	7.45E-05	44.985364
rs9844949	A	G	A	G	0.01972	0.033604	0.152	0.129	1.39E-01	0.022713	0.003549	2.83E-08	5.11E-05	30.874634
rs9918487	G	A	G	A	-0.05331	0.27275	0.9685	0.999183	3.53E-01	0.293901	0.006354	5.13E-17	0.000117809	70.391942
rs9928653	T	C	T	C	-0.01848	-0.030129	0.7631	0.834	1.08E-01	0.018722	0.002844	8.46E-11	6.99E-05	42.222578

Iron

SNP	effect_allele.	other_allele.	effect_allele.o	other_allele.	beta.exposure	beta.outcome	eaf.exposure	eaf.outcome	pval.outcome	se.outcome	se.exposure	pval.exposure	R2	Fstat
rs10831924	T	C	T	C	0.06204	0.033851	0.9131	0.99482	7.35E-01	0.099933	0.007982	8.06E-15	0.000473618	60.411571
rs12328766	A	G	A	G	0.03348	0.01269	0.6129	0.629	3.84E-01	0.014584	0.004341	1.29E-14	0.00046634	59.482789
rs13007705	C	T	C	T	-0.02771	0.018227	0.544	0.571	2.44E-01	0.015634	0.004194	4.05E-11	0.00034228	43.653211
rs13081052	T	C	T	C	0.03643	-0.028937	0.248	0.152	1.82E-01	0.021682	0.005395	1.51E-11	0.000357515	45.596913
rs140393761	A	G	A	G	-0.123	0.091808	0.9874	0.9844	1.07E-01	0.056931	0.0201	9.56E-10	0.000296251	37.447093
rs150375987	C	G	C	G	-0.1303	-0.109083	0.9792	0.9759	2.10E-02	0.047262	0.01552	4.96E-17	0.000557485	70.486411
rs1800562	G	A	G	A	-0.3257	-0.043912	0.9509	0.9318	1.31E-01	0.029106	0.01006	1.05E-229	0.008226626	1048.1889
rs190399027	G	A	G	A	-0.1314	0.088088	0.9894	0.9877	1.84E-01	0.066332	0.02111	4.98E-10	0.000306514	38.744875
rs1958078	A	C	A	C	-0.03145	0.033527	0.2447	0.139	1.24E-01	0.021785	0.005338	3.94E-09	0.000272195	34.712362
rs218248	G	A	G	A	-0.03636	0.076611	0.8382	0.869	5.73E-02	0.040306	0.00575	2.64E-10	0.000313538	39.986377
rs2294915	C	T	C	T	-0.03195	-0.003458	0.7616	0.761	8.45E-01	0.017679	0.005059	2.78E-10	0.000312745	39.885254
rs28929474	C	T	C	T	-0.1763	0.076894	0.9866	0.9798	1.37E-01	0.051672	0.0188	7.11E-21	0.000695435	87.940499
rs5995385	T	C	T	C	0.02734	-0.000643	0.5347	0.464	9.63E-01	0.014042	0.004294	1.98E-10	0.000317869	40.538991
rs72839066	A	G	A	G	-0.1163	0.011712	0.9694	0.9625	7.54E-01	0.037426	0.01327	1.99E-18	0.000607468	76.809968
rs7385804	C	A	C	A	-0.05785	0.000256	0.357	0.393	9.86E-01	0.014542	0.004417	3.75E-39	0.001343636	171.53468
rs7775698	C	T	C	T	-0.0471	-0.028548	0.7576	0.763	4.92E-01	0.04155	0.004874	4.56E-22	0.000731925	93.383633
rs806970	C	T	C	T	0.05669	-0.121287	0.945	0.9251	8.43E-06	0.027231	0.009471	2.22E-09	0.000283444	35.827891
rs8177252	C	A	C	A	-0.05473	-0.012705	0.6848	0.668	4.34E-01	0.016225	0.00463	3.25E-32	0.00109478	139.72976
rs855791	A	G	A	G	-0.1527	0.011072	0.3768	0.42	4.46E-01	0.01452	0.004498	2.09E-252	0.008958692	1152.4953
rs9402686	G	A	G	A	-0.05435	0.027144	0.7844	0.713	8.28E-02	0.015645	0.005267	6.25E-25	0.000834495	106.48108

Zinc

SNP	effect_allele.	other_allele.	effect_allele.o	other_allele.	beta.exposure	beta.outcome	eaf.exposure	eaf.outcome	pval.outcome	se.outcome	se.exposure	pval.exposure	R2	Fstat
rs10484100	G	A	G	A	-0.209	-0.003928	0.5	0.0909	0.881	0.026255	0.045	3.30E-06	0.008225083	21.570864
rs11763353	G	A	G	A	-0.192	0.020651	0.5	0.151	0.343	0.021778	0.039	7.00E-07	0.009232191	24.236686
rs1532423	A	G	A	G	0.178	-0.029378	0.5	0.409	0.059	0.015557	0.026	6.40E-12	0.017700954	46.869822
rs2120019	C	T	C	T	-0.287	0.023466	0.5	0.193	0.205	0.018524	0.033	1.55E-18	0.028258323	75.637282
rs4333127	A	G	A	G	0.218	0.011616	0.5	0.901	0.65	0.025603	0.047	3.00E-06	0.008203506	21.513807
rs7148590	A	G	A	G	-0.14	0.020807	0.5	0.527	0.143	0.014207	0.026	1.40E-07	0.011024391	28.994083

Copper

SNP	effect_allele.	other_allele.	effect_allele.o	other_allele.	beta.exposure	beta.outcome	eaf.exposure	eaf.outcome	pval.outcome	se.outcome	se.exposure	pval.exposure	R2	Fstat
rs10014072	G	A	G	A	-0.164	0.01419	0.5	0.695	0.398	0.016785	0.034	1.00E-06	0.008865882	23.266436
rs1175550	A	G	A	G	-0.198	0.036925	0.5	0.759	0.0476	0.018638	0.032	5.00E-10	0.014505881	38.285156
rs12153606	T	G	T	G	-0.159	-0.011927	0.5	0.187	0.539	0.01942	0.034	2.00E-06	0.008337959	21.869377
rs12582659	C	T	C	T	1.262	0.006587	0.5	0.0194	0.92	0.065294	0.27	3.00E-06	0.008329487	21.846968
rs2769264	G	T	G	T	0.313	-0.05423	0.5	0.181	0.00682	0.020046	0.034	3.00E-20	0.031554808	84.74827
rs3857536	T	C	T	C	-0.129	-0.019259	0.5	0.545	0.21	0.015358	0.028	4.00E-06	0.008094561	21.225765

Selenium

SNP	effect_allele.	other_allele.	effect_allele.o	other_allele.	beta.exposure	beta.outcome	eaf.exposure	eaf.outcome	pval.outcome	se.outcome	se.exposure	pval.exposure	R2	Fstat
rs7700970	T	C	T	C	0.265	0.007729	0.5	0.292	0.648	0.016935	0.035	7.17E-13	0.010362102	57.326531

Carotene

SNP	effect_allele.	other_allele.	effect_allele.o	other_allele.	beta.exposure	beta.outcome	eaf.exposure	eaf.outcome	pval.outcome	se.outcome	se.exposure	pval.exposure	R2	Fstat
rs10846742	A	G	A	G	-0.143552	0.023952	0.832123	0.846	2.69E-01	0.02167	0.0208154	5.33E-12	6.85E-05	47.560781
rs9708919	T	C	T	C	0.246529	-0.002407	0.480938	0.454	8.72E-01	0.014948	0.0153225	3.03E-58	0.000372521	258.86705

Folate

SNP	effect_allele.	other_allele.	effect_allele.o	other_allele.	beta.exposure	beta.outcome	eaf.exposure	eaf.outcome	pval.outcome	se.outcome	se.exposure	pval.exposure	R2	Fstat
rs1801133	G	A	G	A	0.096	-0.044565	0.668	0.687	0.00345	0.01524	0.01077	1.00E-28	0.003647073	79.453139
rs652197	C	T	C	T	0.069	0.010024	0.179	0.149	0.633	0.020976	0.01109	2.50E-10	0.001780252	38.711062

Table S5 : Harmonization results for exposure SNPs and Verma et al. (multi-ancestry) T1D outcome

Outcome: Verma, A (T1D)
Multi-ancestry

Vitamin C

SNP	effect_allel	other_allel	effect_allel	other_allel	beta.exposure	beta.outcome	eaf.exposur	eaf.outcom	pval.outcome	se.outcome	se.exposure	pval.exposure	R2	Fstat
rs10051765	T	C	T	C	-0.039	0.0009995	0.6585	0.6373	0.8942	0.01086454	0.0066	3.64E-09	0.0006708	34.91736
rs10136000	A	G	A	G	0.0404	-0.0099503	0.2825	0.2934	0.4121	0.01225408	0.0071	1.33E-08	0.0006221	32.3777
rs10758628	A	C	A	C	0.0304	-0.001998	0.4567	0.4326	0.8338	0.01067423	0.0063	1.28E-06	0.0004474	23.28445
rs10995578	C	G	C	G	0.0296	-0.003992	0.4784	0.4131	0.6959	0.01124643	0.0062	1.91E-06	0.000438	22.79292
rs11062357	T	C	T	C	0.0305	-0.0007002	0.5118	0.6207	0.9527	0.01192168	0.0063	1.46E-06	0.0004504	23.43789
rs11641245	C	G	C	G	0.042	-0.0089398	0.848	0.7796	0.4957	0.01297628	0.0086	1.13E-06	0.0004583	23.85073
rs11641245	C	G	C	T	0.042	-0.0512933	0.848	0.9826	0.4596	0.06607679	0.0086	1.13E-06	0.0004583	23.85073
rs1165189	A	C	A	C	-0.0378	0.0029955	0.7469	0.7476	0.8224	0.01205765	0.0071	1.18E-07	0.0005446	28.34438
rs117885456	A	G	A	G	0.0781	-0.0421012	0.0865	0.0598	0.09611	0.02638265	0.0116	1.70E-11	0.0008707	45.33004
rs12610033	A	T	A	T	-0.0336	0.0039076	0.2831	0.2479	0.7902	0.01479668	0.0073	4.86E-06	0.0004071	21.18521
rs13028225	T	C	T	C	0.1016	0.0069756	0.8569	0.8675	0.6441	0.01556939	0.0089	2.38E-30	0.0024991	130.3189
rs174547	T	C	T	C	-0.0364	0.003992	0.6721	0.7001	0.7204	0.01203342	0.0066	3.84E-08	0.0005844	30.4169
rs185137552	T	C	T	C	0.2368	0.0962189	0.988	0.9981	0.5927	0.20136352	0.0392	1.58E-09	0.0007325	36.49146
rs2366388	A	G	A	G	0.0309	0.0069756	0.4908	0.5718	0.5618	0.01152857	0.0063	8.50E-07	0.0004623	24.05669
rs2559850	A	G	A	G	0.0583	-0.0009995	0.5979	0.5534	0.9227	0.01072526	0.0064	6.30E-20	0.0015928	82.98071
rs2941484	T	C	T	C	0.0341	0.0059175	0.4514	0.4813	0.5808	0.01067755	0.0063	6.37E-08	0.0005629	29.2973
rs33972313	T	C	T	C	-0.3601	-0.0139029	0.0319	0.0386	0.574	0.02588061	0.0179	4.61E-90	0.0077204	404.7065
rs339969	A	C	A	C	-0.0302	0.0091417	0.6068	0.6342	0.4364	0.01169362	0.0063	1.86E-06	0.0004416	22.97909
rs4867910	T	C	T	C	-0.0314	-0.0196926	0.657	0.568	0.08169	0.01107551	0.0068	3.45E-06	0.0004098	21.32266
rs542903	T	C	T	C	-0.0418	0.0246926	0.1429	0.1919	0.0707	0.0139977	0.0088	2.27E-06	0.0004336	22.5625
rs56738967	C	G	C	G	0.041	-0.0009995	0.321	0.2991	0.9316	0.01139133	0.0067	7.62E-10	0.0007194	37.44709
rs6482188	A	C	A	C	-0.0347	0.0202027	0.3086	0.3315	0.05855	0.01047321	0.0068	2.83E-07	0.0005004	26.04001
rs6693447	T	G	T	G	0.0393	-0.0079314	0.5509	0.586	0.4662	0.01075485	0.0064	6.25E-10	0.0007244	37.70728
rs676317	T	C	T	C	-0.0366	0.0079682	0.7264	0.743	0.5279	0.0122352	0.0074	7.33E-07	0.0004701	24.46238
rs73035571	A	G	A	G	0.035	-0.0027037	0.7863	0.8275	0.8555	0.01471862	0.0076	4.14E-06	0.0004076	21.20845
rs73850547	A	G	A	G	0.0333	0.0148886	0.7381	0.7706	0.2742	0.01363648	0.0071	2.85E-06	0.0004227	21.99742
rs7640441	A	C	A	C	0.0358	0.0252152	0.2383	0.2239	0.04004	0.0119824	0.0074	1.19E-06	0.0004497	23.40467
rs7740812	A	G	C	A	-0.0384	0.0089597	0.4057	0.9666	0.7838	0.03448546	0.0064	1.88E-09	0.0006916	36
rs7740812	A	G	A	G	-0.0384	-0.0069756	0.4057	0.2999	0.6297	0.0135426	0.0064	1.88E-09	0.0006916	36
rs78575870	T	C	T	C	0.0551	0.0309748	0.077	0.0578	0.1984	0.02339362	0.0117	2.46E-06	0.0004262	22.17846
rs79234109	A	G	A	G	0.0453	-0.0129162	0.1617	0.142	0.3918	0.01532959	0.0084	7.70E-08	0.0005588	29.08291
rs80246741	A	G	A	G	0.1511	-0.1284017	0.9897	0.9922	0.03021	0.05220969	0.0319	2.21E-06	0.0004311	22.43611
rs868822	T	G	T	G	-0.0329	-0.0303561	0.2648	0.3665	0.01146	0.01165663	0.0071	3.30E-06	0.0004126	21.47213
rs9895661	T	C	T	C	0.0625	0.0114655	0.817	0.7364	0.3359	0.01176582	0.0081	1.05E-14	0.0011433	59.53742
rs9915323	A	T	A	T	0.0321	0.0305292	0.2967	0.3822	0.003777	0.01085204	0.0068	2.60E-06	0.0004282	22.28395

Vitamin D

SNP	effect_allel	other_allel	effect_allel	other_allel	beta.exposure	beta.outcome	eaf.exposur	eaf.outcom	pval.outcome	se.outcome	se.exposure	pval.exposure	R2	Fstat
rs10741657	A	G	A	G	0.0308	-0.0083346	0.5	0.3648	0.4347	0.01059796	0.0022	2.05E-46	0.0028545	196
rs10745742	T	C	T	C	0.0165	-0.003992	0.5	0.4501	0.7373	0.01111276	0.0022	1.88E-14	0.0008126	56.25
rs10888491	A	G	A	G	0.012	0.0079682	0.5	0.4566	0.458	0.01123214	0.0022	8.62E-08	0.0004292	29.75207
rs11195965	A	T	A	T	-0.0157	0.0148886	0.5	0.8319	0.2751	0.0141926	0.0032	6.31E-07	0.0003396	24.07129
rs11203339	T	C	T	C	-0.0104	-0.0009995	0.5	0.3722	0.9582	0.01087245	0.0021	8.56E-07	0.0003143	24.52608
rs12507653	A	T	A	T	0.0118	0.0111621	0.5	0.583	0.347	0.0117574	0.0025	2.21E-06	0.0003538	22.2784
rs12785878	T	G	T	G	0.0363	-0.0099503	0.5	0.6298	0.4147	0.01177347	0.0022	3.81E-62	0.0034638	272.25
rs17082722	T	C	T	C	-0.0703	-0.0018016	0.5	0.9853	0.9674	0.04410255	0.0149	2.22E-06	0.0006398	22.26066
rs17216707	T	C	T	C	0.0263	-0.028914	0.5	0.8248	0.0434	0.01391327	0.0027	8.14E-23	0.0013256	94.88203
rs1809851	A	T	A	T	0.0104	-0.0099503	0.5	0.3994	0.3341	0.01065179	0.0023	4.86E-06	0.0002996	20.44612
rs204286	A	T	A	T	0.01	0.0029042	0.5	0.5969	0.7887	0.01088138	0.0022	4.69E-06	0.0002981	20.66116
rs2597193	A	G	A	G	0.0187	0.0177567	0.5	0.2751	0.1345	0.01164311	0.0022	6.26E-17	0.0009729	72.25
rs3755967	T	C	T	C	-0.0892	0	0.5	0.2426	0.9787	0.01241276	0.0023	0	0.0188639	1504.091
rs4821976	A	G	A	G	0.01	0.0085363	0.5	0.534	0.4216	0.01047219	0.0021	2.74E-06	0.0003396	22.67574
rs6780224	T	C	T	C	-0.0105	0.0001	0.5	0.3235	0.9913	0.01186148	0.0023	4.46E-06	0.000304	20.84121
rs6982502	T	C	T	C	-0.0094	0.0054146	0.5	0.5704	0.6266	0.0111676	0.002	4.04E-06	0.0003019	22.09
rs7011866	T	G	C	T	-0.0404	-0.4890641	0.5	0.9987	0.05736	0.16454821	0.0084	1.35E-06	0.0003976	23.13152
rs7011866	T	G	T	G	-0.0404	0.0032051	0.5	0.0353	0.938	0.04052219	0.0084	1.35E-06	0.0003976	23.13152
rs7675387	T	G	T	G	0.0181	0.0246001	0.5	0.296	0.03456	0.0113477	0.0023	4.18E-15	0.0009903	61.93006
rs7781168	A	G	A	G	0.0096	0.0108587	0.5	0.3827	0.314	0.01067219	0.002	2.95E-06	0.0003015	23.04
rs793000	A	G	A	G	0.0099	0.0167393	0.5	0.555	0.115	0.01042321	0.0022	4.90E-06	0.0002952	20.25
rs8018720	C	G	C	G	-0.0168	0.0058169	0.5	0.8278	0.6647	0.01344643	0.0029	4.72E-09	0.0004923	33.56005
rs904856	A	G	A	G	0.0228	0.0334348	0.5	0.9312	0.0956	0.02052781	0.0045	4.12E-07	0.000339	25.67111
rs914787	T	C	T	C	0.0105	0.0057163	0.5	0.3854	0.6244	0.01167423	0.0022	1.25E-06	0.0003456	22.77893

Retinol

SNP	effect_allel	other_allel	effect_allel	other_allel	beta.exposure	beta.outcome	eaf.exposur	eaf.outcom	pval.outcome	se.outcome	se.exposure	pval.exposure	R2	Fstat
rs1667226	T	A	T	A	0.101271	-0.0176549	0.520114	0.505	0.09784	0.01048571	0.0154605	5.74E-11	6.18E-05	42.9065
rs1883711	C	G	C	G	-0.27778	0.0485602	0.029437	0.0238	0.2706	0.04206837	0.0454347	9.73E-10	5.38E-05	37.37891

Vitamin K (1st model)

SNP	effect_allel	other_allel	effect_allel	other_allel	beta.exposure	beta.outcome	eaf.exposur	eaf.outcom	pval.outcome	se.outcome	se.exposure	pval.exposure	R2	Fstat
rs2108622	T	C	T	C	0.16	0.0163327	0.3	0.2525	0.1814	0.01203163	0.032	8.78E-07	0.0115687	25
rs2192574	C	T	C	T	0.28	0.003992	0.11	0.1355	0.8094	0.0185273	0.058	1.82E-06	0.0107931	23.30559
rs4645543	T	C	T	C	-0.42	-0.001998	0.04	0.0433	0.9416	0.02511735	0.079	2.00E-07	0.0130597	28.2647
rs4852146	C	T	C	T	0.18	-0.004008	0.33	0.3164	0.7422	0.0120375	0.037	2.08E-06	0.0109586	23.66691
rs964184	G	C	G	C	0.23	0.0109399	0.15	0.1629	0.4219	0.01382117	0.042	5.91E-08	0.0138453	29.98866

Vitamin K (2nd model)

SNP	effect_allel	other_allel	effect_allel	other_allel	beta.exposure	beta.outcome	eaf.exposur	eaf.outcom	pval.outcome	se.outcome	se.exposure	pval.exposure	R2	Fstat
rs2108622	T	C	T	C	0.16	0.0163327	0.3	0.2525	0.1814	0.01203163	0.031	2.90E-07	0.0123178	26.63892
rs2192574	C	T	C	T	0.28	0.003992	0.11	0.1355	0.8094	0.0185273	0.057	1.49E-06	0.0111709	24.1305
rs4122275	A	G	A	G	-0.81	0.0004001	0.02	0.0923	0.984	0.01959745	0.16	4.31E-07	0.0118563	25.62891
rs4852146	C	T	C	T	0.19	-0.004008	0.33	0.3164	0.7422	0.0120375	0.035	1.42E-07	0.0136088	29.46939
rs964184	G	C	G	C	0.14	0.0109399	0.15	0.1629	0.4219	0.01382117	0.042	5.91E-08	0.0051749	11.11111

Vitamin K (3rd model)

SNP	effect_allel	other_allel	effect_allel	other_allel	beta.exposure	beta.outcome	eaf.exposur	eaf.outcom	pval.outcome	se.outcome	se.exposure	pval.exposure	R2	Fstat

rs2108622	T	C	T	C	0.16	0.0163327	0.3	0.2525	0.1814	0.01203163	0.032	9.68E-07	0.0115687	25
rs2192574	C	T	C	T	0.29	0.003992	0.11	0.1355	0.8094	0.0185273	0.058	8.23E-07	0.0115687	25
rs4122275	A	G	A	G	-0.78	0.0004001	0.02	0.0923	0.984	0.01959745	0.17	3.90E-06	0.0097596	21.0519
rs4852146	C	T	C	T	0.18	-0.004008	0.33	0.3164	0.7422	0.0120375	0.038	3.23E-06	0.0103953	22.43767

Vitamin B12

SNP	effect_allel	other_allel	effect_allel	other_allel	beta.exposur	beta.outcom	eaf.exposur	eaf.outcom	pval.outcome	se.outcome	se.exposure	pval.exposure	R2	Fstat
rs1131603	C	T	C	T	0.22	-0.0089597	0.06	0.0401	0.751	0.02790765	0.015	4.90E-49	0.004698	215.1111
rs1141321	C	T	C	T	0.07	0.0109399	0.63	0.6775	0.3307	0.01136888	0.007	3.60E-26	0.0021895	100
rs1801222	G	A	G	A	0.12	0.0030045	0.59	0.6942	0.7942	0.01139413	0.007	3.30E-75	0.006407	293.8776
rs2270655	G	C	G	C	0.1	-0.0334534	0.94	0.9519	0.1358	0.02170918	0.015	2.20E-13	0.0009743	44.44444
rs2336573	T	C	T	C	0.31	0.0357308	0.03	0.0889	0.03443	0.01632321	0.019	8.40E-59	0.0058074	266.205
rs34324219	C	A	C	A	0.23	0	0.88	0.8998	0.9943	0.01741888	0.011	1.10E-111	0.0095018	437.1901
rs3742801	T	C	T	C	0.05	0.0164343	0.29	0.3288	0.1214	0.01042372	0.007	1.70E-13	0.0011184	51.02041
rs41281112	C	T	C	T	0.18	-0.0480354	0.95	0.9777	0.1834	0.03446403	0.015	8.90E-35	0.0031497	144
rs602662	A	G	A	G	0.17	0.0006002	0.6	0.4996	0.9513	0.01027806	0.007	2.40E-139	0.0127784	589.7959

Vitamin B6

SNP	effect_allel	other_allel	effect_allel	other_allel	beta.exposur	beta.outcom	eaf.exposur	eaf.outcom	pval.outcome	se.outcome	se.exposure	pval.exposure	R2	Fstat
rs4654748	C	T	C	T	1.45	0.003992	0.5	0.5842	0.6922	0.01124617	0.281	8.00E-18	0.0089998	26.62707

Alpha-tocopherol

SNP	effect_allel	other_allel	effect_allel	other_allel	beta.exposur	beta.outcom	eaf.exposur	eaf.outcom	pval.outcome	se.outcome	se.exposure	pval.exposure	R2	Fstat
rs964184	G	C	G	C	0.04	0.0109399	0.15	0.1629	0.4219	0.01382117	0.01	8.00E-12	0.0042317	16

Gamma and beta-tocopherol

SNP	effect_allel	other_allel	effect_allel	other_allel	beta.exposur	beta.outcom	eaf.exposur	eaf.outcom	pval.outcome	se.outcome	se.exposure	pval.exposure	R2	Fstat
rs11705639	A	C	C	T	0.1257807	-0.0008003	0.1685	0.8396	0.9576	0.01460663	0.0138	5.00E-20	0.0143751	83.07485
rs62508088	T	C	T	C	0.1648766	-0.0198026	0.0992	0.078	0.324	0.02036709	0.0179	1.00E-21	0.0146764	84.84223

Magnesium

SNP	effect_allel	other_allel	effect_allel	other_allel	beta.exposur	beta.outcom	eaf.exposur	eaf.outcom	pval.outcome	se.outcome	se.exposure	pval.exposure	R2	Fstat
rs10043693	A	G	A	G	0.03589	-0.0163327	0.6921	0.7194	0.1758	0.01185153	0.004433	5.63E-16	0.0004501	65.54679
rs1035283	G	A	G	A	-0.06605	-0.0201007	0.9368	0.9172	0.2618	0.01759031	0.00826	1.27E-15	0.0004391	63.9419
rs10747045	T	G	T	G	-0.02741	0.0178399	0.6696	0.7114	0.1477	0.0123	0.004336	2.57E-10	0.0002744	39.96129
rs10952168	G	A	G	A	0.02429	-0.0251127	0.4401	0.4234	0.02278	0.01073724	0.004443	4.57E-08	0.0002053	29.88838
rs10974444	G	C	G	C	0.02687	-0.0291199	0.6607	0.7065	0.01288	0.01136327	0.004233	2.19E-10	0.0002767	40.29385
rs11234579	C	T	C	T	-0.02714	0	0.6373	0.6998	0.9742	0.01272066	0.004453	1.10E-09	0.0002551	37.14619
rs112510641	G	A	G	A	0.03035	0.0139029	0.6104	0.6666	0.2225	0.01175204	0.00418	3.86E-13	0.000362	52.71872
rs113174770	T	G	T	G	0.04893	0.0217615	0.9334	0.9428	0.3472	0.02358189	0.008128	1.74E-09	0.0002489	36.23957
rs115478735	A	T	A	T	-0.04194	-0.0349021	0.8064	0.8411	0.01198	0.01342883	0.00508	1.52E-16	0.000468	68.15998
rs11614506	T	C	T	C	0.03278	-0.0009004	0.7819	0.8086	0.9509	0.0139023	0.004892	2.09E-11	0.0003083	44.89986
rs11694498	T	A	T	A	-0.04061	-0.0007002	0.6086	0.5668	0.9504	0.01077194	0.004166	1.88E-22	0.0006523	95.02272
rs117672478	C	A	C	A	-0.08527	0.0751075	0.9763	0.9812	0.0961	0.04883954	0.01506	1.51E-08	0.0002202	32.05845
rs12203597	G	A	G	A	-0.03104	0.0109399	0.3435	0.4468	0.3272	0.01163801	0.004361	1.10E-12	0.0003479	50.66071
rs12230212	T	A	T	A	0.1637	-0.0086372	0.989	0.9907	0.914	0.07980026	0.02743	2.39E-09	0.0002446	35.61605
rs1229984	T	C	T	C	0.07801	-0.0804511	0.03517	0.0343	0.005709	0.02686378	0.01116	2.79E-12	0.0003356	48.8621
rs12464156	T	C	T	C	-0.02627	-0.0003	0.5962	0.6294	0.9769	0.0107699	0.004107	1.58E-10	0.000281	40.91389
rs1273884	G	A	G	A	0.04993	-0.0086372	0.5595	0.555	0.4093	0.01035969	0.004091	2.87E-34	0.0010222	148.9581
rs12743084	C	G	C	G	-0.08845	-0.0136933	0.4615	0.4321	0.1752	0.00993878	0.004065	5.97E-105	0.0032419	473.4505
rs12918968	A	C	A	C	-0.03944	0.0099503	0.5741	0.5117	0.3559	0.01104745	0.004172	3.25E-21	0.0006135	89.36865
rs13143189	G	A	G	A	0.02938	-0.0069239	0.5727	0.6205	0.5151	0.01061352	0.004194	2.47E-12	0.000337	49.07347
rs13146355	G	A	G	A	-0.06525	-0.0152152	0.5558	0.6191	0.174	0.01098954	0.004024	3.91E-59	0.001803	262.933
rs13170671	C	T	C	T	0.02378	0.0188218	0.5502	0.5962	0.09134	0.01139719	0.004199	1.48E-08	0.0002203	32.07244
rs13193692	G	T	G	T	-0.03149	0.0266419	0.7824	0.8052	0.05693	0.01455995	0.005055	4.69E-10	0.0002665	38.80637
rs142601087	T	C	T	C	0.02922	-0.0286053	0.806	0.7794	0.0178	0.01173291	0.005168	1.57E-08	0.0002196	31.968
rs143135527	C	T	C	T	0.08315	-0.0345914	0.9719	0.9773	0.4274	0.0422074	0.01462	1.28E-08	0.0002222	32.34668
rs17794420	G	A	G	A	0.02859	-0.013592	0.5174	0.5879	0.2547	0.01178699	0.004373	6.19E-11	0.0002935	42.74343
rs17832417	A	T	A	T	0.03899	0.0059821	0.6265	0.6506	0.5831	0.01154923	0.004404	8.47E-19	0.0005382	78.38119
rs1853392	A	G	A	G	-0.05664	-0.0059175	0.09745	0.09351	0.8256	0.02657015	0.008654	5.98E-11	0.0002942	42.83637
rs1890185	A	G	A	G	-0.02732	-0.0211215	0.5875	0.5928	0.04177	0.01018112	0.004063	1.75E-11	0.0003105	45.21346
rs219782	A	G	A	G	-0.06371	-0.0161294	0.7483	0.7291	0.1626	0.0113949	0.004632	4.75E-43	0.0012979	189.1813
rs2439722	T	C	T	C	-0.02569	-0.0118702	0.528	0.5278	0.2711	0.01066352	0.00424	1.36E-09	0.0002521	36.71102
rs250383	A	T	A	T	0.05615	0.0109399	0.8149	0.7627	0.3678	0.01242653	0.005266	1.53E-26	0.0007804	113.6941
rs2510467	A	G	A	G	0.03135	-0.001401	0.4299	0.3904	0.9064	0.01187551	0.004437	1.59E-12	0.0003428	49.92249
rs2542713	A	C	A	C	0.02645	-0.0067225	0.4504	0.4621	0.5068	0.01013036	0.004186	2.64E-10	0.0002742	39.92573
rs2731238	G	A	G	A	0.05395	0.0178399	0.2993	0.3595	0.1149	0.01151556	0.004601	9.56E-32	0.0009436	137.4923
rs2731238	G	T	C	T	-0.1227	0.0178399	0.9493	0.3595	0.1149	0.01151556	0.009953	6.53E-35	0.0010429	151.9781
rs2731238	G	A	C	A	0.05395	0.0099503	0.2993	0.9438	0.6717	0.02487041	0.004601	9.56E-32	0.0009436	137.4923
rs2731238	G	T	G	T	-0.1227	0.0099503	0.9493	0.9438	0.6717	0.02487041	0.009953	6.53E-35	0.0010429	151.9781
rs2818759	G	T	G	T	0.03013	-0.0067225	0.344	0.2907	0.5891	0.01234898	0.00441	8.39E-12	0.0003206	46.67895
rs28441180	G	A	G	A	0.02463	-0.0170444	0.491	0.4779	0.1331	0.01115026	0.004398	2.15E-08	0.0002154	31.36306
rs303968	T	C	T	C	-0.03532	-0.0002	0.6028	0.5748	0.9876	0.01072372	0.004198	3.98E-17	0.000486	70.78749
rs34872471	T	C	T	C	0.04058	-0.2075163	0.7066	0.7019	3.60E-89	0.00842015	0.004434	5.56E-20	0.0005751	83.75924
rs35249105	A	G	A	G	-0.04637	0.0256677	0.538	0.5852	0.02031	0.01118929	0.004121	2.25E-29	0.000869	126.6103
rs35347302	G	A	G	A	-0.02671	-0.0030045	0.7702	0.7905	0.8125	0.01263265	0.004774	2.20E-08	0.000215	31.30278
rs35465213	G	A	G	A	-0.1494	0.0610951	0.9477	0.9531	0.01557	0.02691505	0.009452	2.85E-56	0.0017133	249.8353
rs35934	G	T	G	T	-0.05568	-0.0038072	0.1741	0.1736	0.7922	0.01453673	0.005631	4.71E-23	0.0006712	97.7749
rs3732215	G	C	G	C	-0.02364	-0.0069756	0.4801	0.4498	0.5576	0.01113801	0.003994	3.26E-09	0.0002406	35.03312
rs3783297	T	C	T	C	0.02579	-0.0153167	0.6433	0.5832	0.1707	0.0109977	0.004293	1.89E-09	0.0002479	36.0895
rs3824347	A	G	A	G	0.05695	0.003992	0.5864	0.619	0.7267	0.0113727	0.004257	8.35E-41	0.0012279	178.97
rs3848132	T	A	T	A	-0.04543	0.0089597	0.723	0.7474	0.4781	0.01292398	0.004775	1.82E-21	0.0006214	90.51879
rs3925584	T	C	T	C	0.07105	-0.0009004	0.5475	0.6131	0.9395	0.011225	0.00402	6.73E-70	0.0021413	312.3748
rs4077450	G	T	G	T	-0.03324	0.0059821	0.2184	0.2621	0.6652	0.01298724	0.005478	1.31E-09	0.0002529	36.81951
rs425135	A	C	A	C	-0.03465	-0.0025031	0.1466	0.1824	0.8513	0.01323495	0.005797	2.27E-09	0.0002454	35.72727
rs4962402	T	G	T	G	-0.03358	0.0158733	0.2763	0.278	0.1809	0.01192959	0.004637	4.43E-13	0.0003601	52.44296
rs544934737	T	C	T	C	0.02519	0.0069756	0.5678	0.5272	0.5303	0.01197832	0.004601	4.36E-08	0.0002059	29.97449
rs560609863	T	G	T	G	-0.05315	-0.0127813	0.8518	0.8264	0.5254	0.01992296	0.009142	6.12E-09	0.0002321	33.80058
rs58447148	A	G	A	G	0.04638	-0.0159262	0.9139	0.8981	0.3787	0.01777806	0.007637	1.26E-09	0.0002533	36.88212
rs58560619	T	C	T	C	0.02335	-0.0130852	0.4936	0.5619	0.2096	0.01029566	0.004079	1.04E-08	0.0002251	32.76924
rs592859	C	G	C	G	0.03162	-0.0011006	0.1578	0.1704	0.9376	0.01406556	0.005773	4.31E-08	0.000206	29.99996
rs59359435	T	C	T	C	0.06372	0.0148886	0.9553	0.957	0.5666	0.02720995	0.01039	8.55E-10	0.0002583	37.61148
rs59685687	T	A	T	A	0.02874	0.0009995	0.7714	0.678	0.942	0.01314745	0.005196	3.16E-08	0.0002101	30.59393

rs6003469	A	C	A	C	0.04485	0	0.6924	0.7437	0.9976	0.012725	0.004437	5.11E-24	0.0007014	102.1753
rs606970	A	G	A	G	-0.02401	0.0119286	0.5289	0.5472	0.2761	0.01115408	0.00432	2.74E-08	0.0002122	30.88992
rs62136373	C	T	C	T	0.03313	-0.0095454	0.8549	0.85	0.5271	0.01498597	0.006058	4.51E-08	0.0002054	29.90779
rs623297	C	T	C	T	0.02897	-0.0077298	0.7303	0.7567	0.5559	0.01301556	0.004862	2.56E-09	0.0002438	35.50317
rs636264	G	A	G	A	-0.03387	0	0.2082	0.2005	0.9736	0.01390612	0.005236	9.93E-11	0.0002874	41.8438
rs658903	T	A	T	A	-0.03979	-0.0025031	0.855	0.8743	0.8813	0.01685587	0.006028	4.07E-11	0.0002992	43.57139
rs6667005	T	C	T	C	0.02947	-0.0108587	0.7844	0.7041	0.369	0.01191837	0.004962	2.86E-09	0.0002423	35.27335
rs6936263	G	C	G	T	-0.02746	-0.0065212	0.2853	0.6544	0.5823	0.01185408	0.004564	1.78E-09	0.0002486	36.2001
rs6936263	G	C	G	C	-0.02746	0.0059821	0.2853	0.3941	0.6001	0.01174362	0.004564	1.78E-09	0.0002486	36.2001
rs7039	C	G	C	G	0.03185	0.0139029	0.479	0.4466	0.1774	0.01081607	0.004072	5.26E-15	0.0004201	61.17914
rs7077696	A	G	A	G	0.02798	0.0069756	0.7797	0.7813	0.6131	0.01294821	0.00501	2.34E-08	0.0002142	31.19033
rs711819	T	C	T	C	-0.03593	0.0089597	0.2421	0.2056	0.5297	0.01422883	0.004965	4.61E-13	0.0003596	52.3692
rs7221296	A	G	A	G	-0.04431	-0.0086372	0.8126	0.7394	0.4928	0.01247117	0.005215	1.94E-17	0.0004957	72.19296
rs7241576	A	G	A	G	0.02973	0.0158733	0.6835	0.7246	0.1993	0.01273469	0.004521	4.82E-11	0.000297	43.2435
rs72848405	T	C	T	C	0.02757	0.0305292	0.8042	0.8182	0.02297	0.01376735	0.005049	4.74E-08	0.0002048	29.81692
rs7374260	T	C	T	C	0.02461	-0.0047111	0.5278	0.5131	0.6454	0.01015867	0.003992	7.04E-10	0.000261	38.00512
rs73949333	A	G	A	G	0.03287	0.0049875	0.776	0.716	0.6946	0.01243699	0.005211	2.84E-10	0.0002733	39.78847
rs7416991	T	C	A	C	0.05162	-0.0283995	0.3162	0.6456	0.005962	0.01002883	0.004405	1.04E-31	0.0009425	137.3233
rs7416991	T	G	T	G	-0.05192	-0.0283995	0.6967	0.6456	0.005962	0.01002883	0.004371	1.51E-32	0.0009683	141.0937
rs7416991	T	C	T	C	0.05162	0.0392207	0.3162	0.3964	0.000227	0.01091786	0.004405	1.04E-31	0.0009425	137.3233
rs7416991	T	G	A	G	-0.05192	0.0392207	0.6967	0.3964	0.000227	0.01091786	0.004371	1.51E-32	0.0009683	141.0937
rs7742789	C	T	C	T	0.05486	-0.0250102	0.6933	0.6183	0.0191	0.01042653	0.004413	1.76E-35	0.0010605	154.541
rs77759957	A	G	A	G	0.0746	-0.0322133	0.9728	0.9758	0.3813	0.03564184	0.01319	1.54E-08	0.0002197	31.98807
rs7797740	A	G	A	G	-0.03551	-0.026036	0.1499	0.1291	0.1152	0.01609719	0.006093	5.61E-09	0.0002333	33.96558
rs78433554	C	A	C	A	0.1049	0.0314987	0.986	0.9889	0.5862	0.05935689	0.0192	4.69E-08	0.000205	29.85029
rs7850067	A	G	A	G	0.04308	-0.011769	0.485	0.5106	0.2363	0.00985255	0.004081	4.79E-26	0.0007649	111.4341
rs7867868	C	T	C	T	-0.04157	0.0069756	0.6695	0.6904	0.5314	0.01186505	0.004504	2.69E-20	0.0005848	85.18503
rs7894336	C	T	C	T	0.06273	-0.0050125	0.4679	0.4713	0.6238	0.01016173	0.004011	4.00E-55	0.0016774	244.5937
rs7946549	T	A	T	A	0.04303	0.0009995	0.5441	0.5675	0.8953	0.01036837	0.004036	1.52E-26	0.0007802	113.6686
rs8068318	C	T	C	T	-0.08407	0.0207825	0.3015	0.4025	0.0743	0.01203801	0.004691	8.01E-72	0.0022015	321.182
rs9897596	T	C	T	C	-0.02319	-0.0096464	0.4911	0.4735	0.3662	0.01053087	0.004061	1.13E-08	0.000224	32.60885

Potassium

SNP	effect_allel	other_allel	effect_allel	other_allel	beta.exposur	beta.outcome	eaf.exposur	eaf.outcom	pval.outcome	se.outcome	se.exposure	pval.exposure	R2	Fstat
rs10032927	A	T	A	T	0.01561	-0.0277824	0.5837	0.5866	0.01014	0.01049184	0.002494	4.01E-10	6.49E-05	39.17535
rs10046175	C	A	C	A	0.03717	0.003992	0.9659	0.9672	0.8922	0.02672092	0.006432	7.72E-09	5.59E-05	33.39589
rs10086982	G	A	G	A	-0.02034	-0.0026034	0.7603	0.761	0.8533	0.01378954	0.003195	2.01E-10	6.71E-05	40.52847
rs10227075	T	C	T	C	-0.06498	-0.0608121	0.9796	0.9803	0.07634	0.03232245	0.00832	6.01E-15	0.0001021	60.9976
rs10264133	C	T	C	T	-0.05002	-0.0327298	0.9737	0.9747	0.2716	0.0287875	0.007193	3.67E-12	8.09E-05	48.35788
rs10265221	T	C	T	C	-0.03723	-0.0075283	0.7481	0.7447	0.5398	0.01221633	0.002886	5.03E-38	0.0002756	166.4154
rs10270510	G	C	G	C	-0.06498	-0.0608121	0.9796	0.9803	0.07634	0.03232245	0.00832	6.01E-15	0.0001021	60.9976
rs10279895	A	G	A	G	-0.06565	-0.06145	0.9797	0.9804	0.0734	0.03231735	0.008331	3.44E-15	0.0001039	62.09765
rs10502917	T	C	T	C	-0.02136	-0.0032051	0.3194	0.3169	0.7908	0.01202908	0.002766	1.20E-14	9.88E-05	59.63458
rs10748853	T	C	T	C	-0.03616	-0.0092426	0.1179	0.1162	0.5787	0.01640816	0.003902	2.01E-20	0.0001422	85.87808
rs1076485	C	T	C	T	0.02448	-0.0064206	0.815	0.821	0.6306	0.01331454	0.00308	2.01E-15	0.0001046	63.17153
rs10811662	G	A	G	A	0.01906	0.1380213	0.8401	0.8414	2.79E-22	0.01635179	0.003305	8.20E-09	5.51E-05	33.25852
rs11124938	C	A	C	A	-0.02548	-0.0093435	0.7812	0.7784	0.4688	0.01271837	0.002987	1.57E-17	0.0001205	72.76598
rs111375249	G	T	G	T	-0.05498	-0.0071253	0.9749	0.9758	0.8115	0.02987985	0.007261	3.84E-14	9.60E-05	57.33457
rs111607733	C	A	C	A	-0.05535	-0.0106566	0.9726	0.9736	0.7069	0.02812653	0.006865	7.85E-16	0.0001088	65.00611
rs111724190	T	C	T	C	-0.03147	0.0129162	0.9503	0.9524	0.5646	0.02346352	0.005554	1.49E-08	5.37E-05	32.10567
rs111972532	T	C	T	C	0.03879	-0.0047111	0.8139	0.812	0.7242	0.01319541	0.003086	3.39E-36	0.0002616	157.9966
rs11217192	T	G	T	G	-0.01709	0.0059821	0.7648	0.7659	0.6121	0.01277092	0.002949	7.04E-09	5.56E-05	33.58417
rs11245343	T	C	T	C	-0.02401	-0.0144032	0.7283	0.7268	0.2478	0.01230612	0.002872	6.53E-17	0.0001157	69.89007
rs11264363	G	C	G	C	0.0252	0.0382587	0.4285	0.4216	0.000488	0.01136276	0.002659	2.78E-21	0.0001487	89.81821
rs112734474	T	G	T	G	-0.07519	-0.0038072	0.9782	0.979	0.91	0.03373903	0.008227	6.70E-20	0.0001398	83.52898
rs114048605	C	T	C	T	-0.1319	-0.0358345	0.9894	0.9894	0.6855	0.08585204	0.02131	6.28E-10	0.0002177	38.31093
rs114323080	G	A	G	A	-0.1018	-0.0577351	0.9864	0.9865	0.4347	0.06995357	0.01812	1.99E-08	0.0001794	31.56306
rs115080005	C	T	C	T	0.03996	-0.0033055	0.9722	0.9732	0.9068	0.02848316	0.00691	7.55E-09	5.60E-05	33.4422
rs115223381	T	C	T	C	-0.1006	-0.0368715	0.9865	0.9865	0.6182	0.07161327	0.01814	3.03E-08	0.0001748	30.7554
rs11563587	T	A	T	A	-0.05898	-0.0390527	0.9824	0.9831	0.2741	0.0343625	0.00864	9.02E-12	7.80E-05	46.59959
rs11563956	C	T	C	T	-0.03679	-0.0001	0.9677	0.9689	0.9985	0.02682934	0.006511	1.64E-08	5.34E-05	31.92745
rs11563967	A	G	A	G	-0.03819	-0.0169427	0.9514	0.9533	0.4671	0.02298112	0.005682	1.85E-11	7.56E-05	45.17486
rs11563979	G	A	G	A	-0.04055	-0.0130852	0.9527	0.9546	0.5803	0.02339719	0.00576	1.98E-12	8.29E-05	49.56062
rs11563980	C	T	C	T	-0.03382	-0.018979	0.9393	0.9413	0.3841	0.02137398	0.005234	1.07E-10	6.99E-05	41.75224
rs11563986	T	C	T	C	-0.04416	0.0029955	0.9695	0.9707	0.9187	0.02798724	0.006736	5.74E-11	7.19E-05	42.97877
rs11563990	C	T	C	T	-0.03834	-0.0152152	0.9635	0.9649	0.5596	0.0256176	0.006325	1.38E-09	6.15E-05	36.74372
rs11564010	G	C	G	C	-0.06314	-0.0618754	0.9796	0.9804	0.07045	0.03218418	0.008287	2.66E-14	9.72E-05	58.05164
rs11564019	G	A	G	A	-0.06503	-0.0608121	0.9796	0.9804	0.07635	0.03231939	0.008322	5.79E-15	0.0001022	61.06215
rs11564024	T	G	T	G	-0.0652	-0.0621946	0.9299	0.9304	0.07146	0.0324477	0.008369	6.98E-15	0.0003449	60.69432
rs11564025	T	G	T	G	-0.06489	-0.0599623	0.9795	0.9802	0.08078	0.03237959	0.008315	6.31E-15	0.0001019	60.90193
rs11564194	C	T	C	T	-0.02113	0.0129162	0.8063	0.8034	0.3223	0.01329056	0.003081	7.27E-12	7.79E-05	47.0344
rs116232574	A	G	A	G	-0.1002	-0.0557241	0.9863	0.9864	0.4493	0.06990561	0.01806	2.96E-08	0.0001749	30.78222
rs116355131	C	A	C	A	0.06558	-0.0236781	0.9709	0.9711	0.6286	0.04788699	0.01191	3.76E-08	0.0001723	30.31931
rs11711982	T	C	T	C	0.0239	0.0276152	0.883	0.881	0.09392	0.01703036	0.003863	6.33E-10	6.34E-05	38.27775
rs11743019	A	G	A	G	-0.02052	-0.0188771	0.7361	0.7331	0.1468	0.01277296	0.002981	6.04E-12	7.85E-05	47.3839
rs11822294	C	T	C	T	-0.01797	-0.0025031	0.8251	0.8238	0.8519	0.01358214	0.003169	1.47E-08	5.33E-05	32.15523
rs11970777	C	T	C	T	-0.03918	-0.0362491	0.9638	0.9649	0.1636	0.02511276	0.006303	5.25E-10	6.40E-05	38.63974
rs11974568	T	C	T	C	-0.03409	-0.0463581	0.9488	0.9504	0.04426	0.02198444	0.005567	9.44E-10	6.21E-05	37.4983
rs11983963	T	A	T	A	-0.04007	-0.0288111	0.9647	0.9658	0.272	0.02549745	0.00636	3.08E-10	6.57E-05	39.69396
rs1214759	A	G	A	G	-0.01681	0.0237165	0.4192	0.4164	0.02782	0.01085383	0.002573	6.59E-11	7.07E-05	42.68309
rs12378991	G	A	G	A	-0.03902	0.0227395	0.9411	0.94	0.3338	0.02448673	0.005503	1.39E-12	8.33E-05	50.27771
rs12509595	T	C	T	C	0.03557	-0.0007002	0.7552	0.7555	0.9584	0.01273929	0.002892	9.95E-35	0.0002505	151.2764
rs12549572	T	C	T	C	0.02422	0.0139029	0.6728	0.6784	0.2396	0.01169821	0.002648	6.11E-20	0.0001385	83.65893
rs1273886	A	G	A	G	0.02013	-0.0005001	0.6749	0.6695	0.9688	0.01206097	0.002776	4.30E-13	8.71E-05	52.5834
rs1275979	T	C	T	C	-0.03297	-0.0148091	0.4719	0.4658	0.1826	0.01095434	0.002568	1.09E-37	0.0002729	164.8344
rs12766550	C	G	C	G	0.02881	0.0382587	0.9417	0.9413	0.08967	0.02323367	0.005162	2.44E-08	5.16E-05	31.14946
rs12803281	C	T	C	T	-0.01661	0.0029955	0.5864	0.582	0.7877	0.01069847	0.002465	1.66E-11	7.52E-05	45.40518
rs12940197	G	T	G	T	-0.02288	0.0178399	0.4361	0.4295	0.1736	0.01308138	0.002976	1.55E-14	9.79E-05	59.10799
rs12959198	T	C	T	C	-0.02103	-0.0009004	0.7725	0.7746	0.9414	0.01281633	0.002958	1.22E-12	8.37E-05	50.54547
rs12975656	G	A	G	A	0.02031	0.0009995	0.5826	0.5779	0.9308	0.01177245	0.0027	5.53E-14	9.37E-05	56.58383
rs13042148	C	T	C	T	-0.02296	-0.054773	0.8834	0.8813	0.000885	0.01557959	0.004082	1.91E-08	5.24E-05	31.63718
rs13072118	C	T	C	T	0.0161	-0.0133892	0.4291	0.4247	0.2391	0.01122423	0.002608	6.93E-10	6.31E-05	38.10979
rs13072590	G	A	G	A	0.01745	-0.0073268	0.786	0.7883	0.5952	0.01360204	0.003135	2.69E-08	5.13E-05	30.98245

rs13333693	A	G	A	G	0.02753	0.0049875	0.7554	0.7581	0.6583	0.01229362	0.00284	3.46E-22	0.0001556	93.96708
rs1336173	T	A	T	A	0.04684	-0.0332466	0.9711	0.9723	0.2937	0.03065128	0.007667	1.03E-09	6.25E-05	37.32354
rs138278364	C	A	C	A	-0.0861	-0.1196848	0.981	0.9811	0.03151	0.04945	0.01445	2.63E-09	0.0002018	35.50345
rs138304526	T	C	T	C	0.1353	0.2004889	0.9882	0.9882	0.02888	0.1127273	0.02115	1.63E-10	0.0002325	40.9237
rs138404919	C	T	C	T	-0.1012	-0.0566762	0.9865	0.9866	0.442	0.06994133	0.0181	2.29E-08	0.0001777	31.26107
rs138531692	G	A	G	A	-0.0861	-0.1196848	0.981	0.9811	0.03151	0.04945	0.01445	2.63E-09	0.0002018	35.50345
rs139145497	T	C	T	C	-0.086	-0.1235245	0.9811	0.9812	0.03783	0.0527051	0.01441	2.46E-09	0.0002024	35.61795
rs139316656	G	A	G	A	-0.0861	-0.1196848	0.981	0.9811	0.03151	0.04945	0.01445	2.63E-09	0.0002018	35.50345
rs139777921	T	A	T	A	-0.08715	-0.1128328	0.9828	0.9829	0.07318	0.05638597	0.01523	1.09E-08	0.0001861	32.74424
rs140318611	G	A	G	A	-0.08728	-0.1139529	0.9829	0.983	0.07049	0.05633342	0.01523	1.03E-08	0.0001866	32.84201
rs140501563	G	A	G	A	-0.08845	-0.1270382	0.982	0.9821	0.03826	0.05414209	0.01484	2.61E-09	0.0002019	35.52449
rs141016776	C	T	C	T	-0.08603	-0.1232982	0.981	0.9811	0.03785	0.05259847	0.01438	2.23E-09	0.0002034	35.79168
rs141017725	G	A	G	A	-0.08644	-0.1242036	0.9811	0.9812	0.0366	0.05259541	0.01439	1.93E-09	0.000205	36.08344
rs141935404	C	T	C	T	-0.02551	0.0207825	0.9136	0.9134	0.2663	0.01883163	0.004305	3.21E-09	5.82E-05	35.11354
rs142365336	G	T	G	T	0.1224	0.2062008	0.986	0.986	0.0123	0.1015676	0.01888	9.41E-11	0.0002388	42.02995
rs1427298	C	T	C	T	-0.01837	0.0158733	0.6201	0.6197	0.1268	0.01084209	0.002624	2.67E-12	8.12E-05	49.01067
rs142878005	T	C	T	C	-0.08632	-0.1231851	0.9811	0.9811	0.03812	0.05264821	0.01439	2.03E-09	0.0002045	35.98332
rs142887200	C	T	C	T	-0.06546	-0.0605996	0.9795	0.9803	0.07815	0.03237755	0.008327	3.98E-15	0.0001034	61.79806
rs143341259	T	C	T	C	0.04981	0.0516432	0.9743	0.9742	0.1702	0.03974158	0.008639	8.33E-09	5.56E-05	33.24345
rs143862634	T	G	T	G	-0.08676	-0.1118258	0.9828	0.9828	0.07366	0.05606352	0.01513	9.98E-09	0.0001869	32.88223
rs144261869	A	G	A	G	0.03665	0.0069756	0.9654	0.9668	0.7945	0.02672883	0.006401	1.06E-08	5.49E-05	32.78327
rs144726326	C	T	C	T	-0.0398	-0.0232686	0.9534	0.9553	0.3338	0.02349388	0.005839	9.65E-12	7.78E-05	46.46107
rs144808502	G	A	G	A	-0.08486	-0.1159722	0.9817	0.9819	0.05829	0.05467015	0.01482	1.06E-08	0.0001863	32.7876
rs144843179	G	A	G	A	0.03096	0.0069756	0.7948	0.7939	0.5585	0.01245663	0.002895	1.15E-26	0.0001894	114.3681
rs145391254	C	T	C	T	0.03637	0.001998	0.9666	0.9679	0.9549	0.02671556	0.006427	1.57E-08	5.36E-05	32.02359
rs145557956	A	G	A	G	-0.08581	-0.1201358	0.9809	0.981	0.04194	0.05249158	0.0143	2.02E-09	0.0002046	36.00839
rs146176062	C	T	C	T	-0.08591	-0.1222806	0.9809	0.981	0.03943	0.05265204	0.01438	2.35E-09	0.0002028	35.6919
rs146416293	C	G	C	G	-0.0861	-0.1196848	0.981	0.9811	0.03151	0.04945	0.01445	2.63E-09	0.0002018	35.50345
rs146745107	T	C	T	C	-0.08633	-0.1222806	0.981	0.9811	0.03968	0.0527051	0.0144	2.07E-09	0.0002042	35.94169
rs147888747	A	C	A	C	-0.08684	-0.1187835	0.9828	0.9829	0.06021	0.05627985	0.01531	1.45E-08	0.0001828	32.17283
rs148160104	A	G	A	G	-0.0861	-0.1196848	0.981	0.9811	0.03151	0.04945	0.01445	2.63E-09	0.0002018	35.50345
rs148227305	G	A	G	A	-0.08581	-0.1201358	0.9809	0.981	0.04194	0.05249158	0.0143	2.02E-09	0.0002046	36.00839
rs148768978	G	A	G	A	-0.08633	-0.1222806	0.981	0.9811	0.03968	0.0527051	0.0144	2.07E-09	0.0002042	35.94169
rs149474053	T	C	T	C	0.1332	0.2390169	0.9887	0.9887	0.01116	0.12030893	0.02144	5.38E-10	0.0002193	38.59747
rs149674884	G	A	G	A	0.0641	0.1106465	0.9555	0.9556	0.009662	0.04785944	0.01031	5.19E-10	0.0002197	38.65438
rs149727168	A	T	A	T	-0.08581	-0.1201358	0.9809	0.981	0.04194	0.05249158	0.0143	2.02E-09	0.0002046	36.00839
rs149923520	C	T	C	T	-0.0861	-0.1196848	0.981	0.9811	0.03151	0.04945	0.01445	2.63E-09	0.0002018	35.50345
rs149925191	G	T	G	T	0.03863	-0.0111621	0.9729	0.9739	0.6978	0.02850995	0.006972	3.08E-08	5.14E-05	30.69974
rs1502282	G	C	G	C	-0.01458	-0.0235757	0.5896	0.5916	0.02252	0.01007857	0.0025	5.62E-09	5.63E-05	34.01222
rs150305470	C	G	C	G	-0.08544	-0.1122732	0.9818	0.982	0.06551	0.05460561	0.01476	7.23E-09	0.0001904	33.5081
rs150464897	G	A	G	A	-0.08522	-0.1219417	0.981	0.9811	0.03979	0.05260128	0.01436	2.99E-09	0.0002001	35.21877
rs150589717	G	A	G	A	-0.1405	-0.0186733	0.9888	0.9889	0.8262	0.08367041	0.02043	6.45E-12	0.0002687	47.29507
rs1529897	T	G	A	T	-0.01533	-0.1760214	0.5376	0.9981	0.3802	0.17254056	0.002553	1.97E-09	5.97E-05	36.05643
rs1529897	T	G	T	G	-0.01533	-0.0083346	0.5376	0.5399	0.4473	0.01090995	0.002553	1.97E-09	5.97E-05	36.05643
rs1551287	C	T	C	T	0.02749	-0.0026034	0.2156	0.2172	0.8376	0.01273852	0.00299	4.04E-20	0.00014	84.52927
rs1574817	T	G	T	G	0.01733	0.0049875	0.3903	0.3877	0.6438	0.01124362	0.002612	3.35E-11	7.37E-05	44.02008
rs1609829	T	C	T	C	-0.01753	0.001998	0.293	0.2912	0.8669	0.01168852	0.002702	9.06E-11	6.97E-05	42.09138
rs164746	G	A	G	A	-0.02005	0.0099503	0.6178	0.6118	0.3677	0.01136939	0.002597	1.22E-14	9.87E-05	59.6053
rs17038613	C	T	C	T	0.1229	0.2070142	0.9859	0.9859	0.0114	0.10090612	0.01885	7.24E-11	0.0002416	42.50902
rs17038648	C	T	C	T	0.1127	0.1814879	0.9767	0.9767	0.003359	0.07420663	0.01432	3.74E-15	0.0003519	61.93866
rs17314270	T	C	T	C	0.1032	0.117783	0.9746	0.9746	0.04202	0.06538827	0.01385	9.40E-14	0.0003155	55.52133
rs17367435	T	C	T	C	-0.01507	-0.0050125	0.5555	0.5477	0.6654	0.0115375	0.002667	1.63E-08	5.29E-05	31.92864
rs17390839	G	A	G	A	0.02233	-0.0170444	0.837	0.8342	0.26	0.01487602	0.003495	1.73E-10	6.76E-05	40.82095
rs17472490	G	A	G	A	-0.04054	-0.0211215	0.9654	0.9667	0.4251	0.02589286	0.006389	2.28E-10	6.74E-05	40.26259
rs17472728	G	T	G	T	0.05347	-0.0323166	0.9855	0.986	0.4033	0.0374551	0.009379	1.22E-08	5.44E-05	32.50179
rs17472899	A	G	A	G	-0.05945	-0.0396768	0.9824	0.9832	0.2675	0.03442321	0.008667	7.20E-12	7.87E-05	47.0507
rs17473032	T	C	T	C	-0.06074	-0.0433251	0.9824	0.9831	0.2326	0.03475383	0.008775	4.65E-12	8.02E-05	47.91322
rs17473487	C	T	C	T	-0.06575	-0.0619818	0.9303	0.9308	0.07314	0.03255612	0.008394	4.99E-15	0.0003486	61.35551
rs17473690	G	T	G	T	-0.0628	-0.0598562	0.9811	0.9819	0.0929	0.03359439	0.008636	3.70E-13	8.85E-05	52.88031
rs17473844	T	C	T	C	-0.04674	-0.0119714	0.9708	0.972	0.6754	0.02826046	0.006952	1.85E-11	7.57E-05	45.20202
rs17473985	G	A	G	A	-0.03361	-0.0490852	0.9494	0.951	0.02742	0.02121148	0.005585	1.83E-09	6.00E-05	36.21518
rs17501084	A	G	A	G	-0.05527	-0.0447882	0.9818	0.9826	0.201	0.03352832	0.008478	7.30E-11	7.11E-05	42.50031
rs17501559	A	G	A	G	0.05322	-0.03015	0.9853	0.9859	0.4351	0.03754949	0.009367	1.37E-08	5.40E-05	32.28113
rs17502552	G	T	G	T	-0.06301	-0.0619818	0.9796	0.9804	0.07068	0.0322324	0.008296	3.21E-14	9.65E-05	57.68747
rs17502782	C	A	C	A	-0.0337	-0.0462534	0.9507	0.9523	0.04695	0.0222199	0.005628	2.18E-09	5.94E-05	35.85516
rs17502922	C	G	C	G	0.06535	-0.023371	0.9708	0.971	0.6326	0.04788827	0.0119	4.07E-08	0.0001714	30.15763
rs17502970	C	A	C	A	-0.04408	0.0049875	0.9695	0.9707	0.8452	0.02804719	0.006699	4.86E-11	7.25E-05	43.29754
rs1757225	A	G	A	G	-0.02184	-0.0171462	0.3802	0.3757	0.1194	0.01084668	0.002547	1.05E-17	0.0001218	73.52709
rs185372147	T	G	T	G	-0.06575	-0.0619818	0.9303	0.9308	0.07314	0.03255612	0.008394	4.99E-15	0.0003486	61.35551
rs190945315	A	C	A	C	-0.06543	-0.0641119	0.9302	0.9307	0.0638	0.03248138	0.008378	6.02E-15	0.0003465	60.99204
rs191079	T	C	T	C	0.01581	0.0119286	0.5793	0.5851	0.3145	0.01246378	0.002839	2.62E-08	5.14E-05	31.01223
rs191102591	G	T	G	T	-0.04406	0.0049875	0.9695	0.9707	0.8557	0.02793648	0.006699	4.98E-11	7.24E-05	43.25826
rs1979845	C	T	C	T	0.03115	0.0119286	0.8267	0.829	0.375	0.01417296	0.003306	4.62E-21	0.000147	88.77903
rs1984669	C	T	C	T	-0.0221	0.0401818	0.7592	0.76	0.000771	0.01232449	0.002834	6.66E-15	0.0001007	60.81138
rs1989061	T	C	T	C	0.01826	-0.0034058	0.3219	0.3256	0.7689	0.01169439	0.002709	1.62E-11	7.52E-05	45.43427
rs199680901	C	T	C	T	-0.01837	0.0069756	0.6385	0.6407	0.547	0.01134949	0.002631	3.01E-12	8.07E-05	48.75022
rs1997596	C	T	C	T	-0.01956	0.0139029	0.666	0.665	0.2309	0.01199923	0.002733	8.62E-13	8.48E-05	51.22222
rs2050542	T	C	T	C	0.04403	-0.0091417	0.9731	0.9743	0.7743	0.03155587	0.007712	1.16E-08	5.46E-05	32.59593
rs210139	A	C	A	C	-0.01598	0.0119286	0.5911	0.594	0.2603	0.01054592	0.0024	2.89E-11	7.34E-05	44.3334
rs2168785	C	T	C	T	0.01863	0.0276152	0.3675	0.3647	0.01251	0.01131888	0.002684	4.09E-12	7.98E-05	48.17932
rs2195880	C	G	C	G	0.01569	0.0139029	0.7004	0.6985	0.2347	0.01157577	0.002629	2.46E-09	5.90E-05	35.61761
rs219791	T	G	T	G	0.0173	-0.0038072	0.6668	0.6667	0.7433	0.01149184	0.002688	1.26E-10	6.86E-05	41.42225
rs2207404	G	A	G	A	-0.0484	-0.0097474	0.9636	0.9646	0.7081	0.02569821	0.006281	1.35E-14	9.83E-05	59.37904
rs2255390	A	G	A	G	0.02748	0.0139029	0.4329	0.4324	0.1867	0.01050791	0.002426	1.05E-29	0.0002125	128.3074
rs2327429	T	C	T	C	-0.02284	0.0049875	0.737	0.7387	0.652	0.01214005	0.002788	2.65E-16	0.0001111	67.113
rs2340534	G	A	G	A	0.01826	0.0198026	0.5376	0.5388	0.06112	0.01089898	0.002458	1.15E-13	9.14E-05	55.18713
rs2497318	C	T	C	T	-0.02938	-0.044579	0.6293	0.6246	3.44E-05	0.01029566	0.002633	6.77E-29	0.0002062	124.5093
rs2569882	T	C	T	C	-0.01474	-0.0110609	0.5253	0.5303	0.3005	0.01053342	0.002462	2.21E-09	5.94E-05	35.8442
rs2761244	T	C	T	C	0.01525	0.0079682	0.3969	0.3972	0.439	0.01077679	0.002484	8.53E-10	6.24E-05	37.6909
rs2823263	T	C	T	C	-0.02564	0.0139029	0.7425	0.7419	0.2746	0.01260179	0.002846	2.17E-19	0.0001344	81.16452
rs28398484	C	G	C	G	-0.04176	-0.0018016	0.9686	0.9699	0.9483	0.02770969	0.006668	3.89E-10	6.56E-05	39.22201
rs28398495	A	G	A	G	-0.03906	-0.0134906	0.9512	0.9531	0.5645	0.02312526	0.005686	6.69E-12	7.90E-05	47.19009
rs28398500	C	G	C	G	-0.0384	-0.0152152	0.9513	0.9533	0.5171	0.0231574	0.005685	1.49E-11	7.64E-05	45.62486

rs28398507	G	A	G	A	-0.03811	-0.0176549	0.9515	0.9534	0.4508	0.02304056	0.005688	2.15E-11	7.51E-05	44.89094
rs28398535	T	C	T	C	-0.03384	-0.020713	0.9598	0.9613	0.4034	0.02427526	0.005987	1.62E-08	5.35E-05	31.94789
rs28398544	A	G	A	G	-0.03329	-0.0173496	0.9595	0.9611	0.4796	0.02413495	0.005932	2.04E-08	5.27E-05	31.49382
rs28416181	T	G	T	G	0.02165	0.0148886	0.71	0.7112	0.1945	0.01149464	0.002647	3.04E-16	0.0001108	66.8972
rs28430881	C	A	C	A	-0.02156	0.0168571	0.5611	0.5601	0.1011	0.01066276	0.002429	7.34E-19	0.0001305	78.78481
rs28446035	C	T	C	T	0.03696	0.003992	0.9657	0.9671	0.8711	0.02656327	0.006433	9.40E-09	5.52E-05	33.00934
rs28558845	G	C	G	C	-0.02063	0.0009995	0.82	0.8229	0.9445	0.01320306	0.003066	1.76E-11	7.50E-05	45.27455
rs28570591	G	A	G	A	-0.06608	-0.0643252	0.9798	0.9806	0.06157	0.03228316	0.008333	2.30E-15	0.0001052	62.88359
rs3176466	C	T	C	T	0.04031	0.064851	0.8668	0.868	1.40E-05	0.01581556	0.003543	5.89E-30	0.0002144	129.4444
rs34070447	A	G	A	G	0.02013	0.0314987	0.5474	0.5493	0.001824	0.01026582	0.00238	2.89E-17	0.0001185	71.53748
rs34136790	C	T	C	T	0.02587	0.0029955	0.8161	0.8208	0.8701	0.01702245	0.004021	1.29E-10	6.93E-05	41.39279
rs34917191	T	C	T	C	-0.01332	-0.0060181	0.4203	0.4214	0.5564	0.01023673	0.002409	3.32E-08	5.06E-05	30.57277
rs35444	A	G	A	G	-0.0336	-0.0044097	0.6131	0.6124	0.672	0.01043929	0.00241	3.97E-44	0.0003218	194.3768
rs35619052	G	A	G	A	0.02036	-0.0120726	0.7815	0.7797	0.4412	0.01548444	0.003609	1.73E-08	5.33E-05	31.82598
rs35619990	C	T	C	T	-0.01762	-0.0138961	0.5513	0.5552	0.185	0.01033724	0.002433	4.59E-13	8.69E-05	52.44784
rs35812759	A	G	A	G	0.01552	-0.0011006	0.7522	0.752	0.9243	0.01184286	0.002755	1.79E-08	5.26E-05	31.73513
rs36092406	G	A	G	A	-0.04798	-0.011769	0.9635	0.9645	0.6496	0.02568903	0.006277	2.19E-14	9.68E-05	58.42736
rs3751767	T	C	T	C	-0.01723	-0.0372866	0.6902	0.6914	0.001768	0.01148622	0.002891	2.59E-09	5.88E-05	35.52012
rs3790604	C	A	C	A	0.04697	-0.0185714	0.9322	0.9339	0.3895	0.02115969	0.004789	1.12E-22	0.0001593	96.19477
rs3802177	G	A	G	A	0.01547	0.0667236	0.7385	0.7381	9.02E-09	0.01237296	0.00278	2.70E-08	5.13E-05	30.96642
rs3848132	T	A	T	A	-0.03504	0.0089597	0.7463	0.7474	0.4781	0.01292398	0.002922	4.26E-33	0.0002381	143.8029
rs4114858	A	G	A	G	0.01947	0.0049875	0.8371	0.8392	0.7563	0.0147676	0.003459	1.87E-08	5.25E-05	31.68333
rs4413681	G	C	G	C	0.06552	-0.0253178	0.9709	0.9711	0.6064	0.04802347	0.01196	4.44E-08	0.0001705	30.01134
rs4461961	C	T	C	T	0.01818	0.0217615	0.6471	0.651	0.0543	0.01166429	0.002611	3.43E-12	8.03E-05	48.48127
rs4485922	A	G	A	G	-0.01442	-0.0130852	0.46	0.4632	0.2156	0.01042245	0.002442	3.56E-09	5.78E-05	34.86898
rs4677143	A	G	A	G	0.01596	0.0119286	0.6379	0.639	0.2533	0.0108	0.002609	9.70E-10	6.20E-05	37.42119
rs4737371	G	A	G	A	-0.02244	0.0129162	0.8207	0.8197	0.3672	0.01424158	0.003276	7.74E-12	7.77E-05	46.91999
rs4745804	T	C	T	C	-0.01661	-0.0143018	0.5313	0.5293	0.1999	0.01096964	0.002577	1.20E-10	6.88E-05	41.5442
rs4766578	T	A	T	A	0.01805	0.0544882	0.3993	0.4073	1.19E-06	0.01192679	0.002668	1.39E-11	7.58E-05	45.77019
rs4809849	C	T	C	T	0.01779	0.0168571	0.4172	0.4206	0.1526	0.01180306	0.002675	3.03E-11	7.33E-05	44.22872
rs4846476	G	C	G	C	0.01665	0.0059821	0.7784	0.779	0.6098	0.01253138	0.002851	5.38E-09	5.65E-05	34.10626
rs4848713	C	T	C	T	0.02442	0.0029955	0.8902	0.8933	0.8644	0.01888546	0.004187	5.65E-09	5.63E-05	34.01617
rs4867732	C	G	C	G	0.02908	0.0129162	0.9196	0.9237	0.537	0.02118418	0.004584	2.30E-10	6.67E-05	40.24386
rs4924538	A	T	A	T	-0.01606	-0.0036065	0.5128	0.5114	0.7527	0.0112727	0.002619	8.81E-10	6.23E-05	37.60279
rs4936409	A	G	A	G	-0.01693	-0.0131866	0.491	0.492	0.2309	0.01082577	0.002558	3.75E-11	7.25E-05	43.80391
rs547340372	G	A	G	A	-0.05554	-0.0596439	0.976	0.977	0.05353	0.02910714	0.007827	1.33E-12	8.43E-05	50.35251
rs55650455	T	C	T	C	-0.01947	-0.0217345	0.8204	0.8179	0.1258	0.01391582	0.003288	3.26E-09	5.81E-05	35.06455
rs56218647	C	A	C	A	0.02388	0.0089597	0.9123	0.9116	0.6173	0.0181852	0.004155	9.36E-09	5.47E-05	33.03137
rs56237637	T	A	T	A	0.04136	-0.0411345	0.9645	0.9659	0.1616	0.02824796	0.007107	6.08E-09	5.67E-05	33.86791
rs56329049	G	A	G	A	-0.04065	-0.0031048	0.9643	0.9656	0.9072	0.02630153	0.006417	2.45E-10	6.65E-05	40.12888
rs56376587	A	C	A	C	-0.02835	0.0029955	0.5817	0.5782	0.8018	0.01124337	0.002564	2.19E-28	0.0002025	122.256
rs56790613	A	G	A	G	0.0377	0.0059821	0.9673	0.9686	0.8129	0.02719082	0.006576	1.01E-08	5.50E-05	32.86693
rs569550	T	G	T	G	0.03941	0.0266419	0.6382	0.6391	0.02078	0.01161378	0.002628	8.92E-51	0.0003723	224.8859
rs57420239	C	T	C	T	-0.03706	0.001998	0.9678	0.969	0.9412	0.02723367	0.006577	1.80E-08	5.31E-05	31.75083
rs57484993	A	G	A	G	0.03418	0.0168571	0.9219	0.9214	0.4215	0.02075587	0.004607	1.22E-13	9.12E-05	55.04371
rs576943137	C	T	C	T	-0.02548	-0.0004001	0.8922	0.8913	0.9831	0.01956913	0.004521	1.79E-08	5.26E-05	31.76361
rs578097	A	G	A	G	-0.02281	-0.0087381	0.652	0.6533	0.4092	0.01046403	0.002544	3.25E-19	0.0001331	80.39265
rs57850577	A	G	A	G	-0.05408	0.0029955	0.9691	0.9702	0.9059	0.02687168	0.006479	7.43E-17	0.0001166	69.67186
rs57944734	C	T	C	T	-0.04826	-0.0106566	0.9619	0.963	0.6746	0.02518367	0.006162	5.02E-15	0.0001016	61.33823
rs58320941	G	T	G	T	-0.04411	0.0029955	0.9695	0.9708	0.9129	0.02774235	0.006735	5.96E-11	7.18E-05	42.89423
rs59482369	T	C	T	C	-0.04242	-0.0158246	0.959	0.9606	0.5403	0.02541148	0.006235	1.05E-11	7.75E-05	46.288
rs60085321	C	G	C	G	-0.05059	-0.0236781	0.9688	0.9698	0.3926	0.02705893	0.006686	4.02E-14	9.48E-05	57.25278
rs6013199	G	A	G	A	-0.07916	-0.0477207	0.9722	0.9725	0.3811	0.05208214	0.01317	1.89E-09	0.0002053	36.12768
rs6031431	A	G	A	G	0.01961	0.0079682	0.5497	0.5491	0.481	0.01070179	0.002464	1.81E-15	0.0001049	63.33938
rs60772526	C	T	C	T	0.0605	0.0079682	0.127	0.1234	0.6285	0.01634388	0.003746	1.27E-58	0.0004318	260.8406
rs6108787	T	G	T	G	0.01367	0.0109399	0.5405	0.5419	0.2818	0.01046888	0.002403	1.31E-08	5.36E-05	32.36156
rs61169316	G	A	G	A	0.01814	-0.0046106	0.7215	0.7228	0.7012	0.01192755	0.002753	4.56E-11	7.19E-05	43.41723
rs61793790	G	C	G	C	-0.03709	0.0069756	0.9597	0.9598	0.8139	0.02849898	0.006649	2.49E-08	5.21E-05	31.11724
rs61897793	G	A	G	A	-0.02178	-0.0094445	0.8642	0.8628	0.547	0.01547398	0.003653	2.56E-09	5.95E-05	35.5481
rs62193645	C	T	C	T	0.03441	0.0276152	0.8796	0.8776	0.09517	0.01674158	0.003785	1.04E-19	0.0001369	82.649
rs62330329	T	C	T	C	0.02703	0.0089597	0.2697	0.2709	0.6427	0.01947168	0.004425	1.04E-09	6.18E-05	37.31346
rs62374068	A	G	A	G	0.05943	0.0276152	0.7821	0.78	0.02891	0.01278852	0.002918	3.67E-92	0.0006866	414.802
rs62435145	G	T	G	T	-0.0256	0.003992	0.434	0.4276	0.7288	0.01171148	0.0027	2.68E-21	0.0001489	89.89849
rs66482211	A	G	A	G	-0.04204	0.0516432	0.9505	0.9499	0.03174	0.02551862	0.005675	1.34E-13	9.09E-05	54.87742
rs66561220	T	C	T	C	-0.02949	-0.015723	0.7728	0.7734	0.2311	0.01296301	0.003016	1.49E-22	0.0001583	95.60638
rs6697367	C	T	C	T	-0.01868	0	0.6768	0.6756	0.9853	0.01112041	0.002575	4.19E-13	8.72E-05	52.62587
rs6703881	T	C	T	C	-0.02296	-0.0102524	0.1968	0.1975	0.4414	0.01314949	0.00312	1.91E-13	8.97E-05	54.1545
rs6716091	C	T	C	T	0.02352	0.003992	0.7446	0.7426	0.7612	0.01213827	0.002796	4.19E-17	0.0001172	70.76203
rs6755070	G	T	G	T	0.01832	-0.0104545	0.6877	0.6886	0.3828	0.01180612	0.002757	3.10E-11	7.31E-05	44.15475
rs6794202	C	T	C	T	0.02384	0.0629748	0.7896	0.7862	5.70E-07	0.0134574	0.003025	3.39E-15	0.0001029	62.11003
rs6816915	G	A	G	A	-0.01441	-0.0037069	0.4714	0.4769	0.7404	0.0111176	0.002594	2.83E-08	5.11E-05	30.85944
rs6826742	G	T	G	T	-0.01667	0.0119286	0.4695	0.4654	0.2338	0.01046709	0.00243	7.13E-12	7.79E-05	47.06073
rs6877631	T	C	T	C	-0.0154	-0.0011006	0.6149	0.6105	0.9191	0.01107883	0.002594	2.99E-09	5.84E-05	35.24532
rs690054	C	T	C	T	-0.03594	0.0069756	0.5832	0.5775	0.5123	0.01032679	0.002501	8.68E-47	0.0003419	206.5041
rs6934923	G	T	C	T	-0.05549	-0.0945305	0.9748	0.9987	0.6012	0.16808342	0.007262	2.25E-14	9.77E-05	58.38711
rs6934923	G	T	G	T	-0.05549	-0.0064206	0.9748	0.9757	0.8321	0.02989286	0.007262	2.25E-14	9.77E-05	58.38711
rs6941546	G	A	G	A	-0.02568	-0.0047111	0.9129	0.9159	0.8031	0.01865128	0.004349	3.63E-09	5.77E-05	34.86673
rs6961048	C	G	C	G	0.04642	0.0478373	0.8895	0.889	0.00314	0.01701582	0.003761	5.76E-35	0.0002523	152.3364
rs697976	T	C	T	C	0.01364	0.0207825	0.4779	0.4761	0.0366	0.00996633	0.0024	1.34E-08	5.35E-05	32.30028
rs7038668	T	C	T	C	-0.0279	-0.0007002	0.5014	0.5041	0.9477	0.01055306	0.002425	1.38E-30	0.0002192	132.3686
rs71537958	G	C	G	C	-0.01936	0.0089597	0.7389	0.7386	0.4788	0.01267883	0.002891	2.22E-11	7.43E-05	44.84505
rs716877	C	G	C	G	0.03596	-0.0030045	0.5162	0.5082	0.7941	0.01141199	0.002627	1.35E-42	0.0003103	187.3783
rs7187932	G	A	G	A	-0.0223	-0.0027037	0.7746	0.7773	0.8267	0.01232015	0.002875	9.15E-15	9.96E-05	60.16363
rs7218857	T	C	T	C	-0.02113	-0.0133892	0.5618	0.5668	0.2066	0.01049566	0.002471	1.30E-17	0.0001211	73.12292
rs7222451	G	A	G	A	-0.01388	-0.0150121	0.4975	0.5021	0.1589	0.01052474	0.002471	1.99E-08	5.23E-05	31.55248
rs72969822	C	G	C	G	-0.0249	-0.013592	0.8821	0.8807	0.4075	0.01614337	0.003796	5.55E-11	7.13E-05	43.02752
rs73776711	G	A	G	A	-0.0479	-0.0093435	0.9631	0.9641	0.715	0.0252773	0.006173	8.96E-15	9.97E-05	60.21137
rs7416991	T	C	A	C	0.01931	-0.0283995	0.4025	0.6456	0.005962	0.01002883	0.002593	1.01E-13	9.18E-05	55.4574
rs7416991	T	G	T	G	-0.01795	-0.0283995	0.6404	0.6456	0.005962	0.01002883	0.002504	7.97E-13	8.51E-05	51.38783
rs7416991	T	C	T	C	0.01931	0.0392207	0.4025	0.3964	0.000227	0.01091786	0.002593	1.01E-13	9.18E-05	55.4574
rs7416991	T	G	A	G	-0.01795	0.0392207	0.6404	0.3964	0.000227	0.01091786	0.002504	7.97E-13	8.51E-05	51.38783
rs74496669	G	T	G	T	0.03972	-0.0064206	0.9698	0.971	0.8289	0.02945765	0.00716	2.96E-08	5.15E-05	30.7746
rs74518739	C	T	C	T	-0.04059	-0.0127813	0.9527	0.9546	0.5904	0.02344566	0.005761	1.91E-12	8.31E-05	49.64121

rs75248620	G	A	G	A	0.03605	-0.0261387	0.9368	0.9358	0.2421	0.02174413	0.00518	3.55E-12	8.02E-05	48.43408
rs75411357	A	T	A	T	0.03822	0.0099503	0.9656	0.967	0.7187	0.02829311	0.006723	1.35E-08	5.41E-05	32.31879
rs75741381	C	G	C	G	-0.01862	-0.0119714	0.7999	0.8042	0.3734	0.01328648	0.003124	2.60E-09	5.88E-05	35.52526
rs76075621	T	C	T	C	0.03689	0.003992	0.9658	0.9672	0.8708	0.02689235	0.006443	1.05E-08	5.49E-05	32.78242
rs761356	T	C	T	C	0.02838	0.0178399	0.6163	0.6138	0.09894	0.01083189	0.002531	3.76E-29	0.0002082	125.7305
rs76200870	G	A	G	A	-0.05573	-0.0142003	0.9737	0.9748	0.6249	0.02865485	0.007021	2.17E-15	0.0001054	63.00574
rs76666870	T	C	T	C	0.03689	0.003992	0.9658	0.9672	0.8708	0.02689235	0.006443	1.05E-08	5.49E-05	32.78242
rs76821958	A	G	A	G	-0.04048	-0.0304592	0.9734	0.9743	0.2903	0.02794133	0.006957	6.08E-09	5.61E-05	33.85611
rs76987554	C	T	C	T	-0.07778	0.0266419	0.982	0.9827	0.4719	0.03788929	0.009027	7.31E-18	0.0001243	74.24189
rs77081469	C	A	C	A	0.06558	-0.0236781	0.9709	0.9711	0.6286	0.04788699	0.01191	3.76E-08	0.0001723	30.31931
rs7726795	T	C	T	C	0.02854	0.003992	0.6314	0.6336	0.7318	0.01152474	0.002657	7.04E-27	0.0001911	115.3785
rs77290217	T	C	T	C	-0.04754	-0.0198966	0.9649	0.9662	0.4645	0.02662832	0.006553	4.18E-13	8.81E-05	52.63056
rs7749375	G	A	G	A	-0.05485	-0.0021022	0.975	0.9759	0.9439	0.03026888	0.007327	7.38E-14	9.38E-05	56.04036
rs7766997	A	G	A	G	-0.05467	-0.0048116	0.9749	0.9758	0.8734	0.02988724	0.007245	4.68E-14	9.53E-05	56.94051
rs7767054	G	A	G	A	-0.05415	-0.0010005	0.9743	0.9752	0.9727	0.02980357	0.00719	5.22E-14	9.49E-05	56.72038
rs77747253	G	T	G	T	-0.1012	-0.0566762	0.9865	0.9866	0.442	0.06994133	0.0181	2.29E-08	0.0001777	31.26107
rs780101	C	T	C	T	0.02158	-0.025523	0.5339	0.5343	0.01356	0.01008291	0.002504	7.30E-18	0.000123	74.27356
rs78132593	C	A	C	A	0.01948	0.0535408	0.8381	0.835	0.000178	0.01511913	0.003462	1.87E-08	5.24E-05	31.66094
rs7836572	T	C	T	C	0.02144	0.0168571	0.4105	0.4141	0.1264	0.01092194	0.002459	2.91E-18	0.0001259	76.02081
rs78716205	G	A	G	A	0.0679	-0.005113	0.9669	0.9665	0.8637	0.0294875	0.006869	5.19E-23	0.0001618	97.71304
rs79030490	C	A	C	A	-0.07534	0.0217615	0.9817	0.9825	0.5609	0.03740816	0.008966	4.59E-17	0.0001182	70.60798
rs79285851	C	T	C	T	-0.1018	-0.0590072	0.9864	0.9865	0.4245	0.06983342	0.01811	1.93E-08	0.0001796	31.59793
rs79392591	C	G	C	G	-0.0245	0.0009995	0.8683	0.8667	0.9538	0.01539898	0.003564	6.53E-12	7.83E-05	47.25598
rs79557018	C	T	C	T	-0.05487	-0.0055152	0.9749	0.9758	0.8542	0.02988801	0.007246	3.81E-14	9.60E-05	57.34206
rs79590186	C	T	C	T	-0.07237	-0.020611	0.976	0.9769	0.5312	0.03220612	0.007995	1.49E-19	0.0001371	81.93703
rs79889868	G	C	G	C	-0.05507	0.0029955	0.9696	0.9707	0.9183	0.02753291	0.00659	6.80E-17	0.0001169	69.83278
rs80176668	A	G	A	G	-0.07368	0.0089597	0.9774	0.9782	0.7837	0.0323125	0.007748	2.05E-21	0.0001513	90.43156
rs80289662	C	G	C	G	-0.05427	-0.0001	0.9694	0.9705	0.998	0.02734005	0.006589	1.86E-16	0.0001135	67.83919
rs80328223	C	T	C	T	-0.07624	-0.0043093	0.9784	0.9792	0.8984	0.03367781	0.008251	2.62E-20	0.0001429	85.3793
rs8077544	G	A	G	A	0.01629	-0.0060181	0.3428	0.3387	0.6297	0.01239847	0.00285	1.13E-08	5.41E-05	32.67025
rs8125560	A	C	A	C	-0.01906	-0.001401	0.825	0.8227	0.9173	0.01345153	0.003137	1.28E-09	6.11E-05	36.91618
rs820429	G	T	G	T	-0.04348	-0.0304592	0.6755	0.676	0.01234	0.01179949	0.002787	7.92E-55	0.000403	243.3913
rs880315	T	C	T	C	0.04161	0.0129162	0.6739	0.6749	0.2514	0.01122985	0.002572	7.72E-59	0.0004333	261.73
rs923000	A	C	A	C	0.02374	0.003992	0.637	0.6312	0.7119	0.01143801	0.002662	4.99E-19	0.0001317	79.53263
rs9321413	C	G	C	G	0.03641	0.0079682	0.9653	0.9667	0.773	0.02674796	0.006408	1.36E-08	5.40E-05	32.28467
rs9321414	A	C	A	C	0.03723	0.0139029	0.9642	0.9656	0.6208	0.0279023	0.006659	2.30E-08	5.23E-05	31.25849
rs9483608	A	G	A	G	0.03662	0.003992	0.9657	0.967	0.8939	0.02634005	0.006344	7.99E-09	5.58E-05	33.32041
rs9483610	G	A	G	A	0.03663	0.003992	0.9657	0.967	0.8934	0.02654311	0.00635	8.18E-09	5.57E-05	33.27564
rs9483614	C	T	C	T	0.03847	0.0059821	0.9663	0.9676	0.8385	0.02803495	0.006761	1.30E-08	5.42E-05	32.37595
rs9493708	C	T	C	T	0.03629	0.003992	0.9655	0.9669	0.8883	0.02656709	0.006343	1.09E-08	5.48E-05	32.7329
rs9493713	C	A	C	A	0.03717	0.003992	0.9659	0.9672	0.8922	0.02672092	0.006432	7.72E-09	5.59E-05	33.39589
rs9493714	C	T	C	T	0.03664	0.0029955	0.9646	0.966	0.9209	0.02625816	0.006371	9.05E-09	5.54E-05	33.07469
rs9493720	T	C	T	C	0.03665	0.0069756	0.9654	0.9668	0.7945	0.02672883	0.006401	1.06E-08	5.49E-05	32.78327
rs9493722	T	C	T	C	0.03711	0.003992	0.9659	0.9672	0.8922	0.02687143	0.006433	8.22E-09	5.57E-05	33.27782
rs9493723	T	C	T	C	0.03665	0.0069756	0.9654	0.9668	0.7945	0.02672883	0.006401	1.06E-08	5.49E-05	32.78327
rs9493727	A	G	A	G	0.03721	0.0129162	0.9641	0.9656	0.6444	0.02800153	0.006656	2.32E-08	5.23E-05	31.25307
rs9493728	A	G	A	G	0.03714	0.0129162	0.9642	0.9656	0.6437	0.02800689	0.006656	2.48E-08	5.21E-05	31.1356
rs9493729	G	T	G	T	0.03978	-0.0064206	0.9699	0.9711	0.829	0.02945536	0.007166	2.92E-08	5.16E-05	30.81598
rs9603367	C	T	C	T	0.05097	-0.0008003	0.609	0.6024	0.9432	0.01161684	0.002692	7.22E-80	0.0005934	358.4917
rs963837	T	C	T	C	0.0215	0.001998	0.6211	0.6178	0.8751	0.01174821	0.002687	1.29E-15	0.000106	64.02382
rs9819344	C	T	C	T	0.03573	0.003992	0.9518	0.9537	0.8783	0.02392755	0.005812	8.11E-10	6.33E-05	37.7933
rs9821489	G	A	G	A	0.04282	0.0227395	0.8886	0.8923	0.2267	0.01937398	0.004507	2.20E-21	0.0001495	90.26476
rs9822474	A	G	A	G	-0.01603	-0.0010005	0.5519	0.5537	0.9203	0.01038342	0.00239	2.06E-11	7.45E-05	44.98536
rs9844949	A	G	A	G	0.01972	-0.0490852	0.152	0.1528	0.000852	0.01401862	0.003549	2.83E-08	5.11E-05	30.87463
rs9918487	G	A	G	A	-0.05331	0.003992	0.9685	0.9696	0.8738	0.02658342	0.006354	5.13E-17	0.0001178	70.39194
rs9928653	T	C	T	C	-0.01848	0.0029955	0.7631	0.7644	0.7879	0.01231276	0.002844	8.46E-11	6.99E-05	42.22258

Iron

SNP	effect_allel	other_allel	effect_allel	other_allel	beta.exposur	beta.outcom	eaf.exposu	eaf.outcom	pval.outcome	se.outcome	se.exposure	pval.exposure	R2	Fstat
rs10831924	T	C	T	C	0.06204	-0.0382212	0.9131	0.9298	0.04981	0.01875281	0.007982	8.06E-15	0.0004736	60.41157
rs12328766	A	G	A	G	0.03348	-0.0131866	0.6129	0.6125	0.2113	0.0104301	0.004341	1.29E-14	0.0004663	59.48279
rs13007705	C	T	C	T	-0.02771	-0.0056157	0.544	0.5444	0.5747	0.01002296	0.004194	4.05E-11	0.0003423	43.65321
rs13081052	T	C	T	C	0.03643	-0.0139975	0.248	0.2309	0.287	0.01297602	0.005395	1.51E-11	0.0003575	45.59691
rs140393761	A	G	A	G	-0.123	-0.0494004	0.9874	0.9864	0.3052	0.04595842	0.0201	9.56E-10	0.0002963	37.44709
rs150375987	C	G	C	G	-0.1303	-0.0609184	0.9792	0.9779	0.1022	0.03512679	0.01552	4.96E-17	0.0005575	70.48641
rs1800562	G	A	G	A	-0.3257	-0.0390527	0.9509	0.949	0.09956	0.02281735	0.01006	1.05E-229	0.0082266	1048.189
rs190399027	G	A	G	A	-0.1314	-0.0905814	0.9894	0.9885	0.04909	0.04210893	0.02111	4.98E-10	0.0003065	38.74487
rs1958078	A	C	A	C	-0.03145	-0.011769	0.2447	0.2307	0.3634	0.01285051	0.005338	3.94E-09	0.0002722	34.71236
rs218248	G	A	G	A	-0.03636	0.0246926	0.8382	0.8466	0.0858	0.01452755	0.00575	2.64E-10	0.0003135	39.98638
rs2294915	C	T	C	T	-0.03195	-0.0490852	0.7616	0.7625	2.49E-05	0.01108138	0.005059	2.78E-10	0.0003127	39.88525
rs28929474	C	T	C	T	-0.1763	0.0059821	0.9866	0.9856	0.8896	0.04565944	0.0188	7.11E-21	0.0006954	87.9405
rs555273273	C	T	C	T	0.1873	0.0478373	0.984	0.9859	0.5433	0.08294388	0.03115	1.88E-09	0.0008551	36.15426
rs5995385	T	C	T	C	0.02734	-0.0095454	0.5347	0.5275	0.3437	0.0099398	0.004294	1.98E-10	0.0003179	40.53899
rs72839066	A	G	A	G	-0.1163	0.003992	0.9694	0.9674	0.9057	0.03227832	0.01327	1.99E-18	0.0006075	76.80997
rs7385804	C	A	C	A	-0.05785	-0.0171462	0.357	0.3643	0.1118	0.01058036	0.004417	3.75E-39	0.0013436	171.5347
rs7775698	C	T	C	T	-0.0471	0.0129162	0.7576	0.7578	0.2731	0.01197628	0.004874	4.56E-22	0.0007319	93.38363
rs806970	C	T	C	T	0.05669	0.0324672	0.945	0.942	0.1517	0.02361378	0.009471	2.22E-09	0.0002834	35.82789
rs8177252	C	A	C	A	-0.05473	-0.0031048	0.6848	0.6811	0.7857	0.01126301	0.00463	3.25E-32	0.0010948	139.7298
rs855791	A	G	A	G	-0.1527	-0.012275	0.3768	0.3846	0.2669	0.01091122	0.004498	2.09E-252	0.0089587	1152.495
rs9402686	G	A	G	A	-0.05435	0.0109399	0.7844	0.7768	0.3761	0.01298827	0.005267	6.25E-25	0.0008345	106.4811

Zinc

SNP	effect_allel	other_allel	effect_allel	other_allel	beta.exposur	beta.outcom	eaf.exposu	eaf.outcom	pval.outcome	se.outcome	se.exposure	pval.exposure	R2	Fstat
rs10484100	G	A	G	A	-0.209	0.0178585	0.5	0.1123	0.2715	0.01592015	0.045	3.30E-06	0.0082251	21.57086
rs11763353	G	A	G	A	-0.192	0.0041084	0.5	0.179	0.761	0.0135102	0.039	7.00E-07	0.0092322	24.23669
rs1532423	A	G	A	G	0.178	-0.0047111	0.5	0.3754	0.6777	0.01123469	0.026	6.40E-12	0.017701	46.86982
rs2120019	C	T	C	T	-0.287	-0.0109399	0.5	0.26	0.3289	0.01184158	0.033	1.55E-18	0.0282583	75.63728
rs4333127	A	G	A	G	0.218	0.0054146	0.5	0.8977	0.7659	0.0181727	0.047	3.00E-06	0.0082035	21.51381
rs7148590	A	G	A	G	-0.14	-0.0139029	0.5	0.5387	0.1869	0.01074872	0.026	1.40E-07	0.0110244	28.99408

Copper

SNP	effect_allel	other_allel	effect_allel	other_allel	beta.exposur	beta.outcom	eaf.exposu	eaf.outcom	pval.outcome	se.outcome	se.exposure	pval.exposure	R2	Fstat
rs10014072	G	A	G	A	-0.164	0.020611	0.5	0.662	0.0726	0.01126173	0.034	1.00E-06	0.0088659	23.26644

rs1175550	A	G	A	G	-0.198	0.0009995	0.5	0.7192	0.9414	0.01314719	0.032	5.00E-10	0.0145059	38.28516
rs12153606	T	G	T	G	-0.159	-0.0305292	0.5	0.1555	0.0339	0.01478163	0.034	2.00E-06	0.008338	21.86938
rs12582659	C	T	C	T	1.262	-0.0089597	0.5	0.0411	0.7656	0.03114413	0.27	3.00E-06	0.0083295	21.84697
rs2769264	G	T	G	T	0.313	0.0029042	0.5	0.2216	0.8091	0.01208801	0.034	3.00E-20	0.0315548	84.74827
rs3857536	T	C	T	C	-0.129	-0.0059821	0.5	0.5686	0.5939	0.01064031	0.028	4.00E-06	0.0080946	21.22577

Selenium

SNP	effect_allel	other_allel	effect_allel	other_allel	beta.exposur	beta.outcom	eaf.exposur	eaf.outcom	pval.outcome	se.outcome	se.exposure	pval.exposure	R2	Fstat
rs7700970	T	C	T	C	0.265	-0.003992	0.5	0.3241	0.7211	0.01203036	0.035	7.17E-13	0.0103621	57.32653

Carotene

SNP	effect_allel	other_allel	effect_allel	other_allel	beta.exposur	beta.outcom	eaf.exposur	eaf.outcom	pval.outcome	se.outcome	se.exposure	pval.exposure	R2	Fstat
rs10846742	A	G	A	G	-0.143552	-0.0207825	0.832123	0.7653	0.1176	0.0136551	0.0208154	5.33E-12	6.85E-05	47.56078
rs9708919	T	C	T	C	0.246529	-0.0168571	0.480938	0.4464	0.09906	0.01055306	0.0153225	3.03E-58	0.0003725	258.867

Folate

SNP	effect_allel	other_allel	effect_allel	other_allel	beta.exposur	beta.outcom	eaf.exposur	eaf.outcom	pval.outcome	se.outcome	se.exposure	pval.exposure	R2	Fstat
rs1801133	G	A	G	A	0.096	-0.0137947	0.668	0.6986	0.236	0.01151454	0.01077	1.00E-28	0.0036471	79.45314
rs652197	C	T	C	T	0.069	0	0.179	0.2228	0.9749	0.01468265	0.01109	2.50E-10	0.0017803	38.71106

Table S6 : Harmonization results for exposure SNPs and Verma et al. (Hispanic/Latin American) T1D outcome

Outcome: Verma, A (T1D)
Hispanic or Latin American

Vitamin C

SNP	effect_allel	other_allel	effect_allel	other_allel	beta.exposure	beta.outcome	eaf.exposure	eaf.outcome	pval.outcome	se.outcome	se.exposure	pval.exposure	R2	Fstat
rs10051765	T	C	T	C	-0.039	0.000670391	0.6585	0.7109	0.9177	0.00650865	0.0066	3.64E-09	0.000671	34.91736
rs10136000	A	G	A	G	0.0404	-0.002718997	0.2825	0.7592	0.6935	0.00690101	0.0071	1.33E-08	0.000622	32.3777
rs10758628	A	C	A	C	0.0304	-0.008092446	0.4567	0.5641	0.1738	0.00595033	0.0063	1.28E-06	0.000447	23.28445
rs10995578	C	G	C	G	0.0296	0.002951293	0.4784	0.4896	0.6171	0.00590259	0.0062	1.91E-06	0.000438	22.79292
rs11062357	T	C	T	C	0.0305	0.00053201	0.5118	0.7305	0.9362	0.00665013	0.0063	1.46E-06	0.00045	23.43789
rs11641245	C	G	C	G	0.042	-0.009625749	0.848	0.8149	0.2051	0.00759728	0.0086	1.13E-06	0.000458	23.85073
rs11641245	C	G	C	G	0.042	-0.072043554	0.848	0.9973	0.2051	0.05686153	0.0086	1.13E-06	0.000458	23.85073
rs1165189	A	C	A	C	-0.0378	0.001902418	0.7469	0.8059	0.7989	0.00746046	0.0071	1.18E-07	0.000545	28.34438
rs117885456	A	G	A	G	0.0781	-0.007329398	0.0865	0.9485	0.5833	0.01335045	0.0116	1.70E-11	0.000871	45.33004
rs12610033	A	T	A	T	-0.0336	-0.000411529	0.2831	0.3189	0.9482	0.00633121	0.0073	4.86E-06	0.000407	21.18521
rs13028225	T	C	T	C	0.1016	0.013839223	0.8569	0.8691	0.1136	0.00874793	0.0089	2.38E-30	0.002499	130.3189
rs174547	T	C	T	C	-0.0364	-0.006870147	0.6721	0.4911	0.2446	0.00590219	0.0066	3.84E-08	0.000584	30.4169
rs185137552	T	C	T	C	0.2368	-0.030597324	0.988	0.9988	0.7196	0.08522931	0.0392	1.58E-09	0.000732	36.49146
rs2366388	A	G	A	G	0.0309	0.0009052	0.4908	0.5356	0.8786	0.00591634	0.0063	8.50E-07	0.000462	24.05669
rs2559850	A	G	A	G	0.0583	-0.008163523	0.5979	0.4047	0.1746	0.00601143	0.0064	6.30E-20	0.001593	82.98071
rs2941484	T	C	T	C	0.0341	0.003752054	0.4514	0.6327	0.5402	0.00612081	0.0063	6.37E-08	0.000563	29.2973
rs33972313	T	C	T	C	-0.3601	0.02049665	0.0319	0.9721	0.2525	0.01791665	0.0179	4.61E-90	0.00772	404.7065
rs339969	A	C	A	C	-0.0302	0.002646972	0.6068	0.3826	0.6625	0.00607104	0.0063	1.86E-06	0.000442	22.97909
rs4867910	T	C	T	C	-0.0314	-0.007939064	0.657	0.4479	0.1809	0.00593353	0.0068	3.45E-06	0.00041	21.32266
rs542903	T	C	T	C	-0.0418	0.021369629	0.1429	0.1458	0.01058	0.00836057	0.0088	2.27E-06	0.000434	22.5625
rs56738967	C	G	C	G	0.041	0.003477565	0.321	0.216	0.6275	0.00717024	0.0067	7.62E-10	0.000719	37.44709
rs6482188	A	C	A	C	-0.0347	-0.005127975	0.3086	0.735	0.443	0.00668576	0.0068	2.83E-07	0.0005	26.04001
rs6693447	T	G	T	G	0.0393	-0.000251704	0.5509	0.5871	0.9666	0.00599295	0.0064	6.25E-10	0.000724	37.70728
rs676317	T	C	T	C	-0.0366	0.002894937	0.7264	0.7425	0.6678	0.00674811	0.0074	7.33E-07	0.00047	24.46238
rs73035571	A	G	A	G	0.035	0.005459498	0.7863	0.8934	0.5679	0.00956129	0.0076	4.14E-06	0.000408	21.20845
rs73850547	A	G	A	G	0.0333	-0.000683995	0.7381	0.6922	0.9151	0.00639248	0.0071	2.85E-06	0.000423	21.99742
rs7640441	A	C	A	C	0.0358	0.00109262	0.2383	0.7968	0.8813	0.00733302	0.0074	1.19E-06	0.00045	23.40467
rs7740812	A	G	A	G	-0.0384	0.008649897	0.4057	0.9744	0.6433	0.01868228	0.0064	1.88E-09	0.000692	36
rs7740812	A	G	A	G	-0.0384	0.003180854	0.4057	0.756	0.6433	0.0068701	0.0064	1.88E-09	0.000692	36
rs78575870	T	C	T	C	0.0551	0.01400101	0.077	0.9589	0.3463	0.01486307	0.0117	2.46E-06	0.000426	22.17846
rs79234109	A	G	A	G	0.0453	-0.003496842	0.1617	0.8386	0.663	0.00802028	0.0084	7.70E-08	0.000559	29.08291
rs80246741	A	G	A	G	0.1511	-0.03628879	0.9897	0.9956	0.4155	0.04458082	0.0319	2.21E-06	0.000431	22.43611
rs868822	T	G	T	G	-0.0329	-0.009850315	0.2648	0.3237	0.1184	0.00630622	0.0071	3.30E-06	0.000413	21.47213
rs9895661	T	C	T	C	0.0625	0.001641765	0.817	0.4372	0.7827	0.00594842	0.0081	1.05E-14	0.001143	59.53742
rs9915323	A	T	A	T	0.0321	0.007356372	0.2967	0.3973	0.2223	0.00602981	0.0068	2.60E-06	0.000428	22.28395

Vitamin D

SNP	effect_allel	other_allel	effect_allel	other_allel	beta.exposure	beta.outcome	eaf.exposure	eaf.outcome	pval.outcome	se.outcome	se.exposure	pval.exposure	R2	Fstat
rs10741657	A	G	A	G	0.0308	0.002829525	0.5	0.374	0.643	0.00609811	0.0022	2.05E-46	0.002854	196
rs10745742	T	C	T	C	0.0165	0.004700767	0.5	0.4599	0.427	0.00592036	0.0022	1.88E-14	0.000813	56.25
rs10888491	A	G	A	G	0.012	-0.008670742	0.5	0.4354	0.1451	0.00595109	0.0022	8.62E-08	0.000429	29.75207
rs11195965	A	T	A	T	-0.0157	0.007162767	0.5	0.8803	0.4306	0.00908981	0.0032	6.31E-07	0.00034	24.07129
rs11203339	T	C	T	C	-0.0104	-0.007934639	0.5	0.7053	0.2201	0.00647197	0.0021	8.56E-07	0.000314	24.52608
rs12507653	A	T	A	T	0.0118	0.005312256	0.5	0.3419	0.3932	0.00622044	0.0025	2.21E-06	0.000354	22.2784
rs12785878	T	G	T	G	0.0363	-0.009709552	0.5	0.4797	0.1002	0.00590605	0.0022	3.81E-62	0.003464	272.25
rs17082722	T	C	T	C	-0.0703	-0.010106954	0.5	0.987	0.6979	0.02604885	0.0149	2.22E-06	0.00064	22.26066
rs17216707	T	C	T	C	0.0263	-0.00820703	0.5	0.7271	0.2155	0.00662391	0.0027	8.14E-23	0.001326	94.88203
rs1809851	A	T	A	T	0.0104	0.009311802	0.5	0.6409	0.1301	0.00615046	0.0023	4.86E-06	0.0003	20.44612
rs204286	A	T	A	T	0.01	-0.007622312	0.5	0.5467	0.1984	0.00592715	0.0022	4.69E-06	0.000298	20.66116
rs2597193	A	G	A	G	0.0187	-0.000265392	0.5	0.7674	0.9695	0.00698399	0.0022	6.26E-17	0.000973	72.25
rs3755967	T	C	T	C	-0.0892	0.003916249	0.5	0.7671	0.5747	0.00698084	0.0023	0	0.018864	1504.091
rs4821976	A	G	A	G	0.01	-0.001431356	0.5	0.6154	0.8132	0.00606507	0.0021	2.74E-06	0.00034	22.67574
rs6780224	T	C	T	C	-0.0105	0.013868166	0.5	0.7822	0.0524	0.00714854	0.0023	4.46E-06	0.000304	20.84121
rs6982502	T	C	T	C	-0.0094	0.005990655	0.5	0.5085	0.3103	0.00590212	0.002	4.04E-06	0.000302	22.09
rs7011866	T	G	T	C	-0.0404	0.043222729	0.5	0.9712	0.01429	0.01764193	0.0084	1.35E-06	0.000398	23.13152
rs7011866	T	G	T	C	-0.0404	0.32336005	0.5	0.9995	0.01429	0.13198369	0.0084	1.35E-06	0.000398	23.13152
rs7675387	T	G	T	G	0.0181	0.001969555	0.5	0.7721	0.7798	0.00703413	0.0023	4.18E-15	0.00099	61.93006
rs7781168	A	G	A	G	0.0096	-0.001137535	0.5	0.5843	0.8491	0.00598703	0.002	2.95E-06	0.000301	23.04
rs793000	A	G	A	G	0.0099	0.002706256	0.5	0.4222	0.6503	0.00597408	0.0022	4.90E-06	0.000295	20.25
rs8018720	C	G	C	G	-0.0168	-0.005695934	0.5	0.2218	0.4228	0.00710216	0.0029	4.72E-09	0.000492	33.56005
rs904856	A	G	A	G	0.0228	-0.011669775	0.5	0.9073	0.2514	0.01017417	0.0045	4.12E-07	0.000339	25.67111
rs914787	T	C	T	C	0.0105	-0.004187816	0.5	0.5506	0.4802	0.00593175	0.0022	1.25E-06	0.000346	22.77893

Retinol (vitamin A)

SNP	effect_allel	other_allel	effect_allel	other_allel	beta.exposure	beta.outcome	eaf.exposure	eaf.outcome	pval.outcome	se.outcome	se.exposure	pval.exposure	R2	Fstat
rs1667226	T	A	T	A	0.101271	0.006465028	0.520114	0.5156	0.2733	0.00590414	0.0154605	5.74E-11	6.18E-05	42.9065
rs1883711	C	G	C	G	-0.27778	-0.045668471	0.029437	0.0182	0.03853	0.02207273	0.0454347	9.73E-10	5.38E-05	37.37891

Vitamin K (1st model)

SNP	effect_allel	other_allel	effect_allel	other_allel	beta.exposure	beta.outcome	eaf.exposure	eaf.outcome	pval.outcome	se.outcome	se.exposure	pval.exposure	R2	Fstat
rs2108622	T	C	T	C	0.16	-0.004407645	0.3	0.7451	0.5153	0.00677058	0.032	8.78E-07	0.011569	25
rs2192574	C	T	C	T	0.28	0.03212811	0.11	0.90204	0.001209	0.00992527	0.058	1.82E-06	0.010793	23.30559
rs4645543	T	C	T	C	-0.42	-0.00294981	0.04	0.9794	0.8868	0.02077331	0.079	2.00E-07	0.01306	28.2647
rs4852146	C	T	C	T	0.18	0.006560229	0.33	0.7118	0.3138	0.00651463	0.037	2.08E-06	0.010959	23.66691
rs964184	G	C	G	C	0.23	0.003469376	0.15	0.2498	0.6109	0.00681606	0.042	5.91E-08	0.013845	29.98866
**(2nd model)**														
SNP	effect_allel	other_allel	effect_allel	other_allel	beta.exposure	beta.outcome	eaf.exposure	eaf.outcome	pval.outcome	se.outcome	se.exposure	pval.exposure	R2	Fstat
rs2108622	T	C	T	C	0.16	-0.004407645	0.3	0.7451	0.5153	0.00677058	0.031	2.90E-07	0.012318	26.63892
rs2192574	C	T	C	T	0.28	0.03212811	0.11	0.90204	0.001209	0.00992527	0.057	1.49E-06	0.011171	24.1305
rs4122275	A	G	A	G	-0.81	-0.002420012	0.02	0.8935	0.8	0.00956527	0.16	4.31E-07	0.011856	25.62891
rs4852146	C	T	C	T	0.19	0.006560229	0.33	0.7118	0.3138	0.00651463	0.035	1.42E-07	0.013609	29.46939
rs964184	G	C	G	C	0.14	0.003469376	0.15	0.2498	0.6109	0.00681606	0.042	5.91E-08	0.005175	11.11111
**(3rd model)**														
SNP	effect_allel	other_allel	effect_allel	other_allel	beta.exposure	beta.outcome	eaf.exposure	eaf.outcome	pval.outcome	se.outcome	se.exposure	pval.exposure	R2	Fstat
rs2108622	T	C	T	C	0.16	-0.004407645	0.3	0.7451	0.5153	0.00677058	0.032	9.68E-07	0.011569	25
rs2192574	C	T	C	T	0.29	0.03212811	0.11	0.90204	0.001209	0.00992527	0.058	8.23E-07	0.011569	25
rs4122275	A	G	A	G	-0.78	-0.002420012	0.02	0.8935	0.8	0.00956527	0.17	3.90E-06	0.00976	21.0519
rs4852146	C	T	C	T	0.18	0.006560229	0.33	0.7118	0.3138	0.00651463	0.038	3.23E-06	0.010395	22.43767

Vitamin B12

SNP	effect_allel	other_allel	effect_allel	other_allel	beta.exposure	beta.outcome	eaf.exposure	eaf.outcome	pval.outcome	se.outcome	se.exposure	pval.exposure	R2	Fstat
rs1131603	C	T	C	T	0.22	0.007679892	0.06	0.03	0.6571	0.01729705	0.015	4.90E-49	0.004698	215.1111

rs1141321	C	T	C	T	0.07	0.0076305	0.63	0.2347	0.2732	0.00696213	0.007	3.60E-26	0.00219	100
rs1801222	G	A	G	A	0.12	-0.006883398	0.59	0.7644	0.3223	0.00695293	0.007	3.30E-75	0.006407	293.8776
rs2270655	G	C	G	C	0.1	0.005625714	0.94	0.9486	0.6739	0.01336274	0.015	2.20E-13	0.000974	44.44444
rs2336573	T	C	T	C	0.31	-0.001042036	0.03	0.9398	0.9328	0.01240519	0.019	8.40E-59	0.005807	266.205
rs34324219	C	A	C	A	0.23	-0.007422667	0.88	0.0698	0.5215	0.01157982	0.011	1.10E-111	0.009502	437.1901
rs3742801	T	C	T	C	0.05	-0.006387667	0.29	0.7162	0.329	0.00654474	0.007	1.70E-13	0.001118	51.02041
rs41281112	C	T	C	T	0.18	-0.016463842	0.95	0.0322	0.3244	0.01671456	0.015	8.90E-35	0.00315	144
rs602662	A	G	A	G	0.17	-0.006048496	0.6	0.6139	0.3181	0.00606062	0.007	2.40E-139	0.012778	589.7959

Vitamin B6

SNP	effect_allel	other_allel	effect_allel	other_allel	beta.exposure	beta.outcome	eaf.exposure	eaf.outcome	pval.outcome	se.outcome	se.exposure	pval.exposure	R2	Fstat
rs4654748	C	T	C	T	1.45	-0.006097373	0.5	0.4756	0.302	0.00590831	0.281	8.00E-18	0.009	26.62707

Alpha-tocopherol (vitamin E)

SNP	effect_allel	other_allel	effect_allel	other_allel	beta.exposure	beta.outcome	eaf.exposure	eaf.outcome	pval.outcome	se.outcome	se.exposure	pval.exposure	R2	Fstat
rs964184	G	C	G	C	0.04	0.003469376	0.15	0.2498	0.6109	0.00681606	0.01	8.00E-12	0.004232	16

Gamma and beta-tocopherol

SNP	effect_allel	other_allel	effect_allel	other_allel	beta.exposure	beta.outcome	eaf.exposure	eaf.outcome	pval.outcome	se.outcome	se.exposure	pval.exposure	R2	Fstat
rs11705639	A	C	T	C	0.12578066	0.007808588	0.1685	0.762	0.2599	0.00692865	0.0138	5.00E-20	0.014375	83.07485
rs62508088	T	C	T	C	0.16487662	-0.022738011	0.0992	0.948	0.08714	0.01328931	0.0179	1.00E-21	0.014676	84.84223

Magnesium

SNP	effect_allel	other_allel	effect_allel	other_allel	beta.exposure	beta.outcome	eaf.exposure	eaf.outcome	pval.outcome	se.outcome	se.exposure	pval.exposure	R2	Fstat
rs10043693	A	G	A	G	0.03589	-0.006904855	0.6921	0.6461	0.263	0.00617056	0.004433	5.63E-16	0.00045	65.54679
rs1035283	G	A	G	A	-0.06605	0.014420184	0.9368	0.0682	0.2181	0.01170469	0.00826	1.27E-15	0.000439	63.9419
rs10747045	T	G	T	G	-0.02741	-4.45E-05	0.6696	0.6848	0.9947	0.00635103	0.004336	2.57E-10	0.000274	39.96129
rs10952168	G	A	G	A	0.02429	-0.014450147	0.4401	0.6234	0.01766	0.0060894	0.004443	4.57E-08	0.000205	29.88838
rs10974444	G	C	G	C	0.02687	0.013204868	0.6607	0.7094	0.04212	0.00649846	0.004233	2.19E-10	0.000277	40.29385
rs11234579	C	T	C	T	-0.02714	0.004444177	0.6373	0.258	0.51	0.00674382	0.004453	1.10E-09	0.000255	37.14619
rs112510641	G	A	G	A	0.03035	0.003754093	0.6104	0.315	0.5545	0.0063521	0.00418	3.86E-13	0.000362	52.71872
rs113174770	T	G	T	G	0.04893	0.021658729	0.9334	0.9556	0.1305	0.01432456	0.008128	1.74E-09	0.000249	36.23957
rs115478735	A	T	A	T	-0.04194	-0.013282264	0.8064	0.8617	0.1201	0.00854715	0.00508	1.52E-16	0.000468	68.15998
rs11614506	T	C	T	C	0.03278	0.007390866	0.7819	0.7026	0.2521	0.00645491	0.004892	2.09E-11	0.000308	44.89986
rs11694498	T	A	T	A	-0.04061	-0.006739971	0.6086	0.6725	0.2837	0.00628729	0.004166	1.88E-22	0.000652	95.02272
rs117672478	C	A	C	A	-0.08527	0.03548578	0.9763	0.0137	0.1621	0.02538325	0.01506	1.51E-08	0.00022	32.05845
rs12203597	G	A	G	A	-0.03104	-0.001932167	0.3435	0.4749	0.7438	0.00590877	0.004361	1.10E-12	0.000348	50.66071
rs12230212	T	A	T	A	0.1637	-0.016744645	0.989	0.9914	0.6	0.03195543	0.02743	2.39E-09	0.000245	35.61605
rs1229984	T	C	T	C	0.07801	-0.017458158	0.03517	0.06619	0.1413	0.01186822	0.01116	2.79E-12	0.000336	48.8621
rs12464156	T	C	T	C	-0.02627	0.003910011	0.5962	0.7121	0.5487	0.00651668	0.004107	1.58E-10	0.000281	40.91389
rs1273884	G	A	G	A	0.04993	-0.003202243	0.5595	0.3701	0.6003	0.00611115	0.004091	2.87E-34	0.001022	148.9581
rs12743084	C	G	C	G	-0.08845	-0.006039483	0.4615	0.3678	0.3238	0.00611903	0.004065	5.97E-105	0.003242	473.4505
rs12743084	C	G	C	G	-0.08845	-0.006039483	0.4615	0.3678	0.3238	0.00611903	0.004065	5.97E-105	0.003242	473.4505
rs12918968	A	C	A	C	-0.03944	0.016245292	0.5741	0.4865	0.005924	0.00590309	0.004172	3.25E-21	0.000614	89.36865
rs13143189	G	A	G	A	0.02938	0.013924854	0.5727	0.3396	0.02545	0.00623036	0.004194	2.47E-12	0.000337	49.07347
rs13146355	G	A	G	A	-0.06525	-0.004437048	0.5558	0.3776	0.4658	0.00608649	0.004024	3.91E-59	0.001803	262.933
rs13170671	C	T	C	T	0.02378	0.010788619	0.5502	0.4556	0.06866	0.00592456	0.004199	1.48E-08	0.00022	32.07244
rs13193692	G	T	G	T	-0.03149	0.007502856	0.7824	0.2641	0.2624	0.006693	0.005055	4.69E-10	0.000267	38.80637
rs142601087	T	C	T	C	0.02922	0.00025346	0.806	0.8335	0.9748	0.00792062	0.005168	1.57E-08	0.00022	31.968
rs143135527	C	T	C	T	0.08315	0.009418246	0.9719	0.0172	0.678	0.02269457	0.01462	1.28E-08	0.000222	32.34668
rs17794420	G	A	G	A	0.02859	-0.006150051	0.5174	0.3866	0.31	0.00605916	0.004373	6.19E-11	0.000294	42.74343
rs17832417	A	T	A	T	0.03899	0.005458762	0.6265	0.6947	0.3942	0.006407	0.004404	8.47E-19	0.000538	78.38119
rs1853392	A	G	A	G	-0.05664	-0.006840361	0.09745	0.06512	0.5675	0.01195867	0.008654	5.98E-11	0.000294	42.83637
rs1890185	A	G	A	G	-0.02732	-0.002177965	0.5875	0.574	0.7148	0.00596703	0.004063	1.75E-11	0.000311	45.21346
rs219782	A	G	A	G	-0.06371	0.014344389	0.7483	0.7769	0.04292	0.00708715	0.004632	4.75E-43	0.001298	189.1813
rs2439722	T	C	T	C	-0.02569	-0.001853016	0.528	0.4993	0.7532	0.00590132	0.00424	1.36E-09	0.000252	36.71102
rs250383	A	T	A	T	0.05615	-0.007675667	0.8149	0.8013	0.2992	0.00739467	0.005266	1.53E-26	0.00078	113.6941
rs2510467	A	G	A	G	0.03135	-0.001988778	0.4299	0.4614	0.7372	0.00591898	0.004437	1.59E-12	0.000343	49.92249
rs2542713	A	C	A	C	0.02645	0.004705362	0.4504	0.5147	0.4254	0.00590384	0.004186	2.64E-10	0.000274	39.92573
rs2731238	G	A	G	A	0.05395	0.010304746	0.2993	0.125	0.2482	0.00892186	0.004601	9.56E-32	0.000944	137.4923
rs2731238	G	T	T	C	-0.1227	-0.010304746	0.9493	0.875	0.2482	0.00892186	0.009953	6.53E-35	0.001043	151.9781
rs2731238	G	A	G	A	0.05395	0.006853545	0.2993	0.5523	0.2482	0.0059338	0.004601	9.56E-32	0.000944	137.4923
rs2731238	G	T	T	C	-0.1227	-0.006853545	0.9493	0.4477	0.2482	0.0059338	0.009953	6.53E-35	0.001043	151.9781
rs2818759	G	T	G	T	0.03013	0.011849435	0.344	0.7913	0.1027	0.00726068	0.00441	8.39E-12	0.000321	46.67895
rs28441180	G	A	G	A	0.02463	-0.00507988	0.491	0.5324	0.3904	0.00591371	0.004398	2.15E-08	0.000215	31.36306
rs303968	T	C	T	C	-0.03532	0.011522048	0.6028	0.4885	0.05099	0.00590269	0.004198	3.98E-17	0.000486	70.78749
rs34872471	T	C	T	C	0.04058	-0.045411583	0.7066	0.7379	1.28E-11	0.00670678	0.004434	5.56E-20	0.000575	83.75924
rs35249105	A	G	A	G	-0.04637	0.005531233	0.538	0.6131	0.3615	0.00605831	0.004121	2.25E-29	0.000869	126.6103
rs35347302	G	A	G	A	-0.02671	-0.004120658	0.7702	0.1532	0.6147	0.00819216	0.004774	2.20E-08	0.000215	31.30278
rs35465213	G	A	G	A	-0.1494	0.011207386	0.9477	0.0774	0.3102	0.01104176	0.009452	2.85E-56	0.001713	249.8353
rs35934	G	T	G	T	-0.05568	0.006099769	0.1741	0.7781	0.3904	0.00710101	0.005631	4.71E-23	0.000671	97.7749
rs3732215	G	C	G	C	-0.02364	-0.000241956	0.4801	0.4981	0.9671	0.00590137	0.003994	3.26E-09	0.000241	35.03312
rs3783297	T	C	T	C	0.02579	-0.007576258	0.6433	0.6205	0.2129	0.00608046	0.004293	1.89E-09	0.000248	36.0895
rs3824347	A	G	A	G	0.05695	-0.005924452	0.5864	0.6129	0.328	0.00605772	0.004257	8.35E-41	0.001228	178.97
rs3848132	T	A	T	A	-0.04543	0.004392797	0.723	0.762	0.5258	0.0069287	0.004775	1.82E-21	0.000621	90.51879
rs3925584	T	C	T	C	0.07105	-0.003955506	0.5475	0.5829	0.5085	0.00598412	0.00402	6.73E-70	0.002141	312.3748
rs4077450	G	T	G	T	-0.03324	0.00188991	0.2184	0.6255	0.7563	0.00609648	0.005478	1.31E-09	0.000253	36.81951
rs425135	A	C	A	C	-0.03465	0.007288603	0.1466	0.1201	0.4218	0.00907672	0.005797	2.27E-09	0.000245	35.72727
rs4962402	T	G	T	G	-0.03358	0.010672512	0.2763	0.2099	0.1408	0.00724543	0.004637	4.43E-13	0.00036	52.44296
rs544934737	T	C	T	C	0.02519	-0.002868634	0.5678	0.4898	0.6273	0.00590254	0.004601	4.36E-08	0.000206	29.97449
rs560609863	T	G	T	G	-0.05315	0.008150581	0.8518	0.8323	0.3023	0.00789785	0.009142	6.12E-09	0.000232	33.80058
rs58447148	A	G	A	G	0.04638	-0.002895084	0.9139	0.9259	0.7971	0.01126492	0.007637	1.26E-09	0.000253	36.88212
rs58560619	T	C	T	C	0.02335	0.004986252	0.4936	0.6677	0.4259	0.00626414	0.004079	1.04E-08	0.000225	32.76924
rs592859	C	G	C	G	0.03162	-0.005143408	0.1578	0.2076	0.4794	0.00727498	0.005773	4.31E-08	0.000206	29.99996
rs59359435	T	C	T	C	0.06372	0.000921528	0.9553	0.9655	0.9542	0.01616716	0.01039	8.55E-10	0.000258	37.61148
rs59685687	T	A	T	A	0.02874	-0.004044924	0.7714	0.679	0.5224	0.00632019	0.005196	3.16E-08	0.00021	30.59393
rs6003469	A	C	A	C	0.04485	-0.003108148	0.6924	0.7979	0.6726	0.00734787	0.004437	5.11E-24	0.000701	102.1753
rs606970	A	G	A	G	-0.02401	-0.002101667	0.5289	0.5662	0.7241	0.00595373	0.00432	2.74E-08	0.000212	30.88992
rs62136373	C	T	C	T	0.03313	-0.003296598	0.8549	0.1908	0.6607	0.00750934	0.006058	4.51E-08	0.000205	29.90779
rs623297	C	T	C	T	0.02897	-0.004903077	0.7303	0.3107	0.4421	0.00637591	0.004862	2.56E-09	0.000244	35.50317
rs636264	G	A	G	A	-0.03387	0.005243467	0.2082	0.7164	0.423	0.00654615	0.005236	9.93E-11	0.000287	41.8438
rs658903	T	A	T	A	-0.03979	0.007435858	0.855	0.897	0.4439	0.00970739	0.006028	4.07E-11	0.000299	43.57139
rs6667005	T	C	T	C	0.02947	-0.004930875	0.7844	0.7411	0.4643	0.00673617	0.004962	2.86E-09	0.000242	35.27335
rs6936263	G	C	G	C	-0.02746	-0.005478663	0.2853	0.4186	0.3596	0.00598107	0.004564	1.78E-09	0.000249	36.2001
rs6936263	G	C	G	C	-0.02746	0.005408626	0.2853	0.4832	0.3596	0.00590461	0.004564	1.78E-09	0.000249	36.2001
rs7039	C	G	C	G	0.03185	0.007416076	0.479	0.4349	0.2126	0.00595191	0.004072	5.26E-15	0.00042	61.17914
rs7077696	A	G	A	G	0.02798	-0.005137486	0.7797	0.7418	0.446	0.00674211	0.00501	2.34E-08	0.000214	31.19033
rs711819	T	C	T	C	-0.03593	0.001275324	0.2421	0.1771	0.8693	0.00772923	0.004965	4.61E-13	0.00036	52.3692
rs7221296	A	G	A	G	-0.04431	0.004684036	0.8126	0.8266	0.5476	0.00779374	0.005215	1.94E-17	0.000496	72.19296

rs7241576	A	G	A	G	0.02973	0.001973559	0.6835	0.7909	0.7858	0.00725573	0.004521	4.82E-11	0.000297	43.2435
rs72848405	T	C	T	C	0.02757	0.007140285	0.8042	0.8328	0.3665	0.00790729	0.005049	4.74E-08	0.000205	29.81692
rs7374260	T	C	T	C	0.02461	0.005064484	0.5278	0.5108	0.3908	0.00590266	0.003992	7.04E-10	0.000261	38.00512
rs73949333	A	G	A	G	0.03287	0.001916285	0.776	0.7479	0.778	0.00679534	0.005211	2.84E-10	0.000273	39.78847
rs7416991	T	G	A	G	-0.05192	0.015977807	0.6967	0.6269	0.008824	0.00610073	0.004371	1.51E-32	0.000968	141.0937
rs7416991	T	C	T	C	0.05162	0.015977807	0.3162	0.6269	0.008824	0.00610073	0.004405	1.04E-31	0.000942	137.3233
rs7416991	T	G	A	G	-0.05192	0.01578662	0.6967	0.398	0.008824	0.00602773	0.004371	1.51E-32	0.000968	141.0937
rs7416991	T	C	T	C	0.05162	0.01578662	0.3162	0.398	0.008824	0.00602773	0.004405	1.04E-31	0.000942	137.3233
rs7742789	C	T	C	T	0.05486	-0.009316991	0.6933	0.3884	0.1239	0.00605393	0.004413	1.76E-35	0.001061	154.541
rs77759957	A	G	A	G	0.0746	0.001101149	0.9728	0.9859	0.9651	0.02502611	0.01319	1.54E-08	0.00022	31.98807
rs7797740	A	G	A	G	-0.03551	-0.007999097	0.1499	0.08953	0.4389	0.01033475	0.006093	5.61E-09	0.000233	33.96558
rs78433554	C	A	C	A	0.1049	0.048815774	0.986	0.0044	0.2735	0.04458062	0.0192	4.69E-08	0.000205	29.85029
rs7850067	A	G	A	G	0.04308	0.005807479	0.485	0.5237	0.3258	0.00590791	0.004081	4.79E-26	0.000765	111.4341
rs7867868	C	T	C	T	-0.04157	0.00208227	0.6695	0.2586	0.757	0.00673874	0.004504	2.69E-20	0.000585	85.18503
rs7894336	C	T	C	T	0.06273	0.000641478	0.4679	0.5881	0.915	0.00599512	0.004011	4.00E-55	0.001677	244.5937
rs7946549	T	A	T	A	0.04303	-0.009864262	0.5441	0.5408	0.09577	0.00592093	0.004036	1.52E-26	0.00078	113.6686
rs7946549	T	A	T	A	0.04303	-0.009864262	0.5441	0.5408	0.09577	0.00592093	0.004036	1.52E-26	0.00078	113.6686
rs8068318	C	T	C	T	-0.08407	-0.000526451	0.3015	0.4658	0.9293	0.00591518	0.004691	8.01E-72	0.002202	321.182
rs9897596	T	C	T	C	-0.02319	-0.003802614	0.4911	0.605	0.529	0.00603589	0.004061	1.13E-08	0.000224	32.60885

Potassium

SNP	effect_allel	other_allel	effect_allel	other_allel	beta.exposure	beta.outcome	eaf.exposure	eaf.outcome	pval.outcome	se.outcome	se.exposure	pval.exposure	R2	Fstat
rs10032927	A	T	A	T	0.01561	0.01065914	0.5837	0.5344	0.0715	0.00591517	0.002494	4.01E-10	6.49E-05	39.17535
rs10032927	A	T	A	T	0.01561	0.01065914	0.5837	0.5344	0.0715	0.00591517	0.002494	4.01E-10	6.49E-05	39.17535
rs10046175	C	A	C	A	0.03717	0.009770177	0.9659	0.0184	0.6562	0.02195545	0.006432	7.72E-09	5.59E-05	33.39589
rs10086982	G	A	G	A	-0.02034	0.00202734	0.7603	0.259	0.7632	0.00673535	0.003195	2.01E-10	6.71E-05	40.52847
rs10227075	T	C	T	C	-0.06498	-0.006196235	0.9796	0.9898	0.8326	0.02936604	0.00832	6.01E-15	0.000102	60.9976
rs10264133	C	T	C	T	-0.05002	-0.026168601	0.9737	0.014	0.2973	0.02511382	0.007193	3.67E-12	8.09E-05	48.35788
rs10265221	T	C	T	C	-0.03723	-0.002657683	0.7481	0.8048	0.7214	0.00744449	0.002886	5.03E-38	0.000276	166.4154
rs10270510	G	C	G	C	-0.06498	0.006196235	0.9796	0.9898	0.8326	0.02936604	0.00832	6.01E-15	0.000102	60.9976
rs10279895	A	G	A	G	-0.06565	-0.006344557	0.9797	0.9899	0.8295	0.02950957	0.008331	3.44E-15	0.000104	62.09765
rs10502917	T	C	T	C	-0.02136	-0.000204121	0.3194	0.3102	0.9746	0.00637877	0.002766	1.20E-14	9.88E-05	59.63458
rs10748853	T	C	T	C	-0.03616	0.001448186	0.1179	0.08935	0.8886	0.01034418	0.003902	2.01E-20	0.000142	85.87808
rs1076485	C	T	C	T	0.02448	0.001435454	0.815	0.2442	0.8345	0.0068682	0.00308	2.01E-15	0.000105	63.17153
rs10811662	G	A	G	A	0.01906	0.034020189	0.8401	0.1455	4.78E-05	0.00836699	0.003305	8.20E-09	5.51E-05	33.25852
rs11124938	C	A	C	A	-0.02548	0.002168534	0.7812	0.1699	0.7828	0.00785701	0.002987	1.57E-17	0.000121	72.76598
rs111375249	G	T	G	T	-0.05498	0.000652577	0.9749	0.0175	0.9768	0.02250267	0.007261	3.84E-14	9.60E-05	57.33457
rs111607733	C	A	C	A	-0.05535	-0.001615732	0.9726	0.0181	0.9422	0.02213331	0.006865	7.85E-16	0.000109	65.00611
rs111724190	T	C	T	C	-0.03147	0.006587264	0.9503	0.9811	0.7615	0.02166863	0.005554	1.49E-08	5.37E-05	32.10567
rs111972532	T	C	T	C	0.03879	0.017371084	0.8139	0.8645	0.04392	0.00862089	0.003086	3.39E-36	0.000262	157.9966
rs11217192	T	G	T	G	-0.01709	-0.004736407	0.7648	0.8074	0.5267	0.00748248	0.002949	7.04E-09	5.56E-05	33.58417
rs11245343	T	C	T	C	-0.02401	-0.004524933	0.7283	0.6872	0.4771	0.00636418	0.002872	6.53E-17	0.000116	69.89007
rs11264363	G	C	G	C	0.0252	-0.013886245	0.4285	0.4285	0.01986	0.00596232	0.002659	2.78E-21	0.000149	89.81821
rs112734474	T	G	T	G	-0.07519	-0.01038191	0.9782	0.9863	0.6828	0.02538364	0.008227	6.70E-20	0.00014	83.52898
rs114048605	C	T	C	T	-0.1319	-0.012112212	0.9894	0.0018	0.8616	0.06961042	0.02131	6.28E-10	0.000218	38.31093
rs114323080	G	A	G	A	-0.1018	-0.048039655	0.9864	0.0019	0.4782	0.06775692	0.01812	1.99E-08	0.000179	31.56306
rs115080005	C	T	C	T	0.03996	0.014324685	0.9722	0.0131	0.5808	0.02595052	0.00691	7.55E-09	5.60E-05	33.4422
rs115223381	T	C	T	C	-0.1006	-0.040485374	0.9865	0.998	0.5401	0.06604466	0.01814	3.03E-08	0.000175	30.7554
rs11563587	T	A	T	A	-0.05898	0.028030299	0.9824	0.9916	0.3859	0.03233022	0.00864	9.02E-12	7.80E-05	46.59959
rs11563956	C	T	C	T	-0.03679	-0.029300551	0.9677	0.0183	0.1833	0.02201394	0.006511	1.64E-08	5.34E-05	31.92745
rs11563967	A	G	A	G	-0.03819	-0.036859943	0.9514	0.974	0.04681	0.01854122	0.005682	1.85E-11	7.56E-05	45.17486
rs11563979	G	A	G	A	-0.04055	-0.041531186	0.9527	0.0248	0.02859	0.01897268	0.00576	1.98E-12	8.29E-05	49.56062
rs11563980	C	T	C	T	-0.03382	-0.021090665	0.9393	0.0371	0.1767	0.01561115	0.005234	1.07E-10	6.99E-05	41.75224
rs11563986	T	C	T	C	-0.04416	-0.040487419	0.9695	0.9835	0.08055	0.02316214	0.006736	5.74E-11	7.19E-05	42.97877
rs11563990	C	T	C	T	-0.03834	-0.049923575	0.9635	0.0213	0.01457	0.02043536	0.006325	1.38E-09	6.15E-05	36.74372
rs11564010	G	C	G	C	-0.06314	0.008557772	0.9796	0.9899	0.7715	0.02950956	0.008287	2.66E-14	9.72E-05	58.05164
rs11564019	G	A	G	A	-0.06503	-0.006315048	0.9796	0.0101	0.8302	0.02950957	0.008322	5.79E-15	0.000102	61.06215
rs11564024	T	G	T	G	-0.0652	-0.005535039	0.9299	0.9893	0.8468	0.02867896	0.008369	6.98E-15	0.000345	60.69432
rs11564025	T	G	T	G	-0.06489	-0.005348105	0.9795	0.9897	0.8546	0.02922462	0.008315	6.31E-15	0.000102	60.90193
rs11564194	C	T	C	T	-0.02113	-0.00536563	0.8063	0.1638	0.5008	0.0079727	0.003081	7.27E-12	7.79E-05	47.0344
rs116232574	A	G	A	G	-0.1002	-0.050478881	0.9863	0.9981	0.4565	0.06775689	0.01806	2.96E-08	0.000175	30.78222
rs116355131	C	A	C	A	0.06558	-0.014291251	0.9709	0.0038	0.7657	0.04795722	0.01191	3.76E-08	0.000172	30.31931
rs11711982	T	C	T	C	0.0239	0.022201315	0.883	0.9263	0.04934	0.01129263	0.003863	6.33E-10	6.34E-05	38.27775
rs11743019	A	G	A	G	-0.02052	-0.005605828	0.7361	0.6503	0.3648	0.00618745	0.002981	6.04E-12	7.85E-05	47.3839
rs11822294	C	T	C	T	-0.01797	-0.005084898	0.8251	0.123	0.5714	0.00898392	0.003169	1.47E-08	5.33E-05	32.15523
rs11970777	C	T	C	T	-0.03918	-0.039266061	0.9638	0.0186	0.0721	0.02183874	0.006303	5.25E-10	6.40E-05	38.63974
rs11974568	T	C	T	C	-0.03409	-0.018241289	0.9488	0.9725	0.3118	0.01804282	0.005567	9.44E-10	6.21E-05	37.4983
rs11983963	T	A	T	A	-0.04007	0.039162904	0.9647	0.9822	0.07932	0.02231504	0.00636	3.08E-10	6.57E-05	39.69396
rs1214759	A	G	A	G	-0.01681	0.009962483	0.4192	0.4139	0.09633	0.00599067	0.002573	6.59E-11	7.07E-05	42.68309
rs12378991	G	A	G	A	-0.03902	-0.018561881	0.9411	0.06	0.1351	0.01242428	0.005503	1.39E-12	8.33E-05	50.27771
rs12509595	T	C	T	C	0.03557	-0.013752525	0.7552	0.7501	0.04358	0.00681493	0.002892	9.95E-35	0.00025	151.2764
rs12549572	T	C	T	C	0.02422	0.006952758	0.6728	0.5987	0.248	0.0060197	0.002648	6.11E-20	0.000139	83.65893
rs1273886	A	G	A	G	0.02013	-0.008233238	0.6749	0.7162	0.2084	0.0065447	0.002776	4.30E-13	8.71E-05	52.5834
rs1275979	T	C	T	C	-0.03297	-0.00075413	0.4719	0.3512	0.9029	0.0061814	0.002568	1.09E-37	0.000273	164.8344
rs12766550	C	G	C	G	0.02881	0.01429055	0.9417	0.9564	0.3227	0.01444949	0.005162	2.44E-08	5.16E-05	31.14946
rs12803281	C	T	C	T	-0.01661	0.01072789	0.5864	0.4505	0.07042	0.00593029	0.002465	1.66E-11	7.52E-05	45.40518
rs12940197	G	T	G	T	-0.02288	-0.000520736	0.4361	0.4631	0.9297	0.00591746	0.002976	1.55E-14	9.79E-05	59.10799
rs12959198	T	C	T	C	-0.02103	-0.000504582	0.7725	0.7786	0.9436	0.00710679	0.002958	1.22E-12	8.37E-05	50.54547
rs12975656	G	A	G	A	0.02031	0.006125229	0.5826	0.3868	0.3121	0.00605858	0.0027	5.53E-14	9.37E-05	56.58383
rs13042148	C	T	C	T	-0.02296	-0.014746057	0.8834	0.0948	0.1433	0.01007244	0.004082	1.91E-08	5.24E-05	31.63718
rs13072118	C	T	C	T	0.0161	-0.011442145	0.4291	0.532	0.05299	0.00591325	0.002608	6.93E-10	6.31E-05	38.10979
rs13072590	G	A	G	A	0.01745	-0.007556222	0.786	0.1957	0.3095	0.00743723	0.003135	2.69E-08	5.13E-05	30.98245
rs13333693	A	G	A	G	0.02753	0.007248651	0.7554	0.7477	0.286	0.00679349	0.00284	3.46E-22	0.000156	93.96708
rs1336173	T	A	T	A	0.04684	0.015239488	0.9711	0.9837	0.5128	0.02330197	0.007667	1.03E-09	6.25E-05	37.32354
rs138278364	C	A	C	A	-0.0861	-0.096357683	0.981	0.002	0.1447	0.06604365	0.01445	2.63E-09	0.000202	35.50345
rs138304526	T	C	T	C	0.1353	0.152901828	0.9882	0.9978	0.01517	0.06297439	0.02115	1.63E-10	0.000233	40.9237
rs138404919	C	T	C	T	-0.1012	-0.048784975	0.9865	0.0019	0.4713	0.06775691	0.0181	2.29E-08	0.000178	31.26107
rs138531692	G	A	G	A	-0.0861	-0.096357683	0.981	0.002	0.1447	0.06604365	0.01445	2.63E-09	0.000202	35.50345
rs139145497	T	C	T	C	-0.086	-0.093319783	0.9811	0.998	0.1576	0.06604372	0.01441	2.46E-09	0.000202	35.61795
rs139316656	G	A	G	A	-0.0861	-0.096357683	0.981	0.002	0.1447	0.06604365	0.01445	2.63E-09	0.000202	35.50345
rs139777921	T	A	T	A	-0.08715	0.077475577	0.9828	0.9982	0.2657	0.06960968	0.01523	1.09E-08	0.000186	32.74424
rs140318611	G	A	G	A	-0.08728	-0.079860963	0.9829	0.0017	0.265	0.07162418	0.01523	1.03E-08	0.000187	32.84201
rs140501563	G	A	G	A	-0.08845	-0.081781921	0.982	0.0019	0.2273	0.06775636	0.01484	2.61E-09	0.000202	35.52449
rs141016776	C	T	C	T	-0.08603	-0.092263119	0.981	0.002	0.1624	0.06604375	0.01438	2.23E-09	0.000203	35.79168
rs141017725	G	A	G	A	-0.08644	-0.095739281	0.9811	0.0019	0.1576	0.06775604	0.01439	1.93E-09	0.000205	36.08344
rs141935404	C	T	C	T	-0.02551	0.015035842	0.9136	0.0583	0.2325	0.01259283	0.004305	3.21E-09	5.82E-05	35.11354
rs142365336	G	T	G	T	0.1224	0.142217697	0.986	0.0028	0.01087	0.05583734	0.01888	9.41E-11	0.000239	42.02995
rs1427298	C	T	C	T	-0.01837	-0.008806325	0.6201	0.4792	0.136	0.00590632	0.002624	2.67E-12	8.12E-05	49.01067
rs142878005	T	C	T	C	-0.08632	-0.0931877	0.9811	0.998	0.1581	0.06604373	0.01439	2.03E-09	0.000204	35.98332

rs142887200	C	T	C	T	-0.06546	-0.005432718	0.9795	0.0102	0.853	0.02936604	0.008327	3.98E-15	0.000103	61.79806
rs143341259	T	C	T	C	0.04981	0.015572688	0.9743	0.9609	0.3061	0.01522257	0.008639	8.33E-09	5.56E-05	33.24345
rs143862634	T	G	T	G	-0.08676	-0.074621638	0.9828	0.9982	0.2838	0.06960974	0.01513	9.98E-09	0.000187	32.88223
rs144261869	A	G	A	G	0.03665	0.011312761	0.9654	0.9814	0.6048	0.02183931	0.006401	1.06E-08	5.49E-05	32.78327
rs144726326	C	T	C	T	-0.0398	-0.040010327	0.9534	0.0243	0.0368	0.01916203	0.005839	9.65E-12	7.78E-05	46.46107
rs144808502	G	A	G	A	-0.08486	-0.090454478	0.9817	0.0019	0.1818	0.06775616	0.01482	1.06E-08	0.000186	32.7876
rs144843179	G	A	G	A	0.03096	0.016820943	0.7948	0.1393	0.04841	0.00852125	0.002895	1.15E-26	0.000189	114.3681
rs145391254	C	T	C	T	0.03637	0.019875499	0.9666	0.0191	0.3563	0.02155694	0.006427	1.57E-08	5.36E-05	32.02359
rs145557956	A	G	A	G	-0.08581	-0.08981958	0.9809	0.998	0.1737	0.06604381	0.0143	2.02E-09	0.000205	36.00839
rs146176062	C	T	C	T	-0.08591	-0.092131036	0.9809	0.002	0.163	0.06604375	0.01438	2.35E-09	0.000203	35.6919
rs146416293	C	G	C	G	-0.0861	-0.096357683	0.981	0.998	0.1447	0.06604365	0.01445	2.63E-09	0.000202	35.50345
rs146745107	T	C	T	C	-0.08633	-0.092527285	0.981	0.998	0.1613	0.06604374	0.0144	2.07E-09	0.000204	35.94169
rs147888747	A	C	A	C	-0.08684	-0.080607941	0.9828	0.9982	0.247	0.06960962	0.01531	1.45E-08	0.000183	32.17283
rs148160104	A	G	A	G	-0.0861	-0.096357683	0.981	0.998	0.1447	0.06604365	0.01445	2.63E-09	0.000202	35.50345
rs148227305	G	A	G	A	-0.08581	-0.08981958	0.9809	0.002	0.1737	0.06604381	0.0143	2.02E-09	0.000205	36.00839
rs148768978	G	A	G	A	-0.08633	-0.092527285	0.981	0.002	0.1613	0.06604374	0.0144	2.07E-09	0.000204	35.94169
rs149474053	T	C	T	C	0.1332	0.13846266	0.9887	0.9977	0.02458	0.06159371	0.02144	5.38E-10	0.000219	38.59747
rs149674884	G	A	G	A	0.0641	0.047016398	0.9555	0.0082	0.1508	0.03271844	0.01031	5.19E-10	0.00022	38.65438
rs149727168	A	T	A	T	-0.08581	-0.08981958	0.9809	0.998	0.1737	0.06604381	0.0143	2.02E-09	0.000205	36.00839
rs149923520	C	T	C	T	-0.0861	-0.096357683	0.981	0.002	0.1447	0.06604365	0.01445	2.63E-09	0.000202	35.50345
rs149925191	G	T	G	T	0.03863	0.014417266	0.9729	0.0126	0.5858	0.0264537	0.006972	3.08E-08	5.14E-05	30.69974
rs1502282	G	C	G	C	-0.01458	3.68E-05	0.5896	0.6366	0.9953	0.0061347	0.0025	5.62E-09	5.63E-05	34.01222
rs150305470	C	G	C	G	-0.08544	-0.092510844	0.9818	0.9982	0.1839	0.06960936	0.01476	7.23E-09	0.00019	33.5081
rs150464897	G	A	G	A	-0.08522	-0.091074371	0.981	0.002	0.1679	0.06604378	0.01436	2.99E-09	0.0002	35.21877
rs150589717	G	A	G	A	-0.1405	-0.050570364	0.9888	0.0023	0.4114	0.06159606	0.02043	6.45E-12	0.000269	47.29507
rs1529897	T	G	T	A	-0.01533	-0.002100247	0.5376	0.6688	0.7378	0.0062694	0.002553	1.97E-09	5.97E-05	36.05643
rs1529897	T	G	T	A	-0.01533	-0.028551825	0.5376	0.9988	0.7378	0.08522933	0.002553	1.97E-09	5.97E-05	36.05643
rs1551287	C	T	C	T	0.02749	0.008822228	0.2156	0.8386	0.2713	0.00802021	0.00299	4.04E-20	0.00014	84.52927
rs1574817	T	G	T	G	0.01733	-0.006292716	0.3903	0.2913	0.3326	0.00649403	0.002612	3.35E-11	7.37E-05	44.02008
rs1609829	T	C	T	C	-0.01753	-0.000199179	0.293	0.2709	0.9764	0.00663929	0.002702	9.06E-11	6.97E-05	42.09138
rs164746	G	A	G	A	-0.02005	0.004486223	0.6178	0.356	0.4665	0.00616239	0.002597	1.22E-14	9.87E-05	59.6053
rs17038613	C	T	C	T	0.1229	0.140542763	0.9859	0.0028	0.01183	0.05583741	0.01885	7.24E-11	0.000242	42.50902
rs17038648	C	T	C	T	0.1127	0.141765131	0.9767	0.0045	0.001299	0.0440812	0.01432	3.74E-15	0.000352	61.93866
rs17314270	T	C	T	C	0.1032	0.125397941	0.9746	0.9956	0.004903	0.04457801	0.01385	9.40E-14	0.000315	55.52133
rs17367435	T	C	T	C	-0.01507	-0.000208243	0.5555	0.5637	0.9723	0.00594981	0.002667	1.63E-08	5.29E-05	31.92864
rs17390839	G	A	G	A	0.02233	-0.00057416	0.837	0.1815	0.9406	0.00765547	0.003495	1.73E-10	6.76E-05	40.82095
rs17472490	G	A	G	A	-0.04054	-0.02572026	0.9654	0.0175	0.253	0.02250242	0.006389	2.28E-10	6.74E-05	40.26259
rs17472728	G	T	G	T	0.05347	0.00399369	0.9855	0.0074	0.9078	0.03442836	0.009379	1.22E-08	5.44E-05	32.50179
rs17472899	A	G	A	G	-0.05945	-0.029129511	0.9824	0.9916	0.3676	0.0323302	0.008667	7.20E-12	7.87E-05	47.0507
rs17473032	T	C	T	C	-0.06074	-0.031715886	0.9824	0.9916	0.3266	0.03233016	0.008775	4.65E-12	8.02E-05	47.91322
rs17473487	C	T	C	T	-0.06575	-0.005276928	0.9303	0.0107	0.8541	0.02867896	0.008394	4.99E-15	0.000349	61.35551
rs17473690	G	T	G	T	-0.0628	-0.012167174	0.9811	0.0095	0.689	0.03041794	0.008636	3.70E-13	8.85E-05	52.88031
rs17473844	T	C	T	C	-0.04674	-0.049688699	0.9708	0.985	0.04062	0.02427391	0.006952	1.85E-11	7.57E-05	45.20202
rs17473985	G	A	G	A	-0.03361	-0.019080325	0.9494	0.0271	0.2937	0.01817174	0.005585	1.83E-09	6.00E-05	36.21518
rs17501084	A	G	A	G	-0.05527	-0.020334599	0.9818	0.9913	0.5221	0.03177281	0.008478	7.30E-11	7.11E-05	42.50031
rs17501559	A	G	A	G	0.05322	0.004544543	0.9853	0.9926	0.8946	0.03442836	0.009367	1.37E-08	5.40E-05	32.28113
rs17502552	G	T	G	T	-0.06301	-0.009147962	0.9796	0.0101	0.7568	0.02950956	0.008296	3.21E-14	9.65E-05	57.68747
rs17502782	C	A	C	A	-0.0337	-0.017498258	0.9507	0.0267	0.3391	0.01830362	0.005628	2.18E-09	5.94E-05	35.85516
rs17502922	C	G	C	G	0.06535	-0.013634141	0.9708	0.9961	0.7737	0.04734077	0.0119	4.07E-08	0.000171	30.15763
rs17502970	C	A	C	A	-0.04408	-0.040811672	0.9695	0.0165	0.07813	0.02316213	0.006699	4.86E-11	7.25E-05	43.29754
rs1757225	A	G	A	G	-0.02184	-0.007962171	0.3802	0.3271	0.2055	0.00628923	0.002547	1.05E-17	0.000122	73.52709
rs185372147	T	G	T	G	-0.06575	-0.005276928	0.9303	0.9893	0.8541	0.02867896	0.008394	4.99E-15	0.000349	61.35551
rs190945315	A	C	A	C	-0.06543	-0.001056251	0.9302	0.9892	0.9704	0.02854733	0.008378	6.02E-15	0.000347	60.99204
rs191079	T	C	T	C	0.01581	0.007240921	0.5793	0.4395	0.2234	0.00594493	0.002839	2.62E-08	5.14E-05	31.01223
rs191102591	G	T	G	T	-0.04406	-0.042780339	0.9695	0.0165	0.06474	0.02316207	0.006699	4.98E-11	7.24E-05	43.25826
rs1979845	C	T	C	T	0.03115	-0.002678108	0.8267	0.1499	0.7462	0.00826577	0.003306	4.62E-21	0.000147	88.77903
rs1984669	C	T	C	T	-0.0221	0.015015425	0.7592	0.3226	0.01734	0.00631165	0.002834	6.66E-15	0.000101	60.81138
rs1989061	T	C	T	C	0.01826	0.001196202	0.3219	0.2608	0.8585	0.00672024	0.002709	1.62E-11	7.52E-05	45.43427
rs199680901	C	T	C	T	-0.01837	0.00192319	0.6385	0.384	0.7514	0.00606685	0.002631	3.01E-12	8.07E-05	48.75022
rs1997596	C	T	C	T	-0.01956	0.002660122	0.666	0.3465	0.6677	0.00620075	0.002733	8.62E-13	8.48E-05	51.22222
rs2050542	T	C	T	C	0.04403	-0.003921616	0.9731	0.9891	0.8903	0.02841751	0.007712	1.16E-08	5.46E-05	32.59593
rs210139	A	C	A	C	-0.01598	0.005889723	0.5911	0.5502	0.3206	0.00593124	0.0024	2.89E-11	7.34E-05	44.3334
rs2168785	C	T	C	T	0.01863	0.001504514	0.3675	0.6631	0.8092	0.0062428	0.002684	4.09E-12	7.98E-05	48.17932
rs2195880	C	G	C	G	0.01569	0.006104577	0.7004	0.6826	0.3354	0.00633912	0.002629	2.46E-09	5.90E-05	35.61761
rs219791	T	G	T	G	0.0173	0.008590955	0.6668	0.7407	0.2019	0.00673272	0.002688	1.26E-10	6.86E-05	41.42225
rs2207404	G	A	G	A	-0.0484	0.001766928	0.9636	0.0226	0.9294	0.01985313	0.006281	1.35E-14	9.83E-05	59.37904
rs2255390	A	G	A	G	0.02748	0.003334761	0.4329	0.4268	0.5761	0.00596558	0.002426	1.05E-29	0.000212	128.3074
rs2327429	T	C	T	C	-0.02284	-0.008234008	0.737	0.6236	0.1765	0.00609024	0.002788	2.65E-16	0.000111	67.113
rs2340534	G	A	G	A	0.01826	0.007561518	0.5376	0.5361	0.2014	0.00591668	0.002458	1.15E-13	9.14E-05	55.18713
rs2497318	C	T	C	T	-0.02938	-0.015923759	0.6293	0.3375	0.01071	0.00623972	0.002633	6.77E-29	0.000206	124.5093
rs2569882	T	C	T	C	-0.01474	-0.001398645	0.5253	0.5034	0.8126	0.00590146	0.002462	2.21E-09	5.94E-05	35.8442
rs2761244	T	C	T	C	0.01525	-0.006779028	0.3969	0.3851	0.2634	0.00606353	0.002484	8.53E-10	6.24E-05	37.6909
rs2823263	T	C	T	C	-0.02564	-0.002603391	0.7425	0.7501	0.7021	0.00681516	0.002846	2.17E-19	0.000134	81.16452
rs28398484	C	G	C	G	-0.04176	-0.040661171	0.9686	0.9825	0.07074	0.02250203	0.006668	3.89E-10	6.56E-05	39.22201
rs28398495	A	G	A	G	-0.03906	-0.036653479	0.9512	0.9744	0.04981	0.01868169	0.005686	6.69E-12	7.90E-05	47.19009
rs28398500	C	G	C	G	-0.0384	-0.036211044	0.9513	0.974	0.05085	0.01854124	0.005685	1.49E-11	7.64E-05	45.62486
rs28398507	G	A	G	A	-0.03811	-0.036563304	0.9515	0.026	0.04862	0.01854123	0.005688	2.15E-11	7.51E-05	44.89094
rs28398535	T	C	T	C	-0.03384	-0.030863483	0.9598	0.977	0.1169	0.01968334	0.005987	1.62E-08	5.35E-05	31.94789
rs28398544	A	G	A	G	-0.03329	-0.028321839	0.9595	0.9767	0.1476	0.01955928	0.005932	2.04E-08	5.27E-05	31.49382
rs28416181	T	G	T	G	0.02165	0.002085921	0.71	0.6595	0.7378	0.00622663	0.002647	3.04E-16	0.000111	66.8972
rs28430881	C	A	C	A	-0.02156	0.002547624	0.5611	0.4001	0.6723	0.00602275	0.002429	7.34E-19	0.00013	78.78481
rs28446035	C	T	C	T	0.03696	0.008870006	0.9657	0.0184	0.6863	0.02195546	0.006433	9.40E-09	5.52E-05	33.00934
rs28558845	G	C	G	C	-0.02063	-0.011099915	0.82	0.8676	0.2022	0.00870582	0.003066	1.76E-11	7.50E-05	45.27455
rs28570591	G	A	G	A	-0.06608	-0.004101831	0.9798	0.0101	0.8891	0.02950957	0.008333	2.30E-15	0.000105	62.88359
rs3176466	C	T	C	T	0.04031	0.02048725	0.8668	0.0839	0.05419	0.01064273	0.003543	5.89E-30	0.000214	129.4444
rs34070447	A	G	A	G	0.02013	-0.001216071	0.5474	0.5128	0.8368	0.00590326	0.00238	2.89E-17	0.000118	71.53748
rs34136790	C	T	C	T	0.02587	-0.005153538	0.8161	0.1066	0.5902	0.00956129	0.004021	1.29E-10	6.93E-05	41.39279
rs34917191	T	C	T	C	-0.01332	-0.005925875	0.4203	0.347	0.3391	0.00619861	0.002409	3.32E-08	5.06E-05	30.57277
rs35444	A	G	A	G	-0.0336	0.000551458	0.6131	0.6524	0.9288	0.00619616	0.00241	3.97E-44	0.000322	194.3768
rs35619052	G	A	G	A	0.02036	0.00119242	0.7815	0.1763	0.8775	0.00774299	0.003609	1.73E-08	5.33E-05	31.82598
rs35619990	C	T	C	T	-0.01762	-0.006547195	0.5513	0.5858	0.2746	0.00599011	0.002433	4.59E-13	8.69E-05	52.44784
rs35812759	A	G	A	G	0.01552	0.003355237	0.7522	0.8056	0.6524	0.00745608	0.002755	1.79E-08	5.26E-05	31.73513
rs36092406	G	A	G	A	-0.04798	0.000496328	0.9635	0.0226	0.9802	0.01985313	0.006277	2.19E-14	9.68E-05	58.42736
rs3751767	T	C	T	C	-0.01723	-0.005986607	0.6902	0.6464	0.332	0.00617176	0.002891	2.59E-09	5.88E-05	35.52012
rs3790604	C	A	C	A	0.04697	-0.004545519	0.9322	0.1253	0.6103	0.00891278	0.004789	1.12E-22	0.000159	96.19477
rs3802177	G	A	G	A	0.01547	0.017271813	0.7385	0.2658	0.009715	0.00667897	0.00278	2.70E-08	5.13E-05	30.96642
rs3848132	T	A	T	A	-0.03504	0.004392797	0.7463	0.762	0.5258	0.0069287	0.002922	4.26E-33	0.000238	143.8029
rs4114858	A	G	A	G	0.01947	-0.001924062	0.8371	0.8863	0.8358	0.00929498	0.003459	1.87E-08	5.25E-05	31.68333
rs4413681	G	C	G	C	0.06552	0.012372964	0.9709	0.9962	0.7967	0.04795723	0.01196	4.44E-08	0.000171	30.01134

rs4461961	C	T	C	T	0.01818	-0.000756318	0.6471	0.2793	0.9087	0.00657668	0.002611	3.43E-12	8.03E-05	48.48127
rs4485922	A	G	A	G	-0.01442	-0.004424101	0.46	0.4286	0.4583	0.0059624	0.002442	3.56E-09	5.78E-05	34.86898
rs4677143	A	G	A	G	0.01596	-0.006556059	0.6379	0.6408	0.2865	0.00615015	0.002609	9.70E-10	6.20E-05	37.42119
rs4737371	G	A	G	A	-0.02244	0.000213652	0.8207	0.1669	0.9782	0.00791303	0.003276	7.74E-12	7.77E-05	46.91999
rs4745804	T	C	T	C	-0.01661	-0.008004794	0.5313	0.6001	0.1838	0.00602317	0.002577	1.20E-10	6.88E-05	41.5442
rs4766578	T	A	T	A	0.01805	-0.006068242	0.3993	0.3134	0.3403	0.00636084	0.002668	1.39E-11	7.58E-05	45.77019
rs4809849	C	T	C	T	0.01779	-0.003687901	0.4172	0.5794	0.5371	0.00597715	0.002675	3.03E-11	7.33E-05	44.22872
rs4846476	G	C	G	C	0.01665	-0.014446627	0.7784	0.7539	0.03491	0.00684999	0.002851	5.38E-09	5.65E-05	34.10626
rs4848713	C	T	C	T	0.02442	0.00662337	0.8902	0.2395	0.3382	0.00691375	0.004187	5.65E-09	5.63E-05	34.01617
rs4867732	C	G	C	G	0.02908	0.001565936	0.9196	0.7211	0.8122	0.00657956	0.004584	2.30E-10	6.67E-05	40.24386
rs4924538	A	T	A	T	-0.01606	0.00174106	0.5128	0.507	0.768	0.0059019	0.002619	8.81E-10	6.23E-05	37.60279
rs4936409	A	G	A	G	-0.01693	-0.00548145	0.491	0.5649	0.3569	0.00595163	0.002558	3.75E-11	7.25E-05	43.80391
rs547340372	G	A	G	A	-0.05554	-0.002822242	0.976	0.0122	0.9162	0.02687849	0.007827	1.33E-12	8.43E-05	50.35251
rs55650455	T	C	T	C	-0.01947	-0.015277954	0.8204	0.8478	0.06283	0.00821395	0.003288	3.26E-09	5.81E-05	35.06455
rs56218647	C	A	C	A	0.02388	0.010010421	0.9123	0.1159	0.2774	0.0092177	0.004155	9.36E-09	5.47E-05	33.03137
rs56237637	T	A	T	A	0.04136	0.023383514	0.9645	0.9792	0.2579	0.02067508	0.007107	6.08E-09	5.67E-05	33.86791
rs56329049	G	A	G	A	-0.04065	-0.029130973	0.9643	0.0215	0.1522	0.02034286	0.006417	2.45E-10	6.65E-05	40.12888
rs56376587	A	C	A	C	-0.02835	-0.004091712	0.5817	0.5774	0.4935	0.0059733	0.002564	2.19E-28	0.000202	122.256
rs56790613	A	G	A	G	0.0377	0.012956508	0.9673	0.9823	0.5624	0.02237739	0.006576	1.01E-08	5.50E-05	32.86693
rs569550	T	G	T	G	0.03941	0.005419381	0.6382	0.473	0.3594	0.0059099	0.002628	8.92E-51	0.000372	224.8859
rs57420239	C	T	C	T	-0.03706	-0.029267794	0.9678	0.0174	0.1946	0.02256576	0.006577	1.80E-08	5.31E-05	31.75083
rs57484993	A	G	A	G	0.03418	0.007730853	0.9219	0.8369	0.3332	0.00798642	0.004607	1.22E-13	9.12E-05	55.04371
rs576943137	C	T	C	T	-0.02548	-0.008884475	0.8922	0.091	0.3864	0.01025921	0.004521	1.79E-08	5.26E-05	31.76361
rs578097	A	G	A	G	-0.02281	-0.000192198	0.652	0.6533	0.9752	0.00619992	0.002544	3.25E-19	0.000133	80.39265
rs57850577	A	G	A	G	-0.05408	0.004363584	0.9691	0.9804	0.8373	0.02128578	0.006479	7.43E-17	0.000117	69.67186
rs57944734	C	T	C	T	-0.04826	0.003782579	0.9619	0.0237	0.8456	0.01939784	0.006162	5.02E-15	0.000102	61.33823
rs58320941	G	T	G	T	-0.04411	-0.040580063	0.9695	0.0165	0.07969	0.02316214	0.006735	5.96E-11	7.18E-05	42.89423
rs59482369	T	C	T	C	-0.04242	0.001393134	0.959	0.9748	0.9409	0.01882614	0.006235	1.05E-11	7.75E-05	46.288
rs60085321	C	G	C	G	-0.05059	0.000374811	0.9688	0.9795	0.9855	0.02082285	0.006686	4.02E-14	9.48E-05	57.25278
rs6013199	G	A	G	A	-0.07916	0.045778432	0.9722	0.0057	0.2427	0.03919386	0.01317	1.89E-09	0.000205	36.12768
rs6031431	A	G	A	G	0.01961	0.00091443	0.5497	0.6481	0.882	0.00617858	0.002464	1.81E-15	0.000105	63.33938
rs60772526	C	T	C	T	0.0605	0.004382181	0.127	0.8425	0.5883	0.00810015	0.003746	1.27E-58	0.000432	260.8406
rs6108787	T	G	T	G	0.01367	-0.004130362	0.5405	0.6781	0.5132	0.00631554	0.002403	1.31E-08	5.36E-05	32.36156
rs61169316	G	A	G	A	0.01814	-0.001176103	0.7215	0.3271	0.8514	0.00628932	0.002753	4.56E-11	7.19E-05	43.41723
rs61793790	G	C	G	C	-0.03709	0.00030985	0.9597	0.9697	0.9853	0.0172139	0.006649	2.49E-08	5.21E-05	31.11724
rs61897793	G	A	G	A	-0.02178	-0.023203649	0.8642	0.0951	0.02107	0.01005793	0.003653	2.56E-09	5.95E-05	35.5481
rs62193645	C	T	C	T	0.03441	0.013608097	0.8796	0.1052	0.1571	0.00961703	0.003785	1.04E-19	0.000137	82.649
rs62330329	T	C	T	C	0.02703	0.005094582	0.2697	0.2845	0.4358	0.0065399	0.004425	1.04E-09	6.18E-05	37.31346
rs62374068	A	G	A	G	0.05943	0.014627481	0.7821	0.8145	0.05404	0.0075908	0.002918	3.67E-92	0.000687	414.802
rs62435145	G	T	G	T	-0.0256	-0.007328798	0.434	0.5277	0.2151	0.00591032	0.0027	2.68E-21	0.000149	89.89849
rs66482211	A	G	A	G	-0.04204	0.004050352	0.9505	0.9665	0.8048	0.01639819	0.005675	1.34E-13	9.09E-05	54.87742
rs66561220	T	C	T	C	-0.02949	-0.004036533	0.7728	0.8002	0.5843	0.0073794	0.003016	1.49E-22	0.000158	95.60638
rs6697367	C	T	C	T	-0.01868	-0.005398879	0.6768	0.275	0.4141	0.00660817	0.002575	4.19E-13	8.72E-05	52.62587
rs6703881	T	C	T	C	-0.02296	0.010203513	0.1968	0.1287	0.2469	0.00881132	0.00312	1.91E-13	8.97E-05	54.1545
rs6716091	C	T	C	T	0.02352	0.002171433	0.7446	0.1772	0.779	0.00772752	0.002796	4.19E-17	0.000117	70.76203
rs6755070	G	T	G	T	0.01832	-0.011195689	0.6877	0.2682	0.09275	0.00666014	0.002757	3.10E-11	7.31E-05	44.15475
rs6794202	C	T	C	T	0.02384	0.011872197	0.7896	0.2503	0.08141	0.00681136	0.003025	3.39E-15	0.000103	62.11003
rs6816915	G	A	G	A	-0.01441	-0.009715948	0.4714	0.5957	0.106	0.00601234	0.002594	2.83E-08	5.11E-05	30.85944
rs6826742	G	T	G	T	-0.01667	0.001216797	0.4695	0.4785	0.8367	0.00590678	0.00243	7.13E-12	7.79E-05	47.06073
rs6877631	T	C	T	C	-0.0154	-0.002933895	0.6149	0.6872	0.6451	0.0063642	0.002594	2.99E-09	5.84E-05	35.24532
rs690054	C	T	C	T	-0.03594	-0.008661066	0.5832	0.3648	0.1576	0.00612956	0.002501	8.68E-47	0.000342	206.5041
rs6934923	G	T	T	C	-0.05549	-0.176600658	0.9748	0.9995	0.1809	0.13198853	0.007262	2.25E-14	9.77E-05	58.38711
rs6934923	G	T	T	C	-0.05549	-0.029940567	0.9748	0.9823	0.1809	0.02237711	0.007262	2.25E-14	9.77E-05	58.38711
rs6941546	G	A	G	A	-0.02568	0.007817309	0.9129	0.0654	0.5127	0.01193482	0.004349	3.63E-09	5.77E-05	34.86673
rs6961048	C	G	C	G	0.04642	0.010133264	0.8895	0.8657	0.2414	0.00865351	0.003761	5.76E-35	0.000252	152.3364
rs697976	T	C	T	C	0.01364	0.002118752	0.4779	0.5065	0.7199	0.00590181	0.0024	1.34E-08	5.35E-05	32.30028
rs7038668	T	C	T	C	-0.0279	-0.007076875	0.5014	0.5095	0.2305	0.00590231	0.002425	1.38E-30	0.000219	132.3686
rs71537958	G	C	G	C	-0.01936	-0.002076575	0.7389	0.7893	0.7742	0.00723545	0.002891	2.22E-11	7.43E-05	44.84505
rs716877	C	G	C	G	0.03596	-0.013608162	0.5162	0.5658	0.02223	0.00595283	0.002627	1.35E-42	0.00031	187.3783
rs7187932	G	A	G	A	-0.0223	-0.003652244	0.7746	0.1766	0.6369	0.0077378	0.002875	9.15E-15	9.96E-05	60.16363
rs7218857	T	C	T	C	-0.02113	-0.003481466	0.5618	0.4182	0.5609	0.0059819	0.002471	1.30E-17	0.000121	73.12292
rs7222451	G	A	G	A	-0.01388	-0.002713962	0.4975	0.5453	0.6471	0.00592568	0.002471	1.99E-08	5.23E-05	31.55248
rs72969822	C	G	C	G	-0.0249	0.009464108	0.8821	0.8822	0.301	0.00915291	0.003796	5.55E-11	7.13E-05	43.02752
rs73776711	G	A	G	A	-0.0479	0.001363984	0.9631	0.0228	0.9453	0.01976788	0.006173	8.96E-15	9.97E-05	60.21137
rs7416991	T	G	A	G	-0.01795	0.01578662	0.6404	0.398	0.008824	0.00602773	0.002504	7.97E-13	8.51E-05	51.38783
rs7416991	T	C	T	C	0.01931	0.01578662	0.4025	0.398	0.008824	0.00602773	0.002593	1.01E-13	9.18E-05	55.4574
rs7416991	T	G	A	G	-0.01795	0.015977807	0.6404	0.6269	0.008824	0.00610073	0.002504	7.97E-13	8.51E-05	51.38783
rs7416991	T	C	T	C	0.01931	0.015977807	0.4025	0.6269	0.008824	0.00610073	0.002593	1.01E-13	9.18E-05	55.4574
rs74496669	G	T	G	T	0.03972	0.00979783	0.9698	0.0165	0.6724	0.02316272	0.00716	2.96E-08	5.15E-05	30.7746
rs74518739	C	T	C	T	-0.04059	-0.041512215	0.9527	0.0248	0.02867	0.01897268	0.005761	1.91E-12	8.31E-05	49.64121
rs75248620	G	A	G	A	0.03605	-0.021459589	0.9368	0.049	0.1163	0.01366853	0.00518	3.55E-12	8.02E-05	48.43408
rs75411357	A	T	A	T	0.03822	0.016119943	0.9656	0.9812	0.458	0.02172499	0.006723	1.35E-08	5.41E-05	32.31879
rs75741381	C	G	C	G	-0.01862	-0.011348833	0.7999	0.7858	0.1146	0.00719191	0.003124	2.60E-09	5.88E-05	35.52526
rs76075621	T	C	T	C	0.03689	0.012229176	0.9658	0.9816	0.5776	0.02195543	0.006443	1.05E-08	5.49E-05	32.78242
rs761356	T	C	T	C	0.02838	0.004570599	0.6163	0.6517	0.4603	0.00619322	0.002531	3.76E-29	0.000208	125.7305
rs76200870	G	A	G	A	-0.05573	-0.001357516	0.9737	0.0179	0.9516	0.02225435	0.007021	2.17E-15	0.000105	63.00574
rs76666870	T	C	T	C	0.03689	0.012229176	0.9658	0.9816	0.5776	0.02195543	0.006443	1.05E-08	5.49E-05	32.78242
rs76821958	A	G	A	G	-0.04048	0.033765676	0.9734	0.985	0.1642	0.02427439	0.006957	6.08E-09	5.61E-05	33.85611
rs76987554	C	T	C	T	-0.07778	-0.005559519	0.982	0.0121	0.8367	0.02698796	0.009027	7.31E-18	0.000124	74.24189
rs77081469	C	A	C	A	0.06558	-0.014291251	0.9709	0.0038	0.7657	0.04795722	0.01191	3.76E-08	0.000172	30.31931
rs7726795	T	C	T	C	0.02854	-0.001903382	0.6314	0.722	0.7725	0.0065861	0.002657	7.04E-27	0.000191	115.3785
rs77290217	T	C	T	C	-0.04754	0.008040506	0.9649	0.9774	0.6855	0.0198531	0.006553	4.18E-13	8.81E-05	52.63056
rs7749375	G	A	G	A	-0.05485	0.003926499	0.975	0.0174	0.8619	0.02256609	0.007327	7.38E-14	9.38E-05	56.04036
rs7766997	A	G	A	G	-0.05467	0.0016877	0.9749	0.9825	0.9404	0.02250267	0.007245	4.68E-14	9.53E-05	56.94051
rs7767054	G	A	G	A	-0.05415	0.002945664	0.9743	0.0178	0.8949	0.02231564	0.00719	5.22E-14	9.49E-05	56.72038
rs77747253	G	T	G	T	-0.1012	-0.048784975	0.9865	0.0019	0.4713	0.06775691	0.0181	2.29E-08	0.000178	31.26107
rs780101	C	T	C	T	0.02158	-0.007532008	0.5339	0.5116	0.2018	0.00590283	0.002504	7.30E-18	0.000123	74.27356
rs78132593	C	A	C	A	0.01948	0.018737826	0.8381	0.1218	0.03783	0.00902158	0.003462	1.87E-08	5.24E-05	31.66094
rs7836572	T	C	T	C	0.02144	0.000823854	0.4105	0.4244	0.8901	0.00596996	0.002459	2.91E-18	0.000126	76.02081
rs78716205	G	A	G	A	0.0679	-0.000597599	0.9669	0.0181	0.9788	0.02213331	0.006869	5.19E-23	0.000162	97.71304
rs79030490	C	A	C	A	-0.07534	-0.007040602	0.9817	0.0123	0.7922	0.02677035	0.008966	4.59E-17	0.000118	70.60798
rs79285851	C	T	C	T	-0.1018	-0.048852731	0.9864	0.0019	0.471	0.06775691	0.01811	1.93E-08	0.00018	31.59793
rs79392591	C	G	C	G	-0.0245	0.007512679	0.8683	0.8818	0.411	0.00913951	0.003564	6.53E-12	7.83E-05	47.25598
rs79557018	C	T	C	T	-0.05487	0.00126015	0.9749	0.0175	0.9552	0.02250267	0.007246	3.81E-14	9.60E-05	57.34206
rs79590186	C	T	C	T	-0.07237	-0.008552516	0.976	0.0148	0.7262	0.02443576	0.007995	1.49E-19	0.000137	81.93703
rs79889868	G	C	G	C	-0.05507	-0.003560251	0.9696	0.9807	0.8684	0.0214473	0.00659	6.80E-17	0.000117	69.83278
rs80176668	A	G	A	G	-0.07368	-0.007356037	0.9774	0.9856	0.7666	0.0247678	0.007748	2.05E-21	0.000151	90.43156
rs80289662	C	G	C	G	-0.05427	0.004375248	0.9694	0.9807	0.8382	0.02144729	0.006589	1.86E-16	0.000114	67.83919
rs80328223	C	T	C	T	-0.07624	-0.010470431	0.9784	0.0136	0.6809	0.0254755	0.008251	2.62E-20	0.000143	85.3793

rs8077544	G	A	G	A	0.01629	-0.008716688	0.3428	0.7089	0.1795	0.0064953	0.00285	1.13E-08	5.41E-05	32.67025
rs8125560	A	C	A	C	-0.01906	0.012550158	0.825	0.8421	0.1208	0.00809166	0.003137	1.28E-09	6.11E-05	36.91618
rs820429	G	T	G	T	-0.04348	-0.006761097	0.6755	0.3407	0.2776	0.00622569	0.002787	7.92E-55	0.000403	243.3913
rs880315	T	C	T	C	0.04161	0.006340428	0.6739	0.5453	0.2845	0.00592563	0.002572	7.72E-59	0.000433	261.73
rs923000	A	C	A	C	0.02374	-0.002753487	0.637	0.7087	0.6714	0.00649407	0.002662	4.99E-19	0.000132	79.53263
rs9321413	C	G	C	G	0.03641	0.01372261	0.9653	0.9813	0.5285	0.02178192	0.006408	1.36E-08	5.40E-05	32.28467
rs9321414	A	C	A	C	0.03723	0.023702079	0.9642	0.9794	0.254	0.02077308	0.006659	2.30E-08	5.23E-05	31.25849
rs9483608	A	G	A	G	0.03662	0.009678541	0.9657	0.9815	0.6587	0.02189715	0.006344	7.99E-09	5.58E-05	33.32041
rs9483610	G	A	G	A	0.03663	0.009634747	0.9657	0.0185	0.66	0.02189715	0.00635	8.18E-09	5.57E-05	33.27564
rs9483614	C	T	C	T	0.03847	0.014227107	0.9663	0.0184	0.5169	0.02195541	0.006761	1.30E-08	5.42E-05	32.37595
rs9493708	C	T	C	T	0.03629	0.006316774	0.9655	0.0187	0.7721	0.02178198	0.006343	1.09E-08	5.48E-05	32.7329
rs9493713	C	A	C	A	0.03717	0.009770177	0.9659	0.0184	0.6562	0.02195545	0.006432	7.72E-09	5.59E-05	33.39589
rs9493714	C	T	C	T	0.03664	0.008601824	0.9646	0.019	0.6909	0.02161262	0.006371	9.05E-09	5.54E-05	33.07469
rs9493720	T	C	T	C	0.03665	0.011312761	0.9654	0.9814	0.6048	0.02183931	0.006401	1.06E-08	5.49E-05	32.78327
rs9493722	T	C	T	C	0.03711	0.009770177	0.9659	0.9816	0.6561	0.02195545	0.006433	8.22E-09	5.57E-05	33.27782
rs9493723	T	C	T	C	0.03665	0.011312761	0.9654	0.9814	0.6048	0.02183931	0.006401	1.06E-08	5.49E-05	32.78327
rs9493727	A	G	A	G	0.03721	0.023604518	0.9641	0.9793	0.2549	0.0207239	0.006656	2.32E-08	5.23E-05	31.25307
rs9493728	A	G	A	G	0.03714	0.023791029	0.9642	0.9793	0.2511	0.02072389	0.006656	2.48E-08	5.21E-05	31.1356
rs9493729	G	T	G	T	0.03978	0.009682017	0.9699	0.0165	0.6758	0.02316272	0.007166	2.92E-08	5.16E-05	30.81598
rs9603367	C	T	C	T	0.05097	-0.006341769	0.609	0.3292	0.3125	0.00627898	0.002692	7.22E-80	0.000593	358.4917
rs963837	T	C	T	C	0.0215	-0.002342073	0.6211	0.5857	0.6956	0.00598996	0.002687	1.29E-15	0.000106	64.02382
rs9819344	C	T	C	T	0.03573	0.012743539	0.9518	0.0259	0.4926	0.01857659	0.005812	8.11E-10	6.33E-05	37.7933
rs9821489	G	A	G	A	0.04282	-0.000849807	0.8886	0.0797	0.9378	0.01089496	0.004507	2.20E-21	0.000149	90.26476
rs9822474	A	G	A	G	-0.01603	-0.000718751	0.5519	0.676	0.909	0.00630483	0.00239	2.06E-11	7.45E-05	44.98536
rs9844949	A	G	A	G	0.01972	-0.010131317	0.152	0.1118	0.2792	0.00936351	0.003549	2.83E-08	5.11E-05	30.87463
rs9918487	G	A	G	A	-0.05331	0.003856935	0.9685	0.02	0.8551	0.02107615	0.006354	5.13E-17	0.000118	70.39194
rs9928653	T	C	T	C	-0.01848	0.009276833	0.7631	0.7783	0.1914	0.00710324	0.002844	8.46E-11	6.99E-05	42.22258

Iron

SNP	effect_allel	other_allel	effect_allel	other_allel	beta.exposure	beta.outcome	eaf.exposure	eaf.outcome	pval.outcome	se.outcome	se.exposure	pval.exposure	R2	Fstat
rs10831924	T	C	T	C	0.06204	-0.027316233	0.9131	0.9519	0.04755	0.01378911	0.007982	8.06E-15	0.000474	60.41157
rs12328766	A	G	A	G	0.03348	-7.52E-05	0.6129	0.6678	0.9907	0.00626464	0.004341	1.29E-14	0.000466	59.48279
rs13007705	C	T	C	T	-0.02771	-0.002479085	0.544	0.464	0.6755	0.00591667	0.004194	4.05E-11	0.000342	43.65321
rs13081052	T	C	T	C	0.03643	-0.008462795	0.248	0.2718	0.202	0.00663228	0.005395	1.51E-11	0.000358	45.59691
rs140393761	A	G	A	G	-0.123	-0.018353003	0.9874	0.9897	0.53	0.02922453	0.0201	9.56E-10	0.000296	37.44709
rs150375987	C	G	C	G	-0.1303	-0.011119083	0.9792	0.9695	0.5173	0.01715908	0.01552	4.96E-17	0.000557	70.48641
rs1800562	G	A	G	A	-0.3257	-0.023434891	0.9509	0.025	0.2151	0.01889911	0.01006	1.05E-229	0.008227	1048.189
rs190399027	G	A	G	A	-0.1314	0.016957783	0.9894	0.0117	0.5365	0.02743978	0.02111	4.98E-10	0.000307	38.74487
rs1958078	A	C	A	C	-0.03145	-0.009487352	0.2447	0.1848	0.2122	0.00760204	0.005338	3.94E-09	0.000272	34.71236
rs218248	G	A	G	A	-0.03636	0.003609618	0.8382	0.2277	0.6078	0.00703629	0.00575	2.64E-10	0.000314	39.98638
rs2294915	C	T	C	T	-0.03195	-0.016613149	0.7616	0.4126	0.005568	0.0059932	0.005059	2.78E-10	0.000313	39.88525
rs28929474	C	T	C	T	-0.1763	0.055903344	0.9866	0.0059	0.1468	0.03852746	0.0188	7.11E-21	0.000695	87.9405
rs555273273	C	T	C	T	0.1873	0.023266516	0.984	0.0034	0.6464	0.05068958	0.03115	1.88E-09	0.000855	36.15426
rs5995385	T	C	T	C	0.02734	0.005633734	0.5347	0.6083	0.3513	0.00604478	0.004294	1.98E-10	0.000318	40.53899
rs72839066	A	G	A	G	-0.1163	-0.005844048	0.9694	0.9683	0.7289	0.01684164	0.01327	1.99E-18	0.000607	76.80997
rs7385804	C	A	C	A	-0.05785	-0.008436748	0.357	0.6912	0.1866	0.00638664	0.004417	3.75E-39	0.001344	171.5347
rs7775698	C	T	C	T	-0.0471	0.001281771	0.7576	0.1803	0.8674	0.00767527	0.004874	4.56E-22	0.000732	93.38363
rs806970	C	T	C	T	0.05669	0.026110262	0.945	0.0399	0.08321	0.01507521	0.009471	2.22E-09	0.000283	35.82789
rs8177252	C	A	C	A	-0.05473	0.005172467	0.6848	0.385	0.3938	0.00606385	0.00463	3.25E-32	0.001095	139.7298
rs855791	A	G	A	G	-0.1527	-0.006088357	0.3768	0.4475	0.3051	0.00593407	0.004498	2.09E-252	0.008959	1152.495
rs9402686	G	A	G	A	-0.05435	-0.003940081	0.7844	0.1969	0.5951	0.00742012	0.005267	6.25E-25	0.000834	106.4811

Zinc

SNP	effect_allel	other_allel	effect_allel	other_allel	beta.exposure	beta.outcome	eaf.exposure	eaf.outcome	pval.outcome	se.outcome	se.exposure	pval.exposure	R2	Fstat
rs10484100	G	A	G	A	-0.209	-0.000325316	0.5	0.0693	0.9776	0.01161844	0.045	3.30E-06	0.008225	21.57086
rs11763353	G	A	G	A	-0.192	-0.002109694	0.5	0.1184	0.8176	0.00913288	0.039	7.00E-07	0.009232	24.23669
rs1532423	A	G	A	G	0.178	-0.011263744	0.5	0.4166	0.05981	0.00598499	0.026	6.40E-12	0.017701	46.86982
rs2120019	C	T	C	T	-0.287	-0.005347924	0.5	0.2693	0.4213	0.00665165	0.033	1.55E-18	0.028258	75.63728
rs4333127	A	G	A	G	0.218	-0.010602652	0.5	0.1514	0.1978	0.00823187	0.047	3.00E-06	0.008204	21.51381
rs7148590	A	G	A	G	-0.14	0.00399825	0.5	0.4308	0.5023	0.00595864	0.026	1.40E-07	0.011024	28.99408

Copper

SNP	effect_allel	other_allel	effect_allel	other_allel	beta.exposure	beta.outcome	eaf.exposure	eaf.outcome	pval.outcome	se.outcome	se.exposure	pval.exposure	R2	Fstat
rs10014072	G	A	G	A	-0.164	0.00136033	0.5	0.72	0.8364	0.00657164	0.034	1.00E-06	0.008866	23.26644
rs1175550	A	G	A	G	-0.198	-0.00098769	0.5	0.8074	0.8953	0.0074825	0.032	5.00E-10	0.014506	38.28516
rs12153606	T	G	T	G	-0.159	0.004758797	0.5	0.8832	0.6045	0.00918687	0.034	2.00E-06	0.008338	21.86938
rs12582659	C	T	C	T	1.262	-0.002042277	0.5	0.0378	0.8948	0.0154718	0.27	3.00E-06	0.008329	21.84697
rs2769264	G	T	G	T	0.313	0.009992679	0.5	0.3415	0.1083	0.00622209	0.034	3.00E-20	0.031555	84.74827
rs3857536	T	C	T	C	-0.129	0.002169202	0.5	0.4537	0.7143	0.00592678	0.028	4.00E-06	0.008095	21.22577

Selenium

SNP	effect_allel	other_allel	effect_allel	other_allel	beta.exposure	beta.outcome	eaf.exposure	eaf.outcome	pval.outcome	se.outcome	se.exposure	pval.exposure	R2	Fstat
rs7700970	T	C	T	C	0.265	-0.000645025	0.5	0.7627	0.9259	0.00693575	0.035	7.17E-13	0.010362	57.32653

Carotene

SNP	effect_allel	other_allel	effect_allel	other_allel	beta.exposure	beta.outcome	eaf.exposure	eaf.outcome	pval.outcome	se.outcome	se.exposure	pval.exposure	R2	Fstat
rs10846742	A	G	A	G	-0.143552	-0.008663772	0.832123	0.289	0.1831	0.00650922	0.0208154	5.33E-12	6.85E-05	47.56078
rs9708919	T	C	T	C	0.246529	0.00150183	0.480938	0.6033	0.8037	0.00603145	0.0153225	3.03E-58	0.000373	258.867

Folate

SNP	effect_allel	other_allel	effect_allel	other_allel	beta.exposure	beta.outcome	eaf.exposure	eaf.outcome	pval.outcome	se.outcome	se.exposure	pval.exposure	R2	Fstat
rs1801133	G	A	G	A	0.096	0.000410299	0.668	0.3958	0.9456	0.0060338	0.01077	1.00E-28	0.003647	79.45314
rs652197	C	T	C	T	0.069	0.005857757	0.179	0.8463	0.474	0.00818122	0.01109	2.50E-10	0.00178	38.71106

Table S7 : Harmonization results for exposure SNPs and Verma et al. (African) T1D outcome

Outcome: Verma, A (T1D)
African American or Afro-Caribbean

Vitamin C

SNP	effect_allel	other_allel	effect_allel	other_allele.out	beta.exposure	beta.outcome	eaf.exposure	eaf.outcome	pval.outcome	se.outcome	se.exposure	pval.exposure	R2	Fstat
rs10051765	T	C	T	C	-0.039	0.004504455	0.6585	0.5089	0.2785	0.004155401	0.0066	3.64E-09	0.00067083	34.9173554
rs10136000	A	G	A	G	0.0404	-0.00558866	0.2825	0.6339	0.1949	0.004312241	0.0071	1.33E-08	0.00062207	32.3777028
rs10758628	A	C	A	C	0.0304	-0.00028959	0.4567	0.5707	0.9448	0.004196932	0.0063	1.28E-06	0.00044744	23.2844545
rs10995578	C	G	C	G	0.0296	0.003367083	0.4784	0.2178	0.5038	0.005033009	0.0062	1.91E-06	0.000438	22.792924
rs11062357	T	C	T	C	0.0305	-0.00528487	0.5118	0.9138	0.4752	0.00740178	0.0063	1.46E-06	0.00045039	23.4378937
rs11641245	C	G	C	G	0.042	0.005500983	0.848	0.9753	0.6813	0.013384386	0.0086	1.13E-06	0.00045832	23.8507301
rs11641245	C	G	C	G	0.042	0.001708386	0.848	0.5151	0.6813	0.004156657	0.0086	1.13E-06	0.00045832	23.8507301
rs1165189	A	C	A	C	-0.0378	-0.00032407	0.7469	0.6923	0.9426	0.004500963	0.0071	1.18E-07	0.00054462	28.3443761
rs117885456	A	G	A	G	0.0781	-0.04377597	0.0865	0.9866	0.01538	0.018066849	0.0116	1.70E-11	0.0008707	45.3300386
rs12610033	A	T	A	T	-0.0336	-0.00613522	0.2831	0.0681	0.4569	0.008246262	0.0073	4.86E-06	0.00040712	21.185213
rs13028225	T	C	T	C	0.1016	0.00868501	0.8569	0.9004	0.2106	0.006936909	0.0089	2.38E-30	0.0024991	130.318899
rs174547	T	C	T	C	-0.0364	0.000170123	0.6721	0.9217	0.9824	0.00773287	0.0066	3.84E-08	0.00058442	30.4168962
rs185137552	T	C	T	C	0.2368	-0.03837865	0.988	0.9995	0.6795	0.092926514	0.0392	1.58E-09	0.00073249	36.4914619
rs2366388	A	G	A	G	0.0309	0.003831483	0.4908	0.8305	0.4891	0.005536825	0.0063	8.50E-07	0.00046227	24.0566893
rs2559850	A	G	A	G	0.0583	0.000121416	0.5979	0.5617	0.9766	0.004186763	0.0064	6.30E-20	0.00159275	82.9807129
rs2941484	T	C	T	C	0.0341	0.004621723	0.4514	0.2954	0.3101	0.004553422	0.0063	6.37E-08	0.00056292	29.2973041
rs33972313	T	C	T	C	-0.3601	-0.00500161	0.0319	0.9313	0.5427	0.008212824	0.0179	4.61E-90	0.00772036	404.706501
rs339969	A	C	A	C	-0.0302	0.004413906	0.6068	0.2566	0.3532	0.004756365	0.0063	1.86E-06	0.00044157	22.9790879
rs4867910	T	C	T	C	-0.0314	0.001075778	0.657	0.35	0.8048	0.004355375	0.0068	3.45E-06	0.00040976	21.3226644
rs542903	T	C	T	C	-0.0418	0.003775775	0.1429	0.3146	0.3986	0.004473667	0.0088	2.27E-06	0.00043357	22.5625
rs56738967	C	G	C	G	0.041	0.004908857	0.321	0.2749	0.2916	0.004652945	0.0067	7.62E-10	0.0007194	37.4470929
rs6482188	A	C	A	C	-0.0347	0.003327755	0.3086	0.5824	0.4297	0.004212348	0.0068	2.83E-07	0.00050036	26.0400087
rs6693447	T	G	T	G	0.0393	-0.00197873	0.5509	0.7059	0.664	0.004559287	0.0064	6.25E-10	0.00072439	37.7072754
rs676317	T	C	T	C	-0.0366	0.007783139	0.7264	0.8048	0.1376	0.005241171	0.0074	7.33E-07	0.00047006	24.4623813
rs73035571	A	G	A	G	0.035	-0.00134967	0.7863	0.9307	0.8692	0.008179841	0.0076	4.14E-06	0.00040756	21.2084488
rs73850547	A	G	A	G	0.0333	-0.00421652	0.7381	0.8996	0.5419	0.006912324	0.0071	2.85E-06	0.00042272	21.9974211
rs7640441	A	C	A	C	0.0358	-0.00294448	0.2383	0.7952	0.5674	0.005147692	0.0074	1.19E-06	0.00044975	23.4046749
rs7740812	A	G	A	G	-0.0384	-0.00102412	0.4057	0.9273	0.8981	0.008000903	0.0064	1.88E-09	0.00069162	36
rs7740812	A	G	A	G	-0.0384	-0.00259649	0.4057	0.9894	0.8981	0.020285114	0.0064	1.88E-09	0.00069162	36
rs78575870	T	C	T	C	0.0551	0.026685692	0.077	0.9853	0.122	0.01726112	0.0117	2.46E-06	0.0004262	22.1784645
rs79234109	A	G	A	G	0.0453	-0.00506797	0.1617	0.9344	0.5461	0.008390684	0.0084	7.70E-08	0.0005588	29.0829082
rs80246741	A	G	A	G	0.1511	-0.00612628	0.9897	0.9979	0.8929	0.045379862	0.0319	2.21E-06	0.00043114	22.4361101
rs868822	T	G	T	G	-0.0329	-0.01370745	0.2648	0.7227	0.003136	0.0046403	0.0071	3.30E-06	0.00041263	21.4721285
rs9895661	T	C	T	C	0.0625	0.006241386	0.817	0.4797	0.1334	0.004158152	0.0081	1.05E-14	0.00114329	59.5374181
rs9915323	A	T	A	T	0.0321	-0.00573827	0.2967	0.3158	0.1993	0.004469055	0.0068	2.60E-06	0.00042822	22.2839533

Vitamin D

rs10741657	A	G	A	G	0.0308	-0.00722005	0.5	0.2656	0.1248	0.004703613	0.0022	2.05E-46	0.00285448	196
rs10745742	T	C	T	C	0.0165	-0.00116546	0.5	0.3644	0.7871	0.004316533	0.0022	1.88E-14	0.00081262	56.25
rs10888491	A	G	A	G	0.012	-0.0007064	0.5	0.7794	0.8875	0.00500995	0.0022	8.62E-08	0.00042921	29.7520661
rs11195965	A	T	A	T	-0.0157	0.002129513	0.5	0.6972	0.6373	0.004521258	0.0032	6.31E-07	0.00033962	24.0712891
rs11203339	T	C	T	C	-0.0104	-9.14E-05	0.5	0.491	0.9824	0.004155437	0.0021	8.56E-07	0.00031432	24.5260771
rs12507653	A	T	A	T	0.0118	0.007133516	0.5	0.7199	0.1231	0.004626145	0.0025	2.21E-06	0.0003538	22.2784
rs12785878	T	G	T	G	0.0363	0.006490616	0.5	0.7538	0.1782	0.004822152	0.0022	3.81E-62	0.00346382	272.25
rs17082722	T	C	T	C	-0.0703	0.016254749	0.5	0.9649	0.15	0.01128802	0.0149	2.22E-06	0.00063976	22.2606639
rs17216707	T	C	T	C	0.0263	-0.00165886	0.5	0.9349	0.8435	0.008420604	0.0027	8.14E-23	0.0013256	94.8820302
rs1809851	A	T	A	T	0.0104	0.000742639	0.5	0.6018	0.861	0.00424365	0.0023	4.86E-06	0.00029959	20.4461248
rs204286	A	T	A	T	0.01	0.001189926	0.5	0.4354	0.7767	0.00418988	0.0022	4.69E-06	0.00029808	20.661157
rs2597193	A	G	A	G	0.0187	0.001693047	0.5	0.5428	0.685	0.004170067	0.0022	6.26E-17	0.00097292	72.25
rs3755967	T	C	T	C	-0.0892	0.000751557	0.5	0.9091	0.9174	0.007226509	0.0023	0	0.01886388	1504.09074
rs4821976	A	G	A	G	0.01	-1.28E-05	0.5	0.3798	0.9976	0.004280288	0.0021	2.74E-06	0.00033961	22.675737
rs6780224	T	C	T	C	-0.0105	0.005179797	0.5	0.3862	0.2246	0.004266719	0.0023	4.46E-06	0.00030398	20.8412098
rs6982502	T	C	T	C	-0.0094	0.011571223	0.5	0.1596	0.04131	0.005672168	0.002	4.04E-06	0.00030187	22.09
rs7011866	T	G	T	C	-0.0404	0.014869766	0.5	0.991	0.4988	0.021996695	0.0084	1.35E-06	0.00039756	23.1315193
rs7011866	T	G	T	C	-0.0404	0.034088446	0.5	0.9983	0.4988	0.050426696	0.0084	1.35E-06	0.00039756	23.1315193
rs7675387	T	G	T	G	0.0181	0.012447079	0.5	0.8363	0.0266	0.00561438	0.0023	4.18E-15	0.0009903	61.9300567
rs7781168	A	G	A	G	0.0096	0.004289812	0.5	0.5157	0.302	0.004156795	0.002	2.95E-06	0.00030148	23.04
rs793000	A	G	A	G	0.0099	0.003639463	0.5	0.4764	0.3814	0.004159386	0.0022	4.90E-06	0.00029524	20.25
rs8018720	C	G	C	G	-0.0168	-0.00158942	0.5	0.1432	0.7889	0.005930687	0.0029	4.72E-09	0.00049233	33.5600476
rs904856	A	G	A	G	0.0228	0.011351244	0.5	0.907	0.1126	0.007152643	0.0045	4.12E-07	0.00033898	25.6711111
rs914787	T	C	T	C	0.0105	0.006572856	0.5	0.8776	0.2998	0.006338337	0.0022	1.25E-06	0.00034565	22.7789256

Retinol (vitamin a)

rs1667226	T	A	T	A	0.101271	0.001728041	0.520114	0.5971	0.6832	0.004235395	0.0154605	5.74E-11	6.18E-05	42.9065037
rs1883711	C	G	C	G	-0.27778	-0.03846552	0.029437	0.0054	0.1747	0.028346002	0.0454347	9.73E-10	5.38E-05	37.3789087

Vitamin K (1st model)

SNP	effect_allel	other_allel	effect_allel	other_allele.out	beta.exposure	beta.outcome	eaf.exposure	eaf.outcome	pval.outcome	se.outcome	se.exposure	pval.exposure	R2	Fstat
rs2108622	T	C	T	C	0.16	0.000421539	0.3	0.9032	0.9525	0.007025653	0.032	8.78E-07	0.01156872	25
rs2192574	C	T	C	T	0.28	0.001535037	0.11	0.90565	0.8286	0.007106653	0.058	1.82E-06	0.01079309	23.3055886
rs4645543	T	C	T	C	-0.42	0.000208034	0.04	0.9539	0.9834	0.009906368	0.079	2.00E-07	0.01305972	28.2647012
rs4852146	C	T	C	T	0.18	-0.00684726	0.33	0.7024	0.1318	0.004543635	0.037	2.08E-06	0.01095859	23.6669102
rs964184	G	C	G	C	0.23	0.001068789	0.15	0.2058	0.8355	0.005138409	0.042	5.91E-08	0.01384525	29.9886621
**(2nd model)**														
SNP	effect_allel	other_allel	effect_allel	other_allele.out	beta.exposure	beta.outcome	eaf.exposure	eaf.outcome	pval.outcome	se.outcome	se.exposure	pval.exposure	R2	Fstat
rs12609820	C	T	C	T	0.16	0.00380816	0.31	0.1715	0.4898	0.005511086	0.03	3.06E-07	0.01314168	28.4444444
rs2108622	T	C	T	C	0.16	0.000421539	0.3	0.9032	0.9525	0.007025653	0.031	2.90E-07	0.01231778	26.6389178
rs2192574	C	T	C	T	0.28	0.001535037	0.11	0.90565	0.8286	0.007106653	0.057	1.49E-06	0.01117085	24.1305017
rs4122275	A	G	A	G	-0.81	0.002363199	0.02	0.6597	0.5897	0.004384414	0.16	4.31E-07	0.01185629	25.6289063
rs4852146	C	T	C	T	0.19	-0.00684726	0.33	0.7024	0.1318	0.004543635	0.035	1.42E-07	0.01360878	29.4693878
rs964184	G	C	G	C	0.14	0.001068789	0.15	0.2058	0.8355	0.005138409	0.042	5.91E-08	0.00517491	11.1111111
**(3rd model)**														
SNP	effect_allel	other_allel	effect_allel	other_allele.out	beta.exposure	beta.outcome	eaf.exposure	eaf.outcome	pval.outcome	se.outcome	se.exposure	pval.exposure	R2	Fstat
rs2108622	T	C	T	C	0.16	0.000421539	0.3	0.9032	0.9525	0.007025653	0.032	9.68E-07	0.01156872	25
rs2192574	C	T	C	T	0.29	0.001535037	0.11	0.90565	0.8286	0.007106653	0.058	8.23E-07	0.01156872	25
rs4122275	A	G	A	G	-0.78	0.002363199	0.02	0.6597	0.5897	0.004384414	0.17	3.90E-06	0.00975957	21.0519031
rs4852146	C	T	C	T	0.18	-0.00684726	0.33	0.7024	0.1318	0.004543635	0.038	3.23E-06	0.01039533	22.4376731

Vitamin B12

rs1131603	C	T	C	T	0.22	-0.01886601	0.06	0.0082	0.4129	0.02303542	0.015	4.90E-49	0.00469797	215.111111
rs1141321	C	T	C	T	0.07	0.00665228	0.63	0.2034	0.1975	0.005160807	0.007	3.60E-26	0.00218953	100
rs1801222	G	A	G	A	0.12	-0.00766565	0.59	0.8043	0.1431	0.0052361	0.007	3.30E-75	0.00640704	293.877551
rs2270655	G	C	G	C	0.1	-0.00528705	0.94	0.9567	0.6046	0.010206669	0.015	2.20E-13	0.00097426	44.4444444
rs2336573	T	C	T	C	0.31	0.011373937	0.03	0.6958	0.01176	0.004515259	0.019	8.40E-59	0.00580736	266.204986
rs34324219	C	A	C	A	0.23	0.007335461	0.88	0.0654	0.3825	0.00840259	0.011	1.10E-111	0.00950182	437.190083
rs3742801	T	C	T	C	0.05	0.003493322	0.29	0.8135	0.5122	0.005333317	0.007	1.70E-13	0.00111838	51.0204082
rs41281112	C	T	C	T	0.18	0.001115827	0.95	0.0053	0.9687	0.028610956	0.015	8.90E-35	0.00314974	144
rs602662	A	G	A	G	0.17	0.005285009	0.6	0.4958	0.2033	0.004154882	0.007	2.40E-139	0.01277837	589.795918

Vitamin B6

rs4654748	C	T	C	T	1.45	-0.01164744	0.5	0.1237	0.06482	0.006309555	0.281	8.00E-18	0.00899981	26.6270691

Alpha-tocopherol (vitamin E)

SNP	effect_allel	other_allel	effect_allel	other_allele.out	beta.exposure	beta.outcome	eaf.exposure	eaf.outcome	pval.outcome	se.outcome	se.exposure	pval.exposure	R2	Fstat
rs964184	G	C	G	C	0.04	0.001068789	0.15	0.2058	0.8355	0.005138409	0.01	8.00E-12	0.00423168	16

Gamma and beta-tocopherol

SNP	effect_allel	other_allel	effect_allel	other_allele.out	beta.exposure	beta.outcome	eaf.exposure	eaf.outcome	pval.outcome	se.outcome	se.exposure	pval.exposure	R2	Fstat
rs11705639	A	C	T	C	0.12578066	-0.00521573	0.1685	0.9107	0.4742	0.007284538	0.0138	5.00E-20	0.01437511	83.07485
rs62508088	T	C	T	C	0.16487662	0.015711034	0.0992	0.9718	0.2106	0.012548749	0.0179	1.00E-21	0.01467645	84.8422328

Magnesium

SNP	effect_allel	other_allel	effect_allel	other_allele.out	beta.exposure	beta.outcome	eaf.exposure	eaf.outcome	pval.outcome	se.outcome	se.exposure	pval.exposure	R2	Fstat
rs10043693	A	G	A	G	0.03589	0.000550303	0.6921	0.8597	0.927	0.005981554	0.004433	5.63E-16	0.00045008	65.5467939
rs1035283	G	A	G	A	-0.06605	-0.0106213	0.9368	0.1631	0.05883	0.005622713	0.00826	1.27E-15	0.00043906	63.9419018
rs10747045	T	G	T	G	-0.02741	0.007513795	0.6696	0.8829	0.2447	0.006460701	0.004336	2.57E-10	0.00027444	39.9612923
rs10952168	G	A	G	A	0.02429	0.000958297	0.4401	0.6192	0.8231	0.004278114	0.004443	4.57E-08	0.00020528	29.8883819
rs10974444	G	C	G	C	0.02687	0.006189265	0.6607	0.8789	0.331	0.006367556	0.004233	2.19E-10	0.00027673	40.2938528
rs11234579	C	T	C	T	-0.02714	0.007500528	0.6373	0.0867	0.3095	0.00738241	0.004453	1.10E-09	0.00025511	37.1461918
rs112510641	G	A	G	A	0.03035	0.0004409	0.6104	0.1371	0.9415	0.006039728	0.00418	3.86E-13	0.00036203	52.7187164
rs113174770	T	G	T	G	0.04893	-0.01827494	0.9334	0.9728	0.1524	0.012770747	0.008128	1.74E-09	0.00024889	36.2395705
rs115478735	A	T	A	T	-0.04194	-0.00568827	0.8064	0.9582	0.5836	0.010380053	0.00508	1.52E-16	0.00046801	68.1599758
rs11614506	T	C	T	C	0.03278	0.014747503	0.7819	0.956	0.1453	0.010128779	0.004892	2.09E-11	0.00030835	44.8998615
rs11694498	T	A	T	A	-0.04061	0.005151044	0.6086	0.6389	0.2336	0.004324974	0.004166	1.88E-22	0.00065234	95.0227178
rs117672478	C	A	C	A	-0.08527	-0.04073472	0.9763	0.0041	0.2103	0.03250975	0.01506	1.51E-08	0.00022018	32.0584545
rs12203597	G	A	G	A	-0.03104	-0.00201936	0.3435	0.2044	0.6954	0.005151435	0.004361	1.10E-12	0.0003479	50.6607076
rs12230212	T	A	T	A	0.1637	0.056259281	0.989	0.9964	0.1049	0.03468513	0.02743	2.39E-09	0.00024461	35.6160493
rs1229984	T	C	T	C	0.07801	-0.02316508	0.03517	0.01096	0.2456	0.019952698	0.01116	2.79E-12	0.00033555	48.8621043
rs12464156	T	C	T	C	-0.02627	-0.00131086	0.5962	0.7145	0.7754	0.004599515	0.004107	1.58E-10	0.00028098	40.9138869
rs1273884	G	A	G	A	0.04993	0.001853046	0.5595	0.4975	0.6558	0.004154812	0.004091	2.87E-34	0.00102224	148.958117
rs12743084	C	G	C	G	-0.08845	0.00551935	0.4615	0.3521	0.2045	0.00434937	0.004065	5.97E-105	0.00324187	473.450495
rs12918968	A	C	A	C	-0.03944	0.003808043	0.5741	0.2879	0.4063	0.004588004	0.004172	3.25E-21	0.00061355	89.3686532
rs13143189	G	A	G	A	0.02938	0.003453216	0.5727	0.2177	0.4926	0.005033842	0.004194	2.47E-12	0.000337	49.0734658
rs13146355	G	A	G	A	-0.06525	0.003425535	0.5558	0.1399	0.5674	0.005988698	0.004024	3.91E-59	0.00180299	262.932995
rs13170671	C	T	C	T	0.02378	-0.00477185	0.5502	0.2066	0.3522	0.005131017	0.004199	1.48E-08	0.00022028	32.0724363
rs13193692	G	T	G	T	-0.03149	0.002181675	0.7824	0.0689	0.7902	0.008201786	0.005055	4.69E-10	0.00026651	38.8063683
rs142601087	T	C	T	C	0.02922	-0.0039561	0.806	0.6522	0.3645	0.004361739	0.005168	1.57E-08	0.00021956	31.9679961
rs143135527	C	T	C	T	0.08315	0.005503724	0.9719	0.0049	0.853	0.029749858	0.01462	1.28E-08	0.00022216	32.3466837
rs17794420	G	A	G	A	0.02859	-0.00470385	0.5174	0.1609	0.4053	0.005653666	0.004373	6.19E-11	0.00029354	42.7434287
rs17832417	A	T	A	T	0.03899	-0.00160136	0.6265	0.7203	0.7292	0.004628212	0.004404	8.47E-19	0.00053816	78.3811895
rs1890185	A	G	A	G	-0.02732	-0.00440163	0.5875	0.6246	0.3049	0.004290089	0.004063	1.75E-11	0.0003105	45.2134602
rs219782	A	G	A	G	-0.06371	-0.00649813	0.7483	0.6372	0.1325	0.004320566	0.004632	4.75E-43	0.00129791	189.181258
rs2439722	T	C	T	C	-0.02569	0.000250239	0.528	0.5436	0.9524	0.004170651	0.00424	1.36E-09	0.00025213	36.7110237
rs250383	A	T	A	T	0.05615	0.006069211	0.8149	0.5445	0.1457	0.004171279	0.005266	1.53E-26	0.00078042	113.69407
rs2510467	A	G	A	G	0.03135	0.006411539	0.4299	0.2059	0.2122	0.005137451	0.004437	1.59E-12	0.00034283	49.9224893
rs2542713	A	C	A	C	0.02645	0.003965535	0.4504	0.4845	0.34	0.004156745	0.004186	2.64E-10	0.0002742	39.9257336
rs2731238	G	A	G	A	0.05395	0.005185221	0.2993	0.4652	0.213	0.004164836	0.004601	9.56E-32	0.00094363	137.492317
rs2731238	G	T	T	C	-0.1227	-0.00518522	0.9493	0.5348	0.213	0.004164836	0.009953	6.53E-35	0.00104294	151.978137
rs2731238	G	A	G	A	0.05395	0.012556056	0.2993	0.0444	0.213	0.010085186	0.004601	9.56E-32	0.00094363	137.492317
rs2731238	G	T	T	C	-0.1227	-0.01255606	0.9493	0.9556	0.213	0.010085186	0.009953	6.53E-35	0.00104294	151.978137
rs2818759	G	T	G	T	0.03013	-0.00452699	0.344	0.864	0.455	0.006060228	0.00441	8.39E-12	0.00032056	46.6789506
rs28441180	G	A	G	A	0.02463	-0.00108594	0.491	0.5672	0.796	0.004192803	0.004398	2.15E-08	0.00021541	31.363056
rs303968	T	C	T	C	-0.03532	-0.00025352	0.6028	0.5126	0.951	0.004156084	0.004198	3.98E-17	0.00048605	70.7874913
rs34872471	T	C	T	C	0.04058	-0.04960296	0.7066	0.6609	1.17E-29	0.004385761	0.004434	5.56E-20	0.00057506	83.7592361
rs35249105	A	G	A	G	-0.04637	0.00991794	0.538	0.7494	0.03856	0.004793591	0.004121	2.25E-29	0.00086901	126.610278
rs35347302	G	A	G	A	-0.02671	-0.00373949	0.7702	0.1649	0.5042	0.00559804	0.004774	2.20E-08	0.00021499	31.3027827
rs35465213	G	A	G	A	-0.1494	0.030479502	0.9477	0.0119	0.1117	0.01915745	0.009452	2.85E-56	0.00171333	249.835282
rs35934	G	T	G	T	-0.05568	-0.00407934	0.1741	0.8542	0.4886	0.005886497	0.005631	4.71E-23	0.00067122	97.7749041
rs3732215	G	C	G	C	-0.02364	-0.00086683	0.4801	0.1575	0.8791	0.005702839	0.003994	3.26E-09	0.00024061	35.0331205
rs3783297	T	C	T	C	0.02579	-0.00031744	0.6433	0.3327	0.9427	0.004408891	0.004293	1.89E-09	0.00024786	36.0895035
rs3824347	A	G	A	G	0.05695	0.001550035	0.5864	0.7407	0.7436	0.004740168	0.004257	8.35E-41	0.00122794	178.969966
rs3848132	T	A	T	A	-0.04543	0.001976176	0.723	0.8292	0.7206	0.005520044	0.004775	1.82E-21	0.00062144	90.5187862
rs3925584	T	C	T	C	0.07105	0.006063967	0.5475	0.8787	0.3406	0.00636303	0.00402	6.73E-70	0.00214129	312.374848
rs4077450	G	T	G	T	-0.03324	-0.00030078	0.2184	0.6275	0.944	0.004296812	0.005478	1.31E-09	0.00025287	36.8195074
rs425135	A	C	A	C	-0.03465	-0.00998739	0.1466	0.3447	0.02233	0.00437085	0.005797	2.27E-09	0.00024537	35.7272737
rs4962402	T	G	T	G	-0.03358	0.003348195	0.2763	0.3155	0.4537	0.00447022	0.004637	4.43E-13	0.00036013	52.4429595
rs544934737	T	C	T	C	0.02519	0.000781435	0.5678	0.3964	0.8541	0.004246928	0.004601	4.36E-08	0.00020587	29.9744945
rs560609863	T	G	T	G	-0.05315	-0.00183254	0.8518	0.7292	0.6952	0.004674858	0.009142	6.12E-09	0.00023214	33.8005762
rs58447148	A	G	A	G	0.04638	-0.00045966	0.9139	0.8254	0.9329	0.0054722	0.007637	1.26E-09	0.0002533	36.8821226
rs58560619	T	C	T	C	0.02335	0.002756918	0.4936	0.7622	0.5722	0.004879501	0.004079	1.04E-08	0.00022506	32.7692392
rs592859	C	G	C	G	0.03162	0.005620273	0.1578	0.2004	0.2786	0.005189541	0.005773	4.31E-08	0.00020605	29.9999559
rs59359435	T	C	T	C	0.06372	0.005497342	0.9553	0.9583	0.5969	0.010391951	0.01039	8.55E-10	0.00025831	37.6114814
rs59685687	T	A	T	A	0.02874	0.000456799	0.7714	0.6749	0.9181	0.004434944	0.005196	3.16E-08	0.00021012	30.5939282
rs6003469	A	C	A	C	0.04485	0.00134493	0.6924	0.9103	0.8533	0.007269889	0.004437	5.11E-24	0.00070141	102.175327
rs606970	A	G	A	G	-0.02401	-0.00155287	0.5289	0.6076	0.7152	0.004254443	0.00432	2.74E-08	0.00021216	30.8899231
rs62136373	C	T	C	T	0.03313	0.0019982	0.8549	0.1443	0.7357	0.005911834	0.006058	4.51E-08	0.00020541	29.9077908
rs623297	C	T	C	T	0.02897	-0.00557487	0.7303	0.1095	0.4019	0.006652594	0.004862	2.56E-09	0.00024383	35.5031659
rs636264	G	A	G	A	-0.03387	0.008062812	0.2082	0.871	0.1933	0.006197396	0.005236	9.93E-11	0.00028737	41.8438
rs658903	T	A	T	A	-0.03979	-0.00282178	0.855	0.9366	0.7406	0.008525001	0.006028	4.07E-11	0.00029923	43.5713876
rs6667005	T	C	T	C	0.02947	3.84E-05	0.7844	0.3877	0.9929	0.004263697	0.004962	2.86E-09	0.00024225	35.2733536
rs6936263	G	C	G	C	-0.02746	0.000566649	0.2853	0.2693	0.9039	0.004683048	0.004564	1.78E-09	0.00024862	36.200102
rs6936263	G	C	G	C	-0.02746	-0.00058517	0.2853	0.2441	0.9039	0.004836156	0.004564	1.78E-09	0.00024862	36.200102
rs7039	C	G	C	G	0.03185	0.005276819	0.479	0.3274	0.2331	0.004426861	0.004072	5.26E-15	0.0004201	61.1791353
rs7077696	A	G	A	G	0.02798	0.007790281	0.7797	0.8087	0.1403	0.005281546	0.00501	2.34E-08	0.00021422	31.1903299
rs711819	T	C	T	C	-0.03593	0.007946794	0.2421	0.08088	0.2968	0.00761917	0.004965	4.61E-13	0.00035963	52.3691987
rs7221296	A	G	A	G	-0.04431	-0.00625698	0.8126	0.4188	0.1372	0.00421062	0.005215	1.94E-17	0.00049569	72.1929643
rs7241576	A	G	A	G	0.02973	-0.00177746	0.6835	0.8458	0.7572	0.005752282	0.004521	4.82E-11	0.00029698	43.2434967
rs72848405	T	C	T	C	0.02757	0.007036253	0.8042	0.8665	0.2492	0.006107858	0.005049	4.74E-08	0.00020479	29.8169207
rs7374260	T	C	T	C	0.02461	0.005111774	0.5278	0.458	0.2203	0.004169473	0.003992	7.04E-10	0.00026101	38.0051247
rs73949333	A	G	A	G	0.03287	0.001173305	0.776	0.4734	0.7778	0.004160655	0.005211	2.84E-10	0.00027326	39.7884721
rs7416991	T	G	A	G	-0.05192	-0.00070859	0.6967	0.4599	0.8653	0.00416819	0.004371	1.51E-32	0.00096832	141.093743
rs7416991	T	C	T	C	0.05162	-0.00070859	0.3162	0.4599	0.8653	0.00416819	0.004405	1.04E-31	0.00094247	137.323282
rs7416991	T	G	A	G	-0.05192	-0.00077035	0.6967	0.6996	0.8653	0.004531494	0.004371	1.51E-32	0.00096832	141.093743
rs7416991	T	C	T	C	0.05162	-0.00077035	0.3162	0.6996	0.8653	0.004531494	0.004405	1.04E-31	0.00094247	137.323282
rs7742789	C	T	C	T	0.05486	-0.0009701	0.6933	0.6675	0.8258	0.004409556	0.004413	1.76E-35	0.00106051	154.541012
rs77759957	A	G	A	G	0.0746	-0.02165699	0.9728	0.9834	0.1829	0.016259002	0.01319	1.54E-08	0.0002197	31.9880719
rs7797740	A	G	A	G	-0.03551	0.007958218	0.1499	0.06916	0.3311	0.008187467	0.006093	5.61E-09	0.00023328	33.9655763
rs78433554	C	A	C	A	0.1049	-0.00220287	0.986	0.0026	0.9567	0.040793879	0.0192	4.69E-08	0.00020502	29.8502875
rs7850067	A	G	A	G	0.04308	-0.00045392	0.485	0.601	0.9149	0.004242215	0.004081	4.79E-26	0.00076492	111.434123
rs7867868	C	T	C	T	-0.04157	-0.00237098	0.6695	0.2512	0.6209	0.004789864	0.004504	2.69E-20	0.00058484	85.1850309
rs7894336	C	T	C	T	0.06273	0.005603704	0.4679	0.4833	0.1778	0.004157051	0.004011	4.00E-55	0.00167744	244.593691
rs7946549	T	A	T	A	0.04303	0.003633743	0.5441	0.6692	0.4105	0.004415241	0.004036	1.52E-26	0.00078025	113.668565
rs8068318	C	T	C	T	-0.08407	0.003852625	0.3015	0.2848	0.4024	0.004602898	0.004691	8.01E-72	0.00220153	321.182023
rs9897596	T	C	T	C	-0.02319	-0.00544021	0.4911	0.3463	0.2126	0.004366143	0.004061	1.13E-08	0.00022396	32.6088526

Potassium

SNP	effect_allel	other_allel	effect_allel	other_allele.out	beta.exposure	beta.outcome	eaf.exposure	eaf.outcome	pval.outcome	se.outcome	se.exposure	pval.exposure	R2	Fstat
rs10032927	A	T	A	T	0.01561	0.006103577	0.5837	0.5454	0.1435	0.004171959	0.002494	4.01E-10	6.49E-05	39.175352
rs10046175	C	A	C	A	0.03717	0.000408611	0.9659	0.1594	0.9426	0.005675152	0.006432	7.72E-09	5.59E-05	33.3958922

rs10086982	G	A	G	A	-0.02034	0.001569112	0.7603	0.1797	0.7717	0.005410729	0.003195	2.01E-10	6.71E-05	40.5284666
rs10227075	T	C	T	C	-0.06498	-0.01286487	0.9796	0.9051	0.06951	0.007088083	0.00832	6.01E-15	0.00010209	60.9976019
rs10264133	C	T	C	T	-0.05002	-0.00608275	0.9737	0.1222	0.3378	0.006342803	0.007193	3.67E-12	8.09E-05	48.3578801
rs10265221	T	C	T	C	-0.03723	-0.00973298	0.7481	0.8324	0.08011	0.0055617	0.002886	5.03E-38	0.00027556	166.415364
rs10270510	G	C	G	C	-0.06498	0.012864871	0.9796	0.9051	0.06951	0.007088083	0.00832	6.01E-15	0.00010209	60.9976019
rs10279895	A	G	A	G	-0.06565	-0.01295928	0.9797	0.9056	0.06819	0.007104867	0.008331	3.44E-15	0.00010393	62.0976538
rs10502917	T	C	T	C	-0.02136	-0.00222719	0.3194	0.4927	0.5918	0.004155202	0.002766	1.20E-14	9.88E-05	59.6345773
rs10748853	T	C	T	C	-0.03616	-0.00237267	0.1179	0.3425	0.5878	0.004377616	0.003902	2.01E-20	0.00014222	85.8780775
rs1076485	C	T	C	T	0.02448	0.003796816	0.815	0.3309	0.3896	0.004414902	0.00308	2.01E-15	0.00010462	63.1715298
rs10811662	G	A	G	A	0.01906	0.014374714	0.8401	0.1115	0.02937	0.00659996	0.003305	8.20E-09	5.51E-05	33.2585158
rs11124938	C	A	C	A	-0.02548	0.004684315	0.7812	0.1522	0.4181	0.005783105	0.002987	1.57E-17	0.00012051	72.7659832
rs111375249	G	T	G	T	-0.05498	-0.00123635	0.9749	0.1138	0.8498	0.006541529	0.007261	3.84E-14	9.60E-05	57.334575
rs111607733	C	A	C	A	-0.05535	-0.00186239	0.9726	0.1255	0.7665	0.006270677	0.006865	7.85E-16	0.0001088	65.0061083
rs111724190	T	C	T	C	-0.03147	0.002896882	0.9503	0.7653	0.5545	0.004901661	0.005554	1.49E-08	5.37E-05	32.1056698
rs111972532	T	C	T	C	0.03879	-0.00279178	0.8139	0.8332	0.6161	0.005572412	0.003086	3.39E-36	0.00026162	157.996591
rs11217192	T	G	T	G	-0.01709	0.00182763	0.7648	0.7937	0.7218	0.005133793	0.002949	7.04E-09	5.56E-05	33.584167
rs11245343	T	C	T	C	-0.02401	0.002480918	0.7283	0.8779	0.6958	0.00634506	0.002872	6.53E-17	0.00011575	69.8900657
rs11264363	G	C	G	C	0.0252	0.012299148	0.4285	0.2191	0.01432	0.00502211	0.002659	2.78E-21	0.00014874	89.8182123
rs112734474	T	G	T	G	-0.07519	0.002976754	0.9782	0.9015	0.6695	0.00697132	0.008227	6.70E-20	0.00013979	83.5289794
rs114048605	C	T	C	T	-0.1319	-0.00663581	0.9894	0.0149	0.6984	0.017146789	0.02131	6.28E-10	0.00021771	38.3109297
rs114323080	G	A	G	A	-0.1018	-0.01018802	0.9864	0.0192	0.5012	0.015138215	0.01812	1.99E-08	0.00017937	31.563065
rs115080005	C	T	C	T	0.03996	-0.00133478	0.9722	0.1313	0.8283	0.006151046	0.00691	7.55E-09	5.60E-05	33.4422019
rs115223381	T	C	T	C	-0.1006	-0.00599474	0.9865	0.9808	0.6923	0.015138235	0.01814	3.03E-08	0.00017478	30.7554011
rs11563587	T	A	T	A	-0.05898	0.007661503	0.9824	0.9178	0.3111	0.007563181	0.00864	9.02E-12	7.80E-05	46.5995853
rs11563956	C	T	C	T	-0.03679	0.001322869	0.9677	0.1478	0.8211	0.005853401	0.006511	1.64E-08	5.34E-05	31.9274464
rs11563967	A	G	A	G	-0.03819	-0.00179931	0.9514	0.7762	0.7178	0.004984244	0.005682	1.85E-11	7.56E-05	45.1748644
rs11563979	G	A	G	A	-0.04055	-0.0004074	0.9527	0.2182	0.9351	0.005029689	0.00576	1.98E-12	8.29E-05	49.5606222
rs11563980	C	T	C	T	-0.03382	-0.00203994	0.9393	0.2514	0.6699	0.004788599	0.005234	1.07E-10	6.99E-05	41.752239
rs11563986	T	C	T	C	-0.04416	0.003141073	0.9695	0.8585	0.5985	0.005960291	0.006736	5.74E-11	7.19E-05	42.978769
rs11563990	C	T	C	T	-0.03834	0.000412161	0.9635	0.167	0.9408	0.005569749	0.006325	1.38E-09	6.15E-05	36.7437229
rs11564010	G	C	G	C	-0.06314	0.012942838	0.9796	0.9051	0.06784	0.007088082	0.008287	2.66E-14	9.72E-05	58.0516379
rs11564019	G	A	G	A	-0.06503	-0.01284763	0.9796	0.0946	0.0703	0.007098138	0.008322	5.79E-15	0.0001022	61.0621491
rs11564024	T	G	T	G	-0.0652	-0.01283058	0.9299	0.9012	0.06534	0.00696179	0.008369	6.98E-15	0.00034486	60.6943208
rs11564025	T	G	T	G	-0.06489	-0.01275962	0.9795	0.905	0.07174	0.007084745	0.008315	6.31E-15	0.00010193	60.901928
rs11564194	C	T	C	T	-0.02113	0.00579313	0.8063	0.1157	0.3726	0.00649454	0.003081	7.27E-12	7.79E-05	47.0343988
rs116232574	A	G	A	G	-0.1002	-0.0096695	0.9863	0.9806	0.5211	0.015061519	0.01806	2.96E-08	0.00017493	30.7822209
rs116355131	C	A	C	A	0.06558	-0.00470299	0.9709	0.0414	0.6521	0.010427906	0.01191	3.76E-08	0.0001723	30.3193092
rs11711982	T	C	T	C	0.0239	0.018304274	0.883	0.9458	0.04599	0.009175075	0.003863	6.33E-10	6.34E-05	38.2777486
rs11743019	A	G	A	G	-0.02052	-0.01026288	0.7361	0.9343	0.221	0.008384703	0.002981	6.04E-12	7.85E-05	47.3838954
rs11822294	C	T	C	T	-0.01797	-0.00820123	0.8251	0.2125	0.1064	0.005078164	0.003169	1.47E-08	5.33E-05	32.1552341
rs11970777	C	T	C	T	-0.03918	-0.00547078	0.9638	0.1699	0.3229	0.005531624	0.006303	5.25E-10	6.40E-05	38.6397448
rs11974568	T	C	T	C	-0.03409	-0.00937338	0.9488	0.7622	0.05477	0.00487943	0.005567	9.44E-10	6.21E-05	37.4982983
rs11983963	T	A	T	A	-0.04007	0.004246719	0.9647	0.8338	0.4467	0.005580445	0.00636	3.08E-10	6.57E-05	39.6939624
rs1214759	A	G	A	G	-0.01681	0.000919772	0.4192	0.6831	0.8371	0.004464913	0.002573	6.59E-11	7.07E-05	42.6830902
rs12378991	G	A	G	A	-0.03902	0.017823998	0.9411	0.0165	0.2746	0.016307409	0.005503	1.39E-12	8.33E-05	50.2777118
rs12509595	T	C	T	C	0.03557	-0.00333358	0.7552	0.9148	0.6538	0.007441031	0.002892	9.95E-35	0.0002505	151.27639
rs12549572	T	C	T	C	0.02422	0.001967902	0.6728	0.4105	0.6415	0.004222965	0.002648	6.11E-20	0.00013855	83.658932
rs1273886	A	G	A	G	0.02013	0.01758801	0.6749	0.9203	0.02184	0.007670305	0.002776	4.30E-13	8.71E-05	52.5833954
rs1275979	T	C	T	C	-0.03297	-0.00018201	0.4719	0.7849	0.9712	0.00505579	0.002568	1.09E-37	0.00027294	164.834401
rs12766550	C	G	C	G	0.02881	0.029671464	0.9417	0.96	0.005125	0.010600737	0.005162	2.44E-08	5.16E-05	31.1494596
rs12803281	C	T	C	T	-0.01661	-0.00264696	0.5864	0.2645	0.5742	0.004709899	0.002465	1.66E-11	7.52E-05	45.4051817
rs12940197	G	T	G	T	-0.02288	0.000168223	0.4361	0.2285	0.9727	0.004947722	0.002976	1.55E-14	9.79E-05	59.1079894
rs12959198	T	C	T	C	-0.02103	-0.00247013	0.7725	0.6878	0.5816	0.004482995	0.002958	1.22E-12	8.37E-05	50.5454661
rs12975656	G	A	G	A	0.02031	-0.00018099	0.5826	0.1269	0.9766	0.006240991	0.0027	5.53E-14	9.37E-05	56.5838272
rs13042148	C	T	C	T	-0.02296	-0.00532653	0.8834	0.028	0.6721	0.012592266	0.004082	1.91E-08	5.24E-05	31.6371801
rs13072118	C	T	C	T	0.0161	-0.00133923	0.4291	0.2317	0.7855	0.004923657	0.002608	6.93E-10	6.31E-05	38.109792
rs13072590	G	A	G	A	0.01745	0.001620569	0.786	0.0551	0.8588	0.009104321	0.003135	2.69E-08	5.13E-05	30.9824511
rs13333693	A	G	A	G	0.02753	-0.00469674	0.7554	0.7137	0.3068	0.004595639	0.00284	3.46E-22	0.00015561	93.9670824
rs1336173	T	A	T	A	0.04684	0.006331563	0.9711	0.8676	0.3016	0.006129296	0.007667	1.03E-09	6.25E-05	37.3235415
rs138278364	C	A	C	A	-0.0861	-0.02511334	0.981	0.0273	0.04879	0.01274789	0.01445	2.63E-09	0.00020175	35.5034542
rs138304526	T	C	T	C	0.1353	0.033460672	0.9882	0.9834	0.03957	0.01625883	0.02115	1.63E-10	0.00023255	40.923696
rs138404919	C	T	C	T	-0.1012	-0.00998648	0.9865	0.0191	0.5108	0.01517702	0.0181	2.29E-08	0.00017765	31.2610726
rs138531692	G	A	G	A	-0.0861	-0.02511334	0.981	0.0273	0.04879	0.01274789	0.01445	2.63E-09	0.00020175	35.5034542
rs139145497	T	C	T	C	-0.086	-0.02448135	0.9811	0.9728	0.05526	0.012770657	0.01441	2.46E-09	0.00020241	35.6179517
rs139316656	G	A	G	A	-0.0861	-0.02511334	0.981	0.0273	0.04879	0.01274789	0.01445	2.63E-09	0.00020175	35.5034542
rs139777921	T	A	T	A	-0.08715	0.022418592	0.9828	0.9753	0.09395	0.013384234	0.01523	1.09E-08	0.00018608	32.7442446
rs140318611	G	A	G	A	-0.08728	-0.02269094	0.9829	0.0246	0.09064	0.013410719	0.01523	1.03E-08	0.00018663	32.8420054
rs140501563	G	A	G	A	-0.08845	-0.02549871	0.982	0.0258	0.05162	0.013103139	0.01484	2.61E-09	0.00020187	35.5244917
rs141016776	C	T	C	T	-0.08603	-0.02445047	0.981	0.0273	0.05506	0.012747901	0.01438	2.23E-09	0.00020339	35.7916792
rs141017725	G	A	G	A	-0.08644	-0.02466013	0.9811	0.0272	0.05348	0.012770654	0.01439	1.93E-09	0.00020505	36.0834395
rs141935404	C	T	C	T	-0.02551	-0.00519632	0.9136	0.0814	0.4941	0.007596964	0.004305	3.21E-09	5.82E-05	35.1135392
rs142365336	G	T	G	T	0.1224	0.035396157	0.986	0.0196	0.01817	0.014985672	0.01888	9.41E-11	0.00023883	42.0299483
rs1427298	C	T	C	T	-0.01837	-0.00587766	0.6201	0.195	0.2622	0.005243228	0.002624	2.67E-12	8.12E-05	49.0106713
rs142878005	T	C	T	C	-0.08632	-0.02441223	0.9811	0.9727	0.0555	0.012747902	0.01439	2.03E-09	0.00020448	35.9833237
rs142887200	C	T	C	T	-0.06546	-0.01284865	0.9795	0.0947	0.07019	0.007094781	0.008327	3.98E-15	0.00010343	61.7980644
rs143341259	T	C	T	C	0.04981	0.016316939	0.9743	0.9934	0.5246	0.025655564	0.008639	8.33E-09	5.56E-05	33.2436536
rs143862634	T	G	T	G	-0.08676	-0.02240121	0.9828	0.9752	0.09346	0.013357907	0.01513	9.98E-09	0.00018686	32.8822276
rs144261869	A	G	A	G	0.03665	0.001026655	0.9654	0.8382	0.8553	0.005640963	0.006401	1.06E-08	5.49E-05	32.7832725
rs144726326	C	T	C	T	-0.0398	-0.00286686	0.9534	0.2161	0.5698	0.005047288	0.005839	9.65E-12	7.78E-05	46.4610685
rs144808502	G	A	G	A	-0.08486	-0.02266849	0.9817	0.0262	0.08137	0.013005443	0.01482	1.06E-08	0.00018632	32.7876015
rs144843179	G	A	G	A	0.03096	0.003372858	0.7948	0.2851	0.4636	0.004601444	0.002895	1.15E-26	0.00018939	114.368064
rs145391254	C	T	C	T	0.03637	-0.0004678	0.9666	0.1527	0.9351	0.005775349	0.006427	1.57E-08	5.36E-05	32.0235889
rs145557956	A	G	A	G	-0.08581	-0.0239108	0.9809	0.9726	0.06018	0.01272528	0.0143	2.02E-09	0.00020462	36.0083921
rs146176062	C	T	C	T	-0.08591	-0.02420347	0.9809	0.0274	0.05721	0.012725275	0.01438	2.35E-09	0.00020283	35.6918999
rs146416293	C	G	C	G	-0.0861	-0.02511334	0.981	0.9727	0.04879	0.01274789	0.01445	2.63E-09	0.00020175	35.5034542
rs146745107	T	C	T	C	-0.08633	-0.02419552	0.981	0.9727	0.05771	0.012747905	0.0144	2.07E-09	0.00020424	35.9416903
rs147888747	A	C	A	C	-0.08684	-0.02350269	0.9828	0.9753	0.07917	0.013384218	0.01531	1.45E-08	0.00018283	32.1728288
rs148160104	A	G	A	G	-0.0861	-0.02511334	0.981	0.9727	0.04879	0.01274789	0.01445	2.63E-09	0.00020175	35.5034542
rs148227305	G	A	G	A	-0.08581	-0.0239108	0.9809	0.0274	0.06018	0.01272528	0.0143	2.02E-09	0.00020462	36.0083921
rs148768978	G	A	G	A	-0.08633	-0.02419552	0.981	0.0273	0.05771	0.012747905	0.0144	2.07E-09	0.00020424	35.9416903
rs149474053	T	C	T	C	0.1332	0.040446764	0.9887	0.9842	0.0152	0.016658469	0.02144	5.38E-10	0.00021933	38.5974744
rs149674884	G	A	G	A	0.0641	0.020302629	0.9555	0.0624	0.01807	0.008588252	0.01031	5.19E-10	0.00021966	38.6543815
rs149727168	A	T	A	T	-0.08581	-0.0239108	0.9809	0.9726	0.06018	0.01272528	0.0143	2.02E-09	0.00020462	36.0083921
rs149923520	C	T	C	T	-0.0861	-0.02511334	0.981	0.0273	0.04879	0.01274789	0.01445	2.63E-09	0.00020175	35.5034542
rs149925191	G	T	G	T	0.03863	-0.00307164	0.9729	0.1274	0.6219	0.00623051	0.006972	3.08E-08	5.14E-05	30.6997373
rs1502282	G	C	G	C	-0.01458	-0.00468376	0.5896	0.5384	0.261	0.004167049	0.0025	5.62E-09	5.63E-05	34.012224
rs150305470	C	G	C	G	-0.08544	-0.02200712	0.9818	0.9739	0.09125	0.013029675	0.01476	7.23E-09	0.00019042	33.508097
rs150464897	G	A	G	A	-0.08522	-0.02422102	0.981	0.0273	0.05737	0.012747905	0.01436	2.99E-09	0.00020014	35.2187696
rs150589717	G	A	G	A	-0.1405	-0.00085766	0.9888	0.0155	0.9595	0.016816773	0.02043	6.45E-12	0.00026874	47.2950746
rs1529897	T	G	T	A	-0.01533	0.05095511	0.5376	0.9977	0.2401	0.043366051	0.002553	1.97E-09	5.97E-05	36.0564263
rs1529897	T	G	T	A	-0.01533	0.005333129	0.5376	0.2987	0.2401	0.004538833	0.002553	1.97E-09	5.97E-05	36.0564263
rs1551287	C	T	C	T	0.02749	-0.00166065	0.2156	0.7546	0.7305	0.00482748	0.00299	4.04E-20	0.00013999	84.529267
rs1574817	T	G	T	G	0.01733	-0.00322096	0.3903	0.6383	0.4563	0.004323432	0.002612	3.35E-11	7.37E-05	44.0200752
rs1609829	T	C	T	C	-0.01753	0.000709363	0.293	0.2781	0.8786	0.004636362	0.002702	9.06E-11	6.97E-05	42.0913779
rs164746	G	A	G	A	-0.02005	0.004439064	0.6178	0.184	0.4075	0.005361189	0.002597	1.22E-14	9.87E-05	59.6052968
rs17038613	C	T	C	T	0.1229	0.035577063	0.9859	0.0197	0.0173	0.014948346	0.01885	7.24E-11	0.00024156	42.5090165
rs17038648	C	T	C	T	0.1127	0.031395943	0.9767	0.0326	0.007279	0.011697445	0.01432	3.74E-15	0.00035192	61.9386587
rs17314270	T	C	T	C	0.1032	0.019544609	0.9746	0.9642	0.08045	0.011181127	0.01385	9.40E-14	0.00031547	55.5213283

rs17367435	T	C	T	C	-0.01507	0.008131637	0.5555	0.8991	0.2384	0.006897063	0.002667	1.63E-08	5.29E-05	31.9286439
rs17390839	G	A	G	A	0.02233	0.001459356	0.837	0.039	0.8916	0.010730559	0.003495	1.73E-10	6.76E-05	40.820948
rs17472490	G	A	G	A	-0.04054	-0.00312451	0.9654	0.159	0.582	0.005680927	0.006389	2.28E-10	6.74E-05	40.2625904
rs17472728	G	T	G	T	0.05347	-0.00750593	0.9855	0.0671	0.3659	0.008303019	0.009379	1.22E-08	5.44E-05	32.5017912
rs17472899	A	G	A	G	-0.05945	-0.00778033	0.9824	0.9181	0.3042	0.007575782	0.008667	7.20E-12	7.87E-05	47.0507039
rs17473032	T	C	T	C	-0.06074	-0.00839105	0.9824	0.9182	0.2683	0.007579992	0.008775	4.65E-12	8.02E-05	47.9132163
rs17473487	C	T	C	T	-0.06575	-0.01280261	0.9303	0.0982	0.06667	0.006980704	0.008394	4.99E-15	0.00034861	61.355512
rs17473690	G	T	G	T	-0.0628	-0.01244376	0.9811	0.0872	0.09094	0.007363172	0.008636	3.70E-13	8.85E-05	52.8803134
rs17473844	T	C	T	C	-0.04674	0.000376319	0.9708	0.8645	0.9507	0.006069658	0.006952	1.85E-11	7.57E-05	45.2020248
rs17473985	G	A	G	A	-0.03361	-0.01010494	0.9494	0.2355	0.03899	0.004895806	0.005585	1.83E-09	6.00E-05	36.2151818
rs17501084	A	G	A	G	-0.05527	-0.00906273	0.9818	0.9151	0.224	0.007452903	0.008478	7.30E-11	7.11E-05	42.5003107
rs17501559	A	G	A	G	0.05322	-0.00703469	0.9853	0.9326	0.3961	0.008285856	0.009367	1.37E-08	5.40E-05	32.2811279
rs17502552	G	T	G	T	-0.06301	-0.01292669	0.9796	0.0947	0.06851	0.007094779	0.008296	3.21E-14	9.65E-05	57.6874673
rs17502782	C	A	C	A	-0.0337	-0.00950555	0.9507	0.2291	0.05452	0.004943083	0.005628	2.18E-09	5.94E-05	35.8551566
rs17502922	C	G	C	G	0.06535	-0.00465085	0.9708	0.9586	0.6553	0.010427906	0.0119	4.07E-08	0.00017138	30.1576336
rs17502970	C	A	C	A	-0.04408	0.003516571	0.9695	0.1415	0.5552	0.005960289	0.006699	4.86E-11	7.25E-05	43.2975394
rs1757225	A	G	A	G	-0.02184	-0.00196616	0.3802	0.6034	0.6432	0.004246557	0.002547	1.05E-17	0.00012177	73.52709
rs185372147	T	G	T	G	-0.06575	-0.01280261	0.9303	0.9018	0.06667	0.006980704	0.008394	4.99E-15	0.00034861	61.355512
rs190945315	A	C	A	C	-0.06543	-0.01350152	0.9302	0.9017	0.05297	0.006977527	0.008378	6.02E-15	0.00034655	60.9920357
rs191079	T	C	T	C	0.01581	0.004883818	0.5793	0.1942	0.3525	0.005251417	0.002839	2.62E-08	5.14E-05	31.012227
rs191102591	G	T	G	T	-0.04406	0.003565305	0.9695	0.1414	0.5501	0.005962049	0.006699	4.98E-11	7.24E-05	43.2582584
rs1979845	C	T	C	T	0.03115	0.008446809	0.8267	0.4774	0.04222	0.004158941	0.003306	4.62E-21	0.00014702	88.7790316
rs1984669	C	T	C	T	-0.0221	0.005374506	0.7592	0.2085	0.2931	0.005113707	0.002834	6.66E-15	0.00010071	60.8113795
rs1989061	T	C	T	C	0.01826	-0.00394138	0.3219	0.153	0.4945	0.005770694	0.002709	1.62E-11	7.52E-05	45.4342681
rs199680901	C	T	C	T	-0.01837	9.19E-05	0.6385	0.1801	0.9862	0.005406038	0.002631	3.01E-12	8.07E-05	48.7502241
rs1997596	C	T	C	T	-0.01956	0.004164887	0.666	0.3404	0.3419	0.004384091	0.002733	8.62E-13	8.48E-05	51.2222248
rs2050542	T	C	T	C	0.04403	-0.001036	0.9731	0.8731	0.8684	0.00624099	0.007712	1.16E-08	5.46E-05	32.5959264
rs210139	A	C	A	C	-0.01598	-0.00127671	0.5911	0.5217	0.7587	0.004158681	0.0024	2.89E-11	7.34E-05	44.3334028
rs2168785	C	T	C	T	0.01863	0.006432763	0.3675	0.2791	0.1649	0.004631219	0.002684	4.09E-12	7.98E-05	48.1793223
rs2195880	C	G	C	G	0.01569	0.002678989	0.7004	0.8138	0.6155	0.005336631	0.002629	2.46E-09	5.90E-05	35.6176051
rs219791	T	G	T	G	0.0173	0.002710006	0.6668	0.396	0.5235	0.004247658	0.002688	1.26E-10	6.86E-05	41.4222492
rs2207404	G	A	G	A	-0.0484	-0.00211196	0.9636	0.1679	0.7038	0.005557801	0.006281	1.35E-14	9.83E-05	59.3790352
rs2255390	A	G	A	G	0.02748	0.001582653	0.4329	0.3924	0.7099	0.004254443	0.002426	1.05E-29	0.00021247	128.307447
rs2327429	T	C	T	C	-0.02284	-0.00298699	0.737	0.9048	0.6727	0.007078174	0.002788	2.65E-16	0.00011115	67.1130012
rs2340534	G	A	G	A	0.01826	0.002315511	0.5376	0.2885	0.6134	0.004585171	0.002458	1.15E-13	9.14E-05	55.1871275
rs2497318	C	T	C	T	-0.02938	2.82E-05	0.6293	0.1621	0.9957	0.00563675	0.002633	6.77E-29	0.00020618	124.509321
rs2569882	T	C	T	C	-0.01474	-0.00257235	0.5253	0.3866	0.5464	0.004265922	0.002462	2.21E-09	5.94E-05	35.8441982
rs2761244	T	C	T	C	0.01525	-0.00028787	0.3969	0.3726	0.9465	0.004296578	0.002484	8.53E-10	6.24E-05	37.6908997
rs2823263	T	C	T	C	-0.02564	0.003486967	0.7425	0.849	0.5479	0.005801942	0.002846	2.17E-19	0.00013442	81.1645248
rs28398484	C	G	C	G	-0.04176	0.002027175	0.9686	0.8556	0.7319	0.005910132	0.006668	3.89E-10	6.56E-05	39.2220056
rs28398495	A	G	A	G	-0.03906	-0.00099029	0.9512	0.7752	0.8419	0.004976354	0.005686	6.69E-12	7.90E-05	47.1900858
rs28398500	C	G	C	G	-0.0384	-0.00164402	0.9513	0.7759	0.7416	0.00498187	0.005685	1.49E-11	7.64E-05	45.6224564
rs28398507	G	A	G	A	-0.03811	-0.00203487	0.9515	0.2234	0.6831	0.004987418	0.005688	2.15E-11	7.51E-05	44.8909423
rs28398535	T	C	T	C	-0.03384	-0.00278373	0.9598	0.8153	0.6032	0.005353326	0.005987	1.62E-08	5.35E-05	31.9478909
rs28398544	A	G	A	G	-0.03329	-0.00234473	0.9595	0.8142	0.6603	0.005341062	0.005932	2.04E-08	5.27E-05	31.4938174
rs28416181	T	G	T	G	0.02165	0.006367605	0.71	0.5712	0.1292	0.004197499	0.002647	3.04E-16	0.00011079	66.8971966
rs28430881	C	A	C	A	-0.02156	0.004651821	0.5611	0.3557	0.2837	0.004339385	0.002429	7.34E-19	0.00013047	78.7848084
rs28446035	C	T	C	T	0.03696	0.000713982	0.9657	0.16	0.8998	0.005666524	0.006433	9.40E-09	5.52E-05	33.0093386
rs28558845	G	C	G	C	-0.02063	-0.00442824	0.82	0.7276	0.3427	0.004666215	0.003066	1.76E-11	7.50E-05	45.2745513
rs28570591	G	A	G	A	-0.06608	-0.01373735	0.9798	0.0938	0.05381	0.007125182	0.008333	2.30E-15	0.00010524	62.8835867
rs3176466	C	T	C	T	0.04031	0.014640557	0.8668	0.3057	0.001168	0.004508949	0.003543	5.89E-30	0.00021435	129.444407
rs34070447	A	G	A	G	0.02013	0.006043255	0.5474	0.4183	0.1513	0.004211328	0.00238	2.89E-17	0.00011847	71.5374797
rs34136790	C	T	C	T	0.02587	0.001018702	0.8161	0.6356	0.8134	0.004316534	0.004021	1.29E-10	6.93E-05	41.3927911
rs34917191	T	C	T	C	-0.01332	-0.01078835	0.4203	0.5754	0.01026	0.004202707	0.002409	3.32E-08	5.06E-05	30.5727743
rs35444	A	G	A	G	-0.0336	-0.00555247	0.6131	0.5912	0.1889	0.00422562	0.00241	3.97E-44	0.00032184	194.376819
rs35619052	G	A	G	A	0.02036	0.003021654	0.7815	0.0591	0.7316	0.008809488	0.003609	1.73E-08	5.33E-05	31.8259798
rs35619990	C	T	C	T	-0.01762	0.002090545	0.5513	0.3469	0.6322	0.004364394	0.002433	4.59E-13	8.69E-05	52.447838
rs35812759	A	G	A	G	0.01552	-0.00083361	0.7522	0.705	0.855	0.004555234	0.002755	1.79E-08	5.26E-05	31.735126
rs36092406	G	A	G	A	-0.04798	-0.00246415	0.9635	0.1685	0.6572	0.005549897	0.006277	2.19E-14	9.68E-05	58.4273555
rs3751767	T	C	T	C	-0.01723	-0.00577712	0.6902	0.7412	0.2233	0.004743116	0.002891	2.59E-09	5.88E-05	35.5201157
rs3790604	C	A	C	A	0.04697	-0.00853153	0.9322	0.0174	0.5911	0.0158874	0.004789	1.12E-22	0.0001593	96.1947668
rs3802177	G	A	G	A	0.01547	0.015383909	0.7385	0.0935	0.03111	0.007135394	0.00278	2.70E-08	5.13E-05	30.9664225
rs3848132	T	A	T	A	-0.03504	0.001976176	0.7463	0.8292	0.7206	0.005520044	0.002922	4.26E-33	0.00023812	143.802942
rs4114858	A	G	A	G	0.01947	-4.99E-05	0.8371	0.5131	0.9907	0.004156191	0.003459	1.87E-08	5.25E-05	31.683327
rs4413681	G	C	G	C	0.06552	0.005099245	0.9709	0.9586	0.6249	0.010427904	0.01196	4.44E-08	0.00017055	30.0113422
rs4461961	C	T	C	T	0.01818	0.005996157	0.6471	0.6786	0.1777	0.004448188	0.002611	3.43E-12	8.03E-05	48.4812729
rs4485922	A	G	A	G	-0.01442	-0.00778107	0.46	0.3343	0.07723	0.004403551	0.002442	3.56E-09	5.78E-05	34.8689766
rs4677143	A	G	A	G	0.01596	-0.00142416	0.6379	0.7445	0.7651	0.004763086	0.002609	9.70E-10	6.20E-05	37.4211919
rs4737371	G	A	G	A	-0.02244	0.00576816	0.8207	0.1771	0.289	0.005441661	0.003276	7.74E-12	7.77E-05	46.9199909
rs4745804	T	C	T	C	-0.01661	-0.00300807	0.5313	0.4977	0.4692	0.004154799	0.002577	1.20E-10	6.88E-05	41.5442026
rs4766578	T	A	T	A	0.01805	-0.00247402	0.3993	0.08663	0.7373	0.007385138	0.002668	1.39E-11	7.58E-05	45.7701949
rs4809849	C	T	C	T	0.01779	0.004971502	0.4172	0.8536	0.3977	0.00587648	0.002675	3.03E-11	7.33E-05	44.2287152
rs4846476	G	C	G	C	0.01665	0.003633052	0.7784	0.8115	0.4939	0.00531148	0.002851	5.38E-09	5.65E-05	34.1062555
rs4848713	C	T	C	T	0.02442	-0.00645854	0.8902	0.0309	0.5904	0.012004727	0.004187	5.65E-09	5.63E-05	34.0161688
rs4867732	C	G	C	G	0.02908	-0.00082929	0.9196	0.9736	0.9486	0.012957586	0.004584	2.30E-10	6.67E-05	40.243856
rs4924538	A	T	A	T	-0.01606	-0.00316923	0.5128	0.5713	0.4504	0.004197652	0.002619	8.81E-10	6.23E-05	37.6027914
rs4936409	A	G	A	G	-0.01693	-0.00917131	0.491	0.302	0.04264	0.004524572	0.002558	3.75E-11	7.25E-05	43.8039058
rs547340372	G	A	G	A	-0.05554	-0.01324918	0.976	0.1098	0.04616	0.006644525	0.007827	1.33E-12	8.43E-05	50.3525052
rs55650455	T	C	T	C	-0.01947	0.003405585	0.8204	0.9253	0.6667	0.007901589	0.003288	3.26E-09	5.81E-05	35.0645513
rs56218647	C	A	C	A	0.02388	-0.00447079	0.9123	0.0686	0.5864	0.008218369	0.004155	9.36E-09	5.47E-05	33.0313701
rs56237637	T	A	T	A	0.04136	0.007607817	0.9645	0.8387	0.178	0.005647971	0.007107	6.08E-09	5.67E-05	33.8679125
rs56329049	G	A	G	A	-0.04065	0.000739678	0.9643	0.1496	0.899	0.005824235	0.006417	2.45E-10	6.65E-05	40.1288784
rs56376587	A	C	A	C	-0.02835	0.002189722	0.5817	0.8033	0.675	0.005226067	0.002564	2.19E-28	0.00020245	122.255973
rs56790613	A	G	A	G	0.0377	0.000924056	0.9673	0.8473	0.8732	0.005775349	0.006576	1.01E-08	5.50E-05	32.8669263
rs569550	T	G	T	G	0.03941	0.013042584	0.6382	0.8317	0.0188	0.005552398	0.002628	8.92E-51	0.00037234	224.885859
rs57420239	C	T	C	T	-0.03706	0.001689745	0.9678	0.1482	0.7724	0.005846868	0.006577	1.80E-08	5.31E-05	31.7508346
rs57484993	A	G	A	G	0.03418	0.002605386	0.9219	0.9815	0.8661	0.015416483	0.004607	1.22E-13	9.12E-05	55.0437093
rs576943137	C	T	C	T	-0.02548	0.003214996	0.8922	0.0293	0.7944	0.012317992	0.004521	1.79E-08	5.26E-05	31.7636084
rs578097	A	G	A	G	-0.02281	0.005132601	0.652	0.5309	0.2176	0.004162694	0.002544	3.25E-19	0.00013314	80.3926522
rs57850577	A	G	A	G	-0.05408	0.000881085	0.9691	0.8581	0.8827	0.005953278	0.006479	7.43E-17	0.0001166	69.6718602
rs57944734	C	T	C	T	-0.04826	-0.0024827	0.9619	0.1749	0.65	0.005468495	0.006162	5.02E-15	0.00010158	61.3382311
rs58320941	G	T	G	T	-0.04411	0.003237394	0.9695	0.1414	0.5869	0.005962051	0.006735	5.96E-11	7.18E-05	42.8942339
rs59482369	T	C	T	C	-0.04242	-0.00260281	0.959	0.8155	0.6273	0.005355571	0.006235	1.05E-11	7.75E-05	46.2879996
rs60085321	C	G	C	G	-0.05059	-0.00524271	0.9688	0.8568	0.3768	0.005930669	0.006686	4.02E-14	9.48E-05	57.2527848
rs6013199	G	A	G	A	-0.07916	-0.01297818	0.9722	0.0383	0.2304	0.010824167	0.01317	1.89E-09	0.0002053	36.1276756
rs6031431	A	G	A	G	0.01961	0.004825113	0.5497	0.5123	0.2458	0.004155998	0.002464	1.81E-15	0.0001049	63.339376
rs60772526	C	T	C	T	0.0605	0.001667077	0.127	0.7733	0.7365	0.004961539	0.003746	1.27E-58	0.00043184	260.840608
rs6108787	T	G	T	G	0.01367	0.001936342	0.5405	0.4924	0.6414	0.00415524	0.002403	1.31E-08	5.36E-05	32.3615629
rs61169316	G	A	G	A	0.01814	-0.00481767	0.7215	0.1348	0.4281	0.006082918	0.002753	4.56E-11	7.19E-05	43.417233
rs61793790	G	C	G	C	-0.03709	-0.00455051	0.9597	0.9527	0.6416	0.009786047	0.006649	2.49E-08	5.21E-05	31.1172351
rs61897793	G	A	G	A	-0.02178	-0.00051547	0.8642	0.0325	0.9653	0.011715186	0.003653	2.56E-09	5.95E-05	35.5481011
rs62193645	C	T	C	T	0.03441	0.008341505	0.8796	0.0271	0.5144	0.012793719	0.003785	1.04E-19	0.00013687	82.6489951
rs62330329	T	C	T	C	0.02703	-0.00072919	0.2697	0.1127	0.9113	0.006569301	0.004425	1.04E-09	6.18E-05	37.3134616
rs62374068	A	G	A	G	0.05943	0.004993502	0.7821	0.8052	0.3411	0.005245276	0.002918	3.67E-92	0.00068657	414.802042
rs62435145	G	T	G	T	-0.0256	-0.00496438	0.434	0.1924	0.346	0.005270041	0.0027	2.68E-21	0.00014888	89.8984911
rs66482211	A	G	A	G	-0.04204	0.016737919	0.9505	0.9441	0.06417	0.009042636	0.005675	1.34E-13	9.09E-05	54.8774197
rs66561220	T	C	T	C	-0.02949	0.003954905	0.7728	0.9333	0.6348	0.008326115	0.003016	1.49E-22	0.00015833	95.6063792
rs6697367	C	T	C	T	-0.01868	0.004983528	0.6768	0.2904	0.2761	0.004576243	0.002575	4.19E-13	8.72E-05	52.6258686

rs6703881	T	C	T	C	-0.02296	-0.00570056	0.1968	0.3317	0.1963	0.004412196	0.00312	1.91E-13	8.97E-05	54.1545036
rs6716091	C	T	C	T	0.02352	-0.00699613	0.7446	0.2418	0.1493	0.004851683	0.002796	4.19E-17	0.00011719	70.7620328
rs6755070	G	T	G	T	0.01832	0.004804345	0.6877	0.2669	0.3062	0.004696329	0.002757	3.10E-11	7.31E-05	44.1547476
rs6794202	C	T	C	T	0.02384	0.02743781	0.7896	0.0767	0.0004392	0.00780592	0.003025	3.39E-15	0.00010286	62.1100307
rs6816915	G	A	G	A	-0.01441	0.006090733	0.4714	0.7103	0.1835	0.004579498	0.002594	2.83E-08	5.11E-05	30.8594384
rs6826742	G	T	G	T	-0.01667	0.004326848	0.4695	0.423	0.3033	0.004204906	0.00243	7.13E-12	7.79E-05	47.0607292
rs6877631	T	C	T	C	-0.0154	-0.00112069	0.6149	0.8138	0.8337	0.005336636	0.002594	2.99E-09	5.84E-05	35.2453233
rs690054	C	T	C	T	-0.03594	-0.00647435	0.5832	0.2058	0.2077	0.005138375	0.002501	8.68E-47	0.00034192	206.50414
rs6934923	G	T	T	C	-0.05549	-0.08828719	0.9748	0.9997	0.462	0.11995542	0.007262	2.25E-14	9.77E-05	58.3871075
rs6934923	G	T	T	C	-0.05549	-0.00479262	0.9748	0.885	0.462	0.006511706	0.007262	2.25E-14	9.77E-05	58.3871075
rs6941546	G	A	G	A	-0.02568	-0.00126598	0.9129	0.1683	0.8199	0.005552529	0.004349	3.63E-09	5.77E-05	34.8667304
rs6961048	C	G	C	G	0.04642	0.015277551	0.8895	0.8731	0.01437	0.00624083	0.003761	5.76E-35	0.00025225	152.336371
rs697976	T	C	T	C	0.01364	0.003900897	0.4779	0.5219	0.348	0.004158739	0.0024	1.34E-08	5.35E-05	32.3002778
rs7038668	T	C	T	C	-0.0279	-0.00613567	0.5014	0.346	0.1599	0.004367026	0.002425	1.38E-30	0.00021919	132.368583
rs71537958	G	C	G	C	-0.01936	0.001655838	0.7389	0.8904	0.8033	0.00664995	0.002891	2.22E-11	7.43E-05	44.845051
rs716877	C	G	C	G	0.03596	-6.85E-05	0.5162	0.843	0.9906	0.005710219	0.002627	1.35E-42	0.00031026	187.378268
rs7187932	G	A	G	A	-0.0223	0.002228206	0.7746	0.3853	0.6016	0.004268593	0.002875	9.15E-15	9.96E-05	60.1636295
rs7218857	T	C	T	C	-0.02113	-0.00053265	0.5618	0.5683	0.8986	0.004194078	0.002471	1.30E-17	0.0001211	73.1229162
rs7222451	G	A	G	A	-0.01388	-0.00594144	0.4975	0.6619	0.1762	0.004391311	0.002471	1.99E-08	5.23E-05	31.5524757
rs72969822	C	G	C	G	-0.0249	-0.00663125	0.8821	0.9059	0.3514	0.00711508	0.003796	5.55E-11	7.13E-05	43.0275172
rs73776711	G	A	G	A	-0.0479	-0.00204527	0.9631	0.1702	0.7113	0.005527766	0.006173	8.96E-15	9.97E-05	60.211365
rs7416991	T	G	A	G	-0.01795	-0.00070859	0.6404	0.4599	0.8653	0.00416819	0.002504	7.97E-13	8.51E-05	51.3878274
rs7416991	T	C	T	C	0.01931	-0.00070859	0.4025	0.4599	0.8653	0.00416819	0.002593	1.01E-13	9.18E-05	55.4574012
rs7416991	T	G	A	G	-0.01795	-0.00077035	0.6404	0.6996	0.8653	0.004531494	0.002504	7.97E-13	8.51E-05	51.3878274
rs7416991	T	C	T	C	0.01931	-0.00077035	0.4025	0.6996	0.8653	0.004531494	0.002593	1.01E-13	9.18E-05	55.4574012
rs74496669	G	T	G	T	0.03972	-0.00172166	0.9698	0.1405	0.7737	0.005977989	0.00716	2.96E-08	5.15E-05	30.7746013
rs74518739	C	T	C	T	-0.04059	-0.00032677	0.9527	0.2185	0.9485	0.0050272	0.005761	1.91E-12	8.31E-05	49.6412092
rs75248620	G	A	G	A	0.03605	-0.00918064	0.9368	0.0239	0.4996	0.013600955	0.00518	3.55E-12	8.02E-05	48.434076
rs75411357	A	T	A	T	0.03822	0.001128209	0.9656	0.8402	0.842	0.005669393	0.006723	1.35E-08	5.41E-05	32.3187937
rs75741381	C	G	C	G	-0.01862	0.00314961	0.7999	0.6439	0.4678	0.004338306	0.003124	2.60E-09	5.88E-05	35.5252631
rs76075621	T	C	T	C	0.03689	0.000380042	0.9658	0.8404	0.9466	0.00567227	0.006443	1.05E-08	5.49E-05	32.7824228
rs761356	T	C	T	C	0.02838	-0.00212124	0.6163	0.7953	0.6807	0.005148629	0.002531	3.76E-29	0.0002082	125.730456
rs76200870	G	A	G	A	-0.05573	-0.00284348	0.9737	0.1195	0.6568	0.006404237	0.007021	2.17E-15	0.00010545	63.0057434
rs76666870	T	C	T	C	0.03689	0.000380042	0.9658	0.8404	0.9466	0.00567227	0.006443	1.05E-08	5.49E-05	32.7824228
rs76821958	A	G	A	G	-0.04048	-0.00910176	0.9734	0.8774	0.1506	0.00633386	0.006957	6.08E-09	5.61E-05	33.8561056
rs76987554	C	T	C	T	-0.07778	0.01038607	0.982	0.0806	0.1736	0.007631205	0.009027	7.31E-18	0.00012425	74.2418854
rs77081469	C	A	C	A	0.06558	-0.00470299	0.9709	0.0414	0.6521	0.010427906	0.01191	3.76E-08	0.0001723	30.3193092
rs7726795	T	C	T	C	0.02854	-8.98E-05	0.6314	0.3099	0.9841	0.004492102	0.002657	7.04E-27	0.00019107	115.378484
rs77290217	T	C	T	C	-0.04754	-0.00414881	0.9649	0.8417	0.4659	0.0056911	0.006553	4.18E-13	8.81E-05	52.6305634
rs7749375	G	A	G	A	-0.05485	-0.00030137	0.975	0.1134	0.9636	0.006551579	0.007327	7.38E-14	9.38E-05	56.0403554
rs7766997	A	G	A	G	-0.05467	-0.00077131	0.9749	0.886	0.9061	0.006536527	0.007245	4.68E-14	9.53E-05	56.940512
rs7767054	G	A	G	A	-0.05415	-7.13E-05	0.9743	0.1164	0.9914	0.006477569	0.00719	5.22E-14	9.49E-05	56.7203812
rs77747253	G	T	G	T	-0.1012	-0.00998648	0.9865	0.0191	0.5108	0.01517702	0.0181	2.29E-08	0.00017765	31.2610726
rs780101	C	T	C	T	0.02158	-0.00288508	0.5339	0.674	0.515	0.004431766	0.002504	7.30E-18	0.000123	74.2735585
rs78132593	C	A	C	A	0.01948	0.01841	0.8381	0.0393	0.08502	0.010691057	0.003462	1.87E-08	5.24E-05	31.6609381
rs7836572	T	C	T	C	0.02144	0.002765489	0.4105	0.2601	0.5594	0.004735426	0.002459	2.91E-18	0.0001259	76.0208121
rs78716205	G	A	G	A	0.0679	0.012501211	0.9669	0.0108	0.5342	0.02009841	0.006869	5.19E-23	0.00016182	97.7130379
rs79030490	C	A	C	A	-0.07534	0.009252445	0.9817	0.082	0.2218	0.007571559	0.008966	4.59E-17	0.00011817	70.607976
rs79285851	C	T	C	T	-0.1018	-0.0104037	0.9864	0.0193	0.4908	0.015099714	0.01811	1.93E-08	0.00017956	31.5979317
rs79392591	C	G	C	G	-0.0245	6.68E-05	0.8683	0.9143	0.9928	0.007421327	0.003564	6.53E-12	7.83E-05	47.2559804
rs79557018	C	T	C	T	-0.05487	-0.00092783	0.9749	0.1141	0.8871	0.00653403	0.007246	3.81E-14	9.60E-05	57.3420561
rs79590186	C	T	C	T	-0.07237	-0.00082691	0.976	0.1089	0.9017	0.006668669	0.007995	1.49E-19	0.00013713	81.9370283
rs79889868	G	C	G	C	-0.05507	-0.00076633	0.9696	0.86	0.8981	0.005986915	0.00659	6.80E-17	0.00011687	69.8327788
rs80176668	A	G	A	G	-0.07368	0.003953727	0.9774	0.8968	0.5625	0.006828544	0.007748	2.05E-21	0.00015134	90.4315574
rs80289662	C	G	C	G	-0.05427	0.000185098	0.9694	0.8591	0.9756	0.005970889	0.006589	1.86E-16	0.00011354	67.839186
rs80328223	C	T	C	T	-0.07624	0.003033801	0.9784	0.0979	0.6643	0.006990324	0.008251	2.62E-20	0.00014289	85.3793022
rs8077544	G	A	G	A	0.01629	-0.00158523	0.3428	0.2277	0.7492	0.004953836	0.00285	1.13E-08	5.41E-05	32.6702493
rs8125560	A	C	A	C	-0.01906	-0.00350048	0.825	0.8649	0.5646	0.006077222	0.003137	1.28E-09	6.11E-05	36.91618
rs820429	G	T	G	T	-0.04348	-0.01407189	0.6755	0.0759	0.07283	0.007843862	0.002787	7.92E-55	0.00040297	243.391347
rs880315	T	C	T	C	0.04161	0.002926172	0.6739	0.8167	0.5857	0.005369122	0.002572	7.72E-59	0.00043332	261.729999
rs923000	A	C	A	C	0.02374	-0.00169011	0.637	0.8804	0.7919	0.006401926	0.002662	4.99E-19	0.00013171	79.5326269
rs9321413	C	G	C	G	0.03641	0.001048435	0.9653	0.8379	0.8527	0.005636749	0.006408	1.36E-08	5.40E-05	32.28467
rs9321414	A	C	A	C	0.03723	0.001424735	0.9642	0.8343	0.7989	0.005587196	0.006659	2.30E-08	5.23E-05	31.2584934
rs9483608	A	G	A	G	0.03662	0.000430113	0.9657	0.8395	0.9396	0.005659375	0.006344	7.99E-09	5.58E-05	33.3204096
rs9483610	G	A	G	A	0.03663	0.000356721	0.9657	0.1603	0.9499	0.00566223	0.00635	8.18E-09	5.57E-05	33.2756377
rs9483614	C	T	C	T	0.03847	0.000462528	0.9663	0.157	0.9352	0.005710219	0.006761	1.30E-08	5.42E-05	32.3759485
rs9493708	C	T	C	T	0.03629	0.000553644	0.9655	0.1612	0.9223	0.00564943	0.006343	1.09E-08	5.48E-05	32.7329023
rs9493713	C	A	C	A	0.03717	0.000408611	0.9659	0.1594	0.9426	0.005675152	0.006432	7.72E-09	5.59E-05	33.3958922
rs9493714	C	T	C	T	0.03664	0.000279563	0.9646	0.1654	0.9602	0.005591257	0.006371	9.05E-09	5.54E-05	33.0746852
rs9493720	T	C	T	C	0.03665	0.001026655	0.9654	0.8382	0.8553	0.005640963	0.006401	1.06E-08	5.49E-05	32.7832725
rs9493722	T	C	T	C	0.03711	0.000357535	0.9659	0.8406	0.9498	0.005675152	0.006433	8.22E-09	5.57E-05	33.2778152
rs9493723	T	C	T	C	0.03665	0.001026655	0.9654	0.8382	0.8553	0.005640963	0.006401	1.06E-08	5.49E-05	32.7832725
rs9493727	A	G	A	G	0.03721	0.001178613	0.9641	0.8342	0.8329	0.005585846	0.006656	2.32E-08	5.23E-05	31.2530721
rs9493728	A	G	A	G	0.03714	0.001173028	0.9642	0.8342	0.8334	0.005585846	0.006656	2.48E-08	5.21E-05	31.1355952
rs9493729	G	T	G	T	0.03978	-0.00166287	0.9699	0.1403	0.7807	0.005981553	0.007166	2.92E-08	5.16E-05	30.8159776
rs9603367	C	T	C	T	0.05097	-0.00562014	0.609	0.1225	0.3753	0.006336118	0.002692	7.22E-80	0.00059342	358.491742
rs963837	T	C	T	C	0.0215	0.006428756	0.6211	0.8757	0.3072	0.006296528	0.002687	1.29E-15	0.00010603	64.0238206
rs9819344	C	T	C	T	0.03573	0.001127061	0.9518	0.2153	0.8238	0.005054084	0.005812	8.11E-10	6.33E-05	37.7933005
rs9821489	G	A	G	A	0.04282	0.006502389	0.8886	0.4956	0.1175	0.004154881	0.004507	2.20E-21	0.00014948	90.2647554
rs9822474	A	G	A	G	-0.01603	-0.00099997	0.5519	0.5376	0.8107	0.004166561	0.00239	2.06E-11	7.45E-05	44.9853644
rs9844949	A	G	A	G	0.01972	-0.0028513	0.152	0.1806	0.5977	0.005400189	0.003549	2.83E-08	5.11E-05	30.8746343
rs9918487	G	A	G	A	-0.05331	0.001020986	0.9685	0.1449	0.8625	0.005901653	0.006354	5.13E-17	0.00011781	70.3919415
rs9928653	T	C	T	C	-0.01848	-0.00291728	0.7631	0.5105	0.4826	0.004155672	0.002844	8.46E-11	6.99E-05	42.2225783

Iron

SNP	effect_allel	other_allel	effect_allel	other_allele.out	beta.exposure	beta.outcome	eaf.exposure	eaf.outcome	pval.outcome	se.outcome	se.exposure	pval.exposure	R2	Fstat
rs10831924	T	C	T	C	0.06204	-0.00788796	0.9131	0.6862	0.07804	0.004476708	0.007982	8.06E-15	0.00047362	60.4115712
rs12328766	A	G	A	G	0.03348	-0.00281466	0.6129	0.5875	0.5047	0.004219875	0.004341	1.29E-14	0.00046634	59.4827895
rs13007705	C	T	C	T	-0.02771	-0.00352952	0.544	0.4382	0.399	0.004186856	0.004194	4.05E-11	0.00034228	43.6532115
rs13081052	T	C	T	C	0.03643	0.000363474	0.248	0.4475	0.9306	0.004177858	0.005395	1.51E-11	0.00035751	45.5969134
rs140393761	A	G	A	G	-0.123	-0.01572563	0.9874	0.997	0.6791	0.037984625	0.0201	9.56E-10	0.00029625	37.4470929
rs150375987	C	G	C	G	-0.1303	-0.00786439	0.9792	0.9943	0.7757	0.027594343	0.01552	4.96E-17	0.00055748	70.4864109
rs1800562	G	A	G	A	-0.3257	0.02739378	0.9509	0.0116	0.158	0.019400694	0.01006	1.05E-229	0.00822663	1048.1889
rs190399027	G	A	G	A	-0.1314	-0.03827156	0.9894	0.0025	0.3577	0.04159952	0.02111	4.98E-10	0.00030651	38.744875
rs1958078	A	C	A	C	-0.03145	4.17E-06	0.2447	0.4534	0.999	0.004172927	0.005338	3.94E-09	0.00027219	34.7123616
rs218248	G	A	G	A	-0.03636	0.008594987	0.8382	0.2186	0.08724	0.005026308	0.00575	2.64E-10	0.00031354	39.9863773
rs2294915	C	T	C	T	-0.03195	-0.0110383	0.7616	0.1761	0.04298	0.005453706	0.005059	2.78E-10	0.00031274	39.8852544
rs28929474	C	T	C	T	-0.1763	-0.00440952	0.9866	0.0031	0.9064	0.037368847	0.0188	7.11E-21	0.00069544	87.9404991
rs555273273	C	T	C	T	0.1873	0.007711503	0.984	0.0194	0.6086	0.015061529	0.03115	1.88E-09	0.00085513	36.154258
rs5995385	T	C	T	C	0.02734	0.003473288	0.5347	0.6327	0.4202	0.00430929	0.004294	1.98E-10	0.00031787	40.5389915
rs72839066	A	G	A	G	-0.1163	-0.01375479	0.9694	0.9936	0.5977	0.02605074	0.01327	1.99E-18	0.00060747	76.8099679
rs7385804	C	A	C	A	-0.05785	-0.0030505	0.357	0.6709	0.4904	0.004421022	0.004417	3.75E-39	0.00134364	171.534677
rs7775698	C	T	C	T	-0.0471	0.004995099	0.7576	0.2151	0.323	0.005055768	0.004874	4.56E-22	0.00073192	93.3836327
rs806970	C	T	C	T	0.05669	0.031703948	0.945	0.0194	0.03526	0.015061258	0.009471	2.22E-09	0.00028344	35.8278907
rs8177252	C	A	C	A	-0.05473	-0.00197343	0.6848	0.2405	0.6848	0.004860653	0.00463	3.25E-32	0.00109478	139.72976
rs855791	A	G	A	G	-0.1527	-0.00243535	0.3768	0.1521	0.674	0.005784677	0.004498	2.09E-252	0.00895869	1152.49532
rs9402686	G	A	G	A	-0.05435	0.003741165	0.7844	0.0725	0.6407	0.00801106	0.005267	6.25E-25	0.00083449	106.481083

Zinc

SNP	effect_allel	other_allel	effect_allel	other_allele.out	beta.exposure	beta.outcome	eaf.exposure	eaf.outcome	pval.outcome	se.outcome	se.exposure	pval.exposure	R2	Fstat
rs10484100	G	A	G	A	-0.209	0.002531739	0.5	0.2107	0.6195	0.005094043	0.045	3.30E-06	0.00822508	21.5708642
rs11763353	G	A	G	A	-0.192	0.003075215	0.5	0.3899	0.4705	0.0042593	0.039	7.00E-07	0.00923219	24.2366864
rs1532423	A	G	A	G	0.178	-0.00123135	0.5	0.191	0.8155	0.005284761	0.026	6.40E-12	0.01770095	46.8698225
rs2120019	C	T	C	T	-0.287	-0.00216558	0.5	0.4538	0.6037	0.00417261	0.033	1.55E-18	0.02825832	75.6372819
rs4333127	A	G	A	G	0.218	0.005051902	0.5	0.06771	0.5409	0.008268253	0.047	3.00E-06	0.00820351	21.5138072
rs7148590	A	G	A	G	-0.14	-0.00629618	0.5	0.3193	0.1576	0.004455898	0.026	1.40E-07	0.01102439	28.9940828

Copper

SNP	effect_allel	other_allel	effect_allel	other_allele.out	beta.exposure	beta.outcome	eaf.exposure	eaf.outcome	pval.outcome	se.outcome	se.exposure	pval.exposure	R2	Fstat
rs10014072	G	A	G	A	-0.164	0.010659198	0.5	0.5568	0.0108	0.004181718	0.034	1.00E-06	0.00886588	23.266436
rs1175550	A	G	A	G	-0.198	0.001342143	0.5	0.4924	0.7468	0.004155242	0.032	5.00E-10	0.01450588	38.2851563
rs12153606	T	G	T	G	-0.159	-0.00150562	0.5	0.9564	0.8821	0.0101731	0.034	2.00E-06	0.00833796	21.8693772
rs12582659	C	T	C	T	1.262	-0.00088233	0.5	0.1102	0.8938	0.006634059	0.27	3.00E-06	0.00832949	21.8469684
rs2769264	G	T	G	T	0.313	-0.00259272	0.5	0.3135	0.5624	0.004477924	0.034	3.00E-20	0.03155481	84.7482699
rs3857536	T	C	T	C	-0.129	-0.00372135	0.5	0.3295	0.3997	0.004419651	0.028	4.00E-06	0.00809456	21.2257653

Selenium

SNP	effect_allel	other_allel	effect_allel	other_allele.out	beta.exposure	beta.outcome	eaf.exposure	eaf.outcome	pval.outcome	se.outcome	se.exposure	pval.exposure	R2	Fstat
rs7700970	T	C	T	C	0.265	-0.00990673	0.5	0.5511	0.01767	0.004176532	0.035	7.17E-13	0.0103621	57.3265306

Carotene

SNP	effect_allel	other_allel	effect_allel	other_allele.out	beta.exposure	beta.outcome	eaf.exposure	eaf.outcome	pval.outcome	se.outcome	se.exposure	pval.exposure	R2	Fstat
rs10846742	A	G	A	G	-0.143552	-0.00242316	0.832123	0.4861	0.5601	0.004156364	0.0208154	5.33E-12	6.85E-05	47.5607805
rs9708919	T	C	T	C	0.246529	-0.0015749	0.480938	0.6605	0.7194	0.004386921	0.0153225	3.03E-58	0.00037252	258.867046

Folate

SNP	effect_allel	other_allel	effect_allel	other_allele.out	beta.exposure	beta.outcome	eaf.exposure	eaf.outcome	pval.outcome	se.outcome	se.exposure	pval.exposure	R2	Fstat
rs1801133	G	A	G	A	0.096	-0.00796244	0.668	0.1112	0.2283	0.006607836	0.01077	1.00E-28	0.00364707	79.4531389
rs652197	C	T	C	T	0.069	-0.001996	0.179	0.4793	0.6315	0.004158325	0.01109	2.50E-10	0.00178025	38.7110623

Table S8 : Harmonization results for exposure SNPs and Sakaue et al. T1D outcome

Outcome: Sakaue, S (T1D)

Vitamin C

SNP	effect_allel	other_allel	effect_allel	other_allel	beta.expos	beta.outco	eaf.exposu	eaf.outcom	pval.outco	se.outcome	se.exposure	pval.expos	R2	Fstat
rs676317	T	C	T	C	-0.0366	0.225275	0.7264	0.994892	0.480778	0.319515	0.0074	7.33E-07	0.00047	24.46238
rs6693447	T	G	T	G	0.0393	0.054956	0.5509	0.757848	0.253538	0.048131	0.0064	6.25E-10	0.000724	37.70728
rs6482188	A	C	A	C	-0.0347	0.071072	0.3086	0.005822	0.809104	0.294196	0.0068	2.83E-07	0.0005	26.04001
rs10995578	C	G	C	G	0.0296	0.050962	0.4784	0.369966	0.234578	0.042874	0.0062	1.91E-06	0.000438	22.79292
rs542903	T	C	T	C	-0.0418	0.022681	0.1429	0.177535	0.672794	0.053706	0.0088	2.27E-06	0.000434	22.5625
rs174547	T	C	T	C	-0.0364	0.073875	0.6721	0.606732	0.080614	0.042283	0.0066	3.84E-08	0.000584	30.4169
rs2559850	A	G	A	G	0.0583	-0.04287	0.5979	0.347985	0.330666	0.044067	0.0064	6.30E-20	0.001593	82.98071
rs11062357	T	C	T	C	0.0305	0.006989	0.5118	0.802701	0.892283	0.051608	0.0063	1.46E-06	0.00045	23.43789
rs10136000	A	G	A	G	0.0404	0.105709	0.2825	0.157145	0.103512	0.064929	0.0071	1.33E-08	0.000622	32.3777
rs339969	A	C	A	C	-0.0302	-0.00533	0.6068	0.906176	0.939805	0.070555	0.0063	1.86E-06	0.000442	22.97909
rs56738967	C	G	C	G	0.041	0.073347	0.321	0.273205	0.117276	0.046828	0.0067	7.62E-10	0.000719	37.44709
rs9915323	A	T	A	T	0.0321	-0.05853	0.2967	0.452029	0.16154	0.041812	0.0068	2.60E-06	0.000428	22.28395
rs9895661	T	C	T	C	0.0625	-0.04102	0.817	0.46241	0.32182	0.041407	0.0081	1.05E-14	0.001143	59.53742
rs2366388	A	G	A	G	0.0309	-0.03603	0.4908	0.413908	0.394657	0.04233	0.0063	8.50E-07	0.000462	24.05669
rs12610033	A	T	A	T	-0.0336	0.015699	0.2831	0.496261	0.741743	0.047639	0.0073	4.86E-06	0.000407	21.18521
rs79234109	A	G	A	G	0.0453	-0.20813	0.1617	0.003733	0.540351	0.339932	0.0084	7.70E-08	0.000559	29.08291
rs73850547	A	G	A	G	0.0333	0.061776	0.7381	0.293435	0.183735	0.046471	0.0071	2.85E-06	0.000423	21.99742
rs7640441	A	C	A	C	0.0358	-0.06084	0.2383	0.221321	0.217217	0.049307	0.0074	1.19E-06	0.00045	23.40467
rs33972313	T	C	T	C	-0.3601	0.732885	0.0319	0.004064	0.008878	0.280079	0.0179	4.61E-90	0.00772	404.7065
rs4867910	T	C	T	C	-0.0314	0.053126	0.657	0.112094	0.430865	0.067443	0.0068	3.45E-06	0.00041	21.32266
rs1344896862	T	C	T	C	-0.039	0.001696	0.6585	0.643702	0.968855	0.043427	0.0066	3.64E-09	0.000671	34.91736
rs1165189	A	C	A	C	-0.0378	0.033946	0.7469	0.903558	0.623132	0.069078	0.0071	1.18E-07	0.000545	28.34438
rs868822	T	G	T	G	-0.0329	-0.06993	0.2648	0.500007	0.091508	0.041438	0.0071	3.30E-06	0.000413	21.47213
rs2941484	T	C	T	C	0.0341	0.028491	0.4514	0.435317	0.494105	0.041666	0.0063	6.37E-08	0.000563	29.2973
rs10758628	A	C	A	C	0.0304	-0.11898	0.4567	0.162565	0.036809	0.056988	0.0063	1.28E-06	0.000447	23.28445

Vitamin D

SNP	effect_allel	other_allel	effect_allel	other_allel	beta.expos	beta.outco	eaf.exposu	eaf.outcom	pval.outco	se.outcome	se.exposure	pval.expos	R2	Fstat
rs10888491	A	T	G	A	0.0104	-0.03468	0.5	0.723091	0.451492	0.046058	0.0023	4.86E-06	0.0003	20.44612
rs11203339	T	C	T	C	-0.0105	-0.02101	0.5	0.15925	0.709291	0.056347	0.0023	4.46E-06	0.000304	20.84121
rs1809851	A	G	T	A	0.0096	-0.00532	0.5	0.478276	0.899986	0.042353	0.002	2.95E-06	0.000301	23.04
rs11195965	A	G	A	T	0.0228	-0.01033	0.5	0.233146	0.83664	0.050122	0.0045	4.12E-07	0.000339	25.67111
rs2597193	T	G	T	C	-0.0404	0.032791	0.5	0.221225	0.507416	0.049469	0.0084	1.35E-06	0.000398	23.13152
rs10741657	T	C	T	C	-0.0104	0.036486	0.5	0.361233	0.396259	0.04301	0.0021	8.56E-07	0.000314	24.52608
rs12785878	T	C	A	C	-0.0892	0.034138	0.5	0.312576	0.446811	0.044875	0.0023	0	0.018864	1504.091
rs10745742	A	G	A	G	0.012	-0.00426	0.5	0.492813	0.918594	0.041644	0.0022	8.62E-08	0.000429	29.75207
rs8018720	T	C	G	C	0.0263	0.017542	0.5	0.681682	0.692444	0.04435	0.0027	8.14E-23	0.001326	94.88203
rs17082722	A	T	C	T	0.0118	-0.43215	0.5	0.016111	0.012394	0.17281	0.0025	2.21E-06	0.000354	22.2784
rs904856	A	T	G	A	0.01	0.016539	0.5	0.127638	0.787987	0.061499	0.0022	4.69E-06	0.000298	20.66116
rs17216707	A	G	A	G	0.0099	0.08393	0.5	0.916381	0.277233	0.077244	0.0022	4.90E-06	0.000295	20.25
rs204286	T	C	T	A	-0.0094	0.030306	0.5	0.221111	0.540524	0.049519	0.002	4.04E-06	0.000302	22.09
rs4821976	A	T	A	G	-0.0157	-0.02653	0.5	0.912135	0.714588	0.072538	0.0032	6.31E-07	0.00034	24.07129
rs6780224	T	G	A	G	0.0363	-0.138	0.5	0.066015	0.09768	0.083324	0.0022	3.81E-62	0.003464	272.25
rs12507653	T	G	T	A	0.0181	0.000118	0.5	0.609678	0.997814	0.042933	0.0023	4.18E-15	0.00099	61.93006
rs3755967	T	C	T	C	0.0105	0.031298	0.5	0.27508	0.497525	0.046135	0.0022	1.25E-06	0.000346	22.77893
rs7675387	T	C	A	C	0.0165	-0.31034	0.5	0.005689	0.274901	0.284232	0.0022	1.88E-14	0.000813	56.25
rs793000	T	C	T	C	-0.0703	-0.078	0.5	0.658441	0.075397	0.043869	0.0149	2.22E-06	0.00064	22.26066
rs7781168	C	G	A	G	-0.0168	-0.03546	0.5	0.133672	0.55933	0.060741	0.0029	4.72E-09	0.000492	33.56005
rs6982502	A	G	A	G	0.0187	0.052463	0.5	0.562087	0.205777	0.041464	0.0022	6.26E-17	0.000973	72.25
rs914787	A	G	A	G	0.01	0.030549	0.5	0.200484	0.556245	0.051916	0.0021	2.74E-06	0.00034	22.67574

Retinol (Vitamin A)

SNP	effect_allel	other_allel	effect_allel	other_allel	beta.expos	beta.outco	eaf.exposu	eaf.outcom	pval.outco	se.outcome	se.exposure	pval.expos	R2	Fstat
rs1667229	T	A	C	T	0.101271	0.086158	0.520114	0.908865	0.236677	0.072809	0.015461	5.74E-11	6.18E-05	42.9065
rs1667229	C	T	C	T	0.099027	0.086158	0.526657	0.908865	0.236677	0.072809	0.015421	1.35E-10	5.94E-05	41.23592

Vitamin K (1st model)

SNP	effect_allel	other_allel	effect_allel	other_allel	beta.expos	beta.outco	eaf.exposu	eaf.outcom	pval.outco	se.outcome	se.exposure	pval.expos	R2	Fstat
rs964184	G	C	G	C	0.23	-0.07291	0.15	0.278511	0.115825	0.046361	0.042	5.91E-08	0.013845	29.98866
rs2108622	T	C	T	C	0.16	-0.03619	0.3	0.283765	0.433543	0.046211	0.032	8.78E-07	0.011569	25
rs2192574	C	T	C	T	0.28	0.229719	0.11	0.004233	0.498321	0.339252	0.058	1.82E-06	0.010793	23.30559
rs4852146	C	T	C	T	0.18	-0.04888	0.33	0.195138	0.354662	0.052812	0.037	2.08E-06	0.010959	23.66691

Vitamin K (2nd model)

SNP	effect_allel	other_allel	effect_allel	other_allel	beta.expos	beta.outco	eaf.exposu	eaf.outcom	pval.outco	se.outcome	se.exposure	pval.expos	R2	Fstat
rs964184	G	C	G	C	0.14	-0.07291	0.15	0.278511	0.115825	0.046361	0.042	5.91E-08	0.005175	11.11111
rs2108622	T	C	T	C	0.16	-0.03619	0.3	0.283765	0.433543	0.046211	0.031	2.90E-07	0.012318	26.63892
rs2192574	C	T	C	T	0.28	0.229719	0.11	0.004233	0.498321	0.339252	0.057	1.49E-06	0.011171	24.1305
rs4852146	C	T	C	T	0.19	-0.04888	0.33	0.195138	0.354662	0.052812	0.035	1.42E-07	0.013609	29.46939
rs6862071	T	A	T	A	-1.14	0.070353	0.01	0.146199	0.229568	0.058556	0.22	1.35E-07	0.012415	26.85124

Vitamin K (3rd model)

SNP	effect_allel	other_allel	effect_allel	other_allel	beta.expos	beta.outco	eaf.exposu	eaf.outcom	pval.outco	se.outcome	se.exposure	pval.expos	R2	Fstat
rs2108622	T	C	T	C	0.16	-0.03619	0.3	0.283765	0.433543	0.046211	0.032	9.68E-07	0.011569	25
rs2192574	C	T	C	T	0.29	0.229719	0.11	0.004233	0.498321	0.339252	0.058	8.23E-07	0.011569	25
rs4852146	C	T	C	T	0.18	-0.04888	0.33	0.195138	0.354662	0.052812	0.038	3.23E-06	0.010395	22.43767
rs6862071	T	A	T	A	-1.09	0.070353	0.01	0.146199	0.229568	0.058556	0.23	1.73E-06	0.010405	22.45936

Vitamin B12

SNP	effect_allel	other_allel	effect_allel	other_allel	beta.expos	beta.outco	eaf.exposu	eaf.outcom	pval.outco	se.outcome	se.exposure	pval.expos	R2	Fstat
rs1801222	G	A	G	A	0.12	-0.0045	0.59	0.842575	0.937839	0.05774	0.007	3.30E-75	0.006407	293.8776
rs41281112	C	T	C	T	0.18	-0.0676	0.95	0.960698	0.531562	0.108046	0.015	8.90E-35	0.00315	144
rs3742801	T	C	T	C	0.05	0.106636	0.29	0.239549	0.027717	0.048443	0.007	1.70E-13	0.001118	51.02041
rs602662	A	G	A	G	0.17	-1.07148	0.6	5.41E-05	0.736185	3.180306	0.007	2.40E-139	0.012778	589.7959
rs2270655	G	C	G	C	0.1	-0.07131	0.94	0.906843	0.319156	0.07158	0.015	2.20E-13	0.000974	44.44444
rs1141321	C	T	C	T	0.07	-0.07942	0.63	0.777236	0.108901	0.04954	0.007	3.60E-26	0.00219	100

Vitamin B6

SNP	effect_allel	other_allel	effect_allel	other_allel	beta.expos	beta.outco	eaf.exposu	eaf.outcom	pval.outco	se.outcome	se.exposure	pval.expos	R2	Fstat
rs4654748	C	T	C	T	1.45	-0.01873	0.5	0.517398	0.648573	0.0411	0.281	8.00E-18	0.009	26.62707

Alpha-tocopherol

SNP	effect_allel	other_allel	effect_allel	other_allel	beta.expos	beta.outco	eaf.exposu	eaf.outcom	pval.outco	se.outcome	se.exposure	pval.expos	R2	Fstat
rs964184	G	C	G	C	0.04	-0.07291	0.15	0.278511	0.115825	0.046361	0.01	8.00E-12	0.004232	16

Gamma and beta-tocopherol

SNP	effect_allel	other_allel	effect_allel	other_allel	beta.expos	beta.outco	eaf.exposu	eaf.outcom	pval.outco	se.outcome	se.exposure	pval.expos	R2	Fstat
rs62508088	T	C	T	C	0.164877	0.102321	0.0992	0.064992	0.232933	0.08578	0.0179	1.00E-21	0.014676	84.84223

Magnesium

SNP	effect_allel	other_allel	effect_allel	other_allel	beta.expos	beta.outco	eaf.exposu	eaf.outcom	pval.outco	se.outcome	se.exposure	pval.expos	R2	Fstat
rs6667005	T	C	T	C	0.02947	0.066287	0.7844	0.904537	0.34972	0.070885	0.004962	2.86E-09	0.000242	35.27335
rs2818759	G	T	G	T	0.03013	-0.08265	0.344	0.225736	0.095094	0.049519	0.00441	8.39E-12	0.000321	46.67895
rs1890185	A	G	A	G	-0.02732	-0.02843	0.5875	0.544795	0.49253	0.041426	0.004063	1.75E-11	0.000311	45.21346
rs34872471	T	C	T	C	0.04058	-0.38108	0.7066	0.958629	0.000303	0.105474	0.004434	5.56E-20	0.000575	83.75924
rs773725592	T	G	T	G	-0.03358	0.02925	0.2763	0.204946	0.570851	0.051606	0.004637	4.43E-13	0.00036	52.44296
rs7894336	C	T	C	T	0.06273	-0.01239	0.4679	0.587039	0.767335	0.041868	0.004011	4.00E-55	0.001677	244.5937
rs7077696	A	G	A	G	0.02798	0.064777	0.7797	0.962807	0.554969	0.10973	0.00501	2.34E-08	0.000214	31.19033
rs7946549	T	A	T	A	0.04303	-0.00784	0.5441	0.851289	0.893042	0.058303	0.004036	1.52E-26	0.00078	113.6686
rs606970	A	G	A	G	-0.02401	-0.08684	0.5289	0.361895	0.043219	0.042957	0.00432	2.74E-08	0.000212	30.88992
rs3925584	T	C	T	C	0.07105	-0.05818	0.5475	0.683128	0.19109	0.044501	0.00402	6.73E-70	0.002141	312.3748
rs592859	C	G	C	G	0.03162	-0.01068	0.1578	0.363579	0.804822	0.043234	0.005773	4.31E-08	0.000206	29.99996
rs142601087	T	C	T	C	0.02922	-0.07537	0.806	0.835396	0.179733	0.056179	0.005168	1.57E-08	0.00022	31.968
rs2510467	A	G	A	G	0.03135	0.03339	0.4299	0.101332	0.629732	0.069259	0.004437	1.59E-12	0.000343	49.92249
rs11234579	C	T	C	T	-0.02714	0.015647	0.6373	0.981831	0.921822	0.159439	0.004453	1.10E-09	0.000255	37.14619
rs17832417	A	T	A	T	0.03899	-0.04045	0.6265	0.736844	0.389989	0.04706	0.004404	8.47E-19	0.000538	78.38119
rs1951970373	A	G	A	G	-0.04637	-0.05989	0.538	0.488419	0.153185	0.04193	0.004121	2.25E-29	0.000869	126.6103
rs11614506	T	C	T	C	0.03278	0.062482	0.7819	0.917634	0.4154	0.076719	0.004892	2.09E-11	0.000308	44.89986
rs35347302	G	A	G	A	-0.02671	0.016835	0.7702	0.829256	0.758108	0.054664	0.004774	2.20E-08	0.000215	31.30278
rs12230212	T	A	T	A	0.1637	-0.08942	0.989	0.973699	0.49734	0.131761	0.02743	2.39E-09	0.000245	35.61605
rs636264	G	A	G	A	-0.03387	-0.00771	0.2082	0.498703	0.852093	0.041346	0.005236	9.93E-11	0.000287	41.8438
rs303968	T	C	T	C	-0.03532	-0.09881	0.6028	0.156981	0.082741	0.056952	0.004198	3.98E-17	0.000486	70.78749
rs3783297	T	C	T	C	0.02579	-0.02785	0.6433	0.621392	0.511376	0.042408	0.004293	1.89E-09	0.000248	36.0895
rs3848132	T	A	T	A	-0.04543	0.027977	0.723	0.619816	0.513643	0.042832	0.004775	1.82E-21	0.000621	90.51879
rs4077450	G	T	G	T	-0.03324	-0.02026	0.2184	0.40639	0.642432	0.043632	0.005478	1.31E-09	0.000253	36.81951
rs12918968	A	C	A	C	-0.03944	0.017121	0.5741	0.415449	0.683558	0.042002	0.004172	3.25E-21	0.000614	89.36865
rs9897596	T	C	T	C	-0.02319	-0.00462	0.4911	0.809943	0.930262	0.052741	0.004061	1.13E-08	0.000224	32.60885
rs1035283	G	A	G	A	-0.06605	0.07519	0.9368	0.915965	0.337719	0.07843	0.00826	1.27E-15	0.000439	63.9419
rs2060289317	C	T	C	T	-0.08407	0.009001	0.3015	0.630855	0.832744	0.042622	0.004691	8.01E-72	0.002202	321.182
rs1989078891	A	G	A	G	0.02973	0.001813	0.6835	0.717249	0.968537	0.045965	0.004521	4.82E-11	0.000297	43.2435
rs112510641	G	A	G	A	0.03035	-0.21082	0.6104	0.975875	0.118567	0.135072	0.00418	3.86E-13	0.000362	52.71872
rs17794420	G	A	G	A	0.02859	-0.00403	0.5174	0.72372	0.930257	0.046052	0.004373	6.19E-11	0.000294	42.74343
rs711819	T	C	T	C	-0.03593	-0.01868	0.2421	0.573434	0.654309	0.041709	0.004965	4.61E-13	0.00036	52.3692
rs12464156	T	C	T	C	-0.02627	-0.14577	0.5962	0.890312	0.025146	0.0651	0.004107	1.58E-10	0.000281	40.91389
rs3732215	G	C	G	C	-0.02364	-0.06438	0.4801	0.389496	0.129467	0.042458	0.003994	3.26E-09	0.000241	35.03312
rs58560619	T	C	T	C	0.02335	0.08715	0.4936	0.964667	0.444557	0.113994	0.004079	1.04E-08	0.000225	32.76924
rs11694498	T	A	T	A	-0.04061	0.00612	0.6086	0.59721	0.884812	0.042243	0.004166	1.88E-22	0.000652	95.02272
rs219782	A	G	A	G	-0.06371	0.274976	0.7483	0.99153	0.211268	0.219966	0.004632	4.75E-43	0.001298	189.1813
rs6003469	A	C	A	C	0.04485	4.61E-05	0.6924	0.990074	0.999824	0.208252	0.004437	5.11E-24	0.000701	102.1753
rs1273884	G	A	G	A	0.04993	-0.05517	0.5595	0.858354	0.348444	0.058836	0.004091	2.87E-34	0.001022	148.9581
rs7374260	T	C	T	C	0.02461	0.011026	0.5278	0.138094	0.855307	0.060463	0.003992	7.04E-10	0.000261	38.00512
rs7039	C	G	C	G	0.03185	-0.01464	0.479	0.366223	0.733588	0.043027	0.004072	5.26E-15	0.00042	61.17914
rs1229984	T	C	T	C	0.07801	-0.00398	0.03517	0.756115	0.936473	0.049955	0.01116	2.79E-12	0.000336	48.8621
rs1755002318	T	C	T	C	0.02519	0.034022	0.5678	0.103597	0.615349	0.067712	0.004601	4.36E-08	0.000206	29.97449
rs13143189	G	A	G	A	0.02938	-0.01674	0.5727	0.798169	0.776142	0.058862	0.004194	2.47E-12	0.000337	49.07347
rs13146355	G	A	G	A	-0.06525	0.076742	0.5558	0.784114	0.130071	0.050694	0.004024	3.91E-59	0.001803	262.933
rs10043693	A	G	A	G	0.03589	0.046375	0.6921	0.829105	0.397676	0.054831	0.004433	5.63E-16	0.00045	65.54679
rs1743069877	C	T	C	T	0.02378	-0.03265	0.5502	0.751327	0.502243	0.048655	0.004199	1.48E-08	0.00022	32.07244
rs2542713	A	C	A	C	0.02645	-0.01637	0.4504	0.436801	0.696257	0.041922	0.004186	2.64E-10	0.000274	39.92573
rs1258226001	A	T	A	T	0.05615	0.03149	0.8149	0.780094	0.542237	0.051671	0.005266	1.53E-26	0.00078	113.6941
rs1467017814	G	T	G	T	-0.05568	0.011663	0.1741	0.245076	0.809099	0.048278	0.005631	4.71E-23	0.000671	97.7749
rs2439722	T	C	T	C	-0.02569	0.014967	0.528	0.442624	0.724326	0.042437	0.00424	1.36E-09	0.000252	36.71102
rs623297	C	T	C	T	0.02897	-0.01992	0.7303	0.891006	0.764862	0.066601	0.004862	2.56E-09	0.000244	35.50317
rs13193692	G	T	G	T	-0.03149	0.072991	0.7824	0.828918	0.215991	0.058994	0.005055	4.69E-10	0.000267	38.80637
rs77759957	A	G	A	G	0.0746	0.192284	0.9728	0.969627	0.116684	0.122564	0.01319	1.54E-08	0.00022	31.98807
rs7742789	C	T	C	T	0.05486	0.032897	0.6933	0.651116	0.447391	0.043299	0.004413	1.76E-35	0.001061	154.541
rs10952168	G	A	G	A	0.02429	0.02073	0.4401	0.296283	0.664205	0.047753	0.004443	4.57E-08	0.000205	29.88838
rs869287551	A	T	A	T	-0.04194	0.103957	0.8064	0.949163	0.272025	0.094643	0.00508	1.52E-16	0.000468	68.15998
rs66778952	T	G	T	G	-0.02741	0.036757	0.6696	0.871471	0.55172	0.061757	0.004336	2.57E-10	0.000274	39.96129
rs7850067	A	G	A	G	0.04308	0.136529	0.485	0.858685	0.031599	0.063518	0.004081	4.79E-26	0.000765	111.4341
rs898287122	G	C	G	C	0.02687	0.03293	0.6607	0.580812	0.450459	0.043637	0.004233	2.19E-10	0.000277	40.29385
rs1252526575	C	T	C	T	-0.04157	0.106869	0.6695	0.942634	0.221135	0.087346	0.004504	2.69E-20	0.000585	85.18503

rs3824347	A	G	A	G	0.05695	0.036264	0.5864	0.577976	0.391237	0.042297	0.004257	8.35E-41	0.001228	178.97

Potassium

SNP	effect_allel	other_allel	effect_allel	other_alle	beta.expos	beta.outco	eaf.exposu	eaf.outcom	pval.outco	se.outcom	se.exposur	pval.expos	R2	Fstat
rs880315	T	C	T	C	0.04161	0.048105	0.6739	0.316941	0.277886	0.044333	0.002572	7.72E-59	0.000433	261.73
rs3790604	C	A	C	A	0.04697	0.077286	0.9322	0.704604	0.090446	0.045649	0.004789	1.12E-22	0.000159	96.19477
rs78132593	C	A	C	A	0.01948	-0.18703	0.8381	0.95559	0.064843	0.101297	0.003462	1.87E-08	5.24E-05	31.66094
rs11264363	G	C	G	C	0.0252	0.018848	0.4285	0.239995	0.707162	0.050171	0.002659	2.78E-21	0.000149	89.81821
rs143341259	T	C	T	C	0.04981	-0.23002	0.9743	0.994001	0.432119	0.2928	0.008639	8.33E-09	5.56E-05	33.24345
rs199680901	C	T	C	T	-0.01837	0.028598	0.6385	0.30637	0.531829	0.045741	0.002631	3.01E-12	8.07E-05	48.75022
rs1609829	T	C	T	C	-0.01753	-0.09226	0.293	0.493212	0.03005	0.042529	0.002702	9.06E-11	6.97E-05	42.09138
rs4846476	G	C	G	C	0.01665	-0.0123	0.7784	0.736888	0.792856	0.046854	0.002851	5.38E-09	5.65E-05	34.10626
rs6697367	C	T	C	T	-0.01868	-0.01158	0.6768	0.670563	0.792618	0.044061	0.002575	4.19E-13	8.72E-05	52.62587
rs17367435	T	C	T	C	-0.01507	0.159637	0.5555	0.930566	0.050633	0.081673	0.002667	1.63E-08	5.29E-05	31.92864
rs3176466	C	T	C	T	0.04031	0.062059	0.8668	0.906425	0.383003	0.071138	0.003543	5.89E-30	0.000214	129.4444
rs6703881	T	C	T	C	-0.02296	-0.11949	0.1968	0.010946	0.558941	0.204458	0.00312	1.91E-13	8.97E-05	54.1545
rs145391254	C	T	C	T	0.03637	0.332969	0.9666	0.999035	0.655799	0.747033	0.006427	1.57E-08	5.36E-05	32.02359
rs68012176	T	C	T	C	-0.03616	1.205266	0.1179	0.002385	0.007031	0.44716	0.003902	2.01E-20	0.000142	85.87808
rs11245343	T	C	T	C	-0.02401	-0.03835	0.7283	0.706047	0.426393	0.04821	0.002872	6.53E-17	0.000116	69.89007
rs1426071185	A	G	A	G	-0.02184	-0.0074	0.3802	0.601	0.860572	0.042143	0.002547	1.05E-17	0.000122	73.52709
rs12766550	C	G	C	G	0.02881	0.041562	0.9417	0.882844	0.515925	0.063977	0.005162	2.44E-08	5.16E-05	31.14946
rs4745804	T	C	T	C	-0.01661	0.048046	0.5313	0.661676	0.271296	0.043675	0.002577	1.20E-10	6.88E-05	41.5442
rs2497318	C	T	C	T	-0.02938	-0.15312	0.6293	0.912265	0.03362	0.072068	0.002633	6.77E-29	0.000206	124.5093
rs1502282	G	C	G	C	-0.01458	-0.00937	0.5896	0.618142	0.82473	0.042323	0.0025	5.62E-09	5.63E-05	34.01222
rs12803281	C	T	C	T	-0.01661	-0.00524	0.5864	0.851091	0.928336	0.058262	0.002465	1.66E-11	7.52E-05	45.40518
rs1468168529	C	T	C	T	0.02448	0.057373	0.815	0.437216	0.168575	0.041671	0.00308	2.01E-15	0.000105	63.17153
rs4936409	A	G	A	G	-0.01693	-0.01333	0.491	0.463709	0.755471	0.042805	0.002558	3.75E-11	7.25E-05	43.80391
rs1268195353	T	G	T	G	0.03941	0.014065	0.6382	0.240508	0.773747	0.048924	0.002628	8.92E-51	0.000372	224.8859
rs963837	T	C	T	C	0.0215	-0.04253	0.6211	0.64843	0.335221	0.044131	0.002687	1.29E-15	0.000106	64.02382
rs1942088677	C	T	C	T	-0.02551	0.038925	0.9136	0.947258	0.671781	0.091868	0.004305	3.21E-09	5.82E-05	35.11354
rs4766578	T	A	T	A	0.01805	-1.05428	0.3993	5.32E-05	0.738846	3.162397	0.002668	1.39E-11	7.58E-05	45.77019
rs35444	A	G	A	G	-0.0336	-0.0398	0.6131	0.74469	0.396329	0.046923	0.00241	3.97E-44	0.000322	194.3768
rs28430881	C	A	C	A	-0.02156	-0.03656	0.5611	0.484485	0.376978	0.041383	0.002429	7.34E-19	0.00013	78.78481
rs66561220	T	C	T	C	-0.02949	-0.05136	0.7728	0.592293	0.223328	0.04218	0.003016	1.49E-22	0.000158	95.60638
rs11564194	C	T	C	T	-0.02113	0.073234	0.8063	0.981325	0.632566	0.153172	0.003081	7.27E-12	7.79E-05	47.0344
rs2255390	A	G	A	G	0.02748	-0.05107	0.4329	0.49525	0.217184	0.041385	0.002426	1.05E-29	0.000212	128.3074
rs1303347606	A	G	A	G	-0.02281	-0.04184	0.652	0.776581	0.400403	0.049761	0.002544	3.25E-19	0.000133	80.39265
rs9603367	C	T	C	T	0.05097	-0.08256	0.609	0.917558	0.289385	0.07793	0.002692	7.22E-80	0.000593	358.4917
rs716877	C	G	C	G	0.03596	0.096959	0.5162	0.852942	0.097059	0.058434	0.002627	1.35E-42	0.00031	187.3783
rs34917191	T	C	T	C	-0.01332	-0.0447	0.4203	0.196765	0.389628	0.051963	0.002409	3.32E-08	5.06E-05	30.57277
rs35812759	A	G	A	G	0.01552	0.083102	0.7522	0.827692	0.133442	0.055377	0.002755	1.79E-08	5.26E-05	31.73513
rs3848132	T	A	T	A	-0.03504	0.027977	0.7463	0.619816	0.513643	0.042832	0.002922	4.26E-33	0.000238	143.8029
rs690054	C	T	C	T	-0.03594	0.015932	0.5832	0.707832	0.723456	0.045026	0.002501	8.68E-47	0.000342	206.5041
rs57484993	A	G	A	G	0.03418	-0.02716	0.9219	0.886467	0.678349	0.065479	0.004607	1.22E-13	9.12E-05	55.04371
rs1433006247	A	C	A	C	0.02374	0.045049	0.637	0.934819	0.594475	0.08462	0.002662	4.99E-19	0.000132	79.53263
rs13333693	A	G	A	G	0.02753	0.032698	0.7554	0.633979	0.446552	0.042957	0.00284	3.46E-22	0.000156	93.96708
rs79392591	C	G	C	G	-0.0245	0.002576	0.8683	0.906157	0.970695	0.07012	0.003564	6.53E-12	7.83E-05	47.25598
rs7187932	G	A	G	A	-0.0223	0.058289	0.7746	0.736374	0.214507	0.046959	0.002875	9.15E-15	9.96E-05	60.16363
rs9928653	T	C	T	C	-0.01848	-0.10326	0.7631	0.86046	0.082569	0.05948	0.002844	8.46E-11	6.99E-05	42.22258
rs35619990	C	T	C	T	-0.01762	-0.1406	0.5513	0.077209	0.068898	0.077289	0.002433	4.59E-13	8.69E-05	52.44784
rs3751767	T	C	T	C	-0.01723	0.0301	0.6902	0.513299	0.466526	0.041338	0.002891	2.59E-09	5.88E-05	35.52012
rs164746	G	A	G	A	-0.02005	-0.16748	0.6178	0.982212	0.292703	0.159169	0.002597	1.22E-14	9.87E-05	59.6053
rs2168785	C	T	C	T	0.01863	-0.1429	0.3675	0.191925	0.006864	0.052861	0.002684	4.09E-12	7.98E-05	48.17932
rs8077544	G	A	G	A	0.01629	-0.02721	0.3428	0.2635	0.56325	0.047078	0.00285	1.13E-08	5.41E-05	32.67025
rs7218857	T	C	T	C	-0.02113	-0.03321	0.5618	0.108175	0.623427	0.067634	0.002471	1.30E-17	0.000121	73.12292
rs7222451	G	A	G	A	-0.01388	0.00996	0.4975	0.212094	0.846247	0.051365	0.002471	1.99E-08	5.23E-05	31.55248
rs2064929914	A	G	A	G	0.01947	-0.05231	0.8371	0.941411	0.550386	0.087595	0.003459	1.87E-08	5.25E-05	31.68333
rs10502917	T	C	T	C	-0.02136	-0.13871	0.3194	0.277841	0.003187	0.047034	0.002766	1.20E-14	9.88E-05	59.63458
rs12959198	T	C	T	C	-0.02103	-0.01494	0.7725	0.682815	0.740016	0.045015	0.002958	1.22E-12	8.37E-05	50.54547
rs1171399352	A	C	A	C	-0.02835	0.00075	0.5817	0.674398	0.986921	0.045726	0.002564	2.19E-28	0.000202	122.256
rs12975656	G	A	G	A	0.02031	0.005462	0.5826	0.783936	0.91387	0.0505	0.0027	5.53E-14	9.37E-05	56.58383
rs6716091	C	T	C	T	0.02352	0.094572	0.7446	0.945666	0.309498	0.093058	0.002796	4.19E-17	0.000117	70.76203
rs4848713	C	T	C	T	0.02442	0.049106	0.8902	0.481558	0.252976	0.042957	0.004187	5.65E-09	5.63E-05	34.01617
rs191079	T	C	T	C	0.01581	-0.01132	0.5793	0.391353	0.788858	0.042275	0.002839	2.62E-08	5.14E-05	31.01223
rs1427298	C	T	C	T	-0.01837	0.042086	0.6201	0.406943	0.317557	0.042107	0.002624	2.67E-12	8.12E-05	49.01067
rs1551287	C	T	C	T	0.02749	-0.05702	0.2156	0.004925	0.836369	0.276072	0.00299	4.04E-20	0.00014	84.52927
rs2195880	C	G	C	G	0.01569	-0.08757	0.7004	0.690336	0.049322	0.044549	0.002629	2.46E-09	5.90E-05	35.61761
rs62193645	C	T	C	T	0.03441	0.025532	0.8796	0.887453	0.695903	0.065324	0.003785	1.04E-19	0.000137	82.649
rs1275979	T	C	T	C	-0.03297	-0.03252	0.4719	0.775903	0.514848	0.049925	0.002568	1.09E-37	0.000273	164.8344
rs11124938	C	A	C	A	-0.02548	0.14353	0.7812	0.973968	0.278631	0.132481	0.002987	1.57E-17	0.000121	72.76598
rs6108787	T	G	T	G	0.01367	0.038396	0.5405	0.513267	0.352183	0.04127	0.002403	1.31E-08	5.36E-05	32.36156
rs1431175435	A	G	A	G	0.01961	-0.02908	0.5497	0.678403	0.517893	0.044979	0.002464	1.81E-15	0.000105	63.33938
rs4809849	C	T	C	T	0.01779	-0.0404	0.4172	0.363303	0.353266	0.043519	0.002675	3.03E-11	7.33E-05	44.22872
rs114048605	C	T	C	T	-0.1319	-0.57877	0.9894	0.99963	0.647532	1.265924	0.02131	6.28E-10	0.000218	38.31093
rs1989061	T	C	T	C	0.01826	-0.06103	0.3219	0.117074	0.347423	0.064958	0.002709	1.62E-11	7.52E-05	45.43427
rs1997596	C	T	C	T	-0.01956	-0.10184	0.666	0.750942	0.036504	0.048698	0.002733	8.62E-13	8.48E-05	51.22222
rs2823263	T	C	T	C	-0.02564	0.014656	0.7425	0.714898	0.749297	0.045861	0.002846	2.17E-19	0.000134	81.16452
rs219791	T	G	T	G	0.0173	0.12314	0.6668	0.954628	0.219479	0.100284	0.002688	1.26E-10	6.86E-05	41.42225
rs13072118	C	T	C	T	0.0161	-0.00671	0.4291	0.484606	0.871	0.041332	0.002608	6.93E-10	6.31E-05	38.10979
rs9822474	A	G	A	G	-0.01603	-0.06622	0.5519	0.272492	0.152582	0.046293	0.00239	2.06E-11	7.45E-05	44.98536
rs6794202	C	T	C	T	0.02384	1.071229	0.7896	0.999865	0.590702	1.991804	0.003025	3.39E-15	0.000103	62.11003
rs1273886	A	G	A	G	0.02013	-0.03891	0.6749	0.872512	0.529843	0.061939	0.002776	4.30E-13	8.71E-05	52.5834
rs780647553	A	G	A	G	0.01972	-0.08189	0.152	0.015578	0.63268	0.171337	0.003549	2.83E-08	5.11E-05	30.87463
rs13072590	G	A	G	A	0.01745	0.004622	0.786	0.4786	0.917635	0.044697	0.003135	2.69E-08	5.13E-05	30.98245

rs820429	G	T	G	T	-0.04348	-0.03024	0.6755	0.364303	0.516932	0.046657	0.002787	7.92E-55	0.000403	243.3913
rs1979845	C	T	C	T	0.03115	0.032822	0.8267	0.921384	0.66715	0.076319	0.003306	4.62E-21	0.000147	88.77903
rs9821489	G	A	G	A	0.04282	0.021006	0.8886	0.913398	0.777106	0.074204	0.004507	2.20E-21	0.000149	90.26476
rs4677143	A	G	A	G	0.01596	-0.0118	0.6379	0.439171	0.77683	0.041622	0.002609	9.70E-10	6.20E-05	37.42119
rs1452265382	G	T	G	T	-0.01667	-0.03674	0.4695	0.700431	0.422162	0.045766	0.00243	7.13E-12	7.79E-05	47.06073
rs10032927	A	T	A	T	0.01561	0.003132	0.5837	0.663912	0.943419	0.044132	0.002494	4.01E-10	6.49E-05	39.17535
rs6816915	G	A	G	A	-0.01441	-0.05459	0.4714	0.093784	0.44351	0.071237	0.002594	2.83E-08	5.11E-05	30.85944
rs12509595	T	C	T	C	0.03557	0.108244	0.7552	0.700839	0.01642	0.045112	0.002892	9.95E-35	0.00025	151.2764
rs4485922	A	G	A	G	-0.01442	-0.07514	0.46	0.416508	0.074169	0.042081	0.002442	3.56E-09	5.78E-05	34.86898
rs7726795	T	C	T	C	0.02854	0.04885	0.6314	0.733784	0.295779	0.046722	0.002657	7.04E-27	0.000191	115.3785
rs34070447	A	G	A	G	0.02013	-0.01132	0.5474	0.554553	0.787595	0.042016	0.00238	2.89E-17	0.000118	71.53748
rs11743019	A	G	A	G	-0.02052	0.035474	0.7361	0.830313	0.519645	0.055092	0.002981	6.04E-12	7.85E-05	47.3839
rs4867732	C	G	C	G	0.02908	-0.06072	0.9196	0.506265	0.141873	0.041339	0.004584	2.30E-10	6.67E-05	40.24386
rs6877631	T	C	T	C	-0.0154	0.054627	0.6149	0.829878	0.333897	0.056533	0.002594	2.99E-09	5.84E-05	35.24532
rs697976	T	C	T	C	0.01364	0.090339	0.4779	0.567388	0.03	0.041629	0.0024	1.34E-08	5.35E-05	32.30028
rs6941546	G	A	G	A	-0.02568	0.057228	0.9129	0.750629	0.233214	0.048005	0.004349	3.63E-09	5.77E-05	34.86673
rs761356	T	C	T	C	0.02838	0.051006	0.6163	0.540471	0.223225	0.041877	0.002531	3.76E-29	0.000208	125.7305
rs2327429	T	C	T	C	-0.02284	0.033099	0.737	0.482562	0.422422	0.041259	0.002788	2.65E-16	0.000111	67.113
rs2569882	T	C	T	C	-0.01474	-0.01598	0.5253	0.224243	0.753689	0.050933	0.002462	2.21E-09	5.94E-05	35.8442
rs2761244	T	C	T	C	0.01525	0.065619	0.3969	0.295954	0.153011	0.045921	0.002484	8.53E-10	6.24E-05	37.6909
rs210139	A	C	A	C	-0.01598	0.008765	0.5911	0.341196	0.839926	0.043392	0.0024	2.89E-11	7.34E-05	44.3334
rs1214759	A	G	A	G	-0.01681	-0.03396	0.4192	0.348491	0.432524	0.043266	0.002573	6.59E-11	7.07E-05	42.68309
rs55650455	T	C	T	C	-0.01947	-0.01805	0.8204	0.917219	0.811306	0.075584	0.003288	3.26E-09	5.81E-05	35.06455
rs111972532	T	C	T	C	0.03879	-0.12988	0.8139	0.987193	0.478696	0.183337	0.003086	3.39E-36	0.000262	157.9966
rs75741381	C	G	C	G	-0.01862	0.082357	0.7999	0.517489	0.048357	0.041716	0.003124	2.60E-09	5.88E-05	35.52526
rs62435145	G	T	G	T	-0.0256	0.02021	0.434	0.68576	0.65408	0.045101	0.0027	2.68E-21	0.000149	89.89849
rs71537958	G	C	G	C	-0.01936	0.01757	0.7389	0.583825	0.677594	0.042262	0.002891	2.22E-11	7.43E-05	44.84505
rs10265221	T	C	T	C	-0.03723	0.058285	0.7481	0.954308	0.556153	0.099029	0.002886	5.03E-38	0.000276	166.4154
rs61169316	G	A	G	A	0.01814	0.076729	0.7215	0.371055	0.072912	0.042785	0.002753	4.56E-11	7.19E-05	43.41723
rs1431882283	C	T	C	T	0.0605	0.017745	0.127	0.408615	0.67876	0.042845	0.003746	1.27E-58	0.000432	260.8406
rs6961048	C	G	C	G	0.04642	0.02231	0.8895	0.9539	0.827367	0.102304	0.003761	5.76E-35	0.000252	152.3364
rs576943137	C	T	C	T	-0.02548	-0.16374	0.8922	0.897704	0.019357	0.070018	0.004521	1.79E-08	5.26E-05	31.76361
rs3802177	G	A	G	A	0.01547	0.097738	0.7385	0.570221	0.018634	0.041542	0.00278	2.70E-08	5.13E-05	30.96642
rs7836572	T	C	T	C	0.02144	0.051803	0.4105	0.340212	0.234099	0.043536	0.002459	2.91E-18	0.000126	76.02081
rs12549572	T	C	T	C	0.02422	0.09492	0.6728	0.343247	0.029134	0.043508	0.002648	6.11E-20	0.000139	83.65893
rs28416181	T	G	T	G	0.02165	0.028053	0.71	0.625215	0.508955	0.042475	0.002647	3.04E-16	0.000111	66.8972
rs4737371	G	A	G	A	-0.02244	-0.06308	0.8207	0.856336	0.306997	0.061748	0.003276	7.74E-12	7.77E-05	46.91999
rs4461961	C	T	C	T	0.01818	0.020306	0.6471	0.630999	0.637194	0.043056	0.002611	3.43E-12	8.03E-05	48.48127
rs1854158822	T	C	T	C	-0.0279	-0.08119	0.5014	0.47372	0.049416	0.041319	0.002425	1.38E-30	0.000219	132.3686
rs869287551	A	T	A	T	-0.03231	0.103957	0.8437	0.949163	0.272025	0.094643	0.003411	2.96E-21	0.000149	89.72438
rs10811662	G	A	G	A	0.01906	0.214371	0.8401	0.55531	2.68E-07	0.041669	0.003305	8.20E-09	5.51E-05	33.25852
rs28558845	G	C	G	C	-0.02063	0.012196	0.82	0.736192	0.794983	0.046935	0.003066	1.76E-11	7.50E-05	45.27455

Iron

SNP	effect_allel	other_allel	effect_allel	other_allel	beta.expos	beta.outco	eaf.exposu	eaf.outcom	pval.outco	se.outcom	se.exposur	pval.expos	R2	Fstat
rs10831924	T	C	T	C	0.06204	0.024615	0.9131	0.961204	0.82383	0.110568	0.007982	8.06E-15	0.000474	60.41157
rs1958078	A	C	A	C	-0.03145	0.328644	0.2447	0.000999	0.626638	0.675578	0.005338	3.94E-09	0.000272	34.71236
rs12328766	A	G	A	G	0.03348	0.0068	0.6129	0.946712	0.939893	0.090182	0.004341	1.29E-14	0.000466	59.48279
rs13007705	C	T	C	T	-0.02771	0.023754	0.544	0.391798	0.582225	0.043179	0.004194	4.05E-11	0.000342	43.65321
rs855791	A	G	A	G	-0.1527	-0.06823	0.3768	0.565565	0.102001	0.041725	0.004498	2.09E-252	0.008959	1152.495
rs5995385	T	C	T	C	0.02734	-0.04465	0.5347	0.663134	0.304912	0.043525	0.004294	1.98E-10	0.000318	40.53899
rs2294915	C	T	C	T	-0.03195	-0.0459	0.7616	0.548878	0.267574	0.041405	0.005059	2.78E-10	0.000313	39.88525
rs8177252	C	A	C	A	-0.05473	0.050708	0.6848	0.534263	0.220833	0.041417	0.00463	3.25E-32	0.001095	139.7298
rs218248	G	A	G	A	-0.03636	0.016993	0.8382	0.723287	0.71329	0.046247	0.00575	2.64E-10	0.000314	39.98638
rs9402686	G	A	G	A	-0.05435	0.000565	0.7844	0.626077	0.989392	0.042484	0.005267	6.25E-25	0.000834	106.4811
rs72839066	A	G	A	G	-0.1163	1.192999	0.9694	0.999961	0.759445	3.896011	0.01327	1.99E-18	0.000607	76.80997
rs806970	C	T	C	T	0.05669	-0.15807	0.945	0.997344	0.737516	0.471645	0.009471	2.22E-09	0.000283	35.82789
rs7385804	C	A	C	A	-0.05785	-0.03288	0.357	0.151242	0.568585	0.057671	0.004417	3.75E-39	0.001344	171.5347

Zinc

SNP	effect_allel	other_allel	effect_allel	other_allel	beta.expos	beta.outco	eaf.exposu	eaf.outcom	pval.outco	se.outcom	se.exposur	pval.expos	R2	Fstat
rs7148590	A	G	A	G	-0.14	-0.09128	0.5	0.604018	0.03081	0.042267	0.026	1.40E-07	0.011024	28.99408
rs10484100	G	A	G	A	-0.209	-0.13103	0.5	0.059592	0.133599	0.087353	0.045	3.30E-06	0.008225	21.57086
rs2120019	C	T	C	T	-0.287	0.00613	0.5	0.504343	0.88202	0.041302	0.033	1.55E-18	0.028258	75.63728
rs4333127	A	G	A	G	0.218	-0.08726	0.5	0.809091	0.098436	0.052807	0.047	3.00E-06	0.008204	21.51381
rs11763353	G	A	G	A	-0.192	-0.82874	0.5	0.003624	0.030881	0.383924	0.039	7.00E-07	0.009232	24.23669
rs1532423	A	G	A	G	0.178	0.019447	0.5	0.608394	0.645257	0.042243	0.026	6.40E-12	0.017701	46.86982

Copper

SNP	effect_allel	other_allel	effect_allel	other_allel	beta.expos	beta.outco	eaf.exposu	eaf.outcom	pval.outco	se.outcom	se.exposur	pval.expos	R2	Fstat
rs2769264	G	T	G	T	0.313	-0.03343	0.5	0.309693	0.462064	0.045449	0.034	3.00E-20	0.031555	84.74827
rs1308125328	C	T	C	T	1.262	0.02487	0.5	0.166454	0.666102	0.057636	0.27	3.00E-06	0.008329	21.84697
rs10014072	G	A	G	A	-0.164	-0.00915	0.5	0.479171	0.824524	0.041247	0.034	1.00E-06	0.008866	23.26644
rs12153606	T	G	T	G	-0.159	0.129834	0.5	0.002545	0.785378	0.476773	0.034	2.00E-06	0.008338	21.86938
rs3857536	T	C	T	C	-0.129	0.004672	0.5	0.646792	0.913231	0.042875	0.028	4.00E-06	0.008095	21.22577

Selenium

SNP	effect_allel	other_allel	effect_allel	other_allel	beta.expos	beta.outco	eaf.exposu	eaf.outcom	pval.outco	se.outcom	se.exposur	pval.expos	R2	Fstat
rs1441893045	T	C	T	C	0.265	-0.02603	0.5	0.100492	0.719937	0.072593	0.035	7.17E-13	0.010362	57.32653

Carotene

SNP	effect_allel	other_allel	effect_allel	other_allel	beta.expos	beta.outco	eaf.exposu	eaf.outcom	pval.outco	se.outcom	se.exposur	pval.expos	R2	Fstat
rs10846742	A	G	A	G	-0.14355	0.006048	0.832123	0.322412	0.892832	0.044895	0.020815	5.33E-12	6.85E-05	47.56078
rs9708919	T	C	T	C	0.246529	0.069167	0.480938	0.138746	0.258149	0.061168	0.015323	3.03E-58	0.000373	258.867

Folate

SNP	effect_allel	other_allel	effect_allel	other_allel	beta.expos	beta.outco	eaf.exposu	eaf.outcom	pval.outco	se.outcome	se.exposure	pval.exposu	R2	Fstat
rs652197	C	T	C	T	0.069	0.015971	0.179	0.105129	0.823426	0.071572	0.01109	2.50E-10	0.00178	38.71106

Table S9 : Power analysis for the Two-Sample MR Analysis
https://shiny.cnsgenomics.com/mRnd/

Outcome: Chiou, J (T1D)

MR Power	K (cases/sample size)	R2	absolute detected effect (OR)	OR for power at 0.8
**Vitamin C**				
0.98	18 942/520 580	0.02993631	1.170960187	1.12
**Vitamin D**				
0.75	18 942/520 580	0.03507468	1.104972376	1.11
**Retinol (vitamin A)**				
0.06	18 942/520 580	1.75E-04	1.17370892	2.6
**Vitamin K (1st model)**				
0.83	18 942/520 580	0.06022538	1.088139282	1.085
**Vitamin K (2nd model)**				
0.66	18 942/520 580	0.05468707	1.071811361	1.085
**Vitamin K (3rd model)**				
0.31	18 942/520 580	0.04393804	1.051524711	1.1
**Vitamin B12**				
1	18 942/520 580	0.04662448	1.226	1.097
**Vitamin B6**				
0.05	18 942/520 580	0.009	1.01	1.231
**Alpha-tocopherol (vitamin E)**				
1.00	18 942/520 580	0.00423	3.846153846	1.325
**Gamma and beta-tocopherol (vitamin E)**				
0.65	18 942/520 580	0.02905156	1.103	1.125
**Magnesium**				
0.62	18 942/520 580	0.05065426	1.075268817	1.094
**Potassium**				
0.66	18 942/520 580	0.02952837	1.078748652	1.093
**Iron**				
0.16	18 942/520 580	0.02679285	1.043841336	1.128
**Zinc**				
0.94	18 942/520 580	0.08264445	1.090393632	1.073
**Copper**				
0.6	18 942/520 580	0.07968858	1.058201058	1.075
**Selenium**				
0.07	18 942/520 580	0.0103621	1.029	1.21
**Carotene**				
0.05	18 942/520 580	0.000440984	1.031991744	2.01
**Folate**				
0.97	18 942/520 580	0.005427325	1.396648045	1.29

Outcome: Verma, A (T1D)
Multi-ancestry

MR Power	K (cases/sample size)	R2	absolute detected effect (OR)	OR for power at 0.8
**Vitamin C**				
0.09	25 717/609 028	0.02928478	1.022	1.107
**Vitamin D**				
0.12	25 717/609 028	0.03547224	1.027	1.098
**Retinol (vitamin A)**				
0.06	25 717/609 028	1.16E-04	1.191895113	2.75
**Vitamin K (1st model)**				
0.16	25 717/609 028	0.06022538	1.026	1.075
**Vitamin K (2nd model)**				
0.06	25 717/609 028	0.05412862	1.009	1.08
**Vitamin K (3rd model)**				

0.06	25 717/609 028	0.04329234	1.007	1.08
**Vitamin B12**				
0.25	25 717/609 028	0.04662448	1.039	1.085
**Vitamin B6**				
0.05	25 717/609 028	0.008999806	1.003	1.195
**Alpha-tocopherol (vitamin E)**				
0.87	25 717/609 028	0.004231685	1.314	1.285
**Gamma and beta-tocopherol (vitamin E)**				
0.91	25 717/609 028	0.02905156	1.127395716	1.11
**Magnesium**				
1	25 717/609 028	0.05065426	1.184834123	1.083
**Potassium**				
1	25 717/609 028	0.0361415	1.495	1.098
**Iron**				
0.7	25 717/609 028	0.02764798	1.098	1.11
**Zinc**				
0.08	25 717/609 028	0.08264445	1.011	1.065
**Copper**				
0.05	25 717/609 028	0.07968858	1.002004008	1.065
**Selenium**				
0.06	25 717/609 028	0.0103621	1.015228426	1.18
**Carotene**				
0.05	25 717/609 028	0.000440984	1.033057851	1.9
**Folate**				
0.25	25 717/609 028	0.005427325	1.114827202	1.25

Outcome: Verma, A (T1D)

Hispanic or Latin American

MR Power	K (cases/sample size)	R2	absolute detected effect (OR)	OR for power at 0.8
**Vitamin C**				
0.05	2295/57429	0.02928478	1.012145749	1.35
**Vitamin D**				
0.12	2295/57429	0.03547224	1.088139282	1.32
**Retinol (vitamin A)**				
0.05	2295/57429	1.16E-04	1.178	5.43
**Vitamin K (1st model)**				
0.07	2295/57429	0.06022538	1.034	1.25
**Vitamin K (2nd model)**				
0.05	2295/57429	0.05412862	1.014	1.26
**Vitamin K (3rd model)**				
0.05	2295/57429	0.04329234	1.014	1.29
**Vitamin B12**				
0.05	2295/57429	0.04662448	1.022494888	1.28
**Vitamin B6**				
0.05	2295/57429	0.008999806	1.004016064	1.64
**Alpha-tocopherol (vitamin E)**				
0.06	2295/57429	0.00423	1.103752759	1.94
**Gamma and beta-tocopherol (vitamin E)**				
0.22	2295/57429	0.02905156	1.148105626	1.35
**Magnesium**				
0.05	2295/57429	0.05467637	1.006036217	1.26
**Potassium**				
0.33	2295/57429	0.0361415	1.171	1.32
**Iron**				
0.06	2295/57429	0.02764798	1.044	1.36
**Zinc**				
0.05	2295/57429	0.08264445	1.014198783	1.21
**Copper**				
0.05	2295/57429	0.07968858	1.004	1.21
**Selenium**				
0.05	2295/57429	0.0103621	1.002004008	1.595

**Carotene**				
0.05	2295/57429	0.000440984	1.0185	3.8
**Folate**				
0.05	2295/57429	0.005427325	1.0222	1.83

Outcome: Verma, A (T1D)

African American or Afro-Caribbean

MR Power	K (cases/sample size)	R2	absolute detected effect (OR)	OR for power at 0.8
**Vitamin C**				
0.05	6451/115861	0.02928478	1.007	1.21
**Vitamin D**				
0.05	6451/115861	0.03547224	1.004	1.19
**Retinol (vitamin A)**				
0.05	6451/115861	1.16E-04	1.149	4.15
**Vitamin K (1st model)**				
0.05	6451/115861	0.06022538	1.006036217	1.15
**Vitamin K (2nd model)**				
0.05	6451/115861	0.0672703	1.003009027	1.14
**Vitamin K (3rd model)**				
0.05	6451/115861	0.04329234	1.004016064	1.18
**Vitamin B12**				
0.08	6451/115861	0.04662448	1.028	1.17
**Vitamin B6**				
0.05	6451/115861	0.008999806	1.009081736	1.39
**Alpha-tocopherol (vitamin E)**				
0.05	6451/115861	0.004231685	1.027	1.56
**Gamma and beta-tocopherol (vitamin E)**				
0.26	6451/115861	0.02905156	1.1	1.22
**Magnesium**				
0.05	6451/115861	0.05036007	1	1.16
**Potassium**				
0.24	6451/115861	0.0361415	1.086	1.19
**Iron**				
0.05	6451/115861	0.02764798	1.003009027	1.22
**Zinc**				
0.05	6451/115861	0.08264445	1.003	1.125
**Copper**				
0.05	6451/115861	0.07968858	1.003009027	1.13
**Selenium**				
0.06	6451/115861	0.0103621	1.038421599	1.36
**Carotene**				
0.05	6451/115861	0.000440984	1	2.78
**Folate**				
0.06	6451/115861	0.005427325	1.05374078	1.5

Outcome: Saukae, S (T1D)

East asian

MR Power	K (cases/sample size)	R2	absolute detected effect (OR)	OR for power at 0.8
**Vitamin C**				
0.21	1219/133251	0.02161771	1.453488372	2.1
**Vitamin D**				
1	1219/133251	0.0322202	3.318	1.46
**Retinol (vitamin A)**				
0.08	1219/133251	1.21E-04	2.387	7.5
**Vitamin K (1st model)**				
0.64	1219/133251	0.04716566	1.308900524	1.38
**Vitamin K (2nd model)**				
0.1	1219/133251	0.05468707	1.084598698	1.36
**Vitamin K (3rd model)**				
0.09	1219/133251	0.04393804	1.077586207	1.39
**Vitamin B12**				
0.27	1219/133251	0.02661733	1.240694789	1.5
**Vitamin B6**				
0.05	1219/133251	0.008999806	1.013171226	1.85
**Alpha-tocopherol (vitamin E)**				

1.00	1219/133251	0.004231685	6.211180124	2.25
**Gamma and beta-tocopherol (vitamin E)**				
1	1219/133251	0.02905156	1.86	1.48
**Magnesium**				
0.06	1219/133251	0.03323819	1.050420168	1.45
**Potassium**				
1	1219/133251	0.01488229	2.214	1.66
**Iron**				
0.19	1219/133251	0.0156211	1.253	1.67
**Zinc**				
0.09	1219/133251	0.08264445	1.061	1.28
**Copper**				
0.05	1219/133251	0.0651827	1.008	1.32
**Selenium**				
0.07	1219/133251	0.0103621	1.103752759	1.8
**Carotene**				
0.05	1219/133251	0.000440984	1.169	4.8
**Folate**				
0.07	1219/133251	0.001780252	1.26	2.9

OR represents the absolute effect IVW ORs of the MR analyses

Table S10: SNP-Trait Associations in Potassium GWAS

SNP	n_assoc	Reported_traits
rs10086982	2	Telomere length (principal component 2); Telomere length (principal component 1)
rs10265221	46	Estimated glomerular filtration rate in non-diabetics; Hemoglobin; Calculus of kidney (PheCode 594.1); potassium (mean, inv-norm transformed); CHGB protein levels; Creatinine levels (UKB data field 23478); Serum urate levels; potassium (minimum, inv-norm transformed); Gout; Bicarbonate (maximum, inv-norm transformed); Creatinine levels; Estimated glon
rs10279895	2	Diastolic blood pressure; Hypertension
rs10502917	2	potassium (mean, inv-norm transformed); diastolic blood pressure (DBP, maximum, inv-normal transformed)
rs10748853	1	potassium (mean, inv-norm transformed)
rs1076485	14	Educational attainment (MTAG); potassium (mean, inv-norm transformed); Red blood cell count; Systolic blood pressure; Diastolic blood pressure; Triglyceride levels; Triacylglycerol (54:5) levels; Diastolic blood pressure (BMI adjusted); Mean arterial pressure (BMI adjusted); Systolic blood pressure (MTAG); Triacylglycerol (54:6) levels; Total cholesterol levels;
*rs10811662	17	Triglyceride levels; Type 2 diabetes; Random glucose levels; Diabetes / "Sugar"; Type 2 diabetes with neurological manifestations (PheCode 250.24); Sex hormone-binding globulin levels adjusted for BMI; Nonalcoholic fatty liver disease (imputed); Gestational diabetes mellitus; Polyneuropathy in diabetes (PheCode 250.6); Fasting blood glucose; Sex hormone-bin
rs11124938	2	potassium (mean, inv-norm transformed); potassium (minimum, inv-norm transformed)
rs111972532	3	potassium (mean, inv-norm transformed); potassium (minimum, inv-norm transformed); potassium (maximum, inv-norm transformed)
rs11217192	3	Fibrosis-4 index in high alcohol intake; Aspartate aminotransferase platelet ratio index in high alcohol intake; C-reactive protein levels
rs11245343	2	potassium (mean, inv-norm transformed); potassium (maximum, inv-norm transformed)
rs11264363	1	potassium (mean, inv-norm transformed)
rs115478735	246	Total lipids in IDL (UKB data field 23524); Triglycerides to total lipids in large LDL percentage (UKB data field 23618); neutrophil (absolute count, mean, inv-norm transformed); Disorders of lipid metabolism (PheCode 272); High cholesterol; Deep vein thrombosis [DVT] (PheCode 452.2); Free cholesterol levels in LDL; Angiotensin-converting enzyme levels; T
rs11564024	1	Immune reponse to smallpox (secreted IL-1beta)
rs11564194	1	potassium (mean, inv-norm transformed)
rs11743019	2	potassium (mean, inv-norm transformed); potassium (maximum, inv-norm transformed)
rs11822294	1	Serum urate levels
rs1214759	15	Neoplasm of uncertain behavior of skin (PheCode 173); Hypertension (PheCode 401); POMC protein levels; Total testosterone levels; Free testosterone levels; TAFA5 protein levels; Idiopathic pulmonary fibrosis; Bioavailable testosterone levels; Systolic blood pressure x alcohol consumption interaction (2df test); MSLN protein levels; Free androgen index; Essentia
rs12378991	4	potassium (mean, inv-norm transformed); Calcium levels; Kidney stone disease; Calcium levels (UKB data field 30680)
rs12509595	41	Glomerular filtration rate; Red blood cell count; Pulse pressure (BMI adjusted); potassium (mean, inv-norm transformed); Mean arterial pressure x alcohol consumption interaction (2df test); Pulse pressure; Chronic kidney disease; Takes medication for high blood pressure?; potassium (minimum, inv-norm transformed); Systolic blood pressure (BMI adjusted); Diast
rs12549572	1	potassium (mean, inv-norm transformed)
rs1273886	6	potassium (mean, inv-norm transformed); Systolic blood pressure (standard GWA); Systolic blood pressure (weighted GWA); Height; Peptic ulcer disease; Mean corpuscular hemoglobin concentration
rs1275979	4	potassium (mean, inv-norm transformed); Hypertension; Low density lipoprotein cholesterol levels; potassium (maximum, inv-norm transformed)
rs12803281	2	potassium (mean, inv-norm transformed); Red blood cell count
rs12940197	2	potassium (mean, inv-norm transformed); Estimated glomerular filtration rate
rs12959198	1	potassium (mean, inv-norm transformed)
rs12975656	3	potassium (mean, inv-norm transformed); potassium (minimum, inv-norm transformed); Severe COVID-19 infection
rs13042148	6	Sex hormone-binding globulin levels adjusted for BMI; Sex hormone-binding globulin levels; Diastolic blood pressure (MTAG); Diastolic blood pressure; triglyceride (mean, inv-norm transformed); Sex hormone-binding globulin levels in postmenopausal women
rs13072118	1	Medication use for hypertension (number of purchases)
rs13333693	2	potassium (mean, inv-norm transformed); potassium (maximum, inv-norm transformed)
rs1427298	8	Educational attainment (MTAG); potassium (mean, inv-norm transformed); potassium (minimum, inv-norm transformed); Educational attainment; CAMSIS occupational score (MTAG); ISEI occupational score (MTAG); Educational attainment (years of education); SIOPS occupational score (MTAG)
rs1502282	1	Systolic blood pressure (standard GWA)
rs150589717	8	systolic blood pressure (SBP, maximum, inv-normal transformed); diastolic blood pressure (DBP, mean, inv-normal transformed); systolic blood pressure (SBP, mean, inv-normal transformed); potassium (minimum, inv-norm transformed); Hypertension (PheCode 401); Essential hypertension (PheCode 401.1); potassium (mean, inv-norm transformed); diastolic bl
rs1529897	3	FEV1; FVC; Body mass index
rs1551287	2	potassium (mean, inv-norm transformed); potassium (maximum, inv-norm transformed)
rs1574817	1	potassium (mean, inv-norm transformed)
rs1609829	9	Alkaline phosphatase (UKB data field 30610); Body size (confirmatory factor analysis Factor 21); monocyte (absolute count, maximum, inv-norm transformed); Heel bone mineral density; Serum alkaline phosphatase levels; White blood cell count; platelet count (maximum, inv-norm transformed); Platelet count; monocyte (absolute count, mean, inv-norm transfor
rs164746	3	potassium (mean, inv-norm transformed); Plasma homocysteine levels; Creatinine levels (UKB data field 30700)
rs17038648	3	potassium (mean, inv-norm transformed); potassium (minimum, inv-norm transformed); Hypopotassemia (PheCode 276.14)
rs1757225	2	potassium (mean, inv-norm transformed); potassium (minimum, inv-norm transformed)
rs191079	1	Indolepropionate levels
rs1979845	1	potassium (mean, inv-norm transformed)
rs1984669	2	potassium (mean, inv-norm transformed); diastolic blood pressure (DBP, mean, inv-normal transformed)
rs1989061	1	potassium (mean, inv-norm transformed)
rs1997596	2	potassium (maximum, inv-norm transformed); height (mean, inv-normal transformed)
rs210139	2	potassium (mean, inv-norm transformed); Platelet count
rs2168785	2	potassium (mean, inv-norm transformed); Serum creatinine levels
rs2255390	3	potassium (mean, inv-norm transformed); potassium (minimum, inv-norm transformed); potassium (maximum, inv-norm transformed)
rs2327429	15	Coronary artery disease; Angina pectoris; Systolic blood pressure; Stable angina pectoris; Diastolic blood pressure; heart rate (HR, mean, inv-normal transformed); Bicarbonate (mean, inv-norm transformed); Systolic blood pressure (MTAG); Medication use (beta blocking agents); potassium (maximum, inv-norm transformed); Medication use (agents acting on the r
rs2340534	1	potassium (mean, inv-norm transformed)
rs2497318	12	Eosinophil counts; eosinophil (absolute count, maximum, inv-norm transformed); potassium (mean, inv-norm transformed); potassium (minimum, inv-norm transformed); Medication use (adrenergics, inhalants); Chronic inflammatory diseases (ankylosing spondylitis, Crohn's disease, psoriasis, primary sclerosing cholangitis, ulcerative colitis) (pleiotropy); eosinopł
rs2569882	5	Diastolic blood pressure (MTAG); Systolic blood pressure; Diastolic blood pressure; Cardiovascular disease; Pulse pressure
rs2823263	2	potassium (mean, inv-norm transformed); potassium (minimum, inv-norm transformed)
rs28430881	1	potassium (mean, inv-norm transformed)
rs28558845	5	potassium (mean, inv-norm transformed); Diastolic blood pressure; Medication use (calcium channel blockers); Systolic blood pressure; Pulse pressure
rs3176466	7	potassium (mean, inv-norm transformed); potassium (minimum, inv-norm transformed); Type 2 diabetes; Type 2 diabetes (PheCode 250.2); potassium (maximum, inv-norm transformed); Height; Hip circumference adjusted for BMI
rs34070447	9	potassium (mean, inv-norm transformed); potassium (minimum, inv-norm transformed); systolic blood pressure (SBP, mean, inv-normal transformed); systolic blood pressure (SBP, maximum, inv-normal transformed); Systolic blood pressure; High blood pressure / hypertension; Diastolic blood pressure; diastolic blood pressure (DBP, mean, inv-normal transforme
rs35444	26	Blood pressure; Systolic blood pressure; Mean arterial pressure; Diastolic blood pressure; Hypertension (PheCode 401); potassium (mean, inv-norm transformed); sodium (maximum, inv-norm transformed); potassium (minimum, inv-norm transformed); systolic blood pressure (SBP, mean, inv-normal transformed); systolic blood pressure (SBP, maximum, inv-norr
rs35619990	1	potassium (mean, inv-norm transformed)
rs3790604	17	Mean arterial pressure; Diastolic blood pressure; potassium (mean, inv-norm transformed); sodium (maximum, inv-norm transformed); potassium (minimum, inv-norm transformed); Heart failure; Systolic blood pressure; Hypertension; Pulse pressure; Medication use (calcium channel blockers); systolic blood pressure (SBP, mean, inv-normal transformed); Hyperte
rs3802177	17	Type 2 diabetes; Fasting plasma glucose; Hemoglobin A1c (HbA1c, minimum, inv-norm transformed); Diabetes mellitus (PheCode 250); Medication use (drugs used in diabetes); Fasting blood glucose; Fasting glucose; Type ii diabetes; Hemoglobin A1c levels; Hemoglobin A1c (HbA1c, maximum, inv-norm transformed); Type 2 diabetes (PheCode 250.2); Hemog
rs3848132	7	potassium (mean, inv-norm transformed); Urate levels; potassium (minimum, inv-norm transformed); magnesium (Mg, maximum, inv-norm transformed); magnesium (Mg, minimum, inv-norm transformed); Hip circumference adjusted for BMI; magnesium (Mg, mean, inv-norm transformed)
rs4461961	1	potassium (mean, inv-norm transformed)
rs4737371	2	potassium (mean, inv-norm transformed); potassium (minimum, inv-norm transformed)
rs4766578	91	Vitiligo; Cholesterol to total lipids ratio in medium LDL; Total lipid levels in small LDL; Total free cholesterol levels (UKB data field 23419); Medium fluorescent percentage of reticulocytes; SLAMF1 protein levels; Phospholipid levels in LDL; Free cholesterol to total lipids ratio in small HDL; Free cholesterol levels in LDL; Total cholesterol levels (UKB data field :
rs4809849	1	Medication use (diuretics)
rs4846476	2	Central corneal thickness; Free androgen index
rs4848713	1	Chloride levels
rs4867732	1	Body mass index
rs4936409	2	potassium (mean, inv-norm transformed); potassium (maximum, inv-norm transformed)
rs56218647	1	Gut microbiome abundance (class Clostridium sensu stricto sp. (at 3 months) x Vaginal Birth interaction
rs56376587	31	Estimated glomerular filtration rate in non-diabetics; Estimated glomerular filtration rate (creatinine, cystatin c); Estimated glomerular filtration rate (cystatin c); creatinine (mean, inv-norm transformed); potassium (mean, inv-norm transformed); Urea levels (UKB data field 30670); Lymphocyte count; estimated glomerular filtration rate (eGFR, minimum, inv-norm
rs569550	27	Diastolic blood pressure; Mean arterial pressure; Hypertension (PheCode 401); potassium (mean, inv-norm transformed); Takes medication for high blood pressure?; potassium (minimum, inv-norm transformed); Medication use (diuretics); Systolic blood pressure (BMI adjusted); Diastolic blood pressure (MTAG); diastolic blood pressure (DBP, maximum, inv-norn
rs57484993	1	potassium (mean, inv-norm transformed)
rs6031431	16	Mean arterial pressure; Pulse pressure; Diastolic blood pressure; potassium (mean, inv-norm transformed); potassium (minimum, inv-norm transformed); Systolic blood pressure (long-term average); Diastolic blood pressure (MTAG); systolic blood pressure (SBP, maximum, inv-normal transformed); Systolic blood pressure; Medication use (calcium channel blocker
rs60772526	16	Hypertension (PheCode 401); potassium (mean, inv-norm transformed); sodium (maximum, inv-norm transformed); potassium (minimum, inv-norm transformed); Mean arterial pressure; systolic blood pressure (SBP, mean, inv-normal transformed); systolic blood pressure (SBP, maximum, inv-normal transformed); Systolic blood pressure; diastolic blood pressure
rs6108787	16	Mean arterial pressure x alcohol consumption interaction (2df test); Systolic blood pressure; Medication use (diuretics); Systolic blood pressure (BMI adjusted); Diastolic blood pressure; Diastolic blood pressure (UKB data field 4079); Mean arterial pressure x alcohol consumption (light vs heavy) interaction (2df test); Systolic blood pressure (BMI unadjusted); Medi
rs61169316	2	potassium (mean, inv-norm transformed); Takes medication for high blood pressure?
rs61897793	90	Fatty acid(20:4)_[M-H]1- levels; Phosphatidylcholine_42:4_[M+OAc]1- levels; Phosphatidylcholine_38:3_[M+H]1+/Phosphatidylethanolamine_41:3_[M+H]1+/Phosphatidate_43:4_[M+NH4]1+ levels; Neutrophils and lymphocytes in blood (confirmatory factor analysis Factor 35); Phosphatidylcholine_44:5_[M+OAc]1- levels; Phosphatidylcholine-O_38:5_[M+H
rs62193645	2	potassium (mean, inv-norm transformed); potassium (minimum, inv-norm transformed)
rs62330329	1	Estimated glomerular filtration rate in non-diabetics
rs62374068	7	chloride (mean, inv-norm transformed); potassium (mean, inv-norm transformed); chloride (maximum, inv-norm transformed); potassium (minimum, inv-norm transformed); mean corpuscular hemoglobin concentration (MCHC, mean, inv-norm transformed); chloride (minimum, inv-norm transformed); potassium (maximum, inv-norm transformed)
rs62435145	45	Red blood cell count; estimated glomerular filtration rate (eGFR, minimum, inv-norm transformed); Red blood cell erythrocyte count (UKB data field 30010); Hemoglobin; Estimated glomerular filtration rate (creatinine, cystatin c); Estimated glomerular filtration rate (cystatin c); creatinine (mean, inv-norm transformed); potassium (mean, inv-norm transformed); r
rs66482211	1	potassium (mean, inv-norm transformed)
rs66561220	11	potassium (mean, inv-norm transformed); Lymphocyte count; lymphocyte (fraction, mean, inv-norm transformed); Height; systolic blood pressure (SBP, mean, inv-normal transformed); White blood cell count; Systolic blood pressure (BMI unadjusted); neutrophil (fraction, minimum, inv-norm transformed); potassium (maximum, inv-norm transformed); lymphoc
rs6697367	3	potassium (mean, inv-norm transformed); Putamen iron levels (R2* MRI); potassium (maximum, inv-norm transformed)
rs6716091	1	potassium (mean, inv-norm transformed)
rs6755070	4	potassium (mean, inv-norm transformed); Whole body fat free mass (UKB data field 23101); Hip circumference adjusted for BMI; Appendicular lean mass
rs6794202	4	potassium (mean, inv-norm transformed); Takes medication for Diabetes/sugar?; potassium (maximum, inv-norm transformed); Low myopia vs hyperopia
rs690054	3	potassium (mean, inv-norm transformed); potassium (minimum, inv-norm transformed); Retinal venular width
rs6961048	2	Mean arterial pressure x alcohol consumption interaction (2df test); Medication use (diuretics)
rs697976	2	Cysteine-rich protein 2 levels; HSPB6 protein levels
rs7038668	2	potassium (mean, inv-norm transformed); heart rate (HR, minimum, inv-normal transformed)
rs71537958	1	potassium (mean, inv-norm transformed)
rs716877	6	potassium (mean, inv-norm transformed); Urea levels (UKB data field 30670); potassium (minimum, inv-norm transformed); Glomerular filtration rate (creatinine); potassium (maximum, inv-norm transformed); Creatinine levels
rs7187932	1	potassium (mean, inv-norm transformed)
rs7218857	1	potassium (mean, inv-norm transformed)
rs75248620	5	potassium (mean, inv-norm transformed); Diastolic blood pressure; Estimated glomerular filtration rate (creatinine); Systolic blood pressure; Estimated glomerular filtration rate
rs75741381	4	Educational attainment; 25-hydroxyvitamin D levels (skin colour stratified); Serum 25-Hydroxyvitamin D levels; Serum 25-Hydroxyvitamin D levels (conditioned on BMI)
rs761356	3	potassium (mean, inv-norm transformed); potassium (minimum, inv-norm transformed); Pulse pressure
rs76987554	7	Blood pressure traits (multi-trait analysis); Systolic blood pressure; Diastolic blood pressure; Systolic blood pressure x smoking status (ever vs never) interaction (2df test); Systolic blood pressure x smoking status (current vs non-current) interaction (2df test); Pulse pressure; Systolic blood pressure x alcohol consumption interaction (2df test)
rs7726795	1	Hypopotassemia (PheCode 276.14)
rs78132593	9	Estimated glomerular filtration rate in non-diabetics; Hemoglobin A1c (HbA1c, minimum, inv-norm transformed); Caudate iron levels (quantitative susceptibility mapping); estimated glomerular filtration rate (eGFR, mean, inv-norm transformed); High light scatter reticulocyte count; Serum creatinine levels; Other non-epithelial cancer of skin (PheCode 172.2); cre
rs7836572	2	potassium (mean, inv-norm transformed); potassium (minimum, inv-norm transformed)
rs78716205	2	potassium (mean, inv-norm transformed); PSPN protein levels
rs7903146	176	Type 2 diabetes; Glycated hemoglobin levels; Metabolic syndrome; Fasting blood insulin (BMI interaction); Fasting blood glucose (BMI interaction); Proinsulin levels; Body mass index; Random glucose levels; Screening for other diseases and disorders (PheCode 1010.3); High cholesterol; sodium (minimum, inv-norm transformed); chloride (mean, inv-norm tran
rs79392591	1	potassium (mean, inv-norm transformed)
rs80176668	6	potassium (mean, inv-norm transformed); potassium (minimum, inv-norm transformed); systolic blood pressure (SBP, maximum, inv-normal transformed); systolic blood pressure (SBP, mean, inv-normal transformed); Hypopotassemia (PheCode 276.14); diastolic blood pressure (DBP, maximum, inv-normal transformed)
rs8125560	5	High light scatter reticulocyte percentage (UKB data field 30290); High light scatter reticulocyte count; Reticulocyte percentage (UKB data field 30240); Estimated glomerular filtration rate based on creatinine and cystatin C in bottom 99% of individuals by creatinine levels; High light scatter reticulocyte count (UKB data field 30300)
rs820429	3	potassium (mean, inv-norm transformed); diastolic blood pressure (DBP, mean, inv-normal transformed); potassium (maximum, inv-norm transformed)
rs880315	56	Blood pressure; Systolic blood pressure; Hypertension; Diastolic blood pressure; Mean arterial pressure; Takes medication for high blood pressure?; Triglyceride levels; Pulse pressure (BMI adjusted); Hypertension (PheCode 401); potassium (mean, inv-norm transformed); sodium (maximum, inv-norm transformed); Pulse pressure; Low-density lipoprotein levels (M
rs923000	2	potassium (mean, inv-norm transformed); potassium (minimum, inv-norm transformed)
rs9603367	3	potassium (mean, inv-norm transformed); diastolic blood pressure (DBP, mean, inv-normal transformed); potassium (maximum, inv-norm transformed)
rs963837	43	Renal function-related traits (sCR); Renal function-related traits (eGRFcrea); Glomerular filtration rate; Red blood cell count; Chronic kidney disease; Estimated glomerular filtration rate in non-diabetics; Hemoglobin; creatinine (mean, inv-norm transformed); potassium (mean, inv-norm transformed); Urea levels (UKB data field 30670); Urate levels; Urea levels; He
rs9821489	7	Hypertension (PheCode 401); potassium (mean, inv-norm transformed); potassium (minimum, inv-norm transformed); systolic blood pressure (SBP, maximum, inv-normal transformed); Diastolic blood pressure; Essential hypertension (PheCode 401.1); diastolic blood pressure (DBP, maximum, inv-normal transformed)
rs9822474	4	potassium (mean, inv-norm transformed); Blood urea nitrogen (BUN, mean, inv-norm transformed); Blood urea nitrogen (BUN, maximum, inv-norm transformed); Blood urea nitrogen (BUN, minimum, inv-norm transformed)
rs9844949	1	Total cholesterol levels

nerular filtration rate; AMBP protein levels; CKAP4 protein levels; Gout (PheCode 274.1); High blood pressure / hypertension; NPDC1 protein levels; Serum creatinine levels; Diastolic blood pressure; diastolic blood pressure (DBP, mean, inv-normal transformed); Hemoglobin concentration; Hematocrit; Systolic blood pressure; hematocrit (mean, inv-norm transformed); Anemia of chronic

Triacylglycerol (56:4) levels; Pulse pressure
ding globulin levels; Hemoglobin A1c levels; Glucose levels (UKB data field 30740); potassium (mean, inv-norm transformed); Glucose (finger stick, mean, inv-norm transformed); Type 1 diabetes; Biological Sibling: Diabetes / "Sugar"

'otal cholesterol levels (UKB data field 23400); Other chronic ischemic heart disease, unspecified (PheCode 411.8); prothrombin time (PT, mean, inv-norm transformed); Edema (PheCode 782.3); Ratio of apolipoprotein B to apolipoprotein A1 levels; Remnant cholesterol (non-HDL, non-LDL -cholesterol); Concentration of LDL particles (UKB data field 23429); Omega-6 fatty acids level

l hypertension (PheCode 401.1); Medication use for hypertension (number of purchases); Testosterone levels; Creatinine levels (UKB data field 30700)

:olic blood pressure; Bicarbonate (maximum, inv-norm transformed); chloride (minimum, inv-norm transformed); diastolic blood pressure (DBP, maximum, inv-normal transformed); Atrial fibrillation; Blood urea nitrogen levels; Ischemic stroke; systolic blood pressure (SBP, mean, inv-normal transformed); systolic blood pressure (SBP, maximum, inv-normal transformed); Heart failure;

ood pressure (DBP, maximum, inv-normal transformed)

med)

renin-angiotensin system); Essential hypertension (PheCode 401.1); Height; Pulse pressure; FEV1/FVC or gastro-oesophageal reflux disease (pleiotropy)

hil (absolute count, mean, inv-norm transformed); diastolic blood pressure (DBP, mean, inv-normal transformed); Microscopic hematuria (PheCode 593.2); Testosterone levels; Respiratory diseases; Mean platelet thrombocyte volume (UKB data field 30100)

d); diastolic blood pressure (DBP, maximum, inv-normal transformed)
nal transformed); Heart failure; Hypertension; High blood pressure / hypertension; platelet count (mean, inv-norm transformed); Systolic blood pressure (cigarette smoking interaction); diastolic blood pressure (DBP, mean, inv-normal transformed); Diastolic blood pressure (cigarette smoking interaction); Systolic blood pressure x alcohol consumption interaction (2df test); Sodium (mean,

nsion (confirmatory factor analysis Factor 12); Cardiovascular disease; Medication use (agents acting on the renin-angiotensin system); Primary aldosteronism; Sodium (mean, inv-norm transformed); Diastolic blood pressure (BMI unadjusted)
lobin A1c (HbA1c, mean, inv-norm transformed); Alanine levels; Body mass index; Type 2 diabetes (adjusted for BMI); Type 2 diabetes with ophthalmic manifestations (PheCode 250.23); Medication use for T2D (number of purchases)

23400); Current medication use (UKB data field 20003); Other chronic ischemic heart disease, unspecified (PheCode 411.8); Diseases of white blood cells (PheCode 288); potassium (mean, inv-norm transformed); Arthritis (juvenile idiopathic); Phospholipid levels in large LDL; High light scatter reticulocyte percentage of red cells; sodium (minimum, inv-norm transformed); Sarcoidosis; I

transformed); potassium (minimum, inv-norm transformed); MYL3 protein levels; Estimated glomerular filtration rate (creatinine); Serum urate levels; estimated glomerular filtration rate (eGFR, maximum, inv-norm transformed); Blood urea nitrogen (BUN, maximum, inv-norm transformed); estimated glomerular filtration rate (eGFR, mean, inv-norm transformed); Blood urea nitrogen (
nal transformed); systolic blood pressure (SBP, maximum, inv-normal transformed); Heart failure; Systolic blood pressure; Hypertension; Systolic blood pressure (BMI unadjusted); systolic blood pressure (SBP, mean, inv-normal transformed); Medication use (calcium channel blockers); Hypopotassemia (PheCode 276.14); Diastolic blood pressure (BMI adjusted); Mean arterial pressure (B

rs); Hypertension; systolic blood pressure (SBP, mean, inv-normal transformed); Cardiovascular disease; Medication use (agents acting on the renin-angiotensin system); Systolic blood pressure (MTAG); Medication use (diuretics)
(DBP, mean, inv-normal transformed); Hypopotassemia (PheCode 276.14); potassium (maximum, inv-norm transformed); Sodium (mean, inv-norm transformed); Diastolic blood pressure; diastolic blood pressure (DBP, maximum, inv-normal transformed); Essential hypertension (PheCode 401.1); Takes medication for high blood pressure?
cation use (beta blocking agents); Hypertension (confirmatory factor analysis Factor 12); Hypertension; Diastolic blood pressure x alcohol consumption interaction (2df test); Mean arterial pressure (BMI adjusted); Systolic blood pressure (MTAG); Systolic blood pressure x alcohol consumption interaction (2df test); Diastolic blood pressure x alcohol consumption (light vs heavy) interactic

I]1+/Phosphatidylcholine-P_38:4_[M+H]1+ levels; Phosphatidylcholine-O_40:5_[M+H]1+/Phosphatidylcholine-P_40:4_[M+H]1+ levels; Phosphatidylethanolamine-O_36:4_[M-H]1-/Phosphatidylethanolamine-P_36:3_[M-H]1- levels; Phosphatidylethanolamine-O_36:5_[M-H]1-/Phosphatidylethanolamine-P_36:4_[M-H]1- levels; Phosphatidylcholine-O_44:5_[M+H]1+/Phosphatidylcho

ed blood cell count (RBC, minimum, inv-norm transformed); REG3A protein levels; Creatinine levels (UKB data field 23478); red blood cell count (RBC, mean, inv-norm transformed); hematocrit (minimum, inv-norm transformed); Serum creatinine levels; Estimated glomerular filtration rate (creatinine); estimated glomerular filtration rate (eGFR, maximum, inv-norm transformed); Bloo

cyte (fraction, maximum, inv-norm transformed); neutrophil (fraction, mean, inv-norm transformed)

atinine (minimum, inv-norm transformed); Fasting glucose

sformed); Acquired absence of limb (PheCode 1089); Glucosuria (moderate to severe); Hemoglobin A1c (HbA1c, minimum, inv-norm transformed); Takes medication for Diabetes/sugar?; Type 2 diabetes with renal manifestations (PheCode 250.22); Hyperpotassemia (PheCode 276.13); Body mass index (BMI, minimum, inv-normal transformed); red blood cell count (RBC, minimum, in

ITAG); Ischemic stroke (small-vessel); Ischemic stroke; potassium (minimum, inv-norm transformed); sodium (minimum, inv-norm transformed); Resistance to antihypertensive treatment in hypertension; Systolic blood pressure (BMI adjusted); Hemoglobin A1c (HbA1c, mean, inv-norm transformed); lymphocyte (fraction, mean, inv-norm transformed); Atrial fibrillation; systolic blood p

moglobin concentration; Serum urate levels; chloride (maximum, inv-norm transformed); Serum creatinine levels; estimated glomerular filtration rate (eGFR, maximum, inv-norm transformed); magnesium (Mg, minimum, inv-norm transformed); Serum uric acid levels; Estimated glomerular filtration rate; Estimated glomerular filtration rate in diabetes; Blood urea nitrogen (BUN, mean,

c disease (PheCode 285.2); EFNA4 protein levels; LGALS7 or LGALS7B protein levels; TNFRSF11A protein levels; SCARB2 protein levels; potassium (maximum, inv-norm transformed); HDL cholesterol levels (UKB data field 30760); TNFRSF1A protein levels; Glomerular filtration rate; Red blood cell c

s (UKB data field 23445); Hemoglobin A1c (HbA1c, minimum, inv-norm transformed); Total lipid levels in small LDL; Coronary atherosclerosis (PheCode 411.4); prothrombin time (PT, maximum, inv-norm transformed); Phospholipid levels in LDL; Low-density lipoprotein receptor-related protein 8 l

Medication use (calcium channel blockers); Systolic blood pressure (BMI unadjusted); Estimated glomerular filtration rate; Hypertension; Medication use (antithrombotic agents); Systolic blood pressure; Potassium levels; Diastolic blood pressure x alcohol consumption interaction (2df

inv-norm transformed); Essential hypertension (PheCode 401.1); Mean spheric corpuscular volume; diastolic blood pressure (DBP, maximum, inv-normal transformed); platelet count (minimum, inv-norm transformed); PTPRH protein levels; Pulse pressure; Takes medication for high blood pressur

Linoleic acid levels; Gout; Peripheral vascular disease, unspecified (PheCode 443.9); Cholesterol levels in large LDL; Cholesteryl ester levels in large LDL; Total cholesterol minus HDL-C levels; Smoking initiation; Phospholipid levels in small LDL; Atherosclerosis of

(BUN, mean, inv-norm transformed); creatinine (maximum, inv-norm transformed); Serum creatinine levels; Blood urea nitrogen levels; Heel bone mineral density; Blood urea nitrogen (BUN, minimum, inv-norm transformed); creatinine (minimum, inv-norm transformed); MSTN protein levels;
BMI adjusted); Systolic blood pressure (MTAG); REN protein levels; Pulse pressure; Medication use (beta blocking agents); Essential hypertension (PheCode 401.1); Mean arterial pressure (BMI unadjusted); Pulse pressure (BMI unadjusted)

on (2df test)

line-P_44:4_[M+H]1+ levels; Phosphatidylcholine(37:4)_[M+H]1+/Phosphatidylethanolamine(40:3)_[M+H]1+ levels; Phosphatidylcholine(38:4)_[M+H]1+/Phosphatidylethanolamine(41:4)_[M+H]1+ levels; Sphingomyelin_36:2_[M+H]1+ levels; Lysophosphatidylethanolamine_22:4_[M-H]1- levels; Phosphatidylcholine(38:4)_[M+OAc]1-/Phosphatidylserine(42:3)_

d urea nitrogen (BUN, minimum, inv-norm transformed); hemoglobin (minimum, inv-norm transformed); Blood urea nitrogen (BUN, maximum, inv-norm transformed); Estimated glomerular filtration rate; estimated glomerular filtration rate (eGFR, mean, inv-norm transformed); hematocrit (maxi

iv-norm transformed); Gestational diabetes mellitus; magnesium (Mg, mean, inv-norm transformed); Insulin pump user (PheCode 250.3); potassium (minimum, inv-norm transformed); Pulse pressure; Secondary diabetes mellitus (PheCode 249); Weight (minimum, inv-normal transformed); Dermatophytosis (PheCode 110.

pressure (SBP, mean, inv-normal transformed); systolic blood pressure (SBP, maximum, inv-normal transformed); Medication use (diuretics); diastolic blood pressure (DBP, maximum, inv-normal transformed); High blood pressure / hypertension; Bicarbonate (mean, inv-norm transformed); Aldosterone

inv-norm transformed); Serum phosphate levels; Cystatin C levels; hemoglobin (maximum, inv-norm transformed); Blood urea nitrogen levels; Estimated glomerular filtration rate (creatinine); Hematocrit; Bicarbonate (mean, inv-norm transformed); Blood urea nitrogen (BUN, minimum, i
