# Supplementary material for: Association of Vitamins and Minerals with Type 1 Diabetes Risk: A Mendelian Randomization Study
Source: Nutrients. 2025 Oct 20;17(20):3297. doi: 10.3390/nu17203297 (PMC12566610; doi:10.3390/nu17203297)
Supplement: Supplementary file 1 [file nutrients-17-03297-s001.zip › Supplementary Figures.pdf]

Association of vitamins and micronutrients with type 1 diabetes risk: a Mendelian randomization study

Supplemental Figure S1: Scatter plot of MR analysis for micronutrients on the T1D outcomes

1. <u>Chiou, J. (European T1D):</u>

(A)

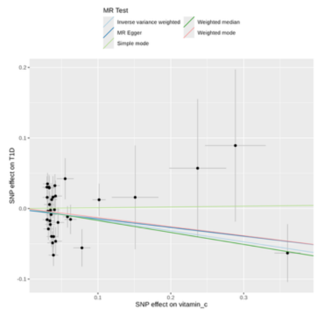

(B)

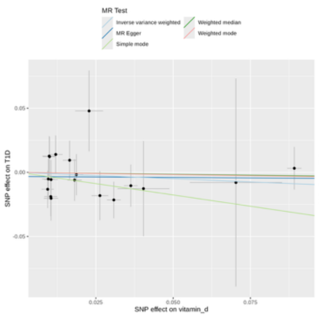

(C)

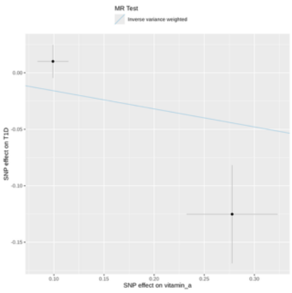

(D)

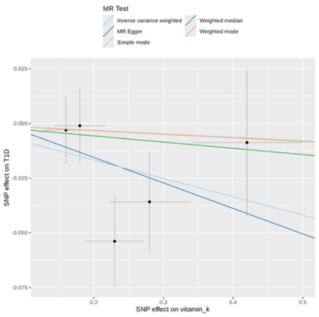

(E)

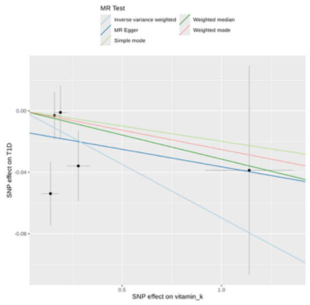

(F)

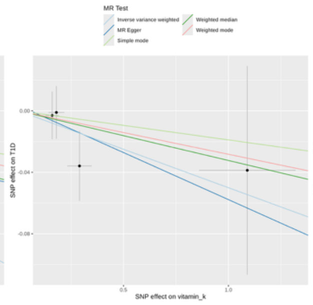

(G)

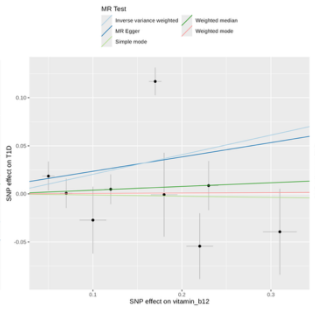

(H)

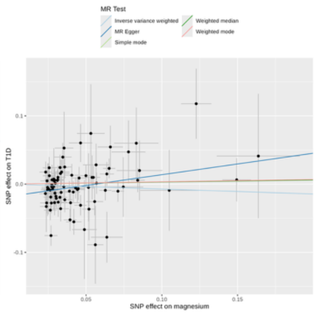

(I)

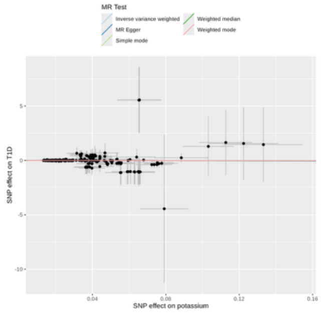

(J)

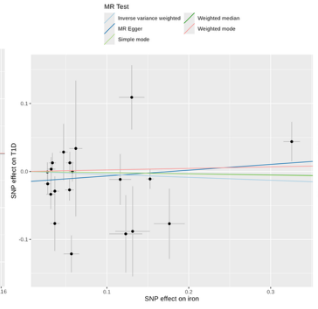

(K)

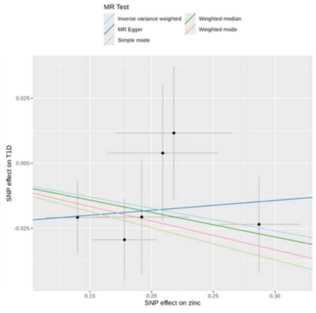

(L)

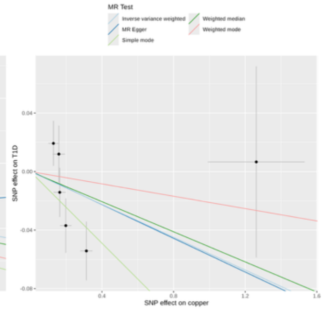

(M)

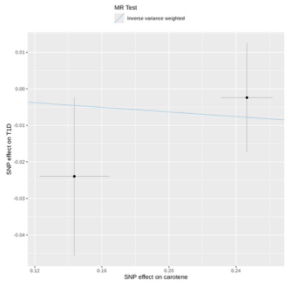

(N)

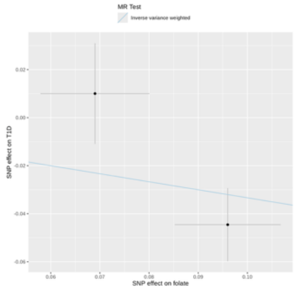

2. Verma, A. (Multi-ancestry T1D)

(A)

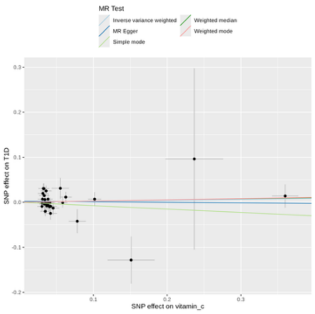

(B)

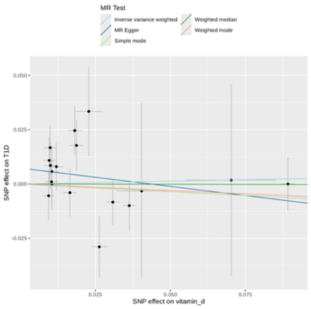

(C)

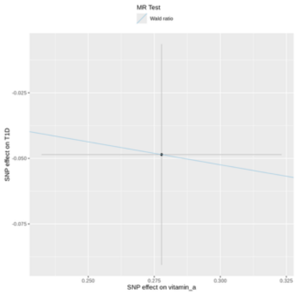

(D)

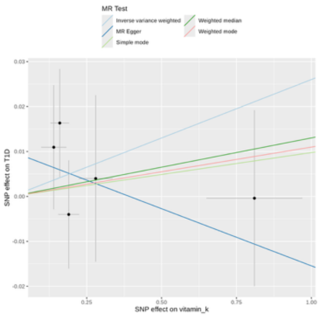

(E)

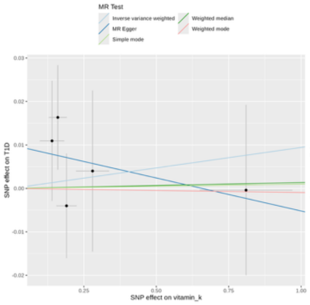

(F)

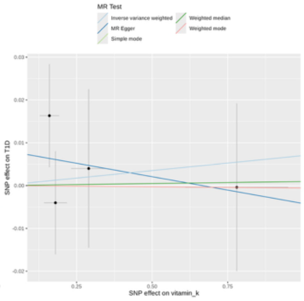

(G)

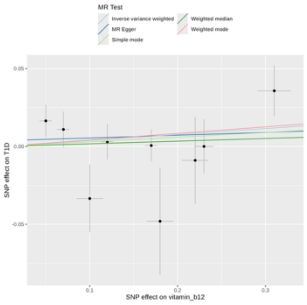

(H)

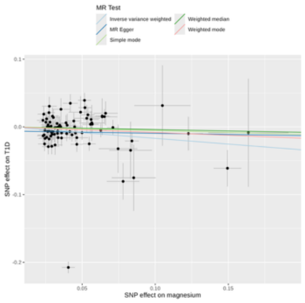

(I)

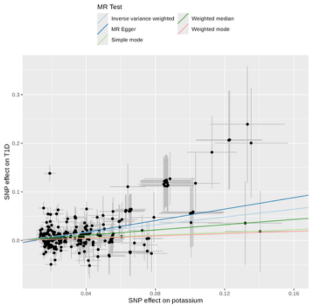

(J)

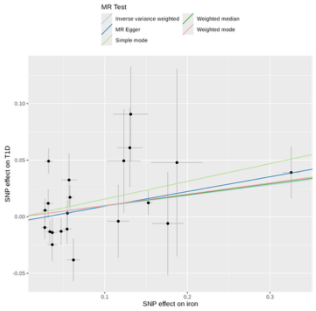

(K)

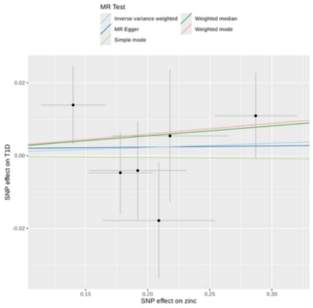

(L)

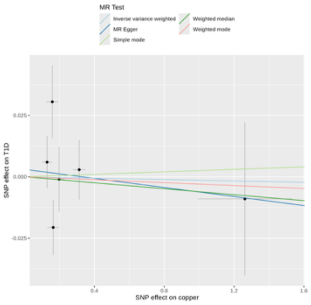

(M)

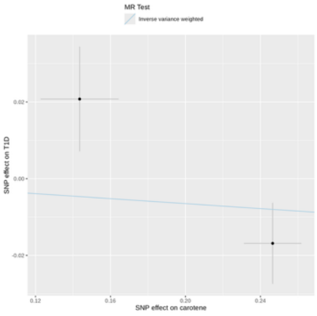

(N)

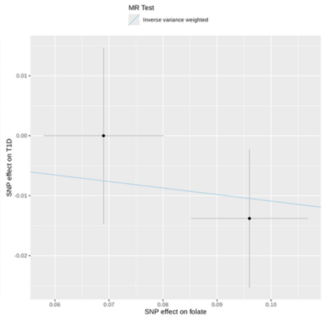

3. Verma, A. (Hispanic/Latin American T1D)

(A)

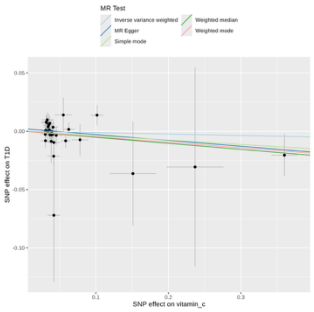

(B)

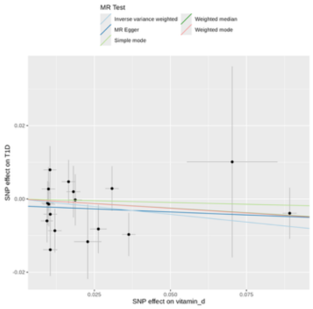

(C)

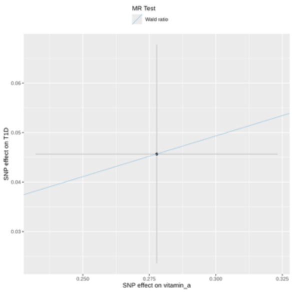

(D)

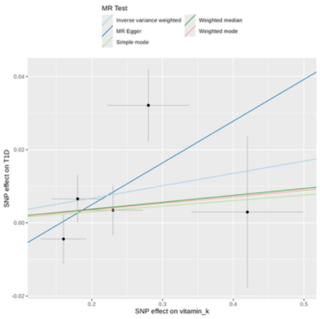

(E)

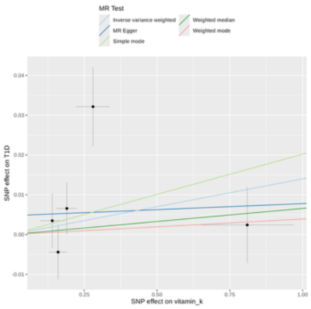

(F)

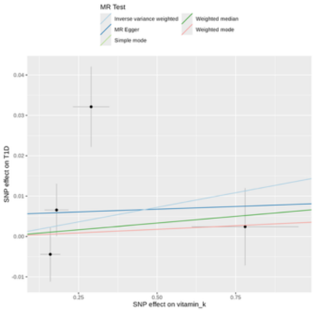

(G)

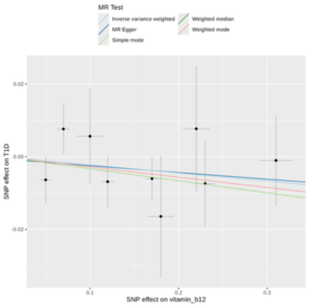

(H)

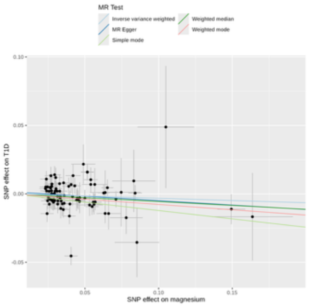

(I)

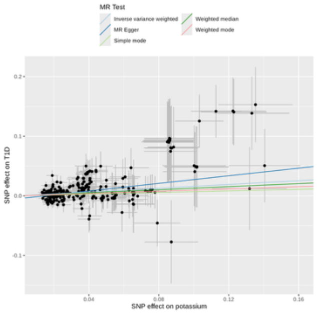

(J)

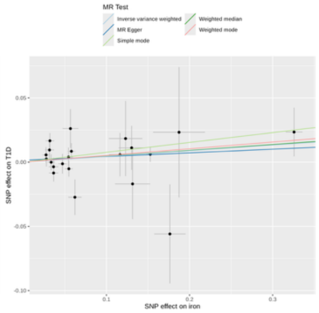

(K)

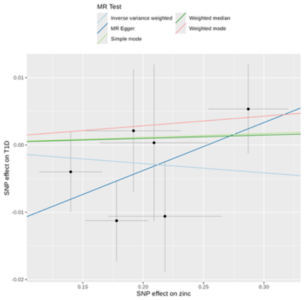

(L)

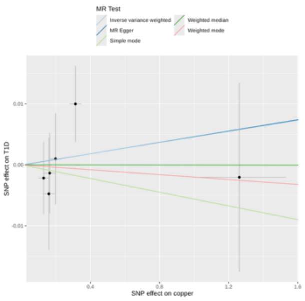

(M)

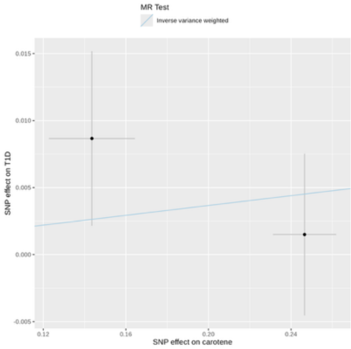

(N)

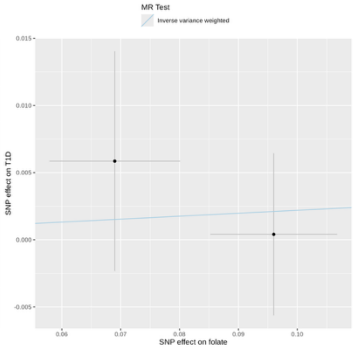

4. Verma, A. (African American/Afro-Carribean T1D)

(A)

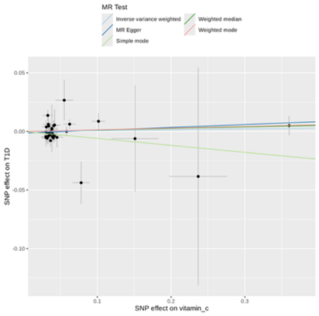

(B)

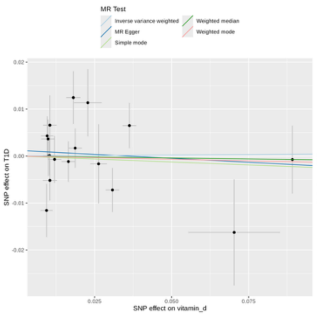

(C)

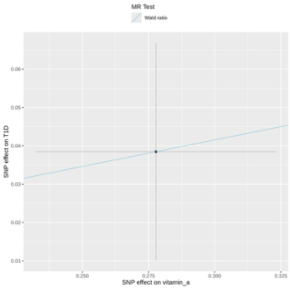

(D)

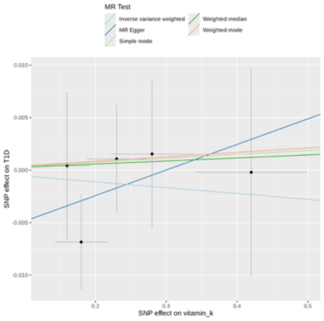

(E)

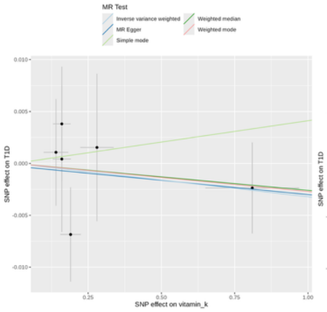

(F)

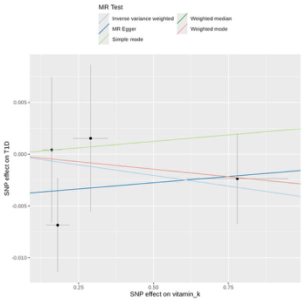

(G)

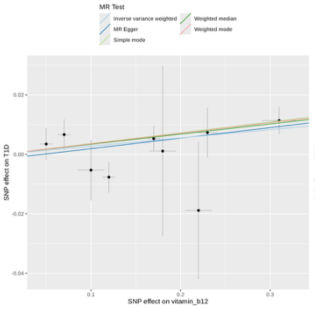

(H)

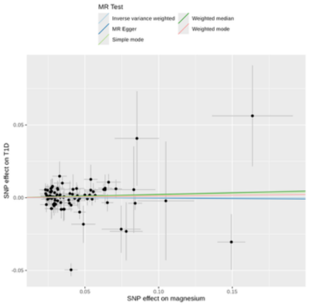

(I)

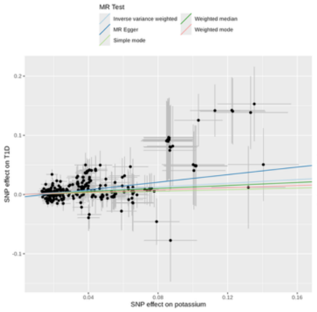

(J)

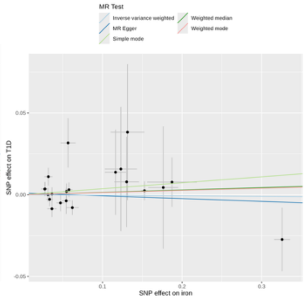

(K)

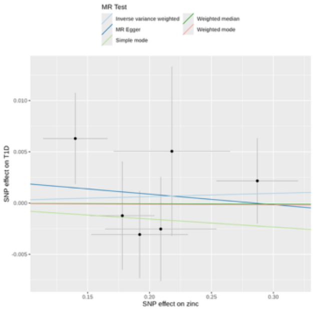

(L)

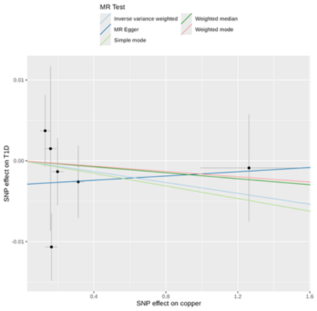

(M)

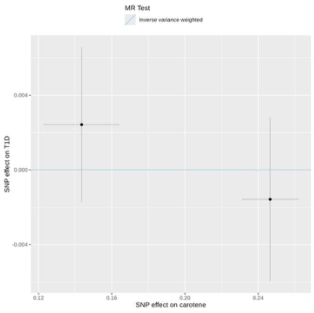

(A) Vitamin C; (B) Vitamin D; (C) Retinol; (D) Vitamin K 1st model; (E) Vitamin K 2nd model; (F) Vitamin K 3rd model; (G) Vitamin B12; (H) Magnesium; (I) Potassium; (J); Iron; (K) Zinc; (L) Copper; (M) Carotene; (N) Folate

Figures for vitamin B6, alpha-, beta-, and gamma-tocopherol, selenium, and folate (for African American/Afro-Carribean T1D GWAS) are not shown due to insufficient numbers of SNPs.

5. Sakaue, S. (East asian T1D)

(A) (B) (C) (D)

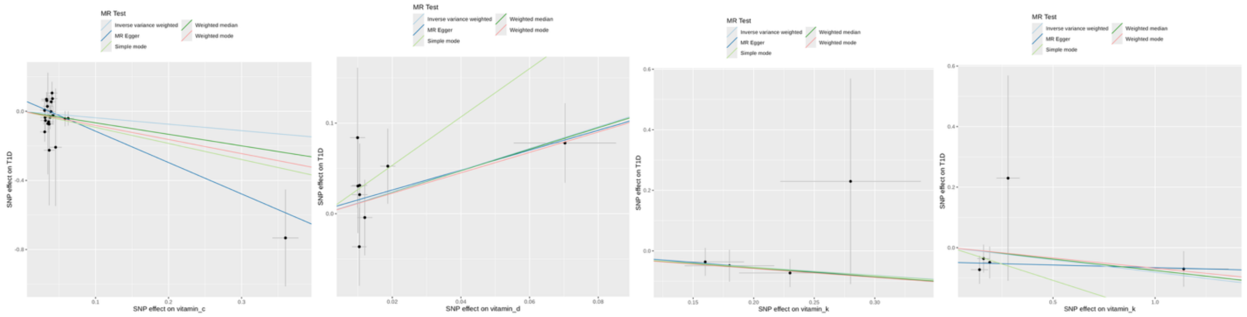

(E) (F) (G) (H)

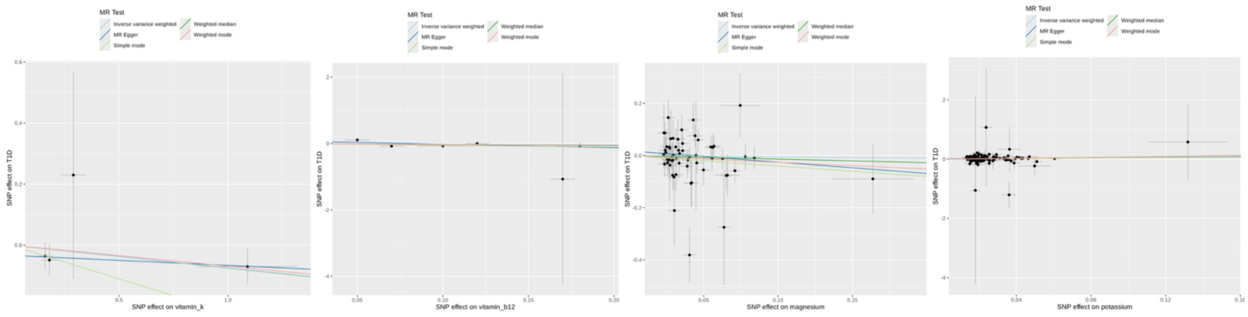

(I) (J) (K) (L)

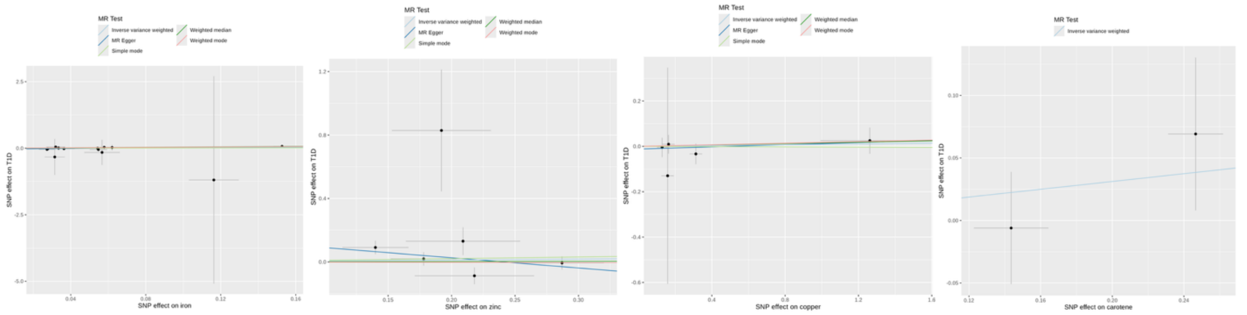

(A) Vitamin C; (B) Vitamin D; (C) Vitamin K 1st model; (D) Vitamin K 2nd model; (E) Vitamin K 3rd model; (F) Vitamin B12; (G) Magnesium; (H) Potassium; (I); Iron; (J) Zinc; (K) Copper; (L) Carotene

Figures for retinol, vitamin B6, alpha-, beta-, and gamma-tocopherol, selenium, folate are not shown due to insufficient numbers of SNPs

Supplemental Figure S2: Forest plots of MR analysis for micronutrients on the T1D outcomes

1. <u>Chiou, J. (European T1D)</u>

(A)

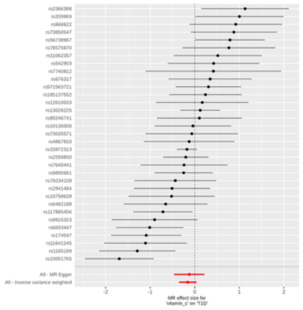

(B)

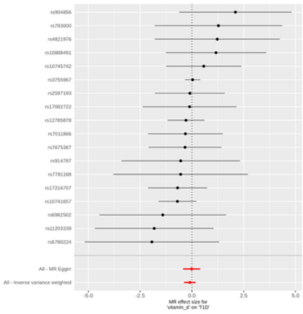

(C)

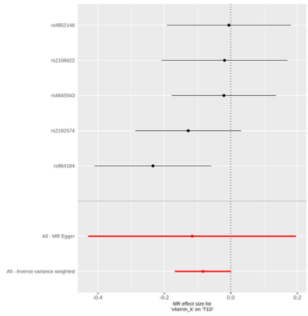

(D)

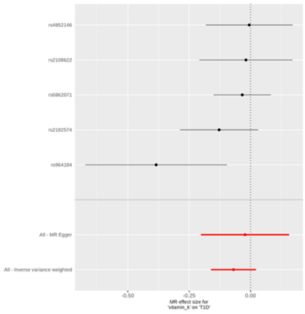

(E)

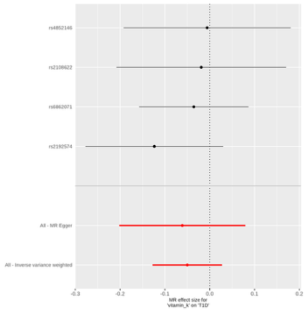

(F)

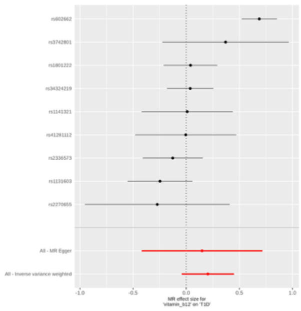

(G)

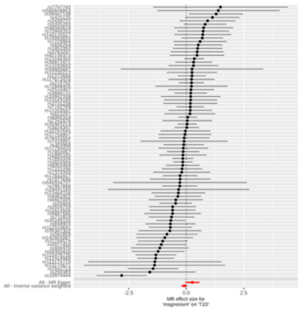

(H)

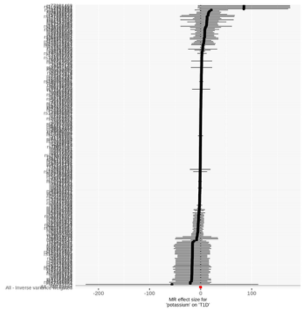

(I)

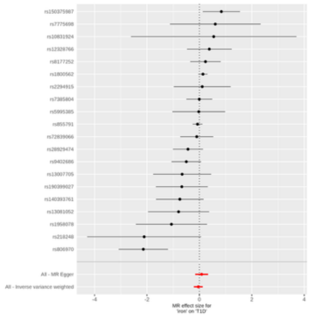

(J)

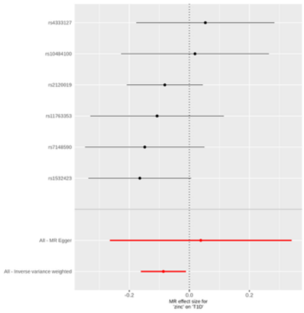

(K)

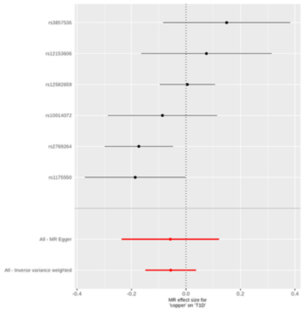

(L)

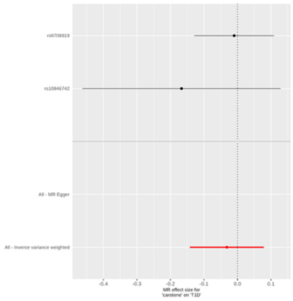

(M)

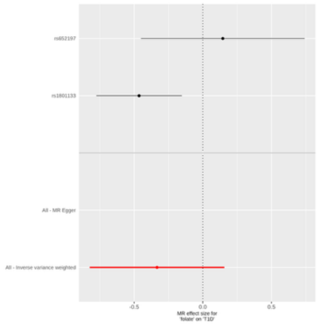

2. <u>Verma, A. (Multi-ancestry T1D)</u>

(A)

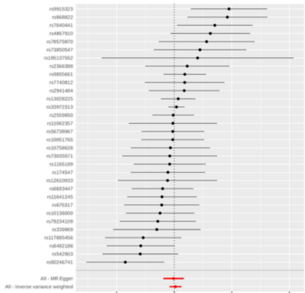

(B)

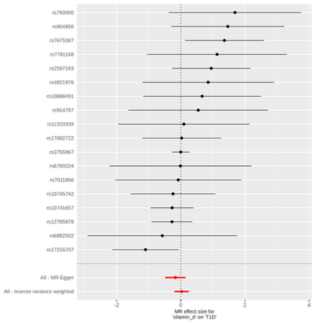

(C)

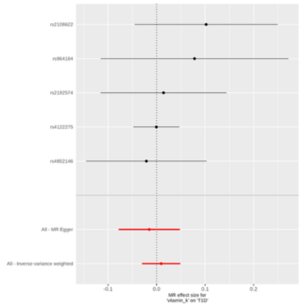

(D)

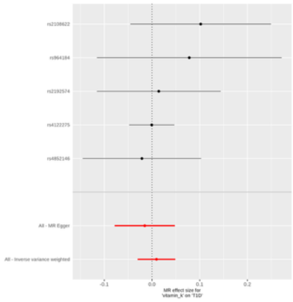

(E)

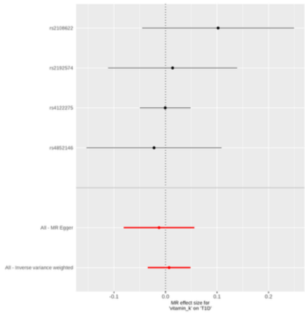

(F)

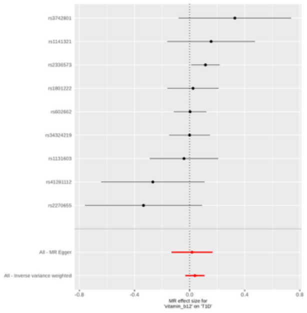

(G)

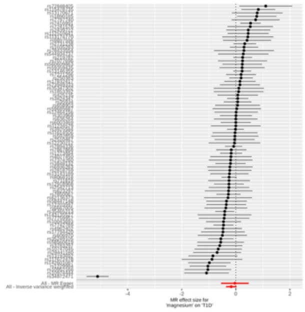

(H)

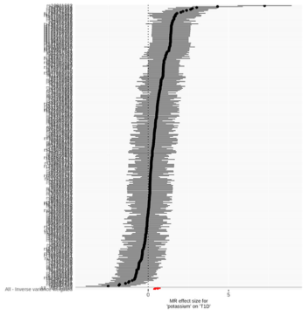

(I)

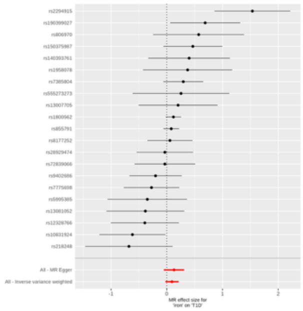

(J)

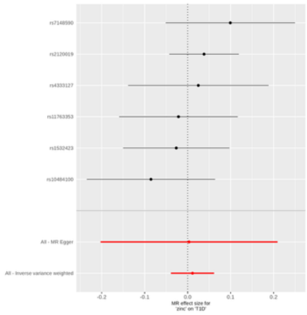

(K)

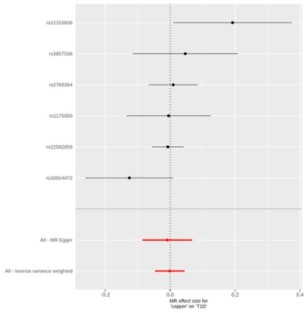

(L)

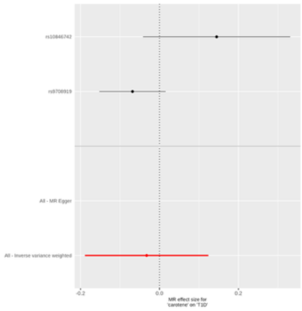

(M)

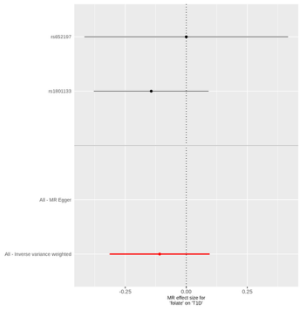

3. Verma, A. (Hispanic/Latin American T1D)

(A)

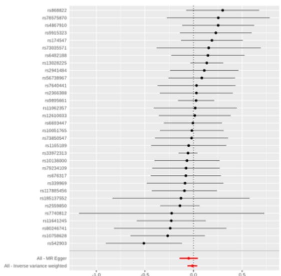

(B)

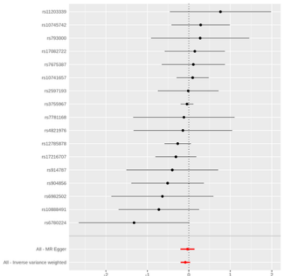

(C)

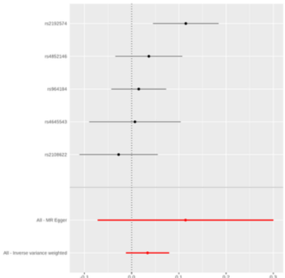

(D)

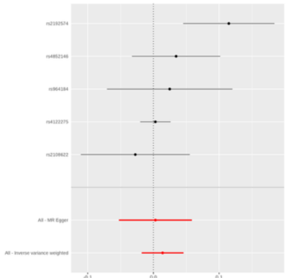

(E)

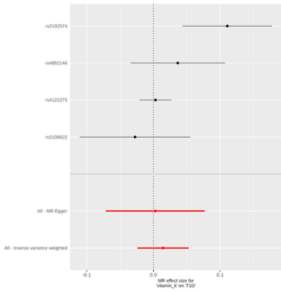

(F)

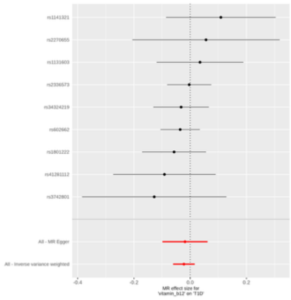

(G)

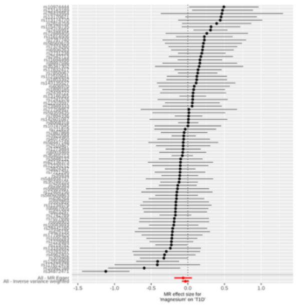

(H)

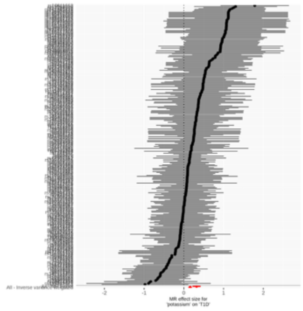

(I)

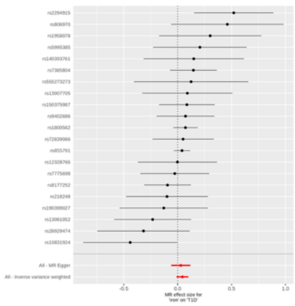

(J)

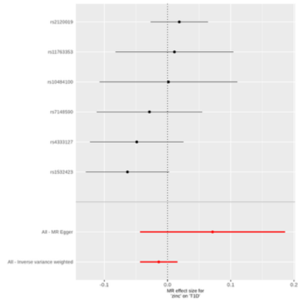

(K)

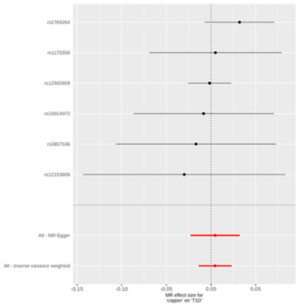

(L)

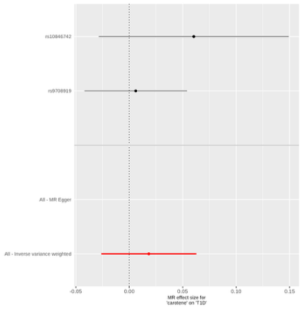

(M)

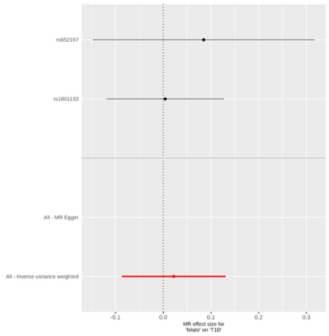

4. Verma, A. (African American/Afro-Carribean T1D)

(A)

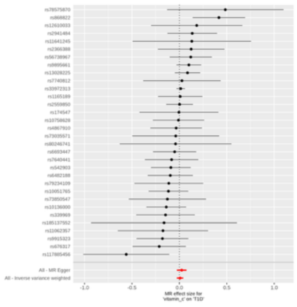

(B)

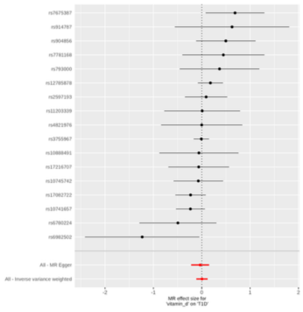

(C)

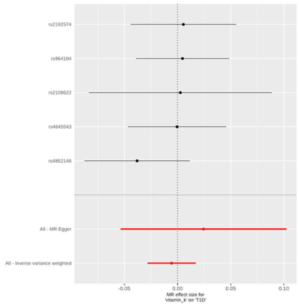

(D)

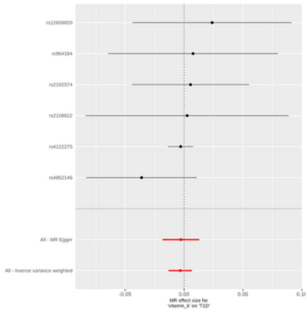

(E)

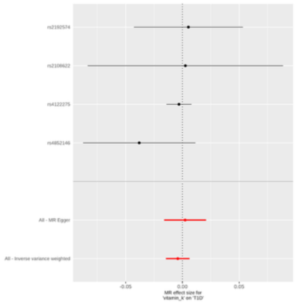

(F)

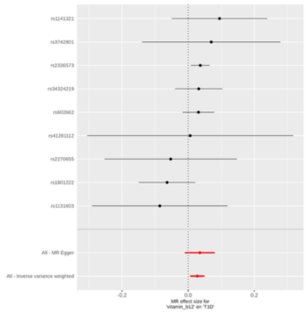

(G)

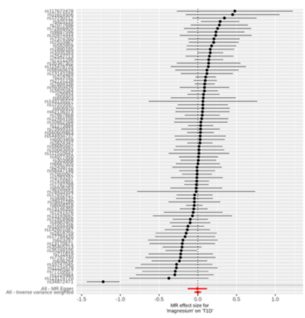

(H)

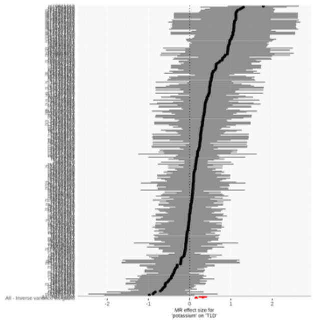

(I)

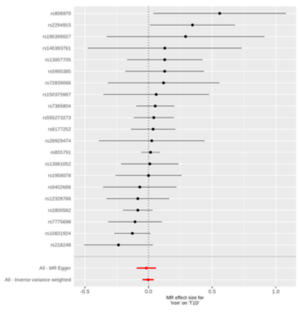

(J)

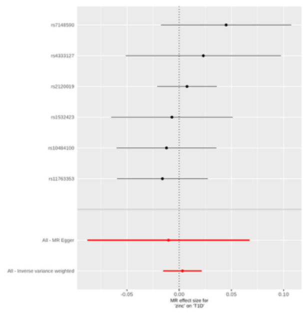

(K)

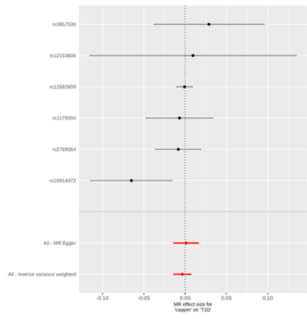

(L)

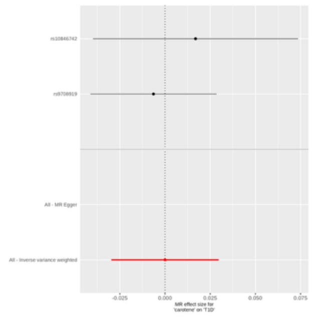

5. <u>Sakaue, S. (East asian T1D)</u>

(A)

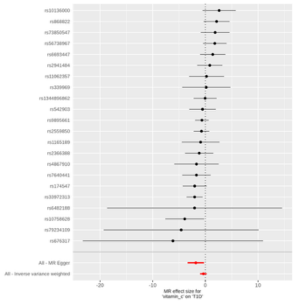

(B)

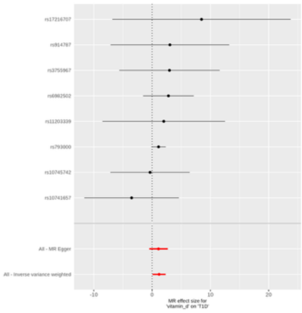

(C)

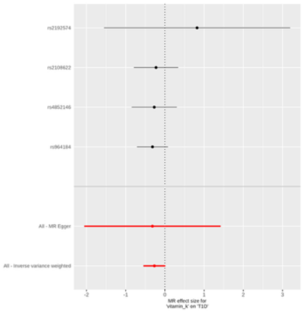

(D)

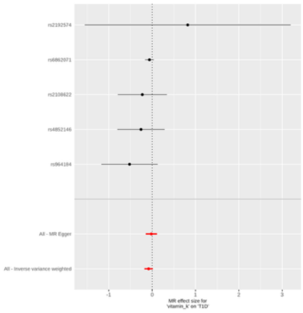

(E)

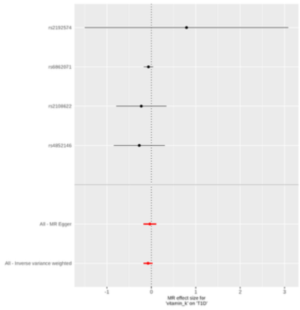

(F)

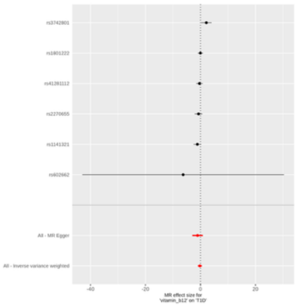

(G)

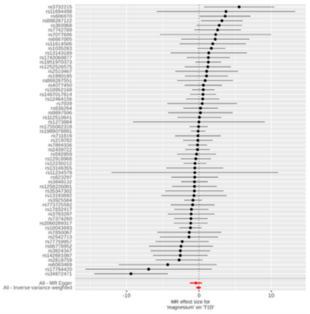

(H)

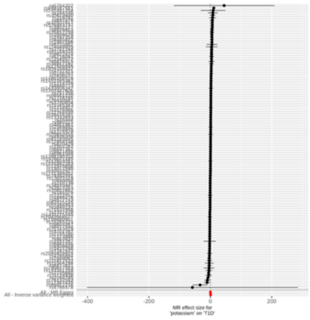

(I)

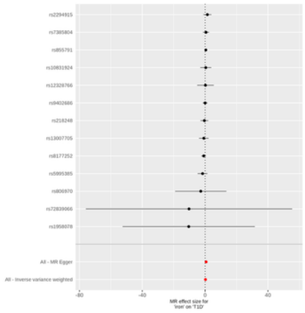

(J)

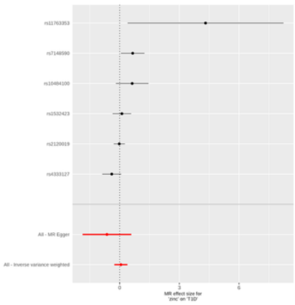

(K)

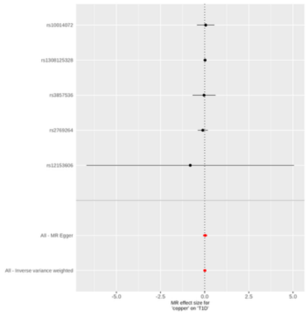

(L)

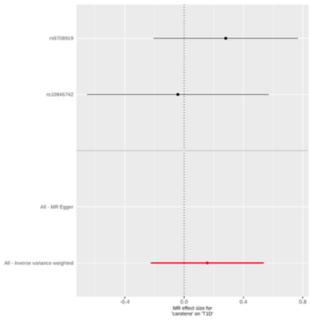

(A) Vitamin C; (B) Vitamin D; (C) Vitamin K 1st model; (D) Vitamin K 2nd model; (E) Vitamin K 3rd model; (F) Vitamin B12; (G) Magnesium; (H) Potassium; (I); Iron; (J) Zinc; (K) Copper; (L) Carotene, (M) Folate

Figures for retinol, vitamin B6, alpha-, beta-, and gamma-tocopherol, selenium, and folate (for African American/Afro-Carribean and East Asian T1D GWAS) are not shown due to insufficient number of SNPs.

Supplemental Figure S3: Leave-one-out sensitivity analysis plot of MR analysis for micronutrients on the T1D outcomes

1. <u>Chiou, J. (European T1D)</u>

(A)

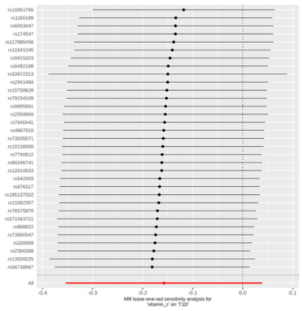

(B)

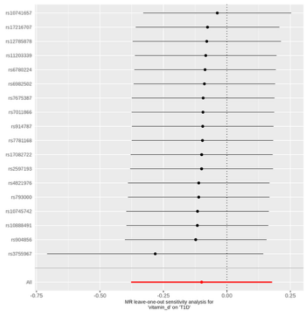

(C)

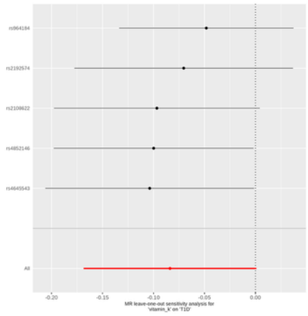

(D)

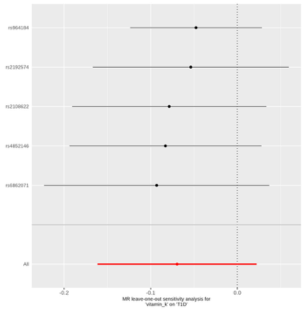

(E)

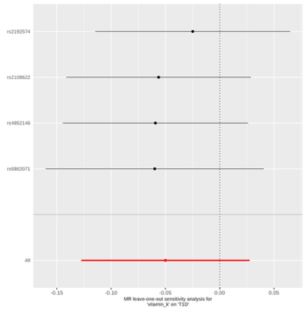

(F)

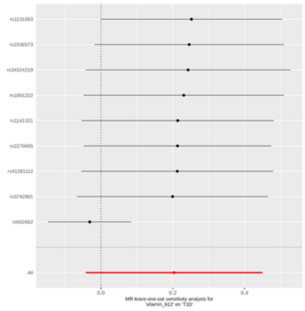

(G)

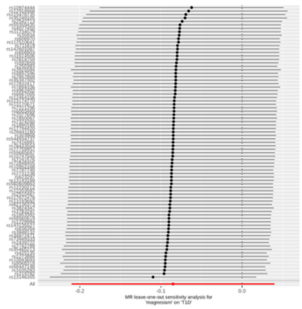

(H)

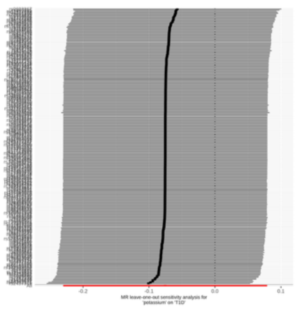

(I)

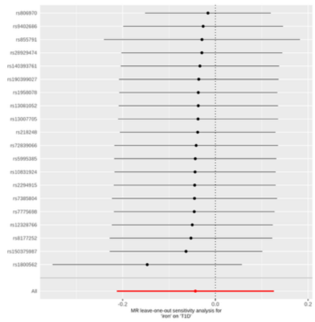

(J)

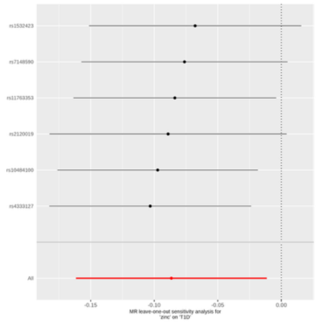

(K)

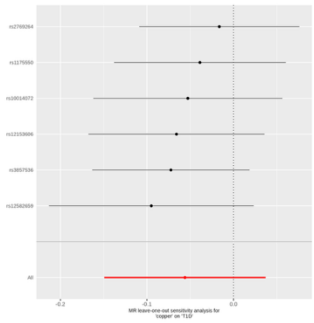

2. Verma, A. (Multi-ancestry T1D)

(A)

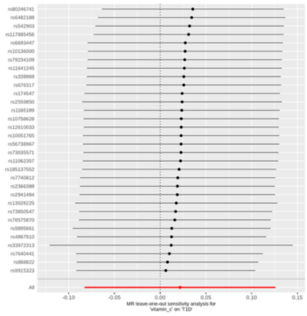

(B)

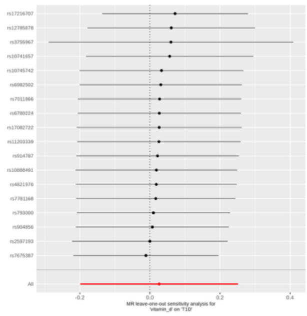

(C)

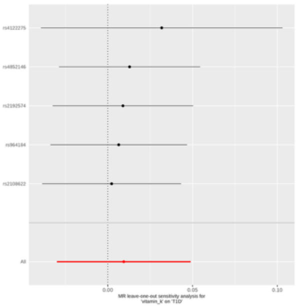

(D)

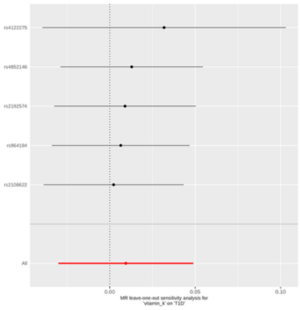

(E)

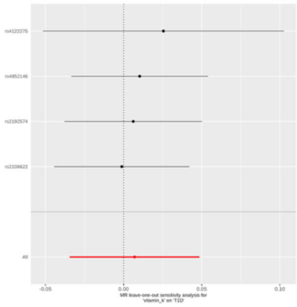

(F)

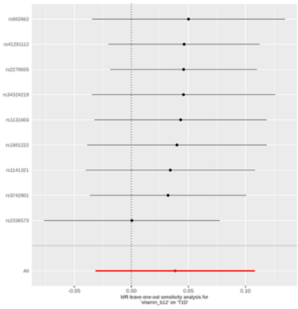

(G)

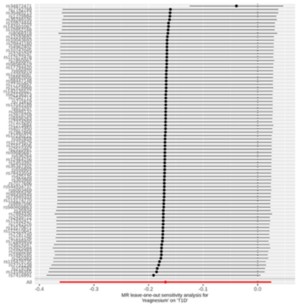

(H)

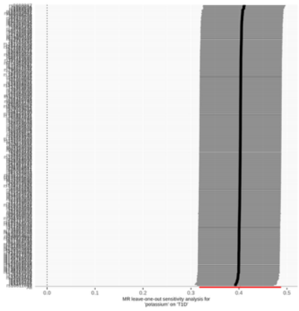

(I)

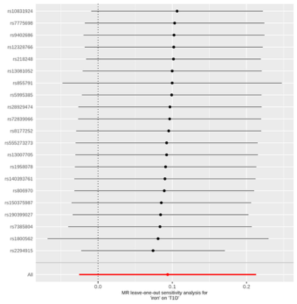

(J)

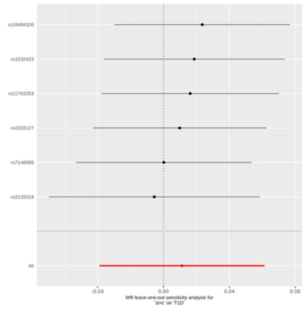

(K)

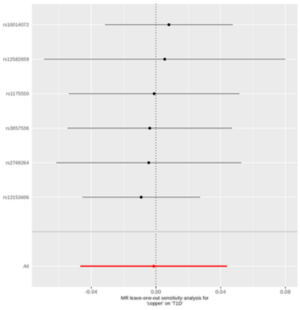

3. Verma, A. (Hispanic/Latin American T1D)

(A)

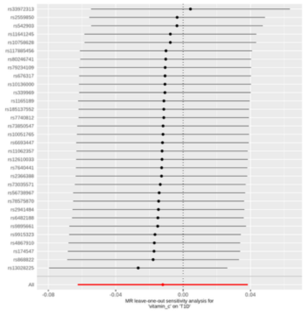

(B)

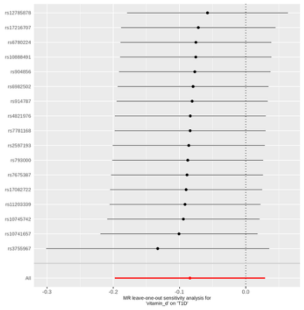

(C)

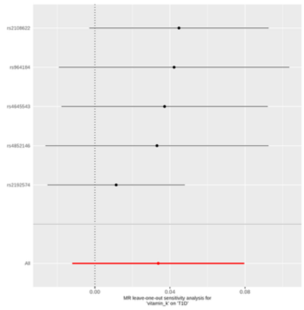

(D)

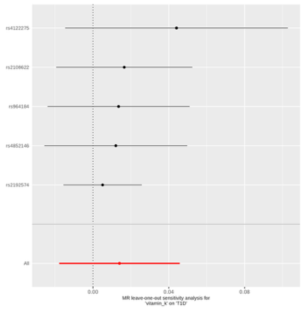

(E)

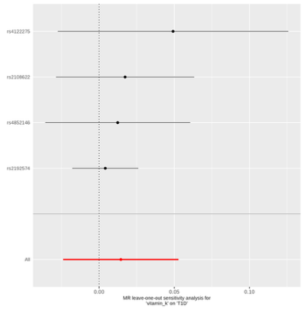

(F)

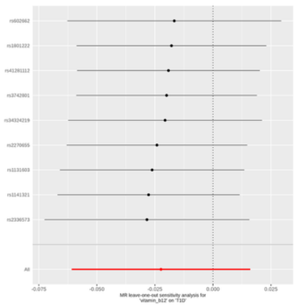

(G)

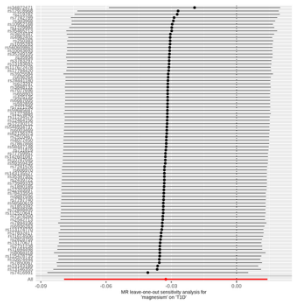

(H)

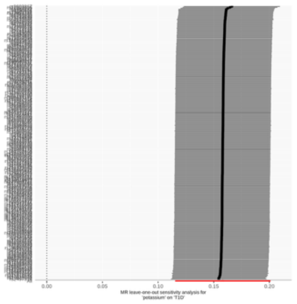

(I)

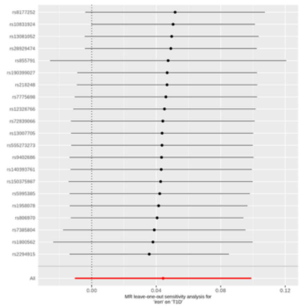

(J)

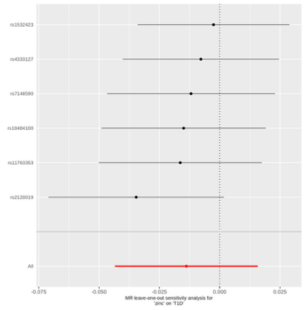

(K)

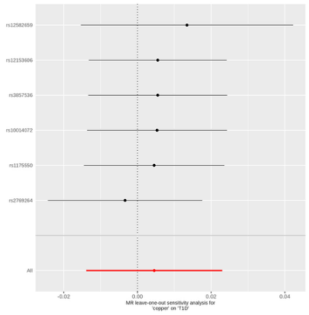

4. Verma, A. (African American/Afro-Carribean T1D)

(A)

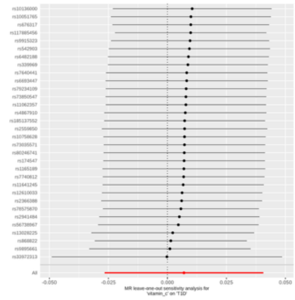

(B)

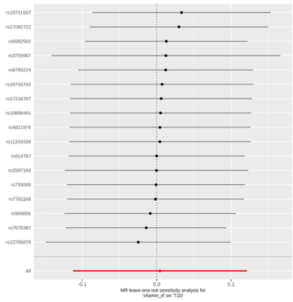

(C)

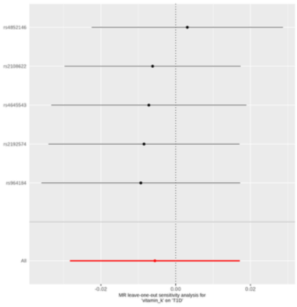

(D)

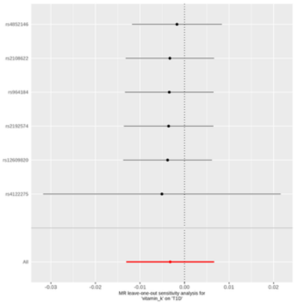

(E)

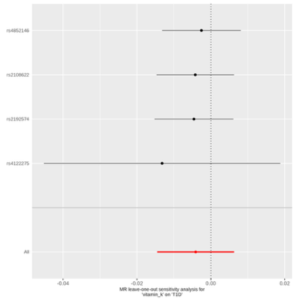

(F)

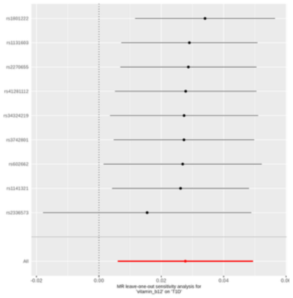

(G)

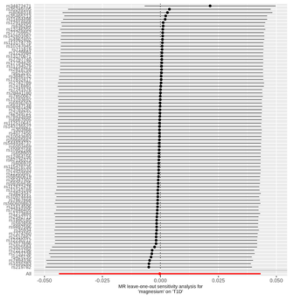

(H)

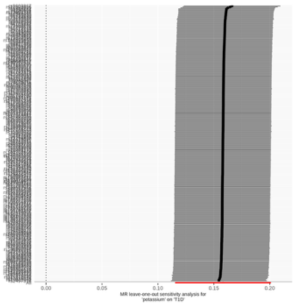

(I)

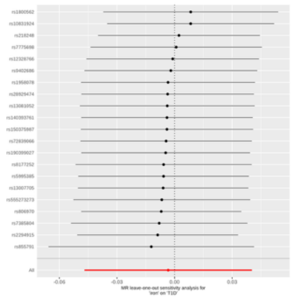

(J)

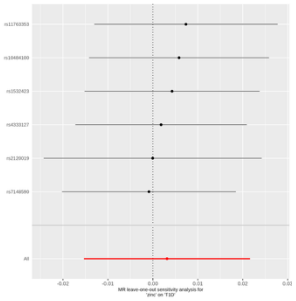

(K)

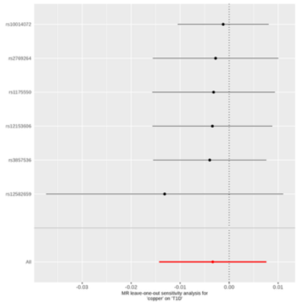

5. Sakaue, S. (East asian T1D)

(A) (B) (C) (D)

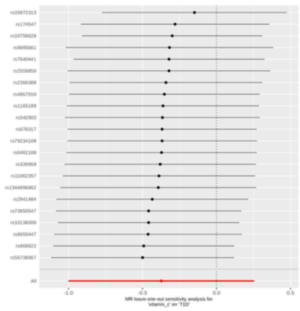
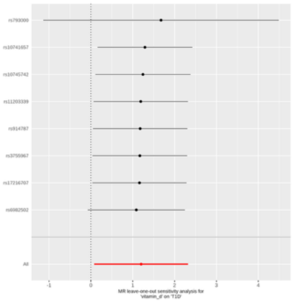
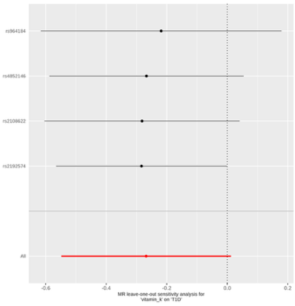
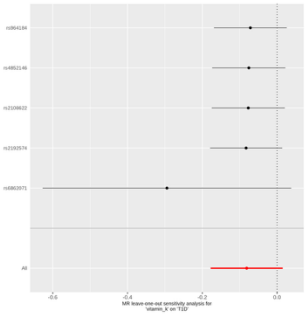

(E) (F) (G) (H)

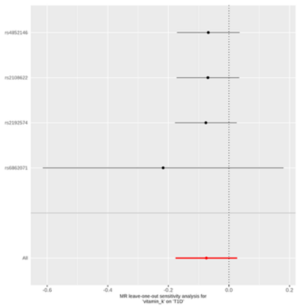
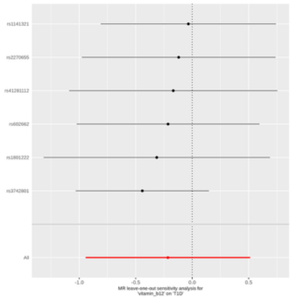
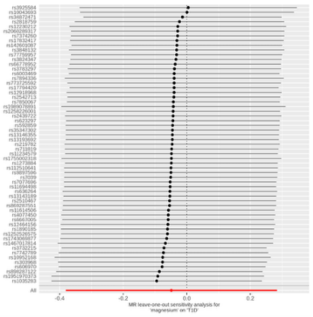
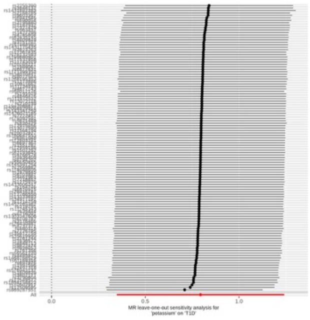

(I) (J) (K)

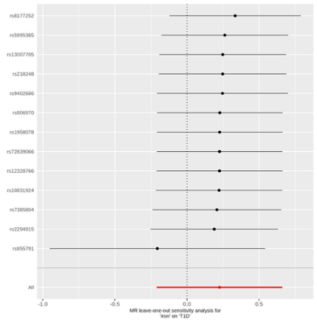
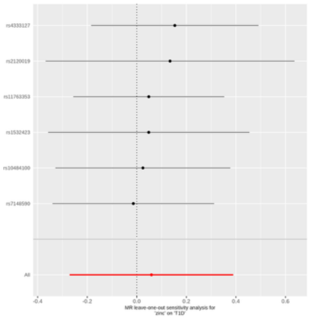
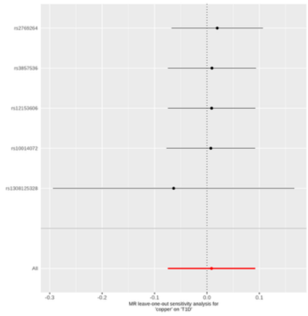

(A) Vitamin C; (B) Vitamin D; (C) Vitamin K 1st model; (D) Vitamin K 2nd model; (E) Vitamin K 3rd model; (F) Vitamin B12; (G) Magnesium; (H) Potassium; (I); Iron; (J) Zinc; (K) Copper

Figures for vitamin retinol, B6, alpha-, beta-, and gamma-tocopherol, selenium, carotene, and folate are not shown due to insufficient numbers of SNPs.
